# Design, Synthesis, and Antifungal Activities of Phenylpyrrole Analogues Based on Alkaloid Lycogalic Acid

**DOI:** 10.3390/molecules29133150

**Published:** 2024-07-02

**Authors:** Shuaiheng Zhang, Zhenghong Zhou, Tienan Wang, Aidang Lu

**Affiliations:** 1 School of Chemical Engineering and Technology, Hebei University of Technology, Tianjin 300401, China; 202231504078@stu.hebut.edu.cn; 2 State Key Laboratory of Elemento-Organic Chemistry, Research Institute of Elemento-Organic Chemistry, College of Chemistry, Nankai University, Tianjin 300071, China; z.h.zhou@nankai.edu.cn; 3 Key Laboratory of Traditional Chinese Medicine Research and Development of Hebei Province, Institute of Traditional Chinese Medicine, Chengde Medical University, Chengde 067000, China

**Keywords:** natural product, lycogalic acid, alkaloid, phenylpyrrole analogues, fungicidal activity, *Rhizoctonia cerealis*, molecular docking

## Abstract

Plant diseases caused by pathogenic fungi seriously affect the yield and quality of crops, cause huge economic losses, and pose a considerable threat to global food security. Phenylpyrrole analogues were designed and synthesized based on alkaloid lycogalic acid. All target compounds were characterized by ^1^H NMR, ^13^C NMR, and HRMS. Their antifungal activities against seven kinds of phytopathogenic fungi were evaluated. The results revealed that most compounds had broad-spectrum fungicidal activities at 50 μg/mL; 14 compounds displayed more than 60% fungicidal activities against *Rhizoctonia cerealis* and *Sclerotinia sclerotiorum*, and in particular, the fungicidal activities of compounds **8g** and **8h** against *Rhizoctonia cerealis* were more than 90%, which could be further developed as lead agents for water-soluble fungicides. The molecular docking results indicate that compounds **8g** and **8h** can interact with 14α-demethylase (*Rc*CYP51) through hydrogen bonding with strong affinity.

## 1. Introduction

Infectious plant diseases caused by plant fungi, bacteria, and viruses seriously affect crop yield and quality. At least 16–20% of global food production is lost due to plant diseases every year, and some crops even suffer losses of up to 30–50% annually [1,2,3]. In particular, diseases caused by plant pathogenic fungi have the widest impact and the greatest harm, accounting for about two-thirds of plant diseases, posing a serious threat to the safety and stability of crop production [4,5], and causing approximately USD 220 billion in economic losses worldwide every year [3,6]. For example, *Rhizoctonia cerealis* (*R. cerealis*), which infects the leaf sheaths and stems of wheat, has a serious impact on growth and yield of wheat [7,8]. *R. cerealis* is prevalent in wheat growing areas all over the world. The incidence of the disease can reach 10–30%, even 60–80% in some seriously affected areas, with more than 20% of wheat ears withered due to disease in particularly serious areas. In China, the annual loss accounts for about 10% of the total wheat yield, and in serious cases, it can reach 30–40%. More than 6.67 million hectares of wheat are threatened by *R. cerealis* every year [9,10,11]. Therefore, the prevention and control of plant pathogenic fungi has become one of the most serious challenges in agricultural development and plant protection; reducing the risk of crop diseases is essential for global food security, which not only requires the effective control of plant pathogens in different cropping systems around the world, but also needs to achieve this goal without damaging natural ecosystems [12,13], so it is of great significance to develop new antifungal agents.

**Figure 1:**
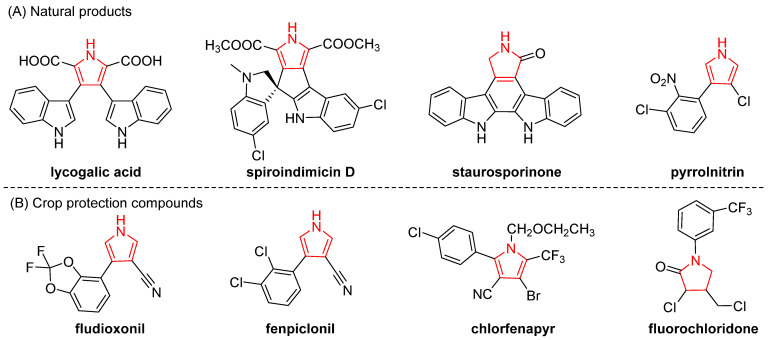
Some natural products (**A**) and crop protection compounds (**B**) containing pyrrole fragments.

The discovery of lead structure is one of the key scientific problems in pesticide discovery; it is an effective way to discover new pesticide molecules with novel skeleton and special mechanisms of action by using active natural products as the precursor [14,15,16]. Natural products are the main or secondary metabolites produced by animals, plants, or microorganisms, which are characterized by their diverse biological activities, low toxicity, and good environmental compatibility; however, most of the natural products have some disadvantages, such as complex chemical structure, difficulty synthesizing, instability in light, and so on. Therefore, the development of new bio-inspired pesticides based on natural products can not only improve the specificity of efficacy, but also provide a practical and powerful strategy to overcome these limitations [15,16,17,18]. Pyrrole is widely known as a bioactive scaffold with multiple activities. The combination of different pharmacophores in the pyrrole ring system leads to the formation of more active compounds. Pyrrole-containing analogs are considered potential sources of bioactive compounds, which can be found in lycogalic acid, spiroindimicin D, staurosporinone, pyrrolnitrin, and other natural products, with a series of important beneficial properties, such as insecticidal, bactericidal, herbicidal, and so on. Some phenylpyrrole drugs have been used in crop protection, such as fludioxonil, fenpiclonil, chlorfenapyr, and fluorochloridone, etc. [19,20,21,22,23] (Figure 1).

Pyrrole-containing compounds, as one of the sterol biosynthesis demethylation inhibitors, have been playing a crucial role in the development of fungicides. They could inhibit the 14*α*-demethylated eburicolin sterol biosynthesis by interfering with the cytochrome P450 system then destroy the fungal cell membrane and affect fungal growth [24]. Therefore, lanosterol 14α-demethylase (CYP51) has become a classical target for novel fungicide development. However, compared to other nitrogen-containing heterocyclic structures such as pyrazole, triazole, pyridine, pyrimidine, etc., there are relatively fewer pesticide varieties of pyrrole compounds, which provides researchers with broader research space [25,26,27].

Alkaloid lycogalic acid, also known as chromophoric acid, is a natural product of two indoles linked by a pyrrole structure. It is not only the intermediate in the biosynthesis of indole [2,3-*a*] carbazole alkaloids, but also has inhibitory effects on various enzymes such as protein kinases, topoisomerases, and cytochrome enzymes [28,29,30,31]. Hydrogen bonding plays an important role in the interaction between small organic molecules and target proteins, so increasing the number of hydrogen bonding receptors or donors is one of the important methods to increase the interaction with target proteins and enhance their biological activity [32]. Lycogalic acid contains three NH and two COOH functional groups in its molecular structure, which are excellent electron donors and acceptors. Moreover, the two carboxylic acid functional groups can greatly improve water solubility, which has an important impact on biological activity, and its enzyme inhibition is also expected to be a potential antibacterial molecule.

**Figure 2:**
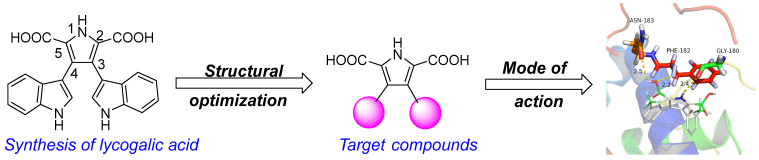
Design of lycogalic acid analogues containing the phenylpyrrole structure.

In this study, the alkaloid lycogalic acid was used as a lead compound, retaining its pyrrole carboxylic acid moiety; structural optimization was achieved by changing the indole structure, and a series of lycogalic acid analogues containing a phenylpyrrole fragment were designed and synthesized (Figure 2). Their bactericidal activities against seven kinds of phytopathogenic fungi were evaluated at the concentration of 50 μg/mL, and the mechanisms of action of the selected high-efficiency compounds were preliminarily evaluated by means of molecular docking.

## 2. Results

### 2.1. Chemicals

**Scheme 1:**
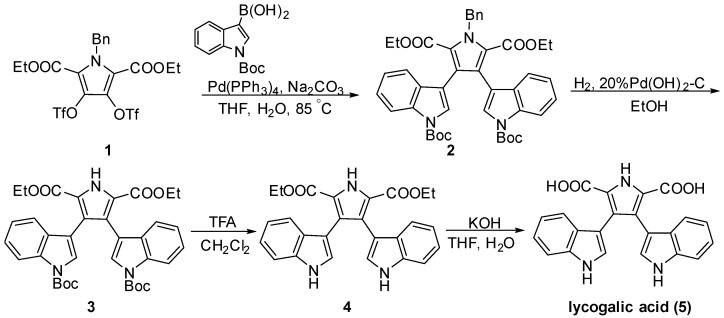
Synthesis of compound **5**.

**Scheme 2:**
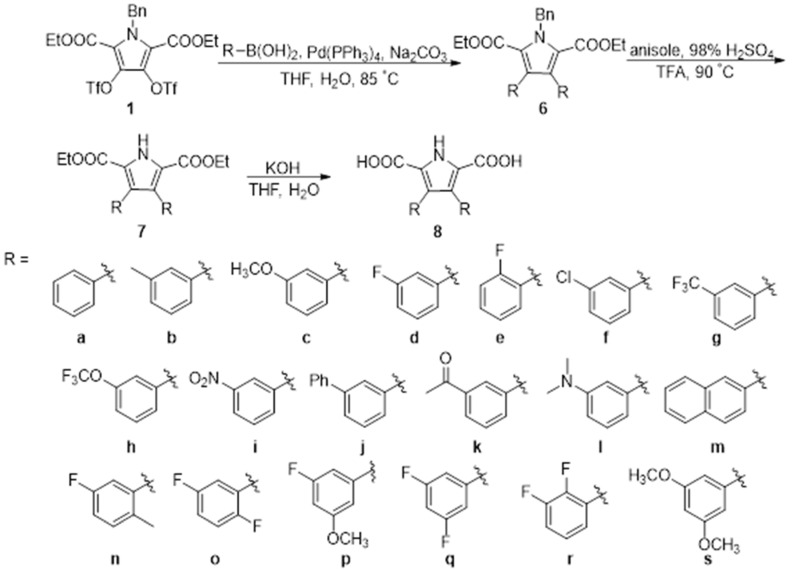
Synthesis o compounds **8a**–**8s**.

The synthesis of compound **1** was performed as shown in Scheme S1, and natural product lycogalic acid (**5**) was obtained following an established method [33]; the key reaction was palladium-catalyzed Suzuki–Miyaura coupling of bis-triflate derivative and indolboronic acid derivative (Scheme 1). For the application of these compounds as novel plant disease protective agents, it is necessary not only to determine their activity and the mode of action but also to obtain these products in multi-gram quantities with simple or no purification procedures. Phenylpyrrole analogues have been designed using simple arylboronic acid as the starting material. The essential intermediate **1** reacted with different substituted arylboronic acids and then benzyl groups were removed and hydrolyzed to afford compounds **8a**–**8s** with satisfactory yields (Scheme 2). All target compounds were confirmed by ^1^H NMR, ^13^C NMR, and HRMS. The copies of spectra are provided in the Supplementary Materials. 

### 2.2. In Vitro Antifungal Activities of Target Compounds ***5*** and ***8a***–***8s***

Using carbendazim as control, the inhibitory effects of compounds **5** and **8a**–**8s** on seven common phytopathogenic fungi at 50 μg/mL were tested by fungicidal growth rate assay. According to the results in Table 1, compounds **8i** and **8r** exhibited higher antifungal activities against *A. solani* than commercial fungicide carbendazim; most of the synthesized target compounds showed inhibition rates higher than 60% against *R. cerealis* and *S. sclerotiorum*. The inhibition rates of compounds **8g** and **8h** against *R. cerealis* reached 92% and 91%, respectively.

### 2.3. Molecular Docking Research

To further elucidate the possible mechanism of the interaction between designed phenylpyrrole analogues and *Rc*CYP51, AutoDock Vina 1.1.2 was used for molecular docking [34]. It can be proven that there are some H-bond interactions and strong binding affinity between phenylpyrrole analogues **8g** and **8h** containing carboxylic acid and *Rc*CYP51.

## 3. Discussion

### 3.1. Synthesis

According to a similar approach reported, the NH of diethyl 2,2′-azanediyldiacetate was protected with benzyl bromide in the presence of K_2_CO_3_ to obtain compound **S2**, then treated with diethyl oxalate via Hinsberg reaction, and the product reacted with trifluoromethanesulfonic anhydride to gain the key intermediate **1**. Although palladium-catalyzed Suzuki coupling of bis-triflate derivative and indolboronic acid derivatives was achieved easily according to the literature [33], many attempts to provide the expected compound **2** failed. Fortunately, the cyclization proceeded smoothly under conditions using a water/THF mixture (2:3, *v*:*v*), and compound **2** was obtained with a yield increased from 64% to 87%. Compound **3** was obtained by debenzylation of compound **2** under mild conditions with a quantitative yield. Treatment of compound **3** with TFA in CH_2_Cl_2_, and then followed by hydrolysis afforded lycogalic acid (**5**). Starting from intermediate **1**, a series of reactions by Suzuki coupling, the removal of the benzyl group, and hydrolysis were performed and compound **8a**–**8s** can be successfully obtained to achieve structural diversity in the derivation of lycogalic acid.

### 3.2. Structure–Activity Relationship (SAR) Analysis for the Antifungal Activity

The in vitro antifungal activities of compounds **5** and **8a**–**8s** were evaluated compared to the commercialized fungicide carbendazim. As shown in Table 1, lycogalic acid and its analogues exhibited broad-spectrum fungicidal activities against seven kinds of phytopathogenic fungi at 50 μg/mL; most target compounds showed poor antifungal activities against *C. arachidicola hori* and *A. solani*, and only compound **8i** and compound **8j** displayed moderate fungicidal activities (more than 60% inhibition rate) against *F. oxysporium* f. sp. *Cucumeris.* The natural product lycogalic acid (**5**) displayed moderate fungicidal activities against *S. sclerotiorum,* with inhibition rates of 75%. After replacing the indole structure with the benzene ring, the bactericidal activity decreased in different degrees (inhibition rate: **5** > **8a**); however, the introduction of electron-withdrawing groups or electron-donating groups on the 3-position of the phenyl group was conducive to the improvement of antifungal activity against *C. arachidicola hori*, *P. piricola*, *R. cerealis*, and *S. sclerotiorum*; in particular, the substitution of the −CF_3_ or −OCF_3_ on the 3-position of the phenyl group resulted in a sharp increase in inhibitory activity against *R. cerealis*, and compounds **8g** and **8h** showed excellent fungicidal activities against *R. cereal,* with inhibition rates of 92% and 91% at 50 μg/mL. Compounds **8d** and **8e** exhibited a different level of antifungal activities, which indicated that the position of the substituent group of the benzene ring had different effects on the activity, and the introduction of −F at the 3-position of the benzene ring was beneficial for improving the activity against *P. grisea* and *S. sclerotiorum*. However, the introduction of −F at the 2-position of the benzene ring was conducive to the improvement of antiviral activity against *F. oxysporium* f. sp. *Cucumeris*, and *C. arachidicola hori*. By comparing **8d**, **8n**, and **8q**, it can be found that the effects of the types and position of substituent groups on the benzene ring are not the same; the introduction of −CH_3_ into the 5-position, and −F into the 4-position was more effective against *F. oxysporium* f. sp. *Cucumeri*, *C. arachidicola hori*, *P. piricola*, and the electron-withdrawing group had a better inhibitory effect; however, it will reduce the activity against *P. grisea*.

### 3.3. Molecular Docking

**Figure 3:**
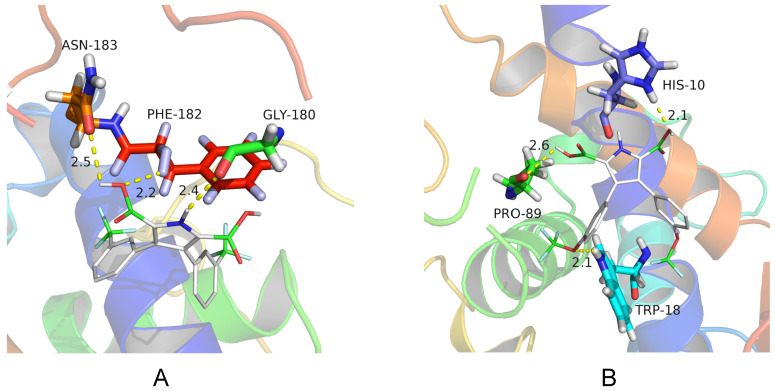
Molecule docking results of **8g** (**A**) and **8h** (**B**) with *Rc*CYP51.

To further elucidate the possible mechanism of the designed compounds at the molecular level, representative compounds **8g**, **8h**, and *Rc*CYP51 were selected as ligands and the receptor for molecular docking research using AutoDock-vina 1.1.2.35. The calculation procedures for molecular docking research were carried out according to the literature and are described in the Supplementary Materials [35]. The main hydrogen bonds between the compound and the amino acid residues are shown in Figure 3. The results showed that compound **8g** was laid into the *Rc*CYP51 active pocket of ASN-183, PHE-182, and GLY-180 (Figure 3A). The H of the carboxyl group formed a hydrogen bond (2.5 Å) with the oxygen of amino acid residue ASN-183, the oxygen of the hydroxyl group formed a conventional hydrogen bond (2.2 Å) with the hydrogen of amino acid residue PHE-182, and the H of the pyrrole formed a hydrogen bond (2.4 Å) with the oxygen of amino acid residue GLY-180. Compound **8h** can provide O-H and O atoms to interact with amino acid residues and form intermolecular hydrogen bonds with the active sites of PRO-89 (2.6 Å) and HIS-10 (2.1 Å). Compared with compound **8g**, the trifluoromethoxy group of compound **8h** can bind to TRP-18 (2.1 Å) (Figure 3B). The binding free energies of compounds **8g** and **8h** to *Rc*CYP51 were −7.5 kcal/mol and −7.1 kcal/mol, respectively. The lower the binding free energy, the higher the affinity between the receptor and the ligand [36]. The results of molecular docking showed that lycogalic acid analogues had a strong interaction with *Rc*CYP51, and the interaction sites and strength were greatly affected by the molecular structure.

## 4. Materials and Methods

### 4.1. Chemicals and Instruments

All reagents used were analytical reagent grade or chemically pure, purchased from commercial sources (Tianjin Guangda Chemical Reagents Ltd., Tianjin, China). The melting points of the target compounds were measured on an X-4 binocular microscope (Beijing Zhongke Instrument Co., Ltd., Beijing, China). Nuclear magnetic resonance (NMR) spectra were acquired with a 400 MHz (100 MHz for ^13^C) instrument (Bruker, Billerica, MA, USA) at room temperature. Chemical shifts were measured relative to residual solvent peaks of CDCl_3_ (^1^H: *δ* = 7.26 ppm; ^13^C: *δ* = 77.0 ppm) and DMSO-*d*_6_ (^1^H: *δ* = 2.5 ppm; ^13^C: *δ* = 39.9 ppm) as internal standards. The following abbreviations are used to designate chemical shift multiplicities: s = singlet, d = doublet, dd = doublet of doublets, t = triplet, m = multiplet, and brs = broad singlet. High-resolution mass spectrometry (HRMS) data were recorded with a quadrupol Fourier transform–electrospray ionization instrument (Varian, Palo Alto, CA, USA). Compound **1** was prepared according to the methods reported by [33]; the detailed synthetic procedure can be seen in the Supplementary Materials.

### 4.2. Synthetic Procedures

#### 4.2.1. Synthesis of Compound **2**–**5**

Diethyl 1-benzyl-3,4-bis(1-(*tert*-butoxycarbonyl)-1*H*-indol-3-yl)-1*H*-pyrrole-2,5-dicarboxylate (**2**). Compound **1** (2.4 g, 4.0 mmol, 1.0 equiv.), (1-(*tert*-butoxycarbonyl)-1*H*-indol-3-yl)boronic acid (3.1 g, 11.9 mmol, 3.0 equiv.), tetrakis(triphenylphosphine)palladium (0.37 g, 0.32 mmol, 0.08 equiv.), sodium carbonate (6.7 g, 63.5 mmol, 16.0 equiv.), and water (30 mL) were added to tetrahydrofuran (THF) (30 mL); the reaction mixture was stirred for 6 h at 85 °C under a nitrogen atmosphere. After the completion of the reaction, the solvent was removed in vacuum, and extracted with ethyl acetate (EtOAc) (3 × 20 mL). The combined organic phases were dried and the solvent was evaporated. The crude product was purified by column chromatography (*V*_PE_:*V*_EA_ = 15:1–4:1) to obtain compound **2**. Yellow liquid, 87% yield; ^1^H NMR (400 MHz, CDCl_3_) *δ* 8.08 (d, *J* = 8.2 Hz, 2H), 7.34 (t, *J* = 7.5 Hz, 2H), 7.22 (d, *J* = 11.1 Hz, 7H), 7.13 (d, *J* = 9.9 Hz, 4H), 6.22 (s, 2H), 3.87 (q, *J* = 7.2 Hz, 4H), 1.55 (s, 18H), 0.55 (t, *J* = 7.1 Hz, 6H); ^13^C NMR (100 MHz, CDCl_3_) *δ* 161.4, 149.6, 138.8, 134.8, 131.5, 128.6, 127.1, 126.4, 126.3, 124.7, 124.0, 122.9, 122.5, 119.8, 114.9, 114.4, 83.4, 60.8, 49.7, 28.1, 12.9.

Diethyl 3,4-bis(1-(*tert*-butoxycarbonyl)-1*H*-indol-3-yl)-1*H*-pyrrole-2,5-dicarboxylate (**3**). The (Pd(OH)_2_, (0.4 g, 20% *w*/*w*) was added to a stirred solution of compound **2** (1.8 g, 2.5 mmol, 1.0 equiv.) in dry ethanol (30 mL). The nitrogen atmosphere was replaced by hydrogen. The resulting mixture was stirred at room temperature until the raw materials disappeared, then filtered through Celite, and concentrated in vacuo to obtain compound **3**. Yellow liquid, 87% yield; ^1^H NMR (400 MHz, CDCl_3_) *δ* 10.17 (s, 1H), 8.10 (d, *J* = 8.2 Hz, 2H), 7.38 (s, 2H), 7.26 (t, *J* = 7.7 Hz, 4H), 7.13 (d, *J* = 8.0 Hz, 2H), 4.19 (q, *J* = 7.1 Hz, 4H), 1.61 (s, 18H), 1.00 (t, *J* = 7.1 Hz, 6H); ^13^C NMR (100 MHz, CDCl_3_) *δ* 160.4, 149.6, 134.8, 125.4, 124.0, 123.6, 122.9, 122.4, 120.2, 114.9, 113.3, 83.5, 61.0, 28.2, 13.7.

Diethyl 3,4-di(1*H*-indol-3-yl)-1*H*-pyrrole-2,5-dicarboxylate (**4**). To a stirred solution of compound **3** (0.83 g, 1.3 mmol, 1.0 equiv.) in CH_2_Cl_2_ (30 mL), trifluoroacetic acid (TFA) (10 mL) was added and the reaction mixture was stirred at room temperature for 2 h. After completion of the reaction, saturated NaHCO_3_ solution was added dropwise to the reaction mixture to adjust the pH to 7–8, extracted with CH_2_Cl_2_ (3 × 20 mL). The combined organic phase was dried with anhydrous sodium sulfate. The crude product was obtained after removing the solvent in vacuo and purified by column chromatography (*V*_PE_:*V*_EA_ = 3:1) to obtain compound **4**. Brown solid, 98% yield, m.p. 123–124 °C (lit. [37] 119−120 °C); ^1^H NMR (400 MHz, CDCl_3_) *δ* 9.97 (s, 1H), 7.76 (d, *J* = 6.8 Hz, 2H), 7.28 (d, *J* = 9.3 Hz, 2H), 7.13 (t, *J* = 7.2 Hz, 2H), 7.06 (t, *J* = 7.5 Hz, 2H), 6.97 (t, *J* = 7.5 Hz, 2H), 6.62 (d, *J* = 10.1 Hz, 2H), 4.14 (q, *J* = 7.1 Hz, 4H), 0.95 (t, *J* = 7.1 Hz, 6H); ^13^C NMR (100 MHz, CDCl_3_) *δ* 160.8, 135.5, 128.0, 125.0, 124.4, 122.9, 121.5, 120.3, 119.3, 111.0, 108.8, 60.8, 13.8.

Lycogalic acid (**5**). To a stirred solution of compound **4** (0.22 g, 0.50 mmol, 1.0 equiv.) in THF (15 mL), KOH (0.19 g, 3.4 mmol, 7.0 equiv.) and water (15 mL) were added; the mixture was stirred at 80 °C for 6 h. After the reaction was completed, the THF was removed in vacuum, and dilute HCl (1 M) was added to adjust the pH to 4–6; the aqueous phase was extracted with ethyl acetate (3 × 15 mL) and the organic phases were combined, dried with anhydrous sodium sulfate, and filtered. The crude product after evaporation was purified by column chromatography (*V*_DCM_:*V*_MeOH_ = 10:1) to obtain compound **5**. Brown solid, 99% yield, m.p. 184–186 °C (lit. [38] 186−188 °C); ^1^H NMR (400 MHz, DMSO-*d*_6_) *δ* 12.17 (s, 2H), 11.54 (s, 1H), 10.80 (d, *J* = 2.6 Hz, 2H), 7.23 (d, *J* = 8.1 Hz, 2H), 7.08 (d, *J* = 8.0 Hz, 2H), 7.01 (d, *J* = 2.4 Hz, 2H), 6.94 (t, *J* = 7.5 Hz, 2H), 6.78 (t, *J* = 7.5 Hz, 2H); ^13^C NMR (100 MHz, DMSO-*d*_6_) *δ* 162.2, 135.9, 128.2, 125.4, 124.6, 123.8, 120.8, 119.9, 118.8, 111.6, 108.6; HRMS (ESI): *m*/*z* [M + NH_4_]^+^ calcd for C_22_H_15_N_3_O_4_: 403.1401; found: 403.1404.

#### 4.2.2. Synthesis of Compounds **6**–**8**

The synthesis procedures of compounds **6a**–**6s** were the same as compound **2**.

Diethyl 1-benzyl-3,4-diphenyl-1*H*-pyrrole-2,5-dicarboxylate (**6a**). White solid, 84% yield, m.p. 110–113 °C (lit. [39] 117−118 °C); ^1^H NMR (400 MHz, CDCl_3_) *δ* 7.77–6.93 (m, 15H), 6.12 (s, 2H), 4.37 (q, *J* = 7.1 Hz, 2H), 4.02 (q, *J* = 7.2 Hz, 2H), 1.35 (t, *J* = 7.1 Hz, 3H), 0.89 (t, *J* = 7.1 Hz, 3H); ^13^C NMR (100 MHz, CDCl_3_) *δ* 160.0, 158.9, 137.4, 135.6, 133.9, 130.8, 130.4, 130.1, 128.7, 127.5, 126.2, 124.7, 124.7, 124.7, 124.6, 119.5, 118.8, 116.3, 62.0, 61.5, 49.8, 13.9, 13.3.

Diethyl 1-benzyl-3,4-di-*m*-tolyl-1*H*-pyrrole-2,5-dicarboxylate (**6b**). White solid, 86% yield, m.p. 71–74 °C; ^1^H NMR (400 MHz, CDCl_3_) *δ* 7.31 (t, *J* = 7.3 Hz, 2H), 7.22 (t, *J* = 7.3 Hz, 1H), 7.13 (d, *J* = 7.5 Hz, 2H), 7.05 (t, *J* = 7.5 Hz, 2H), 6.96 (d, *J* = 7.7 Hz, 2H), 6.91 (s, 2H), 6.84 (d, *J* = 7.5 Hz, 2H), 6.06 (s, 2H), 3.99 (q, *J* = 7.0 Hz, 4H), 2.23 (s, 6H), 0.84 (t, *J* = 7.1 Hz, 6H); ^13^C NMR (100 MHz, CDCl_3_) *δ* 161.8, 138.8, 136.4, 134.4, 131.3, 128.5, 127.5, 127.2, 127.1, 127.0, 126.4, 124.6, 60.5, 49.4, 21.4, 13.4.

Diethyl 1-benzyl-3,4-bis(3-methoxyphenyl)-1*H*-pyrrole-2,5-dicarboxylate (**6c**). White solid, 99% yield, m.p. 66–69 °C; ^1^H NMR (400 MHz, CDCl_3_) *δ* 7.31 (t, *J* = 7.2 Hz, 2H), 7.23 (t, *J* = 7.3 Hz, 1H), 7.17–7.05 (m, 4H), 6.77–6.63 (m, 4H), 6.61 (s, 2H), 6.06 (s, 2H), 4.00 (q, *J* = 7.1 Hz, 4H), 3.64 (s, 6H), 0.86 (t, *J* = 7.1 Hz, 6H); ^13^C NMR (100 MHz, CDCl_3_) *δ* 161.6, 158.6, 138.6, 135.7, 130.9, 130.1, 128.5, 128.2, 127.0, 126.4, 124.5, 123.1, 115.7, 112.5, 107.7, 106.3, 101.4, 60.7, 55.1, 49.4, 13.4.

Diethyl 1-benzyl-3,4-bis(3-fluorophenyl)-1*H*-pyrrole-2,5-dicarboxylate (**6d**). White solid, 61% yield, m.p. 82–85 °C; ^1^H NMR (400 MHz, CDCl_3_) *δ* 7.32 (t, *J* = 7.4 Hz, 2H), 7.23 (d, *J* = 7.4 Hz, 1H), 7.14 (dd, *J* = 14.3, 8.1 Hz, 4H), 6.98–6.69 (m, 6H), 6.10 (s, 2H), 4.01 (q, *J* = 7.1 Hz, 4H), 0.87 (t, *J* = 7.1 Hz, 6H); ^13^C NMR (100 MHz, CDCl_3_) *δ* 163.2, 161.2, 160.8, 138.4, 136.5, 136.4, 129.8, 128.8, 128.7, 128.5, 127.1, 126.4, 126.2, 124.6, 117.5, 117.3, 113.7, 113.5, 60.7, 49.5, 13.3.

Diethyl 1-benzyl-3,4-bis(2-fluorophenyl)-1*H*-pyrrole-2,5-dicarboxylate (**6e**). White solid, 96% yield, m.p. 125–127 °C; ^1^H NMR (400 MHz, CDCl_3_) *δ* 7.32 (t, *J* = 7.5 Hz, 2H), 7.27–7.09 (m, 5H), 7.05–6.84 (m, 6H), 6.21 (s, 2H), 4.00 (q, *J* = 7.1 Hz, 4H), 0.84 (t, *J* = 7.1 Hz, 6H); ^13^C NMR (100 MHz, CDCl_3_) *δ* 161.6, 160.9, 159.2, 138.7, 131.9, 128.9, 128.8, 128.5, 127.0, 126.2, 125.4, 125.1, 123.2, 122.7, 122.5, 114.8, 114.6, 60.6, 49.8, 13.3.

Diethyl 1-benzyl-3,4-bis(3-chlorophenyl)-1*H*-pyrrole-2,5-dicarboxylate (**6f**). White solid, 86% yield, m.p. 63–66 °C; ^1^H NMR (400 MHz, CDCl_3_) *δ* 7.32 (t, *J* = 7.4 Hz, 2H), 7.24 (d, *J* = 7.23 Hz, 1H), 7.21–7.00 (m, 8H), 6.89 (d, *J* = 7.6 Hz, 2H), 6.11 (s, 2H), 4.01 (q, *J* = 7.1 Hz, 4H), 0.88 (t, *J* = 7.1 Hz, 6H); ^13^C NMR (100 MHz, CDCl_3_) *δ* 161.1, 138.4, 136.0, 133.1, 130.7, 129.7, 128.5, 127.1, 126.9, 126.4, 124.6, 60.8, 49.5, 13.3.

Diethyl 1-benzyl-3,4-bis(3-(trifluoromethyl)phenyl)-1*H*-pyrrole-2,5-dicarboxylate (**6g**). White solid, 79% yield, m.p. 65–67 °C; ^1^H NMR (400 MHz, CDCl_3_) *δ* 7.47 (d, *J* = 7.8 Hz, 2H), 7.41 (s, 2H), 7.40–7.25 (m, 5H), 7.21 (d, *J* = 8.0 Hz, 2H), 7.17 (d, *J* = 7.6 Hz, 2H), 6.19 (s, 2H), 4.03 (q, *J* = 7.1 Hz, 4H), 0.85 (t, *J* = 7.1 Hz, 6H); ^13^C NMR (100 MHz, CDCl_3_) *δ* 161.0, 138.4, 135.1, 133.7, 129.8, 128.6, 127.9, 127.6, 127.5, 127.3, 126.5, 124.8, 123.6 (d, *J* = 3.7 Hz), 60.9, 49.7, 13.3.

Diethyl 1-benzyl-3,4-bis(3-(trifluoromethoxy)phenyl)-1*H*-pyrrole-2,5-dicarboxylate (**6h**). Brown solid, 33% yield, m.p. 108–110 °C; ^1^H NMR (400 MHz, CDCl_3_) *δ* 7.83 (s, 1H), 7.34 (d, *J* = 7.5 Hz, 1H), 7.28 (s, 1H), 7.22 (t, *J* = 8.0 Hz, 3H), 7.16 (d, *J* = 7.6 Hz, 1H), 7.06 (d, *J* = 8.5 Hz, 1H), 7.01 (d, *J* = 7.7 Hz, 1H), 6.94 (d, *J* = 8.5 Hz, 2H), 6.82–6.72 (m, 2H), 6.14 (s, 1H), 5.79 (s, 2H), 4.34 (q, *J* = 7.1 Hz, 4H), 1.28 (t, *J* = 7.1 Hz, 6H); ^13^C NMR (100 MHz, CDCl_3_) *δ* 162.6, 161.1, 139.7, 136.3, 130.3, 129.0 (d, *J* = 25.7 Hz), 126.5, 125.6, 123.1, 119.4, 114.0, 111.1, 108.6, 61.3, 49.4, 14.2.

Diethyl 1-benzyl-3,4-bis(3-nitrophenyl)-1*H*-pyrrole-2,5-dicarboxylate (**6i**). Yellow solid, 93% yield, m.p. 118–120 °C; ^1^H NMR (400 MHz, CDCl_3_) *δ* 8.10–8.03 (m, 2H), 8.02 (s, 2H), 7.35 (dd, *J* = 12.3, 5.8 Hz, 6H), 7.28 (d, *J* = 7.5 Hz, 1H), 7.13 (d, *J* = 7.5 Hz, 2H), 6.18 (s, 2H), 4.02 (q, *J* = 7.1 Hz, 4H), 0.84 (t, *J* = 7.1 Hz, 6H); ^13^C NMR (100 MHz, CDCl_3_) *δ* 160.6, 147.6, 138.1, 136.6, 135.9, 128.8, 128.7, 128.6, 127.4, 126.4, 125.7, 125.0, 122.1, 61.1, 50.0, 13.5.

Diethyl 3,4-di([1,1′-biphenyl]-3-yl)-1-benzyl-1*H*-pyrrole-2,5-dicarboxylate (**6j**). White solid, 70% yield, m.p. 146–148 °C; ^1^H NMR (400 MHz, CDCl_3_) *δ* 7.44 (d, *J* = 6.3 Hz, 8H), 7.39 (t, *J* = 7.6 Hz, 5H), 7.37–7.25 (m, 6H), 7.20 (d, *J* = 7.6 Hz, 2H), 7.14 (d, *J* = 7.6 Hz, 2H), 6.18 (s, 2H), 4.05 (q, *J* = 7.1 Hz, 4H), 0.87 (t, *J* = 7.1 Hz, 6H); ^13^C NMR (100 MHz, CDCl_3_) *δ* 161.6, 141.2, 140.2, 138.8, 135.1, 131.4, 129.8, 129.5, 128.7, 128.6, 127.8, 127.2, 127.1, 126.5, 125.4, 124.8, 60.7, 49.6, 13.5.

Diethyl 3,4-bis(3-acetylphenyl)-1-benzyl-1*H*-pyrrole-2,5-dicarboxylate (**6k**). Yellow liquid, 90% yield; ^1^H NMR (400 MHz, CDCl_3_) *δ* 7.79 (s, 2H), 7.71 (s, 2H), 7.35 (t, *J* = 7.7 Hz, 2H), 7.30 (d, *J* = 4.8 Hz, 5H), 7.16 (d, *J* = 7.5 Hz, 2H), 6.17 (s, 2H), 4.01 (q, *J* = 7.3 Hz, 4H), 2.47 (s, 6H), 0.83 (t, *J* = 7.5 Hz, 6H); ^13^C NMR (100 MHz, CDCl_3_) *δ* 198.0, 161.1, 138.5, 136.3, 135.3, 134.9, 130.7, 130.3, 128.6, 127.7, 127.2, 126.7, 126.4, 124.8, 60.8, 49.7, 26.6, 13.5.

Diethyl 1-benzyl-3,4-bis(3-(dimethylamino)phenyl)-1*H*-pyrrole-2,5-dicarboxylate (**6l**). Brown solid, 79% yield, m.p. 145–147 °C; ^1^H NMR (400 MHz, CDCl_3_) *δ* 7.31 (t, *J* = 7.5 Hz, 2H), 7.23 (d, *J* = 7.5 Hz, 1H), 7.16 (d, *J* = 7.6 Hz, 2H), 7.05 (t, *J* = 7.8 Hz, 2H), 6.59 (d, *J* = 8.4 Hz, 2H), 6.54 (d, *J* = 7.6 Hz, 2H), 6.48 (s, 2H), 6.03 (s, 2H), 4.01 (q, *J* = 7.1 Hz, 4H), 2.76 (s, 12H), 0.88 (t, *J* = 7.1 Hz, 6H); ^13^C NMR (100 MHz, CDCl_3_) *δ* 162.0, 149.9, 138.3, 135.2, 131.6, 128.5, 127.8, 127.0, 126.6, 124.6, 120.0, 115.9, 111.6, 60.6, 49.4, 41.0, 13.5.

Diethyl 1-benzyl-3,4-di(naphthalen-2-yl)-1*H*-pyrrole-2,5-dicarboxylate (**6m**). White solid, 95% yield, m.p. 116–118 °C; ^1^H NMR (400 MHz, CDCl_3_) *δ* 7.77 (d, *J* = 7.0 Hz, 2H), 7.73–7.61 (m, 6H), 7.41 (t, *J* = 8.2 Hz, 6H), 7.36–7.25 (m, 5H), 6.21 (s, 2H), 4.01 (q, *J* = 7.1 Hz, 4H), 0.74 (t, *J* = 7.2 Hz, 6H); ^13^C NMR (100 MHz, CDCl_3_) *δ* 161.7, 138.8, 132.9, 132.2, 132.1, 131.2, 129.4, 128.9, 128.6, 127.9, 127.6, 127.2, 126.7, 126.6, 125.7, 125.6, 125.1, 60.7, 49.7, 13.4.

Diethyl 1-benzyl-3,4-bis(5-fluoro-2-methylphenyl)-1*H*-pyrrole-2,5-dicarboxylate (**6n**). Yellow liquid, 73% yield; ^1^H NMR (400 MHz, CDCl_3_) *δ* 7.33 (d, *J* = 11.7 Hz, 3H), 7.08 (s, 4H), 6.98 (s, 2H), 6.71–6.51 (m, 2H), 6.26 (s, 2H), 4.02 (s, 4H), 2.17 (s, 6H), 0.92–0.80 (m, 6H); ^13^C NMR (100 MHz, CDCl_3_) *δ* 160.8 (d, *J* = 27.5 Hz), 138.9, 131.3, 130.3, 128.6, 128.5, 127.0, 126.0, 118.5, 116.6 (d, *J* = 21.6 Hz), 113.8 (dd, *J* = 20.5, 6.7 Hz), 107.2 (d, *J* = 20.9 Hz), 102.7 (d, *J* = 24.5 Hz), 60.5, 49.5, 19.5, 13.2.

Diethyl 1-benzyl-3,4-bis(2,5-difluorophenyl)-1*H*-pyrrole-2,5-dicarboxylate (**6o**). White solid, 56% yield, m.p. 110–112 °C; ^1^H NMR (400 MHz, CDCl_3_) *δ* 7.36 (t, *J* = 7.5 Hz, 2H), 7.29 (d, *J* = 8.0 Hz, 1H), 7.15 (d, *J* = 7.6 Hz, 2H), 7.01–6.91 (m, 4H), 6.85–6.75 (m, 2H), 6.26 (s, 2H), 4.08 (q, *J* = 7.1 Hz, 4H), 0.94 (t, *J* = 7.2 Hz, 6H); ^13^C NMR (100 MHz, CDCl_3_) *δ* 160.5, 158.4 (dd, *J* = 150.5, 3.6 Hz), 156.0 (dd, *J* = 150.4, 3.7 Hz), 138.4, 128.6, 127.1, 126.3, 125.5, 123.9, 118.3 (dd, *J* = 21.3, 6.0 Hz), 115.9 (d, *J* = 9.8 Hz), 115.6 (t, *J* = 8.9 Hz), 115.3 (d, *J* = 10.6 Hz), 60.8, 49.9, 13.4.

Diethyl 1-benzyl-3,4-bis(3-fluoro-5-methoxyphenyl)-1*H*-pyrrole-2,5-dicarboxylate (**6p**). White solid, 96% yield, m.p. 84–86 °C; ^1^H NMR (400 MHz, CDCl_3_) *δ* 7.36 (t, *J* = 7.5 Hz, 2H), 7.29 (d, *J* = 7.2 Hz, 1H), 7.16 (d, *J* = 7.5 Hz, 2H), 6.56–6.46 (m, 4H), 6.45 (s, 2H), 6.11 (s, 2H), 4.08 (q, *J* = 7.3 Hz, 4H), 3.70 (s, 6H), 0.96 (t, *J* = 7.1 Hz, 6H); ^13^C NMR (100 MHz, CDCl_3_) *δ* 162.8 (d, *J* = 243.8 Hz), 161.2, 159.9 (d, *J* = 11.9 Hz), 138.4, 136.8 (d, *J* = 10.9 Hz), 129.8, 128.6, 127.2, 126.5, 124.6, 111.97 (d, *J* = 2 Hz), 110.0 (d, *J* = 22.2 Hz), 100.4 (d, *J* = 24.9 Hz), 60.83, 55.5, 50.0, 13.5.

Diethyl 1-benzyl-3,4-bis(3,5-difluorophenyl)-1*H*-pyrrole-2,5-dicarboxylate (**6q**). White solid, 59% yield, m.p. 96–98 °C; ^1^H NMR (400 MHz, CDCl_3_) *δ* 7.35 (t, *J* = 7.5 Hz, 2H), 7.29 (d, *J* = 6.8 Hz, 1H), 7.14 (d, *J* = 7.4 Hz, 2H), 6.71 (t, *J* = 9.2 Hz, 2H), 6.65 (d, *J* = 7.2 Hz, 4H), 6.14 (s, 2H), 4.07 (q, *J* = 7.1 Hz, 4H), 0.95 (t, *J* = 7.2 Hz, 6H); ^13^C NMR (100 MHz, CDCl_3_) *δ* 163.45 (d, *J* = 13.1 Hz), 161.1, 160.8, 138.2, 137.4 (t, *J* = 10.4 Hz), 128.6, 127.4, 126.5, 124.7, 113.61 (dd, *J* = 18.2, 7.1 Hz), 102.6 (t, *J* = 25.2 Hz), 61.0, 49.7, 13.4.

Diethyl 1-benzyl-3,4-bis(2,3-difluorophenyl)-1*H*-pyrrole-2,5-dicarboxylate (**6r**). Yellow solid, 91% yield, m.p. 92–95 °C; ^1^H NMR (400 MHz, CDCl_3_) *δ* 7.36 (t, *J* = 7.6 Hz, 2H), 7.29 (d, *J* = 8.7 Hz, 2H), 7.21–7.13 (m, 4H), 7.08 (t, *J* = 8.9 Hz, 3H), 6.28 (s, 2H), 4.08 (q, *J* = 7.1 Hz, 4H), 0.93 (t, *J* = 7.2 Hz, 6H); ^13^C NMR (100 MHz, CDCl_3_) *δ* 160.5, 138.4, 128.6, 128.5, 127.1, 126.6, 126.3, 125.6, 124.9 (d, *J* = 12.8 Hz), 123.8, 123.1, 117.4 (d, *J* = 17.3 Hz), 116.2 (d, *J* = 16.9 Hz), 60.8, 49.9, 13.3.

Diethyl 1-benzyl-3,4-bis(3,5-dimethoxyphenyl)-1*H*-pyrrole-2,5-dicarboxylate (**6s**). Yellow solid, 86% yield, m.p. 112–114 °C; ^1^H NMR (400 MHz, CDCl_3_) *δ* 7.38–7.30 (m, 2H), 7.32–7.23 (m, 1H), 7.17 (d, *J* = 7.5 Hz, 2H), 6.32 (dd, *J* = 10.4, 2.2 Hz, 6H), 6.07 (s, 2H), 4.06 (q, *J* = 7.2 Hz, 4H), 3.68 (s, 6H), 0.94 (t, *J* = 7.1 Hz, 6H); ^13^C NMR (100 MHz, CDCl_3_) *δ* 161.7 159.8, 138.6, 136.4, 130.7, 128.5, 127.1, 126.6, 124.5, 108.7, 99.3, 60.7, 55.3, 49.5, 13.5.

General procedure for compounds **7a**–**7s**. To a stirred solution of **6** (0.5 mmol, 1.0 equiv.) in CF_3_COOH (1.5 mL), anisole (0.5 mL), and 98% sulfuric acid (0.2 mL) were added, the reaction mixture was stirred at 90 °C for 1 h. After completion of the reaction, saturated solution was added dropwise to the reaction mixture to adjust the pH to 7–8, and the aqueous phase was extracted with EtOAc, dried over anhydrous sodium sulfate, and the solvent was evaporated. The crude product was purified by column chromatography (*V*_PE_:*V*_EA_ = 20:1) to obtain compounds **7a**–**7s**.

Diethyl 3,4-diphenyl-1*H*-pyrrole-2,5-dicarboxylate (**7a**). White solid, 94% yield, m.p. 141–142 °C (lit. [38] 142−144 °C); ^1^H NMR (400 MHz, CDCl_3_) *δ* 9.83 (s, 1H), 7.25–7.00 (m, 10H), 4.23 (q, *J* = 7.1 Hz, 4H), 1.17 (t, *J* = 7.1 Hz, 6H); ^13^C NMR (100 MHz, CDCl_3_) *δ* 160.4, 133.0, 131.4, 130.8, 127.2, 126.9, 121.5, 60.9, 14.0.

Diethyl 3,4-di-*m*-tolyl-1*H*-pyrrole-2,5-dicarboxylate (**7b**). White solid, 82% yield, m.p. 121–123 °C; ^1^H NMR (400 MHz, CDCl_3_) *δ* 10.15 (s, 1H), 7.42 (d, *J* = 4.4 Hz, 1H), 7.35 (q, *J* = 6.7, 5.9 Hz, 3H), 7.29 (s, 1H), 7.19 (s, 1H), 7.17 (s, 1H), 7.15 (d, *J* = 2.7 Hz, 1H), 4.51 (q, *J* = 7.1 Hz, 4H), 2.53 (s, 6H), 1.46 (t, *J* = 7.2 Hz, 6H); ^13^C NMR (100 MHz, CDCl_3_) *δ* 160.6, 136.5, 133.0, 131.7, 127.9, 127.6, 127.1, 125.8, 121.6, 60.9, 21.4, 14.1.

Diethyl 3,4-bis(3-methoxyphenyl)-1*H*-pyrrole-2,5-dicarboxylate (**7c**). Yellow solid, 75% yield, m.p. 91–93 °C; ^1^H NMR (400 MHz, CDCl_3_) *δ* 9.86 (s, 1H), 7.12 (t, *J* = 7.9 Hz, 2H), 6.79–6.71 (m, 4H), 6.66 (s, 2H), 4.24 (q, *J* = 7.1 Hz, 4H), 3.64 (s, 6H), 1.19 (t, *J* = 7.2 Hz, 6H); ^13^C NMR (100 MHz, CDCl_3_) *δ* 160.4, 158.7, 134.4, 131.2, 128.3, 123.5, 121.6, 116.3, 113.0, 60.9, 55.1, 14.1.

Diethyl 3,4-bis(3-fluorophenyl)-1*H*-pyrrole-2,5-dicarboxylate (**7d**). White solid, 96% yield, m.p. 148–151 °C; ^1^H NMR (400 MHz, CDCl_3_) *δ* 9.9 (s, 1H), 7.2 (q, *J* = 7.5 Hz, 2H), 6.9 (dd, *J* = 22.4, 7.9 Hz, 6H), 4.2 (q, *J* = 7.0 Hz, 4H), 1.2 (t, *J* = 7.1 Hz, 6H); ^13^C NMR (100 MHz, CDCl_3_) *δ* 163.3, 160.2, 135.0 (d, *J* = 8.4 Hz), 129.9, 128.8 (d, *J* = 8.4 Hz), 126.6 (d, *J* = 2.5 Hz), 121.8, 117.8 (d, *J* = 22.0 Hz), 114.1 (d, *J* = 21.0 Hz), 61.2, 14.0.

Diethyl 3,4-bis(2-fluorophenyl)-1*H*-pyrrole-2,5-dicarboxylate (**7e**). White solid, 84% yield, m.p. 133–135 °C; ^1^H NMR (400 MHz, CDCl_3_) *δ* 10.0 (s, 1H), 7.24–7.18 (m, 2H), 7.03–6.94 (m, 6H), 4.2 (q, *J* = 6.9 Hz, 4H), 1.1 (t, *J* = 7.1 Hz, 6H); ^13^C NMR (100 MHz, CDCl_3_) *δ* 160.2, 159.2, 132.2, 129.30 (d, *J* = 8.3 Hz), 124.74, 123.2, 122.9, 121.2 (d, *J* = 16.1 Hz), 114.9 (d, *J* = 22.5 Hz), 61.0, 13.9.

Diethyl 3,4-bis(3-chlorophenyl)-1*H*-pyrrole-2,5-dicarboxylate (**7f**). White solid, 91% yield, m.p. 152–154 °C; ^1^H NMR (400 MHz, CDCl_3_) *δ* 9.95 (s, 1H), 7.24–7.17 (m, 4H), 7.12 (t, *J* = 8.1 Hz, 2H), 6.92 (dt, *J* = 7.5, 1.4 Hz, 2H), 4.24 (q, *J* = 7.1 Hz, 4H), 1.19 (t, *J* = 7.2 Hz, 6H); ^13^C NMR (100 MHz, CDCl_3_) *δ* 160.2, 134.6, 133.2, 131.1, 129.5, 129.0, 128.7, 127.3, 121.9, 61.2, 14.0.

Diethyl 3,4-bis(3-(trifluoromethyl)phenyl)-1*H*-pyrrole-2,5-dicarboxylate (**7g**). White solid, 67% yield, m.p. 123–125 °C; ^1^H NMR (400 MHz, CDCl_3_) *δ* 10.10 (s, 1H), 7.47 (d, *J* = 7.9 Hz, 2H), 7.42 (s, 2H), 7.31 (t, *J* = 7.8 Hz, 2H), 7.23 (d, *J* = 7.8 Hz, 2H), 4.23 (q, *J* = 7.1 Hz, 4H), 1.15 (t, *J* = 7.1 Hz, 6H); ^13^C NMR (100 MHz, CDCl_3_) *δ* 160.2, 134.1, 133.5, 130.1, 129.8, 129.5, 128.0, 124.0 (d, *J* = 3.6 Hz), 122.1, 61.3, 13.8.

Diethyl 3,4-bis(3-(trifluoromethoxy)phenyl)-1*H*-pyrrole-2,5-dicarboxylate (**7h**). White solid, 72% yield, m.p. 164–166 °C; ^1^H NMR (400 MHz, CDCl_3_) *δ* 10.10 (s, 1H), 7.29 (d, *J* = 8.3 Hz, 2H), 7.11 (t, *J* = 7.5 Hz, 4H), 7.01 (s, 2H), 4.29 (q, *J* = 7.1 Hz, 4H), 1.21 (t, *J* = 7.2 Hz, 6H); ^13^C NMR (100 MHz, CDCl_3_) *δ* 160.2, 148.5, 134.7 (d, *J* = 20 Hz), 129.5, 129.3, 128.8, 123.5, 121.9, 119.8, 114.5, 61.3, 13.9.

Diethyl 3,4-bis(3-nitrophenyl)-1*H*-pyrrole-2,5-dicarboxylate (**7i**). Yellow solid, 76% yield, m.p. 174–176 °C; ^1^H NMR (400 MHz, CDCl_3_) *δ* 10.18 (s, 1H), 8.10 (s, 4H), 7.39 (s, 4H), 4.26 (q, *J* = 7.2 Hz, 4H), 1.17 (t, *J* = 7.3 Hz, 6H); ^13^C NMR (100 MHz, CDCl_3_) *δ* 159.8, 147.7, 136.9, 134.2, 128.7, 128.4, 126.1, 122.5, 122.3, 61.6, 14.0.

Diethyl 3,4-di([1,1′-biphenyl]-3-yl)-1*H*-pyrrole-2,5-dicarboxylate (**7j**). White solid, 48% yield, m.p. 149–152 °C; ^1^H NMR (400 MHz, CDCl_3_) *δ* 7.36–7.27 (m, 5H), 7.20 (s, 1H), 7.09 (d, *J* = 7.5 Hz, 5H), 7.01–6.90 (m, 2H), 6.84 (d, *J* = 9.3 Hz, 1H), 6.71–6.49 (m, 2H), 6.26 (s, 2H), 4.06–3.98 (m, 4H), 0.91–0.84 (m, 6H); ^13^C NMR (100 MHz, CDCl_3_) *δ* 160.5, 141.2, 140.3, 133.6, 131.4, 130.2, 129.9, 128.7, 127.9, 127.1, 125.8, 121.7, 61.0, 14.1.

Diethyl 3,4-bis(3-acetylphenyl)-1*H*-pyrrole-2,5-dicarboxylate (**7k**). Yellow solid, 37% yield, m.p. 162–165 °C; ^1^H NMR (400 MHz, CDCl_3_) *δ* 10.03 (s, 1H), 7.80 (d, *J* = 7.2 Hz, 2H), 7.75 (s, 2H), 7.30 (d, *J* = 8.0 Hz, 4H), 4.23 (q, *J* = 7.1 Hz, 4H), 2.45 (s, 6H), 1.15 (t, *J* = 7.2 Hz, 6H); ^13^C NMR (100 MHz, CDCl_3_) *δ* 197.9, 160.2, 136.4, 135.6, 133.3, 131.2, 130.1, 127.8, 127.1, 121.9, 61.2, 26.6, 14.0.

Diethyl 3,4-bis(3-(dimethylamino)phenyl)-1*H*-pyrrole-2,5-dicarboxylate (**7l**). Yellow liquid, 46% yield; ^1^H NMR (400 MHz, CDCl_3_) *δ* 9.96 (s, 1H), 7.72 (d, *J* = 8.4 Hz, 3H), 7.08 (d, *J* = 7.4 Hz, 2H), 6.79 (d, *J* = 8.3 Hz, 3H), 4.26 (dd, *J* = 19.1, 7.2 Hz, 4H), 3.54 (s, 12H), 1.25 (t, *J* = 9.4 Hz, 6H); ^13^C NMR (100 MHz, CDCl_3_) *δ* 160.6, 147.8, 138.5, 133.2, 130.9, 127.5, 122.0, 118.1, 113.4, 57.1, 55.4, 14.3.

Diethyl 3,4-di(naphthalen-2-yl)-1*H*-pyrrole-2,5-dicarboxylate (**7m**). Brown liquid, 72% yield; ^1^H NMR (400 MHz, CDCl_3_) *δ* 10.03 (s, 1H), 7.79–7.70 (m, 4H), 7.68 (d, *J* = 7.7 Hz, 2H), 7.64 (s, 1H), 7.62 (s, 1H), 7.44–7.37 (m, 5H), 7.28 (s, 1H), 4.25 (q, *J* = 7.1 Hz, 4H), 1.13 (t, *J* = 7.1 Hz, 6H); ^13^C NMR (100 MHz, CDCl_3_) *δ* 160.6, 132.9, 132.4, 131.3, 130.6, 130.1, 129.0, 128.0, 127.6, 126.8, 125.8, 125.7, 122.0, 61.0, 14.1.

Diethyl 3,4-bis(5-fluoro-2-methylphenyl)-1*H*-pyrrole-2,5-dicarboxylate (**7n**). Purple liquid, 75% yield; ^1^H NMR (400 MHz, CDCl_3_) *δ* 9.62 (s, 1H), 7.01 (d, *J* = 8.3 Hz, 2H), 6.89 (d, *J* = 9.6 Hz, 2H), 6.81 (d, *J* = 6.5 Hz, 2H), 4.18 (q, *J* = 8.2, 7.7 Hz, 4H), 2.11 (d, *J* = 6.6 Hz, 6H), 1.14–1.06 (m, 6H); ^13^C NMR (100 MHz, CDCl_3_) *δ* 160.4 (d, *J* = 14.4 Hz), 160.3, 130.3, 129.6, 117.0, 115.4, 114.5, 114.3 (d, *J* = 4.7 Hz), 114.1 (d, *J* = 4.2 Hz), 61.1, 46.4, 8.5.

Diethyl 3,4-bis(2,5-difluorophenyl)-1*H*-pyrrole-2,5-dicarboxylate (**7o**). White solid, 78% yield, m.p. 149–151 °C; ^1^H NMR (400 MHz, CDCl_3_) *δ* 10.10 (s, 1H), 7.00–6.89 (m, 4H), 6.78 (s, 2H), 4.24 (q, *J* = 7.2 Hz, 4H), 1.16 (t, *J* = 7.2 Hz, 6H); ^13^C NMR (100 MHz, CDCl_3_) *δ* 159.8, 158.4 (d, *J* = 143.7 Hz), 156.0 (d, *J* = 144.3 Hz), 123.5, 123.0, 122.3 (dd, *J* = 19.0, 8.9 Hz), 118.5 (d, *J* = 23.6 Hz), 116.1 (dd, *J* = 8.4, 5.8 Hz), 115.8 (dd, *J* = 8.7, 3.8 Hz), 61.3, 13.9.

Diethyl 3,4-bis(3-fluoro-5-methoxyphenyl)-1*H*-pyrrole-2,5-dicarboxylate (**7p**). Brown solid, 44% yield, m.p. 101–103 °C; ^1^H NMR (400 MHz, CDCl_3_) *δ* 9.95 (s, 1H), 6.54–6.46 (m,4H), 6.45 (s, 2H), 4.25 (q, *J* = 7.2 Hz, 4H), 3.66 (s, 6H), 1.20 (t, *J* = 7.2 Hz, 6H); ^13^C NMR (100 MHz, CDCl_3_) *δ* 162.7 (d, *J* = 243.8 Hz), 161.5, 159.9 (d, *J* = 11.8 Hz), 135.3 (d, *J* = 10.8 Hz), 129.9, 121.7, 112.4, 110.2 (d, *J* = 22.5 Hz), 100.7 (d, *J* = 25.0 Hz), 61.2, 55.5, 14.0.

Diethyl 3,4-bis(3,5-difluorophenyl)-1*H*-pyrrole-2,5-dicarboxylate (**7q**). White solid, 78% yield, m.p. 158–160 °C; ^1^H NMR (400 MHz, CDCl_3_) *δ* 10.03 (s, 1H), 6.69 (dd, *J* = 23.1, 8.4 Hz, 6H), 4.26 (q, *J* = 7.1 Hz, 4H), 1.20 (t, *J* = 7.1 Hz, 6H); ^13^C NMR (100 MHz, CDCl_3_) *δ* 162.2 (dd, *J* = 247.8, 13.1 Hz), 159.9, 129.4 (d, *J* = 178.5 Hz), 122.0, 114.5, 113.8 (dd, *J* = 18.2, 7.1 Hz), 103.0 (t, *J* = 25.3 Hz), 61.4, 13.9.

Diethyl 3,4-bis(2,3-difluorophenyl)-1*H*-pyrrole-2,5-dicarboxylate (**7r**). Yellow solid, 43% yield, m.p. 127–129 °C; ^1^H NMR (400 MHz, CDCl_3_) *δ* 10.14 (s, 1H), 7.06 (q, *J* = 8.6 Hz, 2H), 6.90 (q, *J* = 7.4 Hz, 2H), 6.77 (s, 2H), 4.26 (q, *J* = 7.1 Hz, 4H), 1.16 (t, *J* = 7.1 Hz, 6H); ^13^C NMR (100 MHz, CDCl_3_) *δ* 159.9, 151.7 (d, *J* = 12.1 Hz), 149.2 (d, *J* = 11.1 Hz), 126.8, 123.4, 123.2, 123.1, 116.6 (d, *J* = 17.1 Hz), 61.3, 13.8.

Dethyl 3,4-bis(3,5-dimethoxyphenyl)-1*H*-pyrrole-2,5-dicarboxylate (**7s**). Yellow solid, 43% yield, m.p. 127–129 °C; Yellow liquid, 92% yield; ^1^H NMR (400 MHz, CDCl_3_) *δ* 10.82 (s, 1H), 7.29 (s, 1H), 5.21 (q, *J* = 7.2 Hz, 4H), 4.60 (s, 12H), 2.16 (t, *J* = 7.2 Hz, 6H); ^13^C NMR (100 MHz, CDCl_3_) *δ* 160.4, 159.8, 134.9, 131.1, 121.5, 109.1, 99.6, 61.0, 55.3, 14.1.

The synthesis procedures of compounds **8a**–**8s** were the same as compound **5**.

3,4-Diphenyl-1*H*-pyrrole-2,5-dicarboxylic acid (**8a**). Yellow solid, 52% yield, m.p. > 250 °C (lit. [40] 249−250 °C); ^1^H NMR (400 MHz, DMSO-*d*_6_) *δ* 12.7 (s, 2H), 12.0 (s, 1H), 7.5 (d, *J* = 7.5 Hz, 1H), 7.4–7.2 (m, 3H), 7.1 (q, *J* = 7.3, 6.5 Hz, 3H), 7.0 (d, *J* = 8.1 Hz, 2H), 6.8 (d, *J* = 12.0 Hz, 1H); ^13^C NMR (100 MHz, DMSO-*d*_6_) *δ* 161.7, 141.8, 131.2, 129.7, 128.2, 127.6, 116.7; HRMS (ESI): *m*/*z* [M + Na]^+^ calcd for C_18_H_13_NO_4_: 330.0737; found: 330.0731.

3,4-Di-*m*-tolyl-1*H*-pyrrole-2,5-dicarboxylic acid (**8b**). Yellow solid, 35% yield, m.p. > 250 °C; ^1^H NMR (400 MHz, DMSO-*d*_6_) *δ* 11.69 (s, 1H), 7.03 (t, *J* = 7.6 Hz, 2H), 6.94 (d, *J* = 7.7 Hz, 2H), 6.89 (s, 2H), 6.82 (d, *J* = 7.5 Hz, 2H), 2.16 (s, 6H); ^13^C NMR (100 MHz, DMSO-*d*_6_) *δ* 162.1, 136.4, 134.2, 131.7, 130.7, 128.1, 127.6, 127.5, 122.7, 21.4; HRMS (ESI): *m*/*z* [M + NH_4_]^+^ calcd for C_20_H_17_NO_4_: 353.1496; found: 353.1491.

3,4-Bis(3-methoxyphenyl)-1*H*-pyrrole-2,5-dicarboxylic acid (**8c**). White solid, 91% yield, m.p. 225–227 °C; ^1^H NMR (400 MHz, DMSO-*d*_6_) *δ* 12.59 (s, 2H), 11.81 (s, 1H), 7.07 (t, *J* = 7.9 Hz, 2H), 6.72 (d, *J* = 8.4 Hz, 2H), 6.66 (s, 1H), 6.64 (s, 1H), 6.62 (s, 2H), 3.59 (s, 6H); ^13^C NMR (100 MHz, DMSO-*d*_6_) *δ* 161.9, 158.5, 135.7, 130.2, 128.6, 123.6, 122.7, 116.9, 112.5, 55.2; HRMS (ESI): *m*/*z* [M + NH_4_]^+^ calcd for C_20_H_17_NO_6_: 385.1394; found: 385.1397.

3,4-Bis(3-fluorophenyl)-1*H*-pyrrole-2,5-dicarboxylic acid (**8d**). White solid, 98% yield, m.p. > 250 °C; ^1^H NMR (400 MHz, DMSO-*d*_6_) *δ* 12.7 (s, 2H), 12.1 (s, 1H), 7.2 (q, *J* = 7.5 Hz, 2H), 7.0–6.8 (m, 6H); ^13^C NMR (100 MHz, DMSO-*d*_6_) *δ* 162.3 (d, *J* = 126.6 Hz), 160.5, 136.7 (d, *J* = 8.5 Hz), 129.4 (d, *J* = 8.5 Hz), 129.0, 127.4, 123.0, 118.0 (d, *J* = 21.5 Hz), 113.9 (d, *J* = 20.6 Hz); ^19^F NMR (376 MHz, DMSO-*d*_6_) *δ* –114.9; HRMS (ESI): *m*/*z* [M + NH_4_]^+^ calcd for C_18_H_11_F_2_NO_4_: 361.0994; found: 361.0992.

3,4-Bis(2-fluorophenyl)-1*H*-pyrrole-2,5-dicarboxylic acid (**8e**). White solid, 98% yield, m.p. > 250 °C; ^1^H NMR (400 MHz, DMSO-*d*_6_) *δ* 12.8 (s, 2H), 12.3 (s, 1H), 7.3–7.2 (m, 2H), 7.1–6.9 (m, 6H). ^13^C NMR (100 MHz, DMSO-*d*_6_) *δ* 116.5, 159.1, 132.7, 129.6 (d, *J* = 8.0 Hz), 124.1, 124.0, 123.8, 122.2 (d, *J* = 16.3 Hz), 115.2 (d, *J* = 22.4 Hz); ^19^F NMR (376 MHz, DMSO-*d*_6_) *δ* –113.8; HRMS (ESI): *m*/*z* [M + Na]^+^ calcd for C_18_H_11_F_2_NO_4_: 366.0548; found: 366.0544.

3,4-Bis(3-chlorophenyl)-1*H*-pyrrole-2,5-dicarboxylic acid (**8f**). White solid, 83% yield, m.p. > 250 °C; ^1^H NMR (400 MHz, DMSO-*d*_6_) *δ* 12.82 (s, 2H), 12.15 (s, 1H), 7.27–7.13 (m, 6H), 7.00 (d, *J* = 7.1 Hz, 2H); ^13^C NMR (100 MHz, DMSO-*d*_6_) *δ* 161.6, 136.2, 132.3, 130.8, 129.8, 129.5, 128.9, 127.1, 123.1; HRMS (ESI): *m*/*z* [M + NH_4_]^+^ calcd for C_18_H_11_ClNO_4_: 393.0403; found: 393.0410.

3,4-Bis(3-(trifluoromethyl)phenyl)-1*H*-pyrrole-2,5-dicarboxylic acid (**8g**). Brown solid, 94% yield, m.p. 242–245 °C; ^1^H NMR (400 MHz, DMSO-*d*_6_) *δ* 12.74 (s, 2H), 12.28 (s, 1H), 7.51 (d, *J* = 6.8 Hz, 2H), 7.41 (d, *J* = 6.6 Hz, 4H), 7.32 (s, 2H); ^13^C NMR (100 MHz, DMSO-*d*_6_) *δ* 161.6, 135.3, 135.0, 128.9, 128.7, 127.7 (d, *J* = 3.5 Hz), 125.9, 123.7, 123.2; ^19^F NMR (376 MHz, DMSO-*d*_6_) *δ* –61.4; HRMS (ESI): *m*/*z* [M + NH_4_]^+^ calcd for C_20_H_11_F_6_NO_4_: 461.0931; found: 461.0939.

3,4-Bis(3-(trifluoromethoxy)phenyl)-1*H*-pyrrole-2,5-dicarboxylic acid (**8h**). Brown solid, 88% yield, m.p. 49–51 °C; ^1^H NMR (400 MHz, DMSO-*d*_6_) *δ* 12.54 (s, 2H), 12.22 (s, 1H), 7.31 (t, *J* = 7.7 Hz, 2H), 7.14 (t, *J* = 8.4 Hz, 4H), 6.95 (s, 2H); ^13^C NMR (100 MHz, DMSO-*d*_6_) *δ* 172.5, 161.6, 148.0, 136.2, 130.5 (d, *J* = 36 Hz), 129.5, 128.7, 123.7, 123.1, 119.7; ^19^F NMR (376 MHz, DMSO-*d*_6_) *δ* –57.1; HRMS (ESI): *m*/*z* [M + NH_4_]^+^ calcd for C_20_H_11_F_6_NO_6_: 493.0829; found: 493.0826.

3,4-Bis(3-nitrophenyl)-1*H*-pyrrole-2,5-dicarboxylic acid (**8i**). Brown solid, 96% yield, m.p. 232–234 °C; ^1^H NMR (400 MHz, DMSO-*d*_6_) *δ* 12.48 (s, 1H), 8.25 (d, *J* = 8.3 Hz, 1H), 8.20 (d, *J* = 7.5 Hz, 1H), 8.05 (d, *J* = 7.9 Hz, 2H), 7.99 (s, 1H), 7.64 (t, *J* = 7.8 Hz, 1H), 7.49 (dt, *J* = 15.6, 7.9 Hz, 4H); ^13^C NMR (100 MHz, DMSO-*d*_6_) *δ* 161.5, 147.4, 141.1, 138.1, 135.6, 129.4, 128.0, 126.0, 122.2; HRMS (ESI): *m*/*z* [M + NH_4_]^+^ calcd for C_18_H_11_N_3_O_8_: 415.0884; found: 415.0892.

3,4-Di([1,1′-biphenyl]-3-yl)-1*H*-pyrrole-2,5-dicarboxylic acid (**8j**). Brown solid, 94% yield, m.p. 243–245 °C; ^1^H NMR (400 MHz, DMSO-*d*_6_) *δ* 12.61 (s, 2H), 11.97 (s, 1H), 7.45 (d, *J* = 7.9 Hz, 2H), 7.40 (d, *J* = 8.6 Hz, 5H), 7.37–7.23 (m, 9H), 7.15 (d, *J* = 7.7 Hz, 2H); ^13^C NMR (100 MHz, DMSO-*d*_6_) *δ* 161.9, 140.8, 139.6, 134.9, 130.5, 130.4, 130.0, 129.3, 128.4, 127.7, 127.1, 125.4, 122.9; HRMS (ESI): *m*/*z* [M + NH_4_]^+^ calcd for C_30_H_21_NO_4_: 477.1809; found: 477.1816.

3,4-Bis(3-acetylphenyl)-1*H*-pyrrole-2,5-dicarboxylic acid (**8k**). Brown solid, 92% yield, m.p. 165–167 °C; ^1^H NMR (400 MHz, DMSO-*d*_6_) *δ* 12.55 (s, 2H), 12.16 (s, 1H), 7.74 (d, *J* = 7.0 Hz, 2H), 7.68 (s, 2H), 7.33 (d, *J* = 7.9 Hz, 4H), 2.41 (s, 6H); ^13^C NMR (100 MHz, DMSO-*d*_6_) *δ* 198.2, 161.7, 136.3, 136.0, 131.3, 129.6, 128.2, 126.8, 123.1, 27.1, 21.6; HRMS (ESI): *m*/*z* [M + NH_4_]^+^ calcd for C_22_H_17_NO_6_: 409.1394; found: 409.1388.

3,4-Bis(3-(dimethylamino)phenyl)-1*H*-pyrrole-2,5-dicarboxylic acid (**8l**). Brown solid, 87% yield, m.p. 184–186 °C; ^1^H NMR (400 MHz, DMSO-*d*_6_) *δ* 7.85 (s, 1H), 7.10 (d, *J* = 8.7 Hz, 2H), 6.94 (s, 3H), 6.83 (t, *J* = 8.5 Hz, 2H), 6.75 (d, *J* = 8.4 Hz, 1H), 1.23 (s, 12H); ^13^C NMR (100 MHz, DMSO-*d*_6_) *δ* 175.0, 163.3, 158.9, 131.0, 129.8, 127.5, 115.3, 114.1, 113.2, 29.5; HRMS (ESI): *m*/*z* [M + Na]^+^ calcd for C_22_H_23_N_3_O_4_: 416.1581; found: 416.1576.

3,4-Di(naphthalen-2-yl)-1*H*-pyrrole-2,5-dicarboxylic acid (**8m**). Brown solid, 51% yield, m.p. 171–173 °C; ^1^H NMR (400 MHz, DMSO-*d*_6_) *δ* 12.11 (s, 1H), 7.77 (d, *J* = 7.4 Hz, 2H), 7.69 (s, 3H), 7.65 (d, *J* = 8.2 Hz, 2H), 7.38 (d, *J* = 6.3 Hz, 5H), 7.26 (d, *J* = 8.1 Hz, 2H); ^13^C NMR (100 MHz, DMSO-*d*_6_) *δ* 162.0, 132.9, 132.1, 130.6, 129.8, 129.7, 129.6, 128.2, 127.8, 126.7, 126.3, 126.2, 123.3; HRMS (ESI): *m*/*z* [M + NH_4_]^+^ calcd for C_26_H_17_NO_4_: 425.1496; found: 425.1492.

3,4-Bis(5-fluoro-2-methylphenyl)-1*H*-pyrrole-2,5-dicarboxylic acid (**8n**). Brown solid, 46% yield, m.p. 191–193 °C; ^1^H NMR (400 MHz, DMSO-*d*_6_) *δ* 12.17 (s, 1H), 7.22 (t, *J* = 7.3 Hz, 1H), 7.04 (t, *J* = 7.7 Hz, 2H), 6.95 (d, *J* = 7.1 Hz, 2H), 6.91–6.82 (m, 1H), 2.06 (s, 6H); ^13^C NMR (100 MHz, DMSO-*d*_6_) *δ* 161.6 (d, *J* = 9.2 Hz), 161.5, 130.9, 129.9, 126.3, 124.1, 117.0, 115.7, 114.0 (d, *J* = 20.1 Hz), 19.6; ^19^F NMR (376 MHz, DMSO-*d*_6_) *δ* –119.1; HRMS (ESI): *m*/*z* [M + NH_4_]^+^ calcd for C_20_H_15_F_2_NO_4_: 389.1307; found: 389.1301.

3,4-Bis(2,5-difluorophenyl)-1*H*-pyrrole-2,5-dicarboxylic acid (**8o**). Yellow solid, 96% yield, m.p. > 250 °C; ^1^H NMR (400 MHz, DMSO-*d*_6_) *δ* 12.56 (s, 1H), 7.15–7.04 (m, 4H), 6.96 (s, 2H); ^13^C NMR (100 MHz, DMSO-*d*_6_) *δ* 161.3, 158.2 (d, *J* = 121.5 Hz), 155.9 (d, *J* = 123.0 Hz), 124.3, 123.7 (dd, *J* = 19.4, 9.2 Hz), 122.8, 119.0 (d, *J* = 24.2 Hz), 116.6 (dd, *J* = 25.6, 9.0 Hz), 116.1 (dd, *J* = 23.7, 8.9 Hz); ^19^F NMR (376 MHz, DMSO-*d*_6_) *δ* –119.5, –120.4; HRMS (ESI): *m*/*z* [M + NH_4_]^+^ calcd for C_18_H_9_F_4_NO_4_: 397.0806; found: 397.0813.

3,4-Bis(3-fluoro-5-methoxyphenyl)-1*H*-pyrrole-2,5-dicarboxylic acid (**8p**). Yellow solid, 75% yield, m.p. > 250 °C; ^1^H NMR (400 MHz, DMSO-*d*_6_) *δ* 12.08 (s, 1H), 6.64 (d, *J* = 11.0 Hz, 2H), 6.52 (d, *J* = 9.7 Hz, 2H), 6.49 (s, 2H), 3.63 (s, 6H); ^13^C NMR (100 MHz, DMSO-*d*_6_) *δ* 162.4 (d, *J* = 241.0 Hz), 161.6, 159.9 (d, *J* = 12.0 Hz), 137.0 (d, *J* = 11.1 Hz), 129.0, 123.0, 110.2 (d, *J* = 21.9 Hz), 100.3 (d, *J* = 25.0 Hz), 55.9; ^19^F NMR (376 MHz, DMSO-*d*_6_) *δ* –113.6; HRMS (ESI): *m*/*z* [M + NH_4_]^+^ calcd for C_20_H_15_F_2_NO_6_: 421.1206; found: 421.1202.

3,4-Bis(3,5-difluorophenyl)-1*H*-pyrrole-2,5-dicarboxylic acid (**8q**). Brown solid, 71% yield, m.p. 237–239 °C; ^1^H NMR (400 MHz, DMSO-*d*_6_) *δ* 12.90 (s, 2H), 12.35 (s, 1H), 7.06 (t, *J* = 9.2 Hz, 2H), 6.83 (d, *J* = 8.2 Hz, 4H); ^13^C NMR (100 MHz, DMSO-*d*_6_) *δ* 163.1 (d, *J* = 13.7 Hz), 161.4, 137.8 (t, *J* = 10.7 Hz), 127.9, 123.2, 114.4 (dd, *J* = 19.2, 7.1 Hz), 102.8 (t, *J* = 25.6 Hz); ^19^F NMR (376 MHz, DMSO-*d*_6_) *δ* –111.7; HRMS (ESI): *m*/*z* [M + NH_4_]^+^ calcd for C_18_H_9_F_4_NO_4_: 397.0806; found: 397.0809.

3,4-Bis(2,3-difluorophenyl)-1*H*-pyrrole-2,5-dicarboxylic acid (**8r**). Brown solid, 96% yield, m.p. > 250 °C; ^1^H NMR (400 MHz, DMSO-*d*_6_) *δ* 12.64 (s, 1H), 7.26 (q, *J* = 8.8 Hz, 2H), 7.01 (q, *J* = 7.4 Hz, 2H), 6.84 (d, *J* = 9.1 Hz, 2H); ^13^C NMR (100 MHz, DMSO-*d*_6_) *δ* 161.3, 150.2 (d, *J* = 179.7 Hz), 147.9 (d, *J* = 181.2 Hz), 127.8, 124.4, 124.3, 124.2, 122.4, 116.9 (d, *J* = 16.9 Hz); ^19^F NMR (376 MHz, DMSO-*d*_6_) *δ* –139.9, –140.0; HRMS (ESI): *m*/*z* [M + NH_4_]^+^ calcd for C_18_H_9_F_4_NO_4_: 397.0806; found: 397.0812.

3,4-Bis(3,5-dimethoxyphenyl)-1*H*-pyrrole-2,5-dicarboxylic acid (**8s**). Yellow solid, 96% yield, m.p. > 250 °C; ^1^H NMR (400 MHz, DMSO-*d*_6_) *δ* 11.78 (s, 1H), 6.31 (s, 2H), 6.27 (d, *J* = 1.9 Hz, 4H), 3.59 (s, 12H); ^13^C NMR (100 MHz, DMSO-*d*_6_) *δ* 161.9, 159.7, 136.3, 130.2, 122.7, 109.4, 98.9, 55.4; HRMS (ESI): *m*/*z* [M + NH_4_]^+^ calcd for C_22_H_21_NO_8_: 445.1605; found: 445.1609.

### 4.3. In Vitro Target Compounds against Seven Phytopathogenic Fungi

Seven common and representative plant pathogenic fungi that are harmful to crops (grains, fruits, and vegetables) and have a range of effects were selected to test the newly synthesized compounds, including *Fusarium oxysporium* f. sp. *Cucumeris* (*F*. *oxysporium* f. sp. *Cucumeris*), *Cercospora arachidicola hori* (*C*. *arachidicola hori*), *Physalospora piricola* (*P*. *piricola*), *Rhizoctonia cerealis* (*R*. *cerealis*), *Alternaria solani* (*A*. *solani*), *Pyricularia grisea* (*P*. *grisea*), and *Sclerotinia sclerotiorum* (*S*. *sclerotiorum*), with the agricultural fungicide carbendazim as the control. The in vitro antifungal activity test was performed using the mycelial growth rate method to evaluate the activity of the compounds. The detailed test procedures are provided in the Supplementary Materials.

### 4.4. Calculation Procedures for Molecular Docking Research

The three-dimensional (3D) structure of cytochrome P450 sterol 14*α*-demethylase enzyme of the *Rhizoctonia cerealis* (*Rc*CYP51) was constructed based on *Saccharomyces cerevisiae* CYP51 crystal structure (PDB code: 4LXJ) as the template using SWISS-MODEL. Detailed procedures are provided in the Supplementary Materials.

In summary, taking inspiration from the molecular structure of natural product lycolic acid (**5**), a series of novel phenylpyrrole analogues containing carboxylic acid functional groups were designed and synthesized. Their fungicidal activities against seven kinds of phytopathogenic fungi at 50 μg/mL were evaluated. Using palladium-catalyzed Suzuki coupling as the key reaction can efficiently achieve the structure optimization of lycogalic acid analogues with dicarboxylic acid functional groups. The target compounds exhibited broad-spectrum fungicidal activities. Most of the compounds showed more than 60% inhibitory activity against *R. cerealis* and *S. sclerotiorum*. Compounds **8g** and **8h** showed over 90% inhibitory rate against *R. cerealis*, similar to carbendazim; the kinds and numbers of the substituents on the benzene ring have different effects on the bactericidal activity. Molecular docking analysis revealed that compounds **8g** and **8h** possessed a stronger affinity to *Rc*CYP51. This work involves leader selection, design and synthesis, structure optimization, and action mechanism of lycogalic acid analogues, which lays a foundation for the application of these compounds as novel plant disease protective agents.

## Figures and Tables

**Table 1:** In vitro antifungal activities of compounds **5**, **8a**–**8s**, and carbendazim at 50 μg/mL ^1^.

Compound	Inhibition Rate (%)						
	*F.C*. ^2^	*C.H.* ^2^	*P.P.* ^2^	*R.C.* ^2^	*A.S.* ^2^	*P.G.* ^2^	*S.S.* ^2^
**5**	20 ± 1	32 ± 1	49 ± 1	65 ± 1	25 ± 1	29 ± 1	75 ± 2
**8a**	26 ± 1	18 ± 1	0	26 ± 1	11 ± 1	29 ± 1	70 ± 1
**8b**	36 ± 1	24 ± 1	33 ± 1	79 ± 1	6 ± 1	14 ± 1	79 ± 1
**8c**	21 ± 1	38 ± 1	31 ± 1	53 ± 1	13 ± 1	50 ± 1	74 ± 1
**8d**	20 ± 1	18 ± 1	20 ± 1	66 ± 1	14 ± 1	43 ± 1	70 ± 1
**8e**	31 ± 1	26 ± 1	12 ± 1	62 ± 1	8 ± 1	14 ± 1	37 ± 1
**8f**	36 ± 1	24 ± 1	66 ± 1	79 ± 1	19 ± 1	29 ± 1	79 ± 1
**8g**	32 ± 1	47 ± 1	57 ± 1	92 ± 1	13 ± 1	43 ± 1	79 ± 1
**8h**	43 ± 1	44 ± 1	49 ± 1	91 ± 1	19 ± 1	69 ± 1	84 ± 1
**8i**	68 ± 1	44 ± 1	71 ± 1	82 ± 1	31 ± 1	50 ± 1	69 ± 1
**8j**	63 ± 1	35 ± 1	56 ± 1	78 ± 1	13 ± 1	43 ± 1	50 ± 1
**8k**	32 ± 1	18 ± 1	49 ± 1	20 ± 1	19 ± 1	43 ± 1	34 ± 1
**8l**	30 ± 1	15 ± 1	66 ± 1	75 ± 1	13 ± 1	79 ± 1	65 ± 1
**8m**	36 ± 1	41 ± 1	61 ± 1	82 ± 1	25 ± 1	50 ± 1	79 ± 1
**8n**	34 ± 1	35 ± 1	49 ± 1	71 ± 1	6 ± 1	29 ± 1	61 ± 1
**8o**	23 ± 1	12 ± 1	49 ± 1	36 ± 1	19 ± 1	14 ± 1	73 ± 1
**8p**	27 ± 1	12 ± 1	62 ± 1	46 ± 1	6 ± 1	14 ± 1	74 ± 1
**8q**	45 ± 1	50 ± 1	49 ± 1	66 ± 1	19 ± 1	14 ± 1	77 ± 1
**8r**	30 ± 1	35 ± 1	49 ± 1	66 ± 1	31 ± 1	43 ± 1	77 ± 1
**8s**	29 ± 1	24 ± 1	41 ± 1	40 ± 1	6 ± 1	43 ± 1	77 ± 1
**carbendazim**	100	52 ± 1	100	100	13 ± 1	100	93 ± 2

^1^ The experiments were repeated three times. All results are expressed as mean ± SD. ^2^ F.C. = *Fusarium oxysporium* f. sp. *Cucumeris*; C.H. = *Cercospora arachidicola hori*; P.P. = *Physalospora piricola*; R.C. = *Rhizoctonia cerealis*; A.S. = *Alternaria solani*; P.G. = *Pyricularia grisea*; S.S. = *Sclerotinia sclerotiorum*.

## Data Availability

All data used to support the findings of this study are included within the article and Supplementary Materials.

## Supplementary Materials

The following supporting information can be downloaded at: https://www.mdpi.com/article/10.3390/molecules29133150/s1, Section S1: Synthesis of compound **1**; Section S2: Detailed bioassay procedures for the in vitro antifungal activities; Section S3: Calculation procedures for molecular docking research; Section S4: Copies of NMR spectra (Figures S1–S128). References [41,42,43] were cited in Supplementary Materials.
