# Supplementary material for: Design, Synthesis, and Antifungal Activities of Phenylpyrrole Analogues Based on Alkaloid Lycogalic Acid
Source: Molecules. 2024 Jul 2;29(13):3150. doi: 10.3390/molecules29133150 (PMC11243374; doi:10.3390/molecules29133150)
Supplement: Supplementary file 1 [file molecules-29-03150-s001.zip › molecules-3071867-supplementary.pdf]

Supplementary Material

Design, Synthesis, and Antifungal Activities of Phenylpyrrole Analogues Based on Alkaloid Lycogalic Acid

Shuaiheng Zhang [1], Zhenghong Zhou [3], Tienan Wang [2,*], Aidang Lu [1,*]

1 School of Chemical Engineering and Technology, Hebei University of Technology, Tianjin 300401, China; 202231504078@stu.hebut.edu.cn (S. Z.); luaidang@hebut.edu.cn (A. L.)

2 Key Laboratory of Traditional Chinese Medicine Research and Development of Hebei Province, Institute of Traditional Chinese Medicine, Chengde Medical University, Chengde, 067000, China

3 State Key Laboratory of Elemento-Organic Chemistry, Research Institute of Elemento-Organic Chemistry, College of Chemistry, Nankai University, Tianjin 300071, China; z.h.zhou@nankai.edu.cn (Z. Z.)

* Correspondence: cdwangtienan@163.com (T. W.); luaidang@hebut.edu.cn (A. L.), Tel. +86-22-60202812 (A. L.)

Contents

Section S1: Synthesis of compound 1 ········ 4

Section S2: Detailed bioassay procedures for the in vitro antifungal activities ········ 4

Section S3: Calculation procedures for molecular docking research ········ 4

Section S4: Copies of NMR spectra (Figures S1–S128) ········ 6

Figures S1–S2. ^{1}H NMR and ^{13}C NMR spectra of **S2** ········ 6

Figures S3–S4. ^{1}H NMR and ^{13}C NMR spectra of **S3** ········ 7

Figures S5–S6. ^{1}H NMR and ^{13}C NMR spectra of **1** ········ 8

Figures S7–S8. ^{1}H NMR and ^{13}C NMR spectra of **2** ········ 9

Figures S9–S10. ^{1}H NMR and ^{13}C NMR spectra of **3** ········ 10

Figures S11–S12. ^{1}H NMR and ^{13}C NMR spectra of **4** ········ 11

Figures S13–S14. ^{1}H NMR and ^{13}C NMR spectra of **5** ········ 12

Figures S15–S16. ^{1}H NMR and ^{13}C NMR spectra of **6a** ········ 13

Figures S17–S18. ^{1}H NMR and ^{13}C NMR spectra of **6b** ········ 14

Figures S19–S20. ^{1}H NMR and ^{13}C NMR spectra of **6c** ········ 15

Figures S21–S22. ^{1}H NMR and ^{13}C NMR spectra of **6d**······16

Figures S23–S24. ^{1}H NMR and ^{13}C NMR spectra of **6e**······17

Figures S25–S26. ^{1}H NMR and ^{13}C NMR spectra of **6f**······18

Figures S27–S28. ^{1}H NMR and ^{13}C NMR spectra of **6g**······19

Figures S29–S30. ^{1}H NMR and ^{13}C NMR spectra of **6h**······20

Figures S31–S32. ^{1}H NMR and ^{13}C NMR spectra of **6i**······21

Figures S33–S34. ^{1}H NMR and ^{13}C NMR spectra of **6j**······22

Figures S35–S36. ^{1}H NMR and ^{13}C NMR spectra of **6k**······23

Figures S37–S38. ^{1}H NMR and ^{13}C NMR spectra of **6l**······24

Figures S39–S40. ^{1}H NMR and ^{13}C NMR spectra of **6m**······25

Figures S41–S42. ^{1}H NMR and ^{13}C NMR spectra of **6n**······26

Figures S43–S44. ^{1}H NMR and ^{13}C NMR spectra of **6o**······27

Figures S45–S46. ^{1}H NMR and ^{13}C NMR spectra of **6p**······28

Figures S47–S48. ^{1}H NMR and ^{13}C NMR spectra of **6q**······29

Figures S49–S50. ^{1}H NMR and ^{13}C NMR spectra of **6r**······30

Figures S51–S52. ^{1}H NMR and ^{13}C NMR spectra of **6s**······31

Figures S53–S54. ^{1}H NMR and ^{13}C NMR spectra of **7a**······32

Figures S55–S56. ^{1}H NMR and ^{13}C NMR spectra of **7b**······33

Figures S57–S58. ^{1}H NMR and ^{13}C NMR spectra of **7c**······34

Figures S59–S60. ^{1}H NMR and ^{13}C NMR spectra of **7d**······35

Figures S61–S62. ^{1}H NMR and ^{13}C NMR spectra of **7e**······36

Figures S63–S64. ^{1}H NMR and ^{13}C NMR spectra of **7f**······37

Figures S65–S66. ^{1}H NMR and ^{13}C NMR spectra of **7g**······38

Figures S67–S68. ^{1}H NMR and ^{13}C NMR spectra of **7h**······39

Figures S69–S70. ^{1}H NMR and ^{13}C NMR spectra of **7i**······40

Figures S71–S72. ^{1}H NMR and ^{13}C NMR spectra of **7j**······41

Figures S73–S74. ^{1}H NMR and ^{13}C NMR spectra of **7k**······42

Figures S75–S76. ^{1}H NMR and ^{13}C NMR spectra of **7l**······43

Figures S77–S78. ^{1}H NMR and ^{13}C NMR spectra of **7m**······44

Figures S79–S80. ^{1}H NMR and ^{13}C NMR spectra of **7n**······45

Figures S81–S82. ^{1}H NMR and ^{13}C NMR spectra of **7o**······46

Figures S83–S84. ^{1}H NMR and ^{13}C NMR spectra of **7p**······47

Figures S85–S86. ^{1}H NMR and ^{13}C NMR spectra of **7q**······48

Figures S87–S88. ^{1}H NMR and ^{13}C NMR spectra of **7r**······49

Figures S89–S90. ^{1}H NMR and ^{13}C NMR spectra of **7s**······50

Figures S91–S92. ^{1}H NMR and ^{13}C NMR spectra of **8a**······51

Figures S93–S94. ^{1}H NMR and ^{13}C NMR spectra of **8b**······52

Figures S95–S96. ^{1}H NMR and ^{13}C NMR spectra of **8c**······53

Figures S97–S98. ^{1}H NMR and ^{13}C NMR spectra of **8d**······54

Figures S99–S100. ^{1}H NMR and ^{13}C NMR spectra of **8e**······55

Figures S101–S102. ^{1}H NMR and ^{13}C NMR spectra of **8f**······56

Figures S103–S104. ^{1}H NMR and ^{13}C NMR spectra of **8g**······57

Figures S105–S106. ^{1}H NMR and ^{13}C NMR spectra of **8h**······58

Figures S107–S108. ^{1}H NMR and ^{13}C NMR spectra of **8i**······59

Figures S109–S110. ^{1}H NMR and ^{13}C NMR spectra of **8j**······60

Figures S111–S112. ^{1}H NMR and ^{13}C NMR spectra of **8k**······61

Figures S113–S114. ^{1}H NMR and ^{13}C NMR spectra of **8l**······62

Figures S115–S116. ^{1}H NMR and ^{13}C NMR spectra of **8m**······63

Figures S117–S128. ^{1}H NMR and ^{13}C NMR spectra of **8n**······64

Figures S119–S120. ^{1}H NMR and ^{13}C NMR spectra of **8o**······65

Figures S121–S122. ^{1}H NMR and ^{13}C NMR spectra of **8p**······66

Figures S123–S124. ^{1}H NMR and ^{13}C NMR spectra of **8q**······67

Figures S125–S126. ^{1}H NMR and ^{13}C NMR spectra of **8r**······68

Figures S127–S128. ^{1}H NMR and ^{13}C NMR spectra of **8s**······69

Section S1: Synthesis of compound 1

Diethyl 2,2'-(benzylazanediyl)diacetate (**S2**). To a stirred solution of diethyl 2,2'-azanediyldiacetate (4.7 g, 25.0 mmol, 1.0 equiv.) in dimethyl sulfoxide (DMSO) (50 mL), K_2CO_3 (5.2 g, 37.5 mmol, 1.5 equiv.) and benzyl bromide (4.7 g, 27.5 mmol, 1.1 equiv.) were added, then the mixture was stirred at 60 °C for 12 h. After the reaction was completed, the solvent was removed in vacuum, and water was added to dilute, the aqueous phase was extracted with dichloromethane (CH_2Cl_2) (5 × 100 mL), the organic phases were combined, washed with saturated sodium chloride solution, dried with anhydrous sodium sulfate, and concentrated in vacuo to obtain compound **S2**. Yellow liquid, 79% yield; ^{1}H NMR (400 MHz, $CDCl_3$) δ 7.38 (d, *J* = 6.7 Hz, 1H), 7.30 (t, *J* = 7.3 Hz, 1H), 7.29–7.20 (m, 1H), 4.15 (q, *J* = 7.2 Hz, 3H), 3.91 (s, 2H), 3.54 (s, 3H), 1.25 (t, *J* = 7.2 Hz, 5H); ^{13}C NMR (100 MHz, $CDCl_3$) δ 171.2, 138.1, 129.1, 128.4, 127.4, 60.4, 57.8, 54.2, 14.3.

Diethyl 1-benzyl-3,4-dihydroxy-1*H*-pyrrole-2,5-dicarboxylate (**S3**). The compound **S2** (5.2 g, 17.4 mmol, 1.0 equiv.) and diethyl oxalate (3.1 g, 20.9 mmol, 1.2 equiv.) were added to the ethanol solution of sodium ethanol (w/w = 20%) (12.9 g, 37.2 mmol, 2.2 equiv.), at room temperature under nitrogen, and then the mixture was stirred at 85 °C for 6 h under nitrogen, glacial acetic acid was added until the solid was completely dissolved. The reaction mixture was then cooled to room temperature followed by the dropwise addition of water to afford a large amount of precipitation. The suspension was filtered to obtain compound **S3**. Yellow solid, 65% yield, m.p. 132–134 °C (lit.[41] 146 °C); ^{1}H NMR (DMSO-d_6, 400 MHz) δ 8.56 (s, 2H), 7.21 (dt, *J* = 29.4, 7.2 Hz, 3H), 6.85 (d, *J* = 7.1 Hz, 2H), 5.74 (s, 2H), 4.20 (q, *J* = 7.1 Hz, 4H), 1.19 (t, *J* = 7.1 Hz, 6H); ^{13}C NMR (100 MHz, DMSO-d_6) δ 160.2, 139.0, 138.5, 127.8 ,126.3, 125.2, 111.6, 59.6, 47.3, 13.7.

Diethyl 1-benzyl-3,4-bis(((trifluoromethyl)sulfonyl)oxy)-1*H*-pyrrole-2,5-dicarboxylate (**1**). The trifluoromethanesulfonic anhydride (6.2 g, 22.0 mmol, 2.2 equiv.) was added to a solution of compound **S3** (3.3 g, 10.0 mmol, 1.0 equiv.) in pyridine (30 mL) at 0 °C and stirred for 4 h. Subsequently water was added, filtered with suction, and recrystallized from ethanol to obtain compound **1**. Yellow solid, 84% yield, m.p. 63–66 °C; ^{1}H NMR (400 MHz, $CDCl_3$) δ 7.31 (t, *J* = 7.2 Hz, 2H), 7.30–7.22 (m, 1H), 6.99 (d, *J* = 7.2 Hz, 2H), 6.19 (s, 2H), 4.36 (q, *J* = 7.1 Hz, 4H), 1.34 (t, *J* = 7.2 Hz, 6H); ^{13}C NMR (100 MHz, $CDCl_3$) δ 157.9, 136.6, 128.8, 128.1, 127.7, 126.1, 117.9, 116.8, 62.5, 49.9, 13.8.

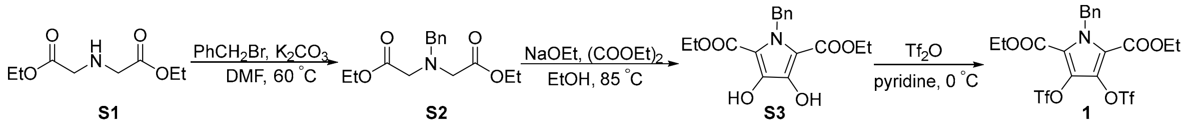

Scheme S1. Synthesis of the compound **1**

Section S2. Detailed bioassay procedures for the in vitro antifungal activities

The fungicidal activities of compounds were evaluated in mycelial growth tests conducted in artificial media against eight plant pathogens at a rate of 50 μg/mL. Each test compound was dissolved in a suitable amount of DMSO and diluted with water containing 0.1% TW-80 to a concentration of 500 μg/mL. To each petri dish was added 1 mL of the test solution and 9 mL of culture medium to make a 50 μg/mL concentration of the test compound, while in another petri dish was added 1 mL distilled water containing 0.1% TW-80 and 9 mL of culture medium as a blank control. A 4 mm diameter of hyphal growth was cut using a hole puncher on a growing fungal culture and the hyphae were moved to the petri dish containing the test compound. Each assay was performed three times. The dishes were stored in controlled environment cabinets (24±1°C) for 4 days, after which the diameter of mycelial growth was measured and the percentage inhibition was calculated using the following equation: Percentage inhibition (%) = (averaged diameter of mycelia in blank controls – averaged diameter of mycelia in medicated tablets) / (averaged diameter of mycelia in blank controls – 4 mm) × 100 [42].

Section S3. Calculation procedures for molecular docking research

The calculation procedures for molecular docking research consist of four steps [25].

Receptor Preparation. The protein sequence of the cytochrome P450 sterol 14α-demethylase enzyme of the *Rhizoctonia cerealis* (*Rc*CYP51) can be available from ref 43. The three-dimensional (3D) structure of *Rc*CYP51 was constructed based on *Saccharomyces cerevisiae* CYP51 crystal structure (PDB code: 4LXJ) as the template using SWISS-MODEL and this was used as the receptor for molecular docking. Water molecules were removed from the target protein and hydrogen atoms were added using AutoDock Tools prior to molecular docking.

Ligand preparation. Target compounds are drawn using ChemOffice 2017 as ligands followed by management of its conformer and the minimisation process.

Molecular Docking Using AutoDock Vina. The input files for AutoDock Vina were prepared using AutoDock Tools The protein was placed in a grid box (grid parameters: center x = 29.56, center y = 18.06, center z = 26.01, size x = 50, size y = 72, size z = 48), using AutoDock Vina at 1.00 Å to define the binding site. The docking procedure was performed using the instructed command prompts.

Analyzing and Output Visualisation using PyMOL. The docking poses were ranked according to their docking scores. The scoring function in Auto Dock was used to predict the binding affinity of one ligand to the receptor molecule. The conformation with the lowest binding affinity was selected for further analysis after the docking process. The docking results included the locations of hydrogen bonds and closely interacting residues were performed by PyMOL software.

References

41. Zong, K.; Reynolds, J.R. 3,4-Alkylenedioxypyrroles: Functionalized derivatives as monomers for new electron-rich conducting and electroactive polymers. *Journal of Organic Chemistry* **2001**, *66*(21), 6873–6882. doi: 10.1021/jo001620l.
42. Zhao, H.P.; Liu, Y.X.; Cui, Z.P.; Beattie, D.; Gu, Y.C.; Wang, Q.M. Design, synthesis, and biological activities of arylmethylamine substituted chlorotriazine and methylthiotriazine compounds. *Journal of Agricultural and Food Chemistry.* **2011**, *59* (21), 11711–11717. doi: 10.1021/jf203383s.
43. Xia, X. M. Studies on the Resistance Mechanism of Rhizoctonia cerealis to Tebuconazole; Shandong Agricultural University: China, 2006; p 101−102.

Section S4. Figures S1–S128. ^{1}H, and ^{13}C NMR spectra of S2–S3, and 1–8

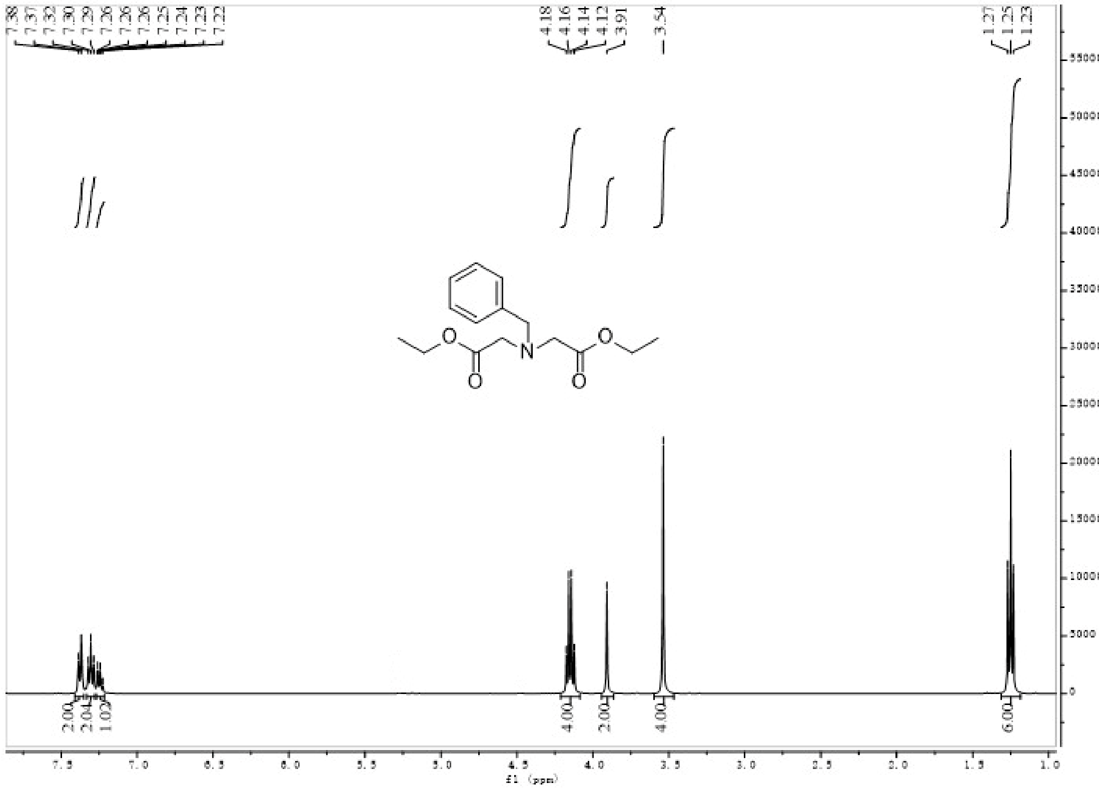

Figure S1. ^{1}H NMR spectrum of **S2**

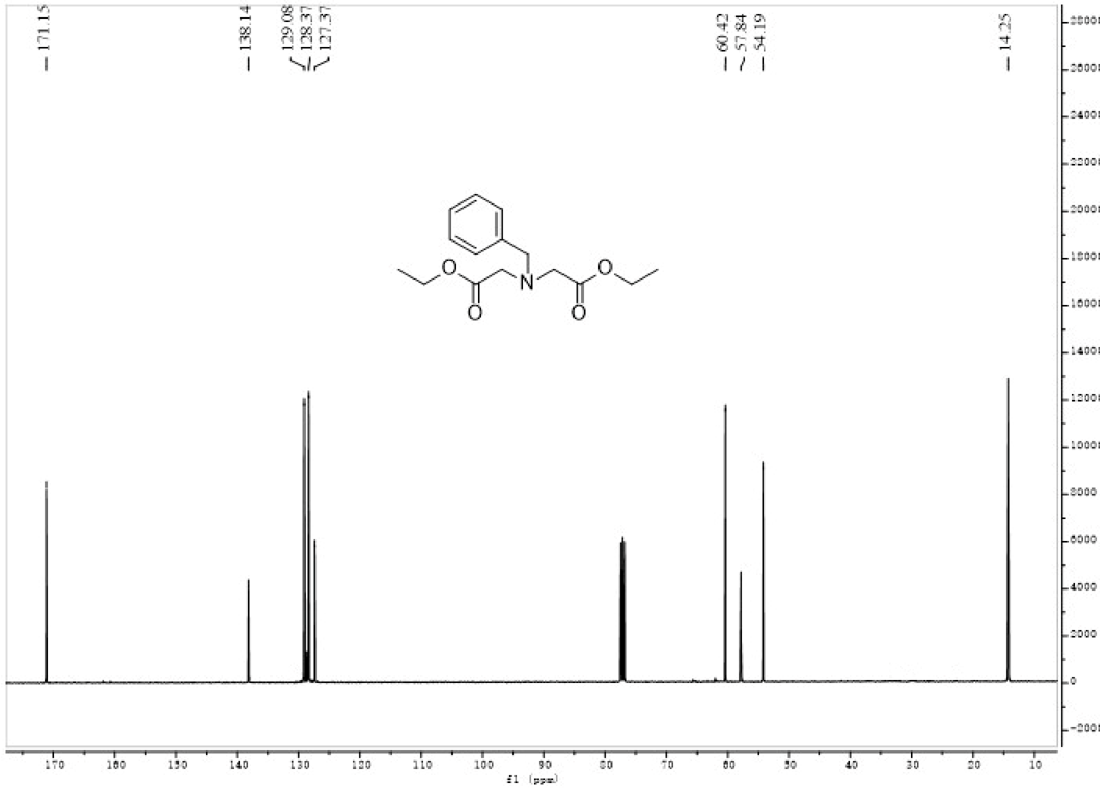

Figure S2. ^{13}C NMR spectrum of **S2**

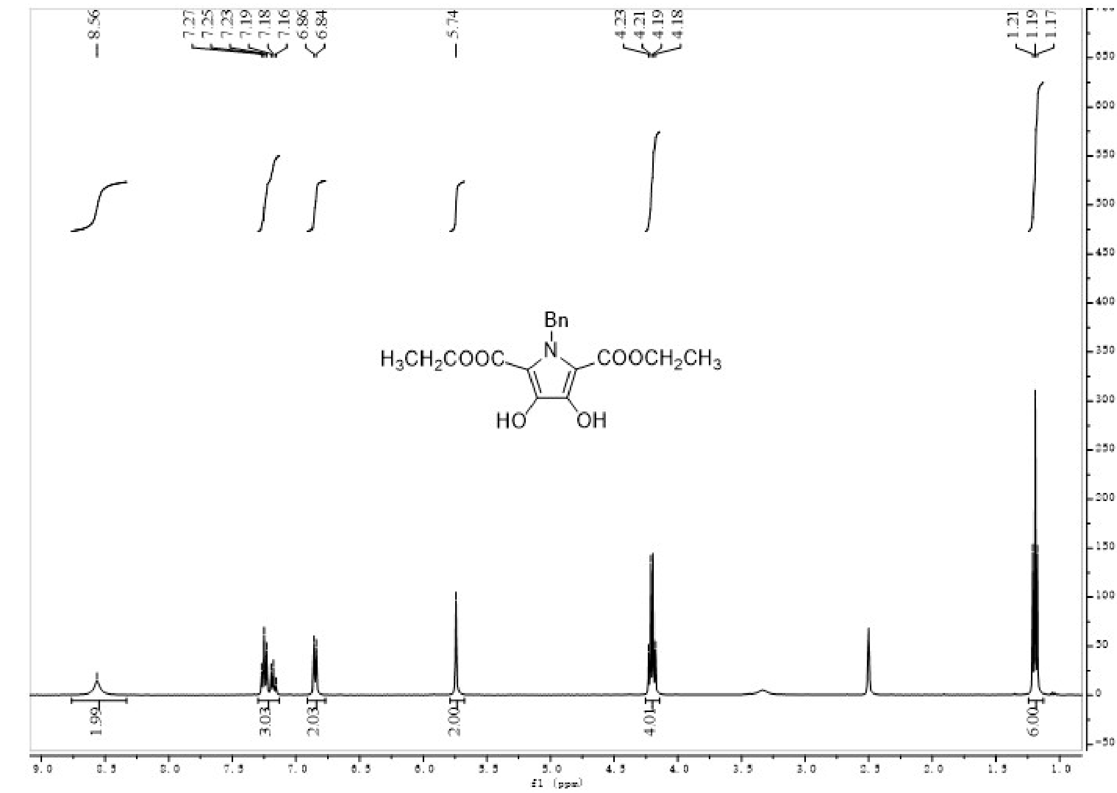

Figure S3. ^{1}H NMR spectrum of **S3**

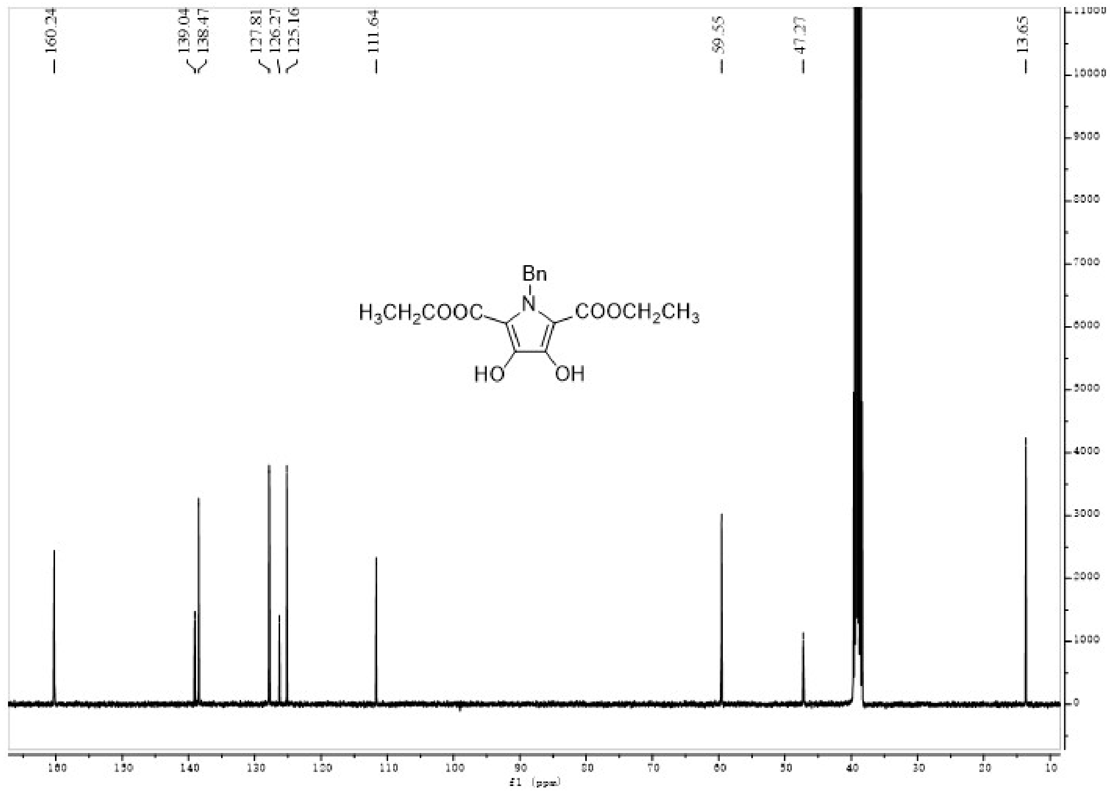

Figure S4. ^{13}C NMR spectrum of **S3**

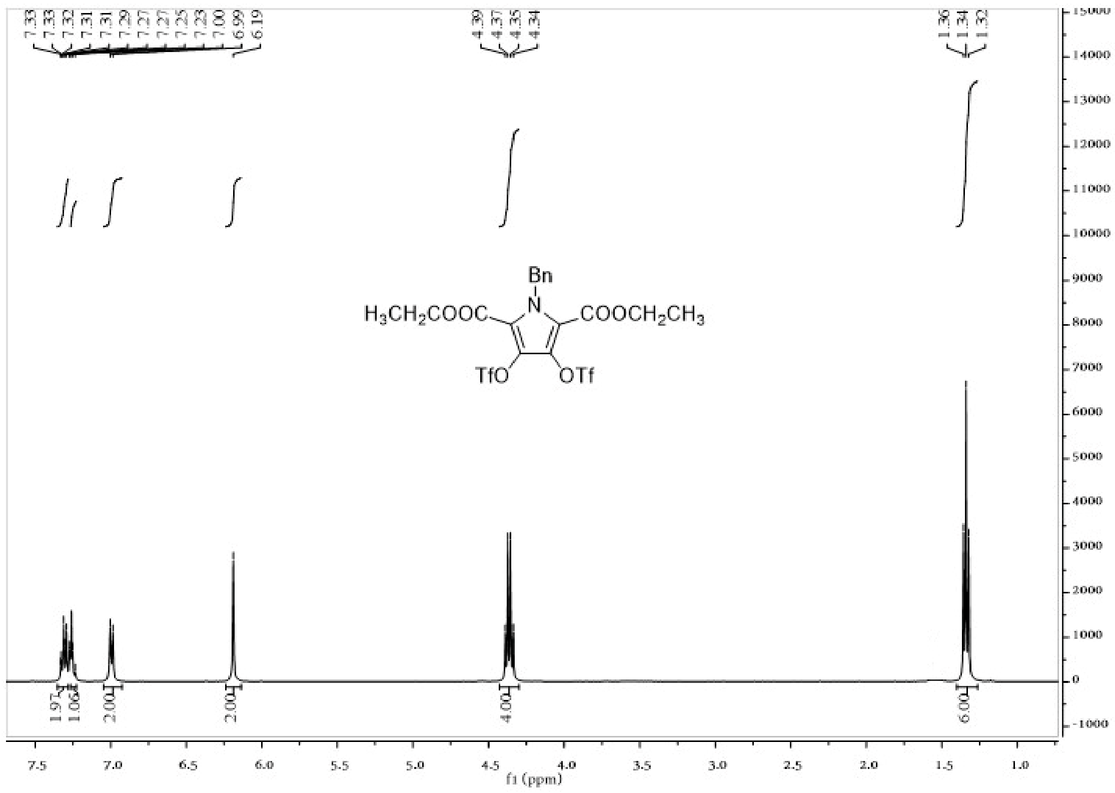

Figure S5. ^{1}H NMR spectrum of **1**

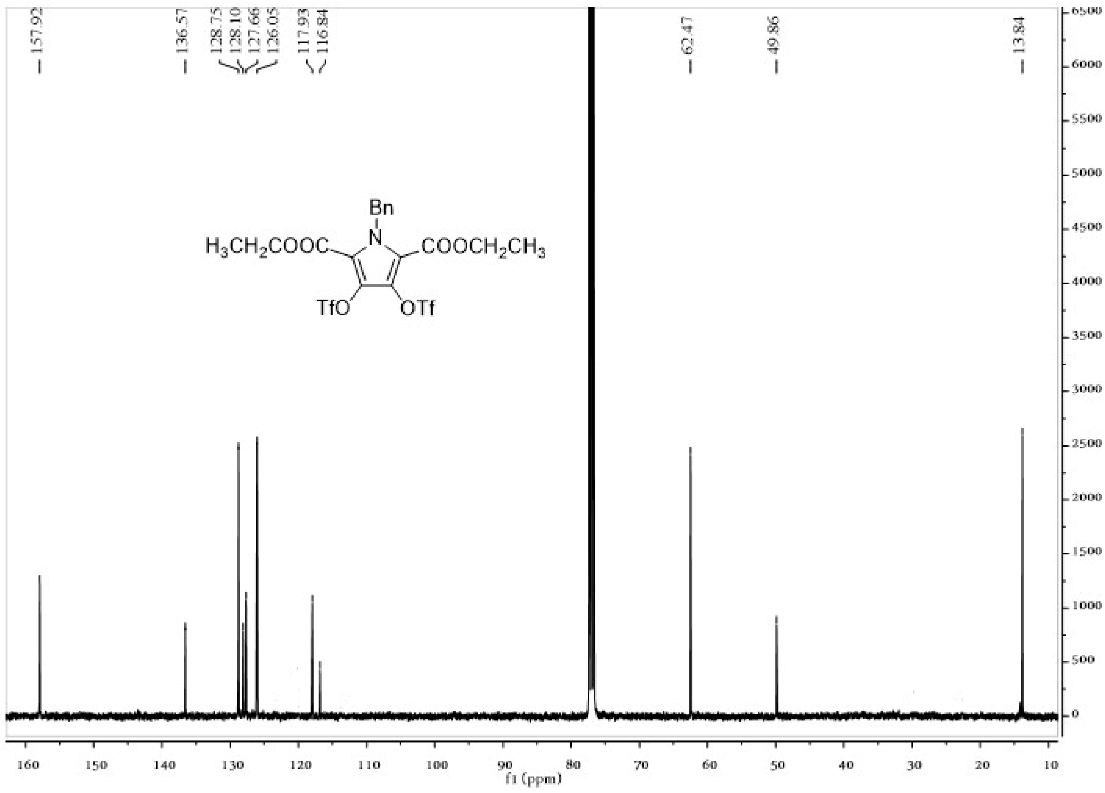

Figure S6. ^{13}C NMR spectrum of **1**

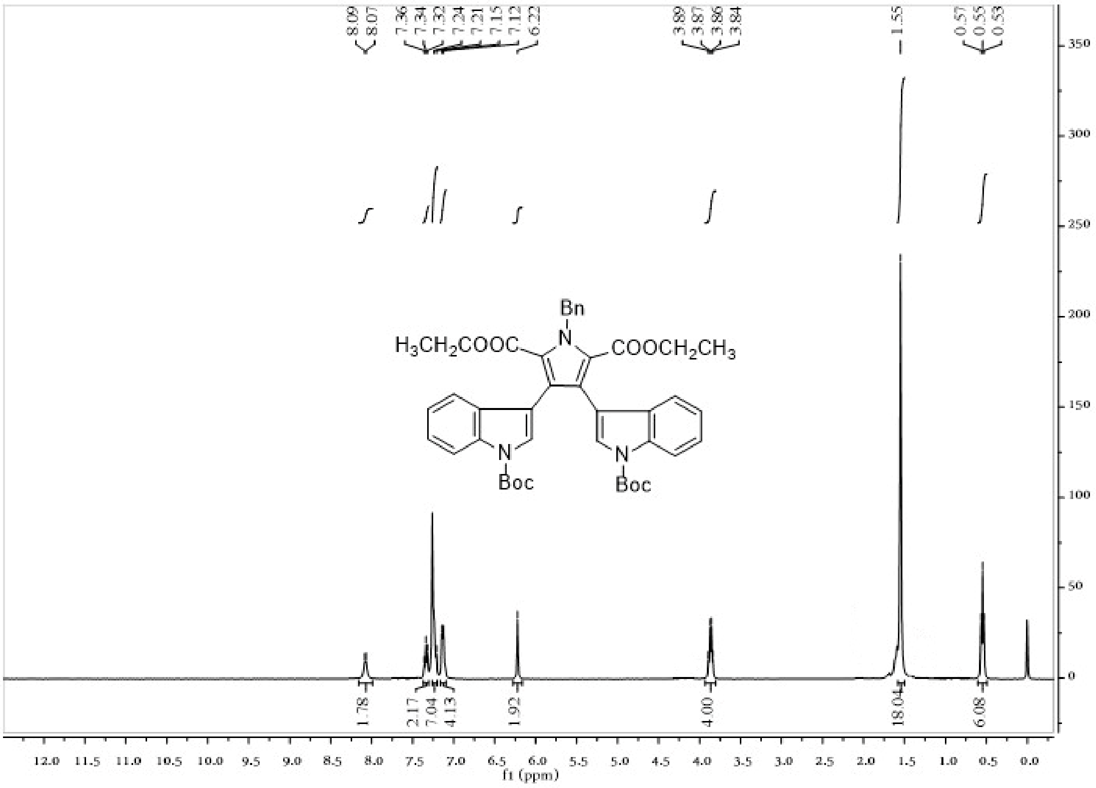

Figure S7. ^{1}H NMR spectrum of **2**

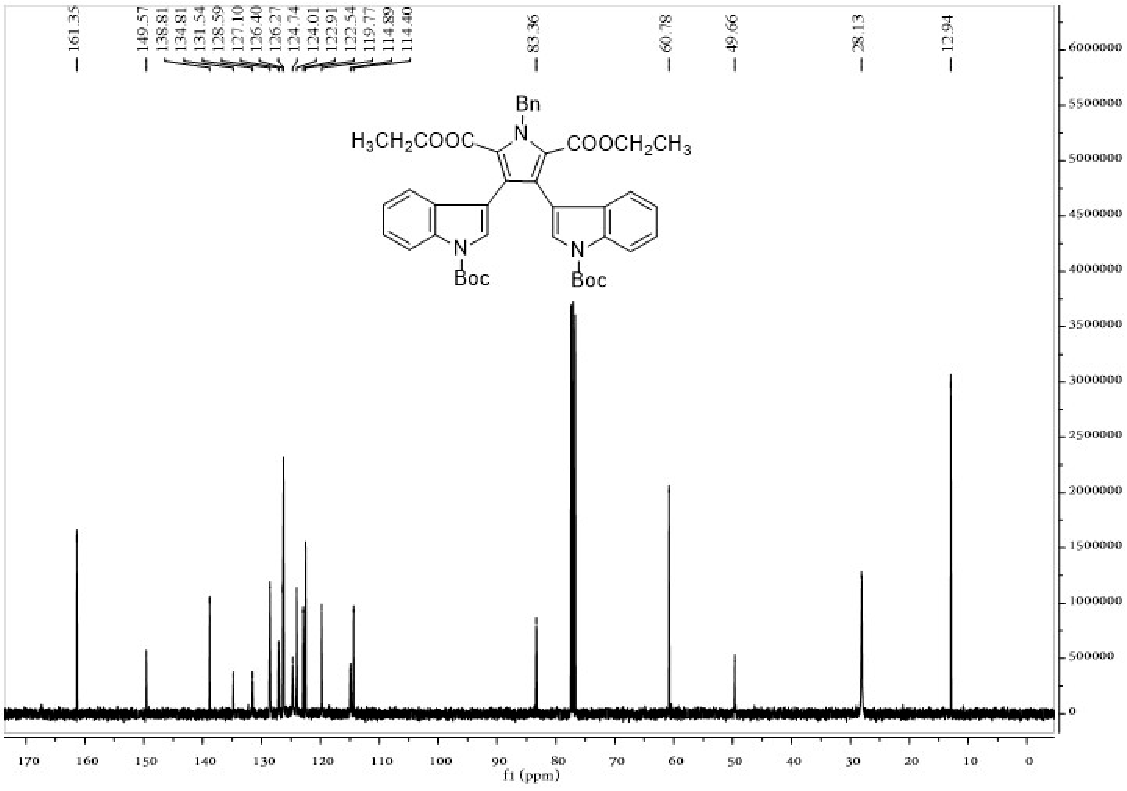

Figure S8. ^{13}C NMR spectrum of **2**

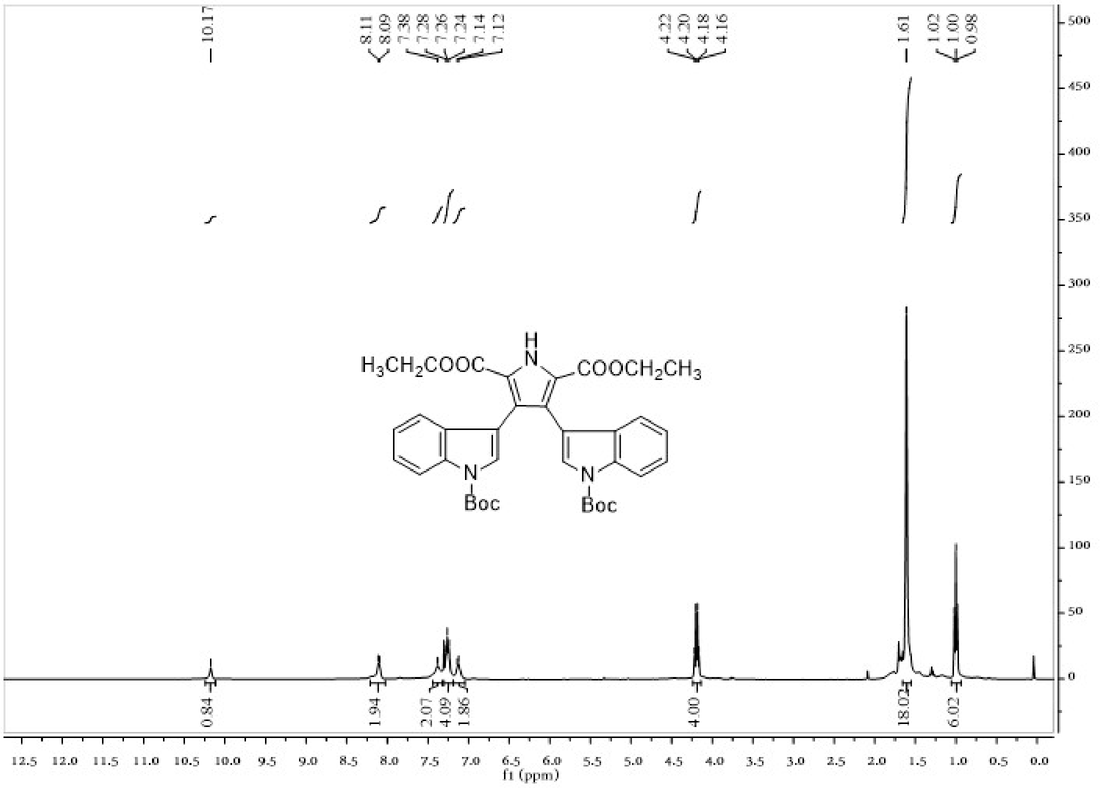

Figure S9. ^{1}H NMR spectrum of **3**

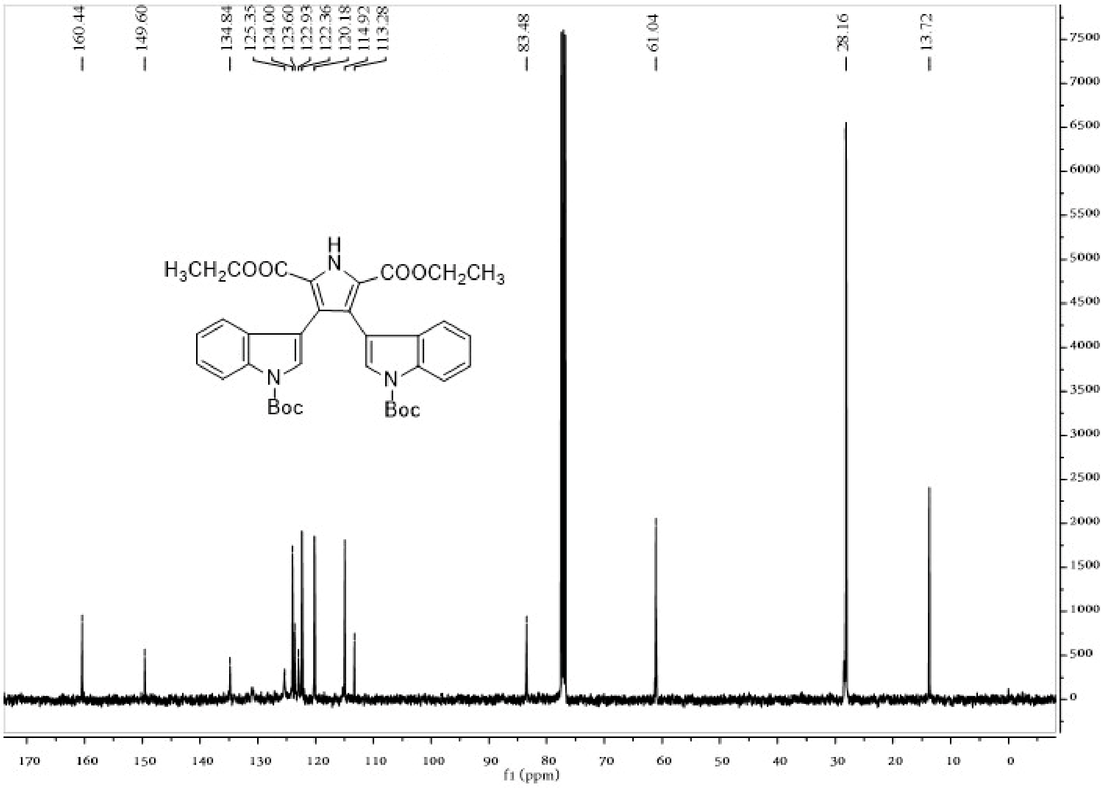

Figure S10. ^{13}C NMR spectrum of **3**

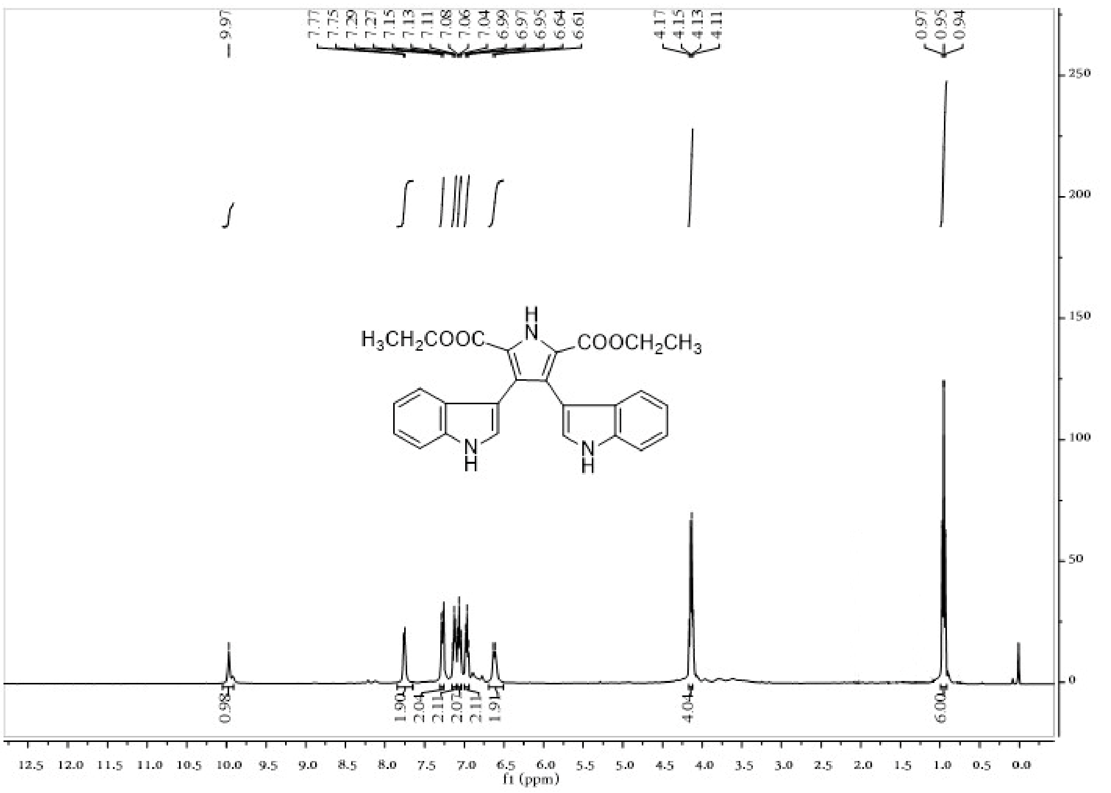

Figure S11. ^{1}H NMR spectrum of **4**

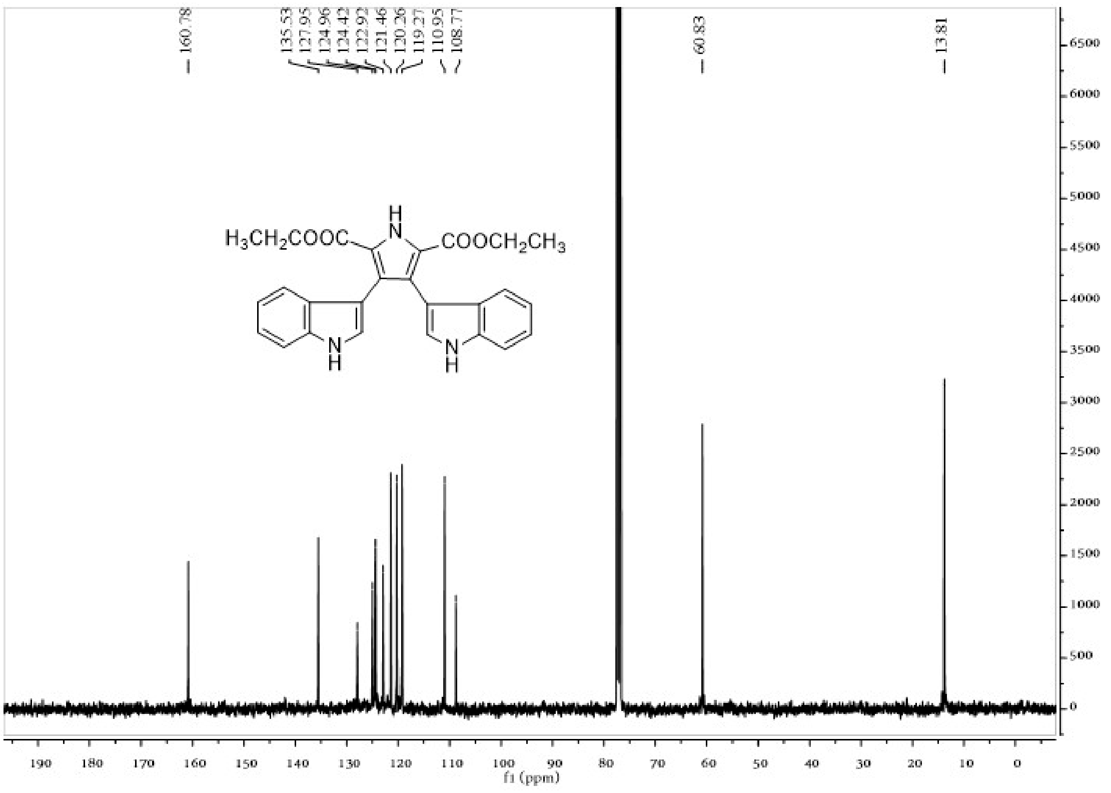

Figure S12. ^{13}C NMR spectrum of **4**

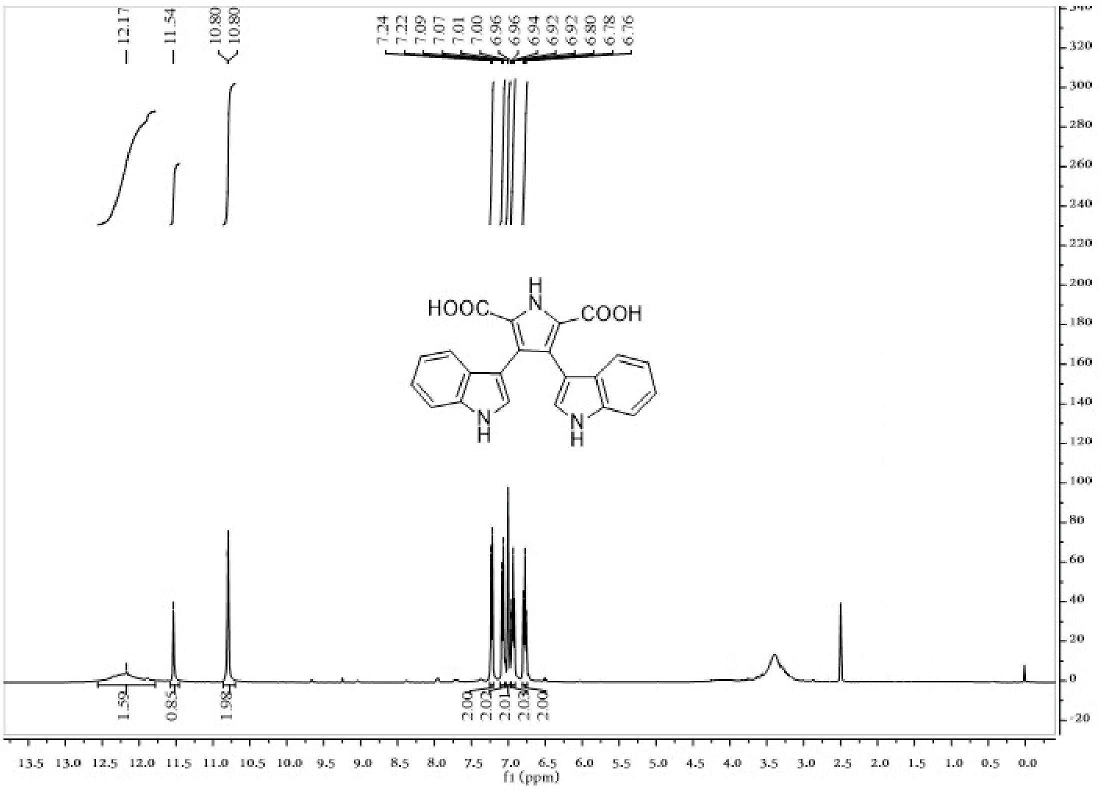

Figure S13. ^{1}H NMR spectrum of **5**

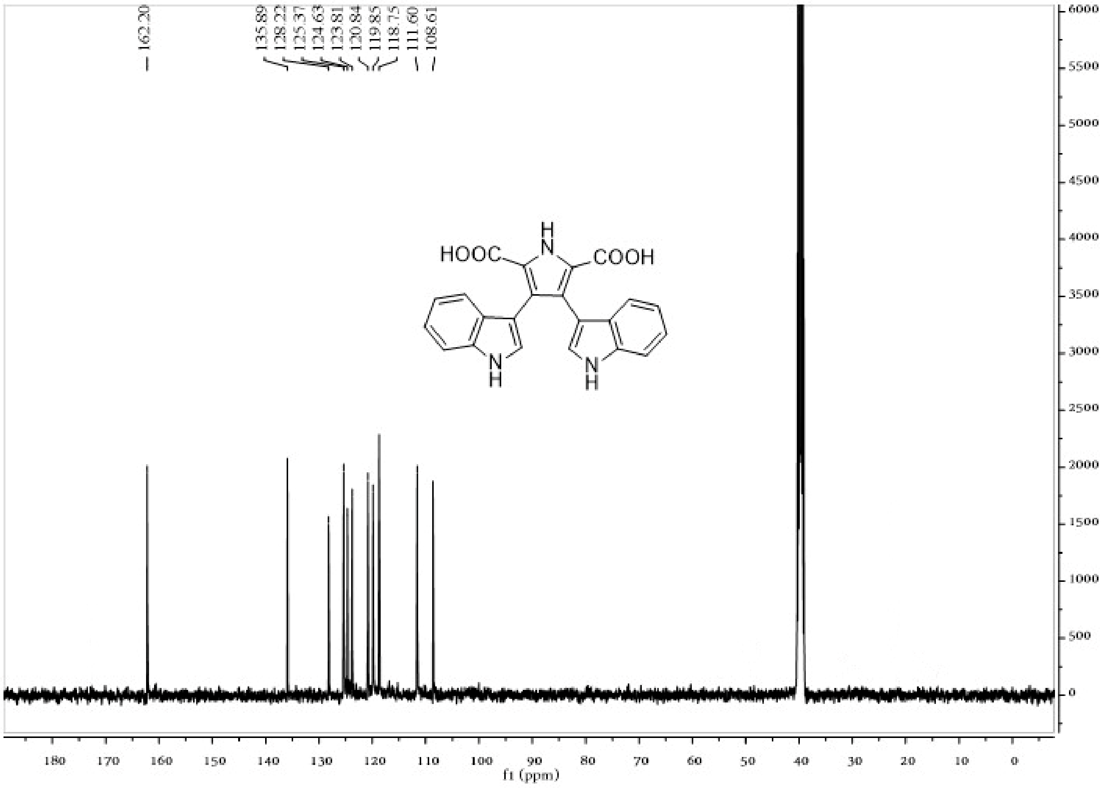

Figure S14. ^{13}C NMR spectrum of **5**

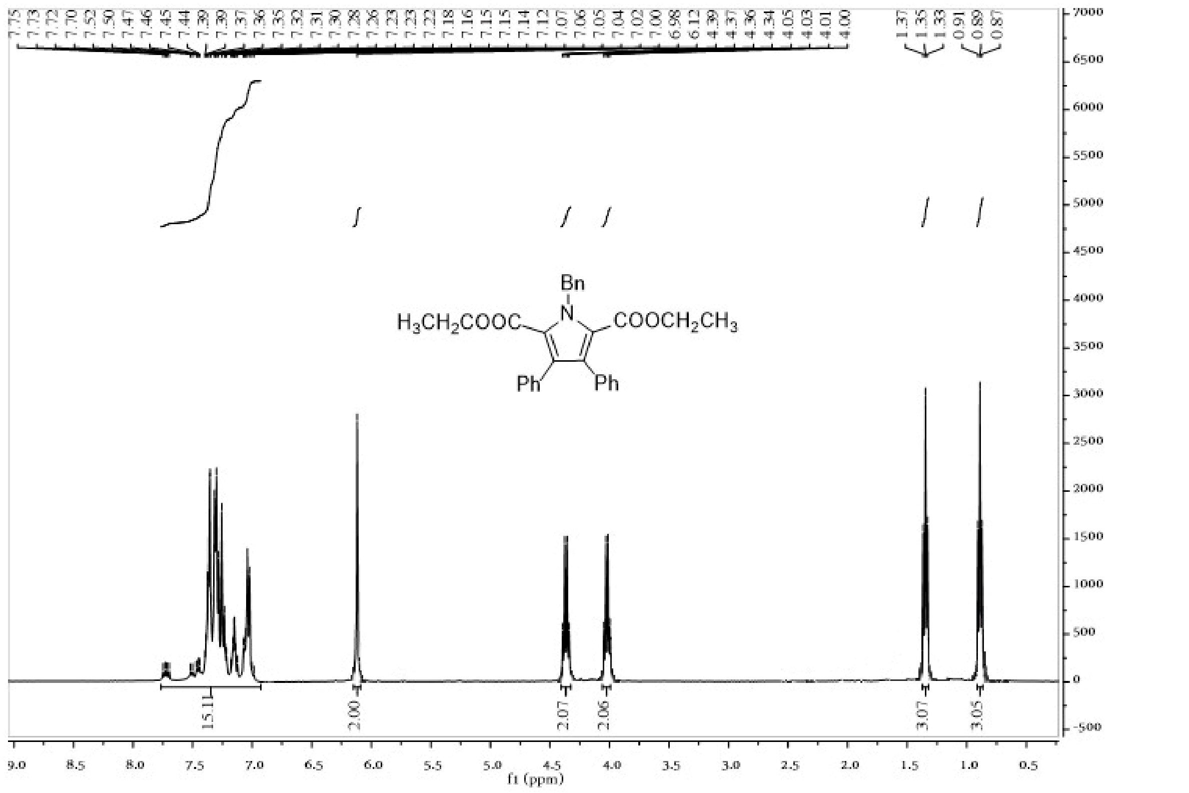

Figure S15. ^{1}H NMR spectrum of **6a**

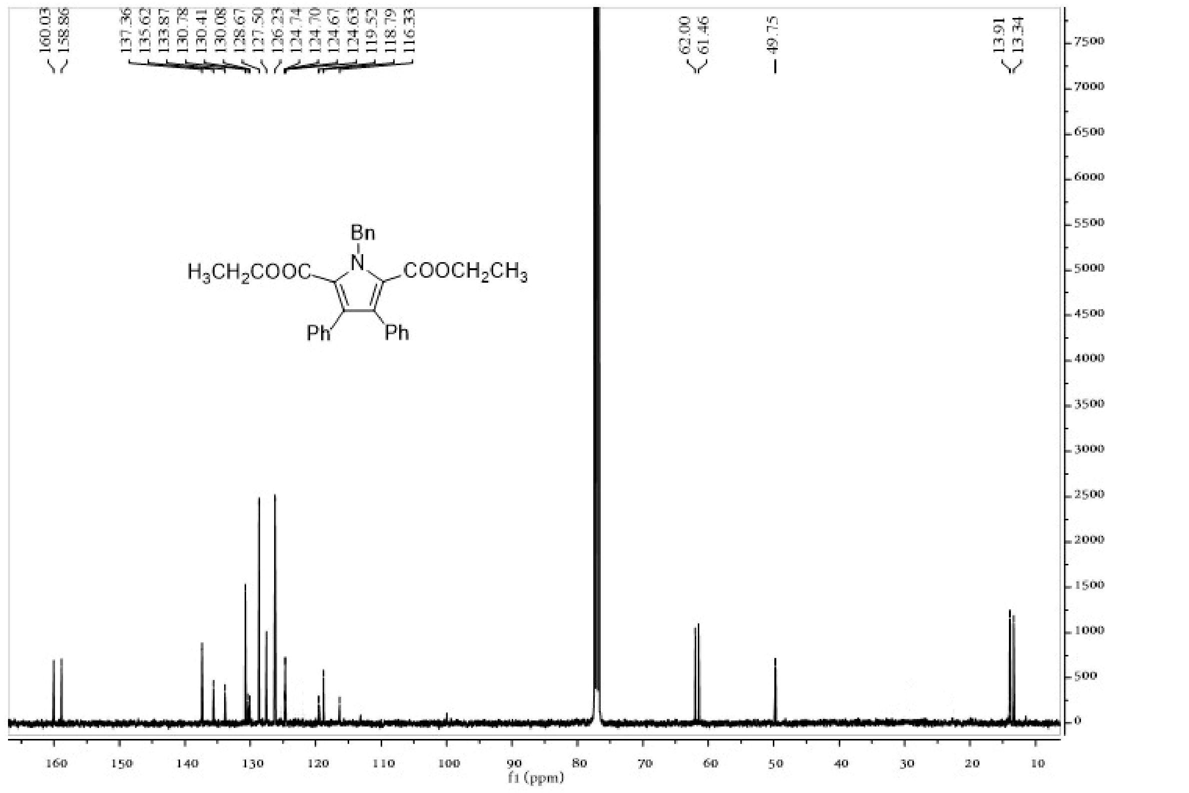

Figure S16 ^{13}C NMR spectrum of **6a**

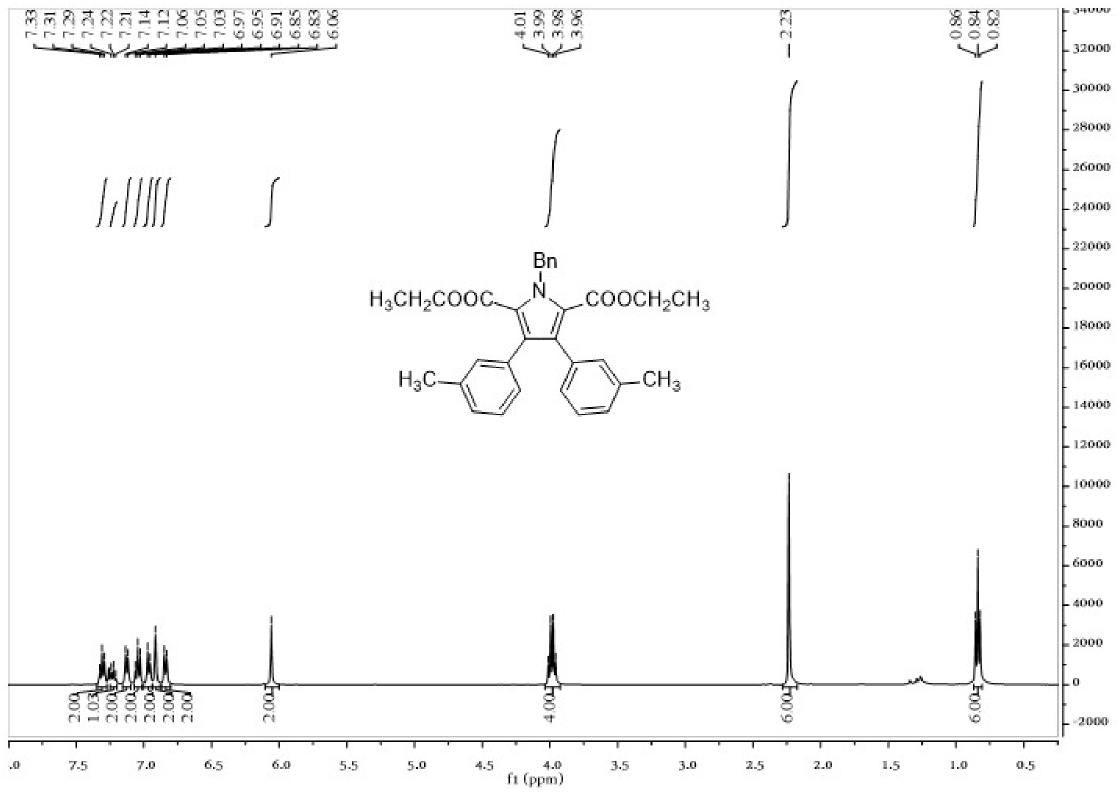

Figure S17. ^{1}H NMR spectrum of **6b**

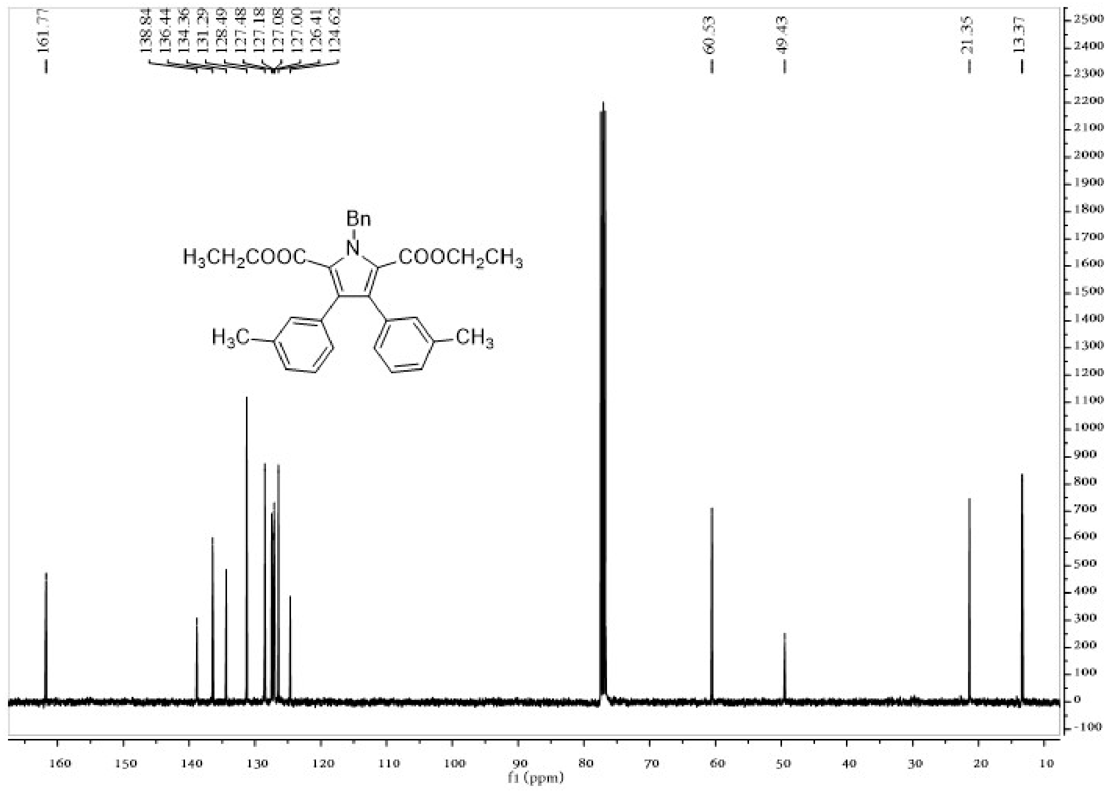

Figure S18. ^{13}C NMR spectrum of **6b**

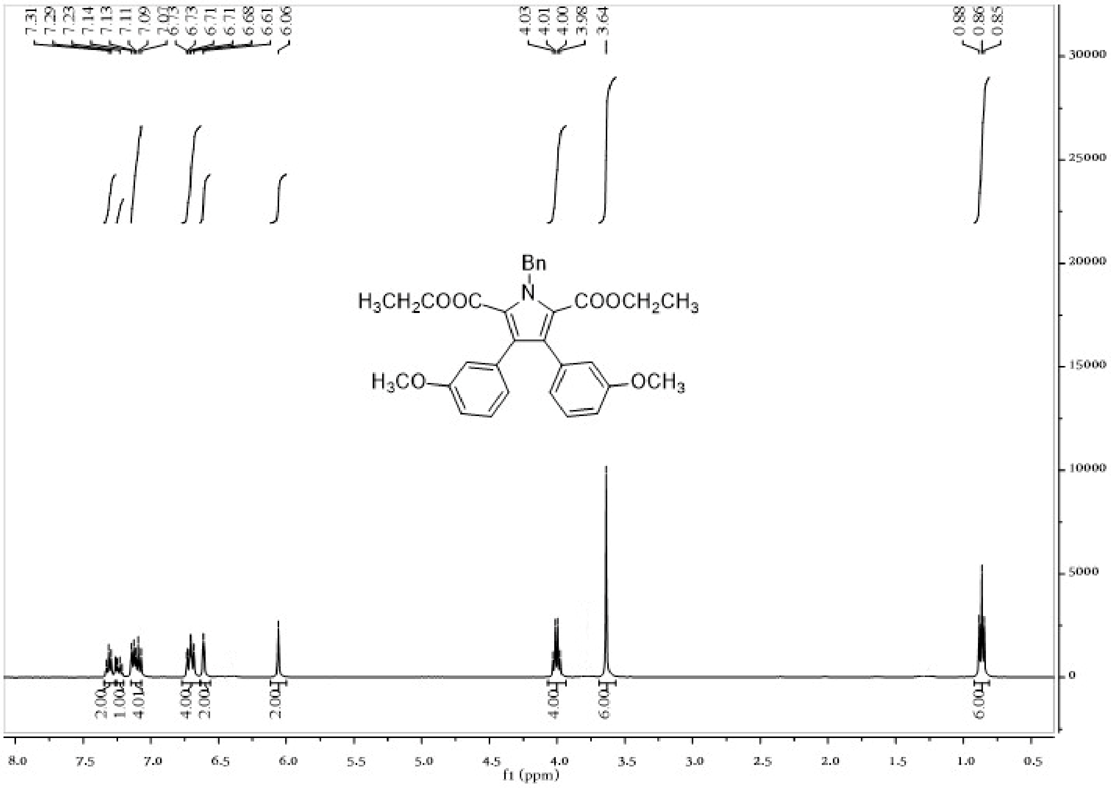

Figure S19. ^{1}H NMR spectrum of **6c**

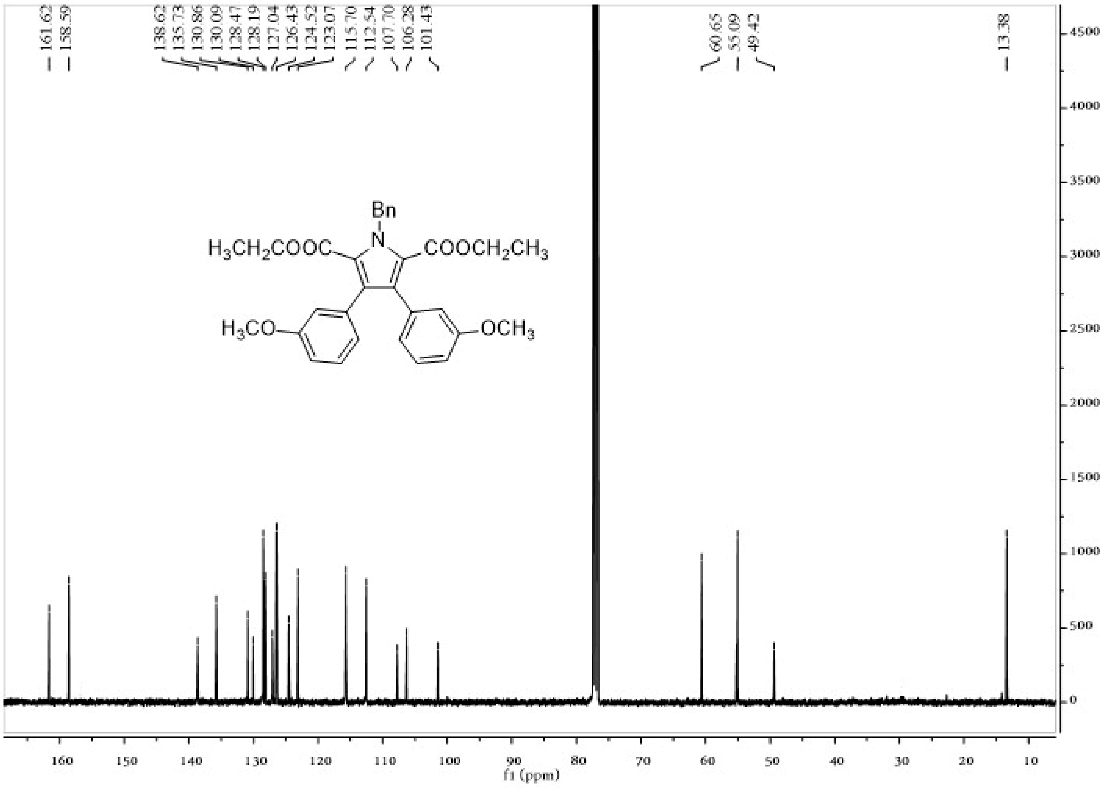

Figure S20. ^{13}C NMR spectrum of **6c**

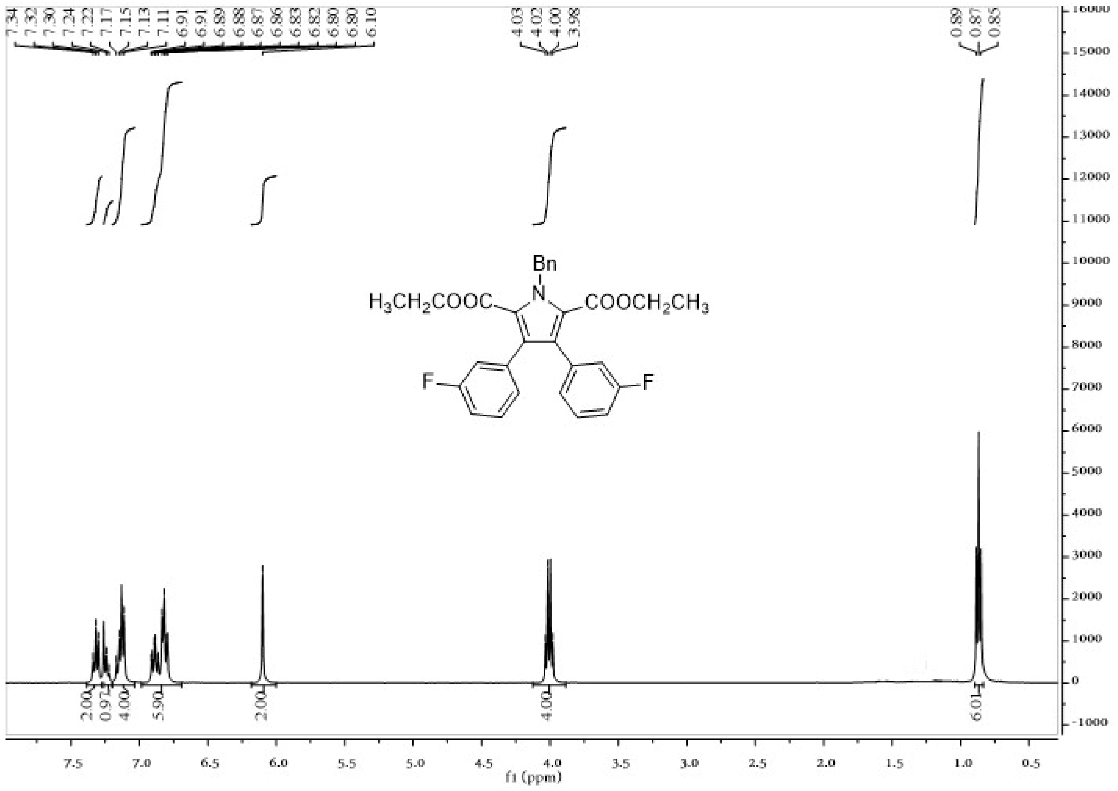

Figure S21. ^{1}H NMR spectrum of **6d**

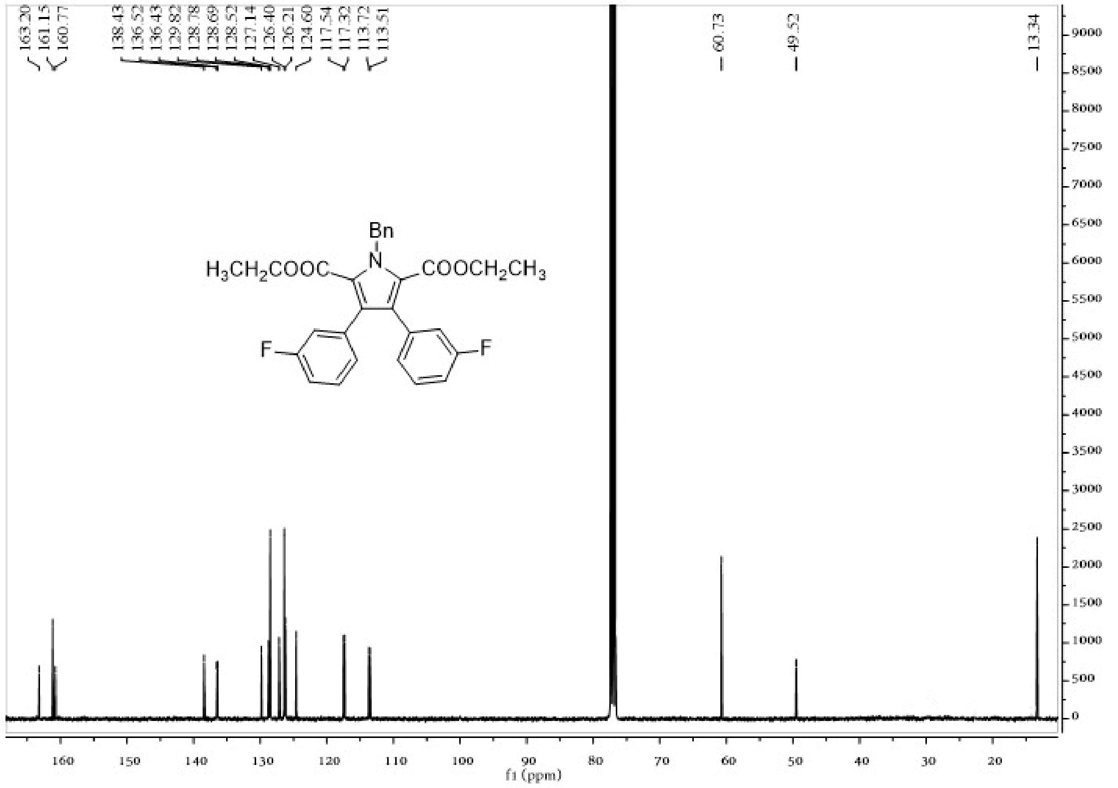

Figure S22. ^{13}C NMR spectrum of **6d**

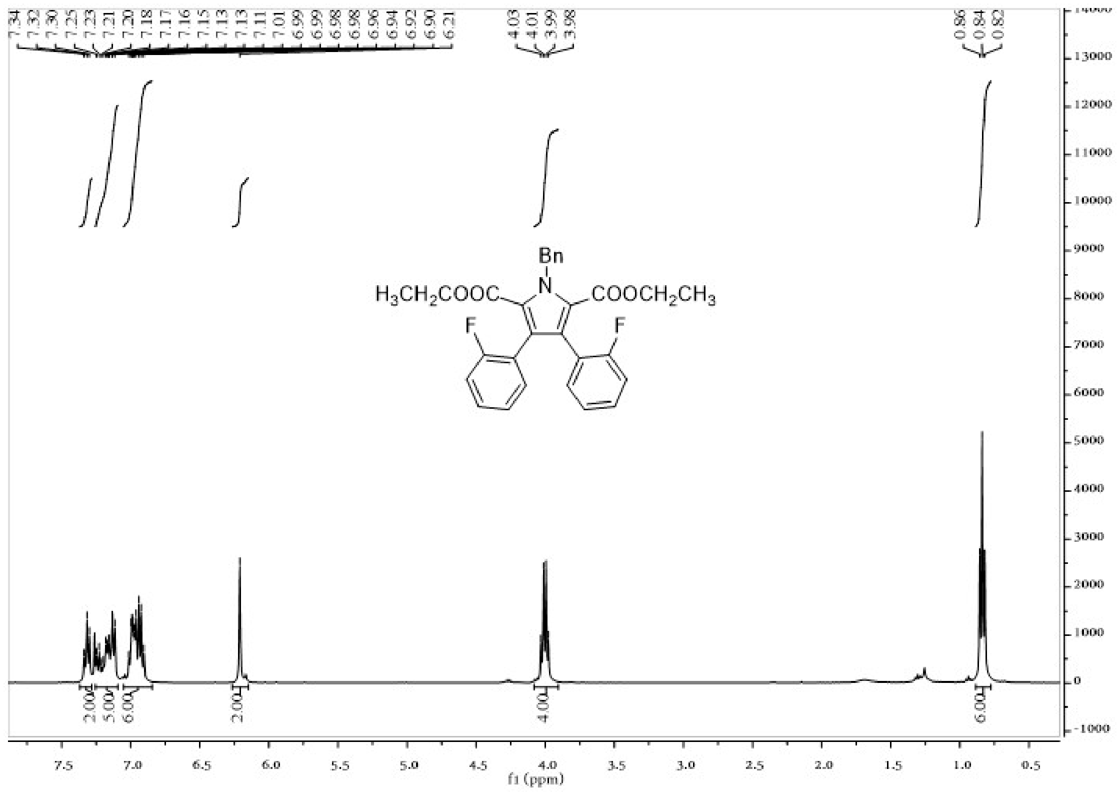

Figure S23. ^{1}H NMR spectrum of **6e**

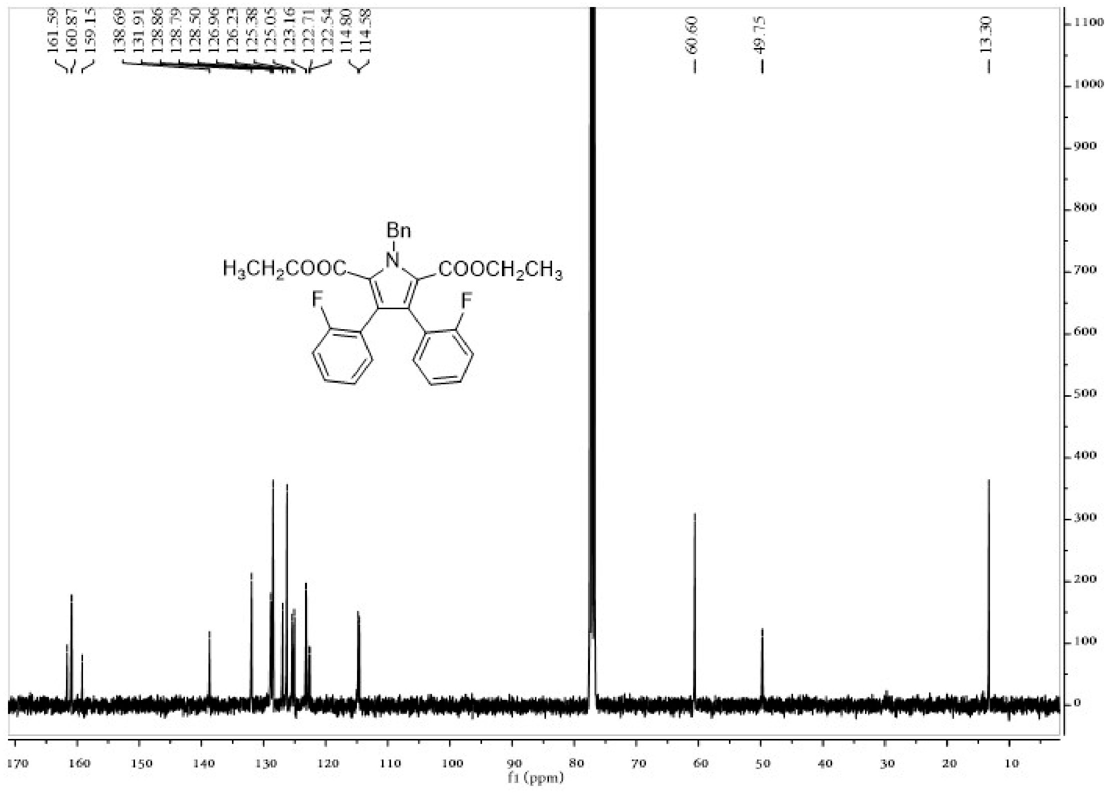

Figure S24. ^{13}C NMR spectrum of **6e**

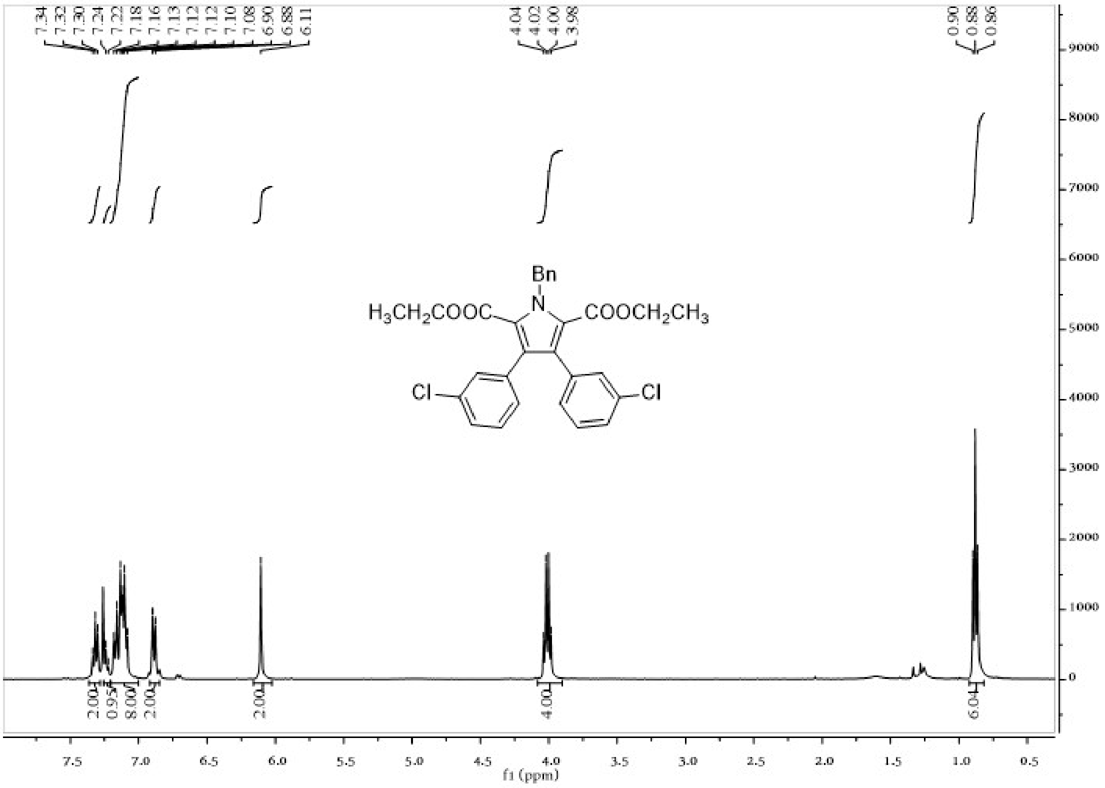

Figure S25. ^{1}H NMR spectrum of **6f**

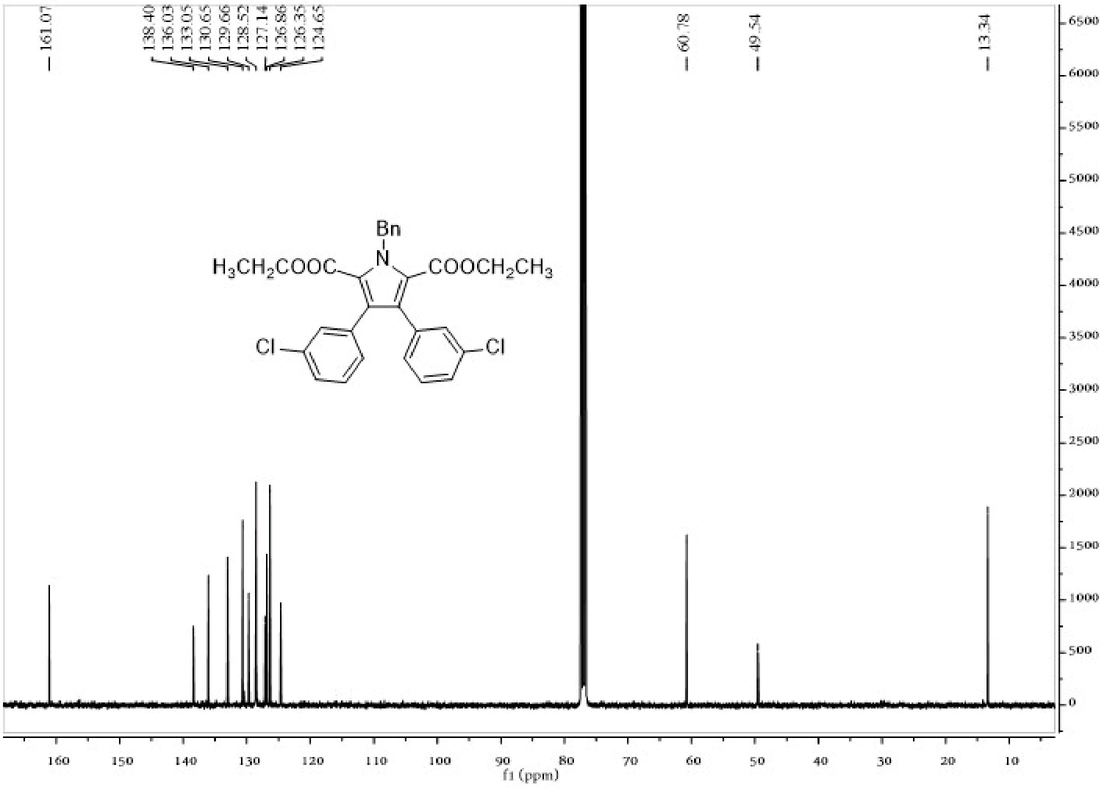

Figure S26. ^{13}C NMR spectrum of **6f**

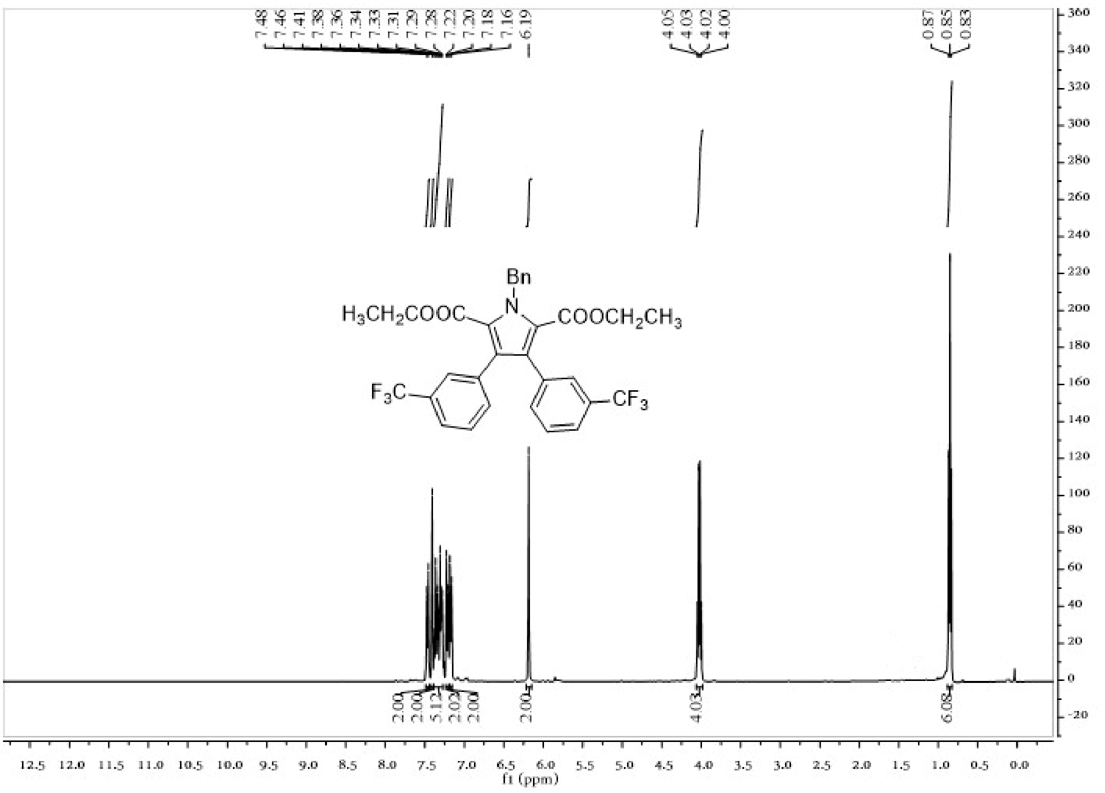

Figure S27. ¹H NMR spectrum of **6g**

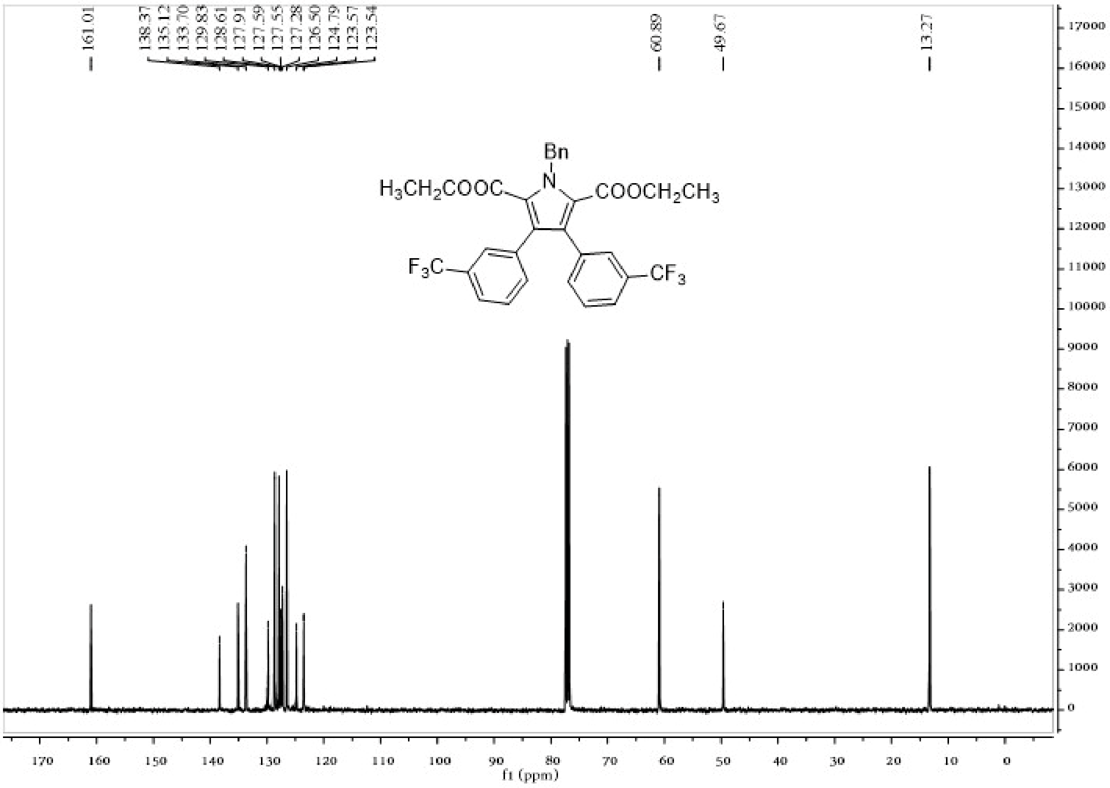

Figure S28. ¹³C NMR spectrum of **6g**

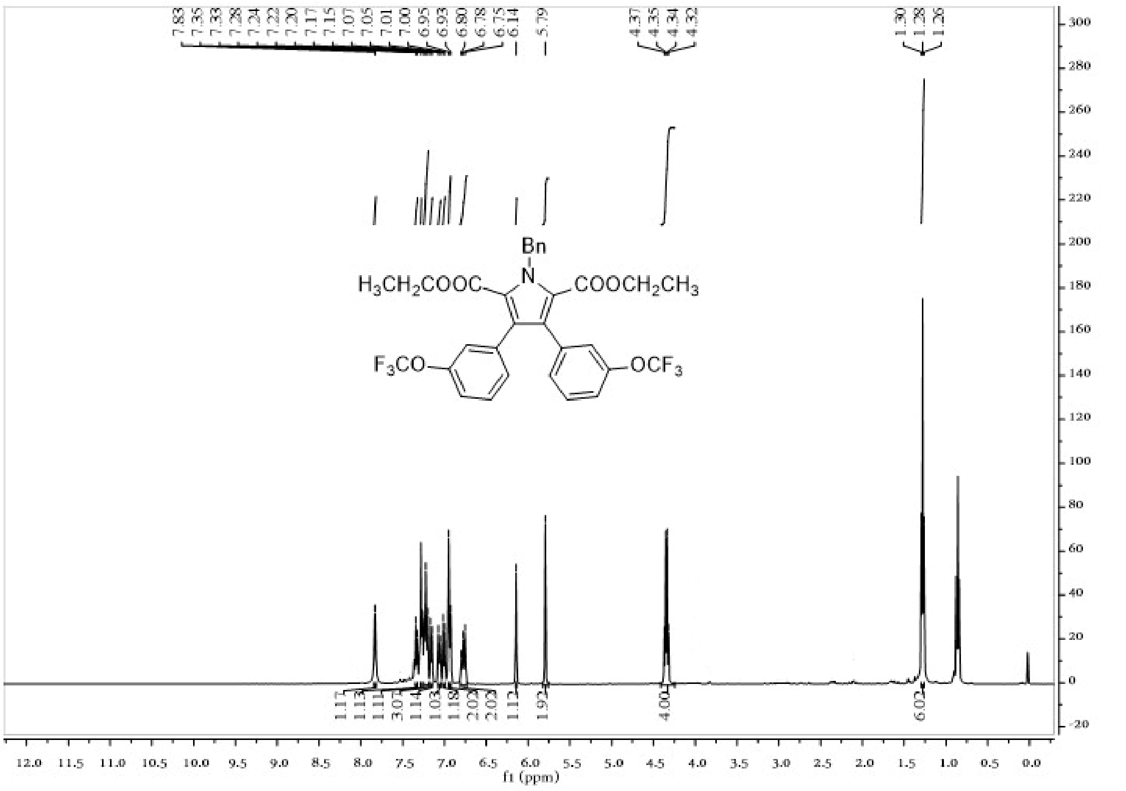

Figure S29. ^{1}H NMR spectrum of **6h**

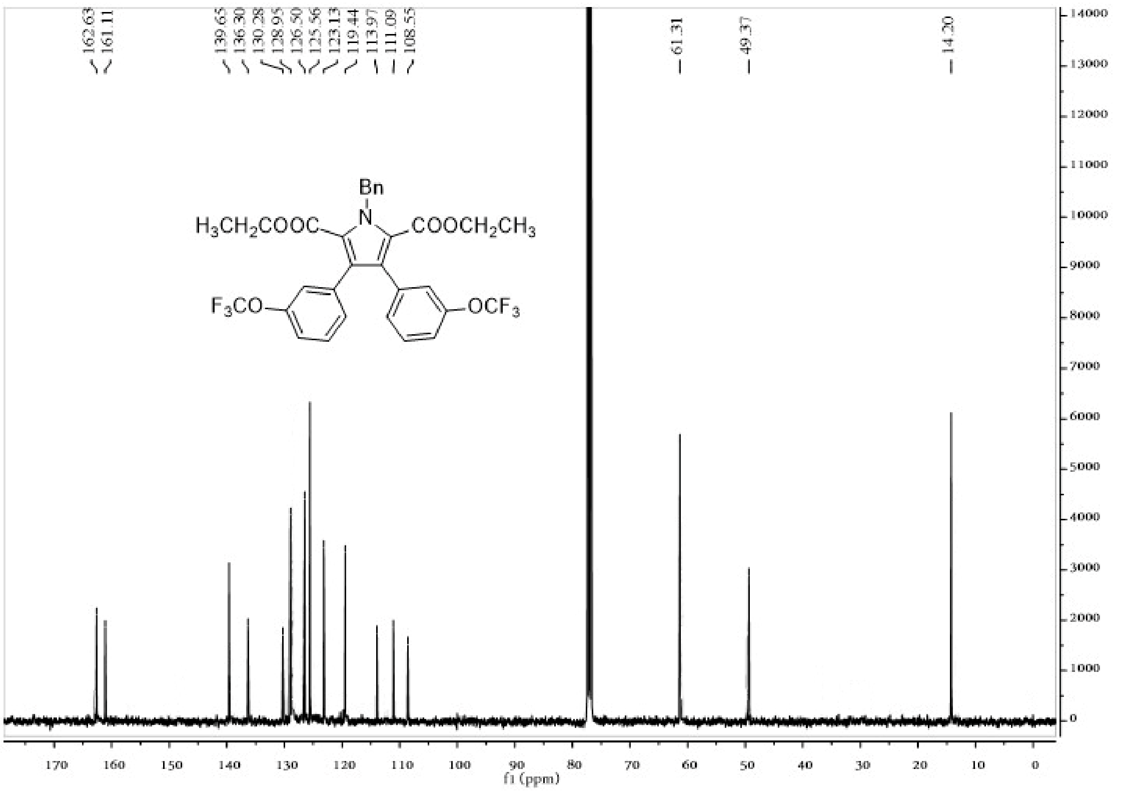

Figure S30. ^{13}C NMR spectrum of **6h**

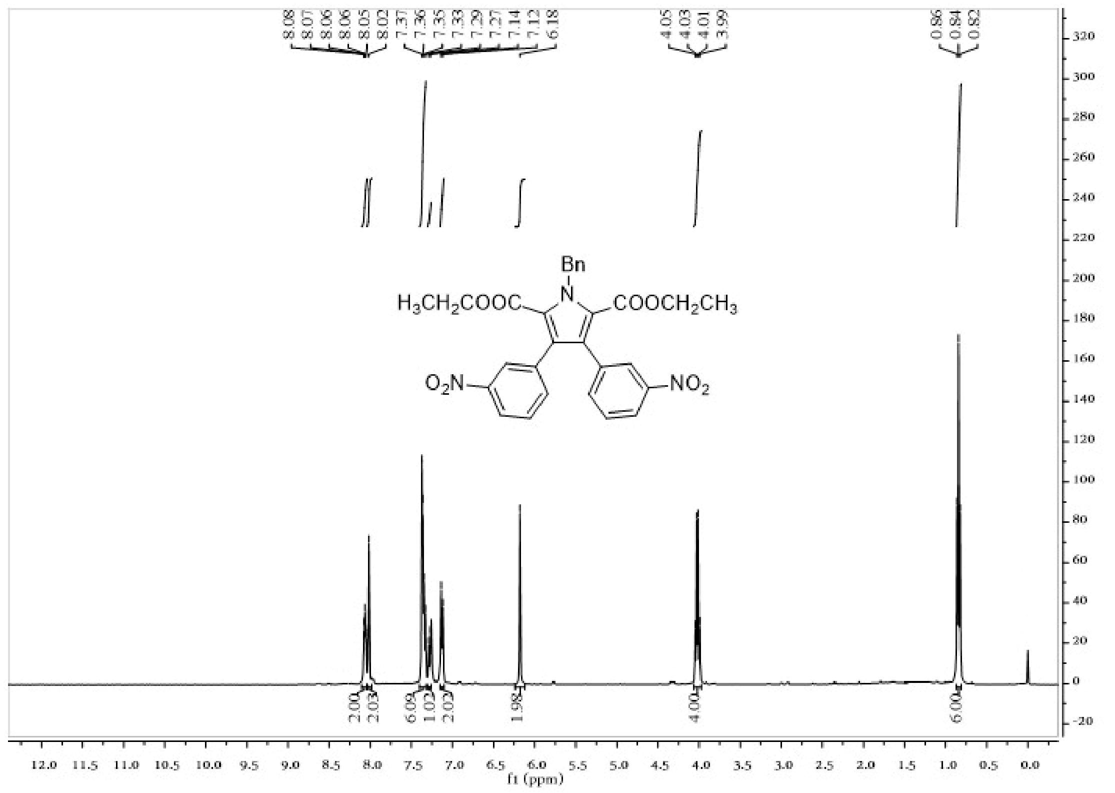

Figure S31. ^{1}H NMR spectrum of **6i**

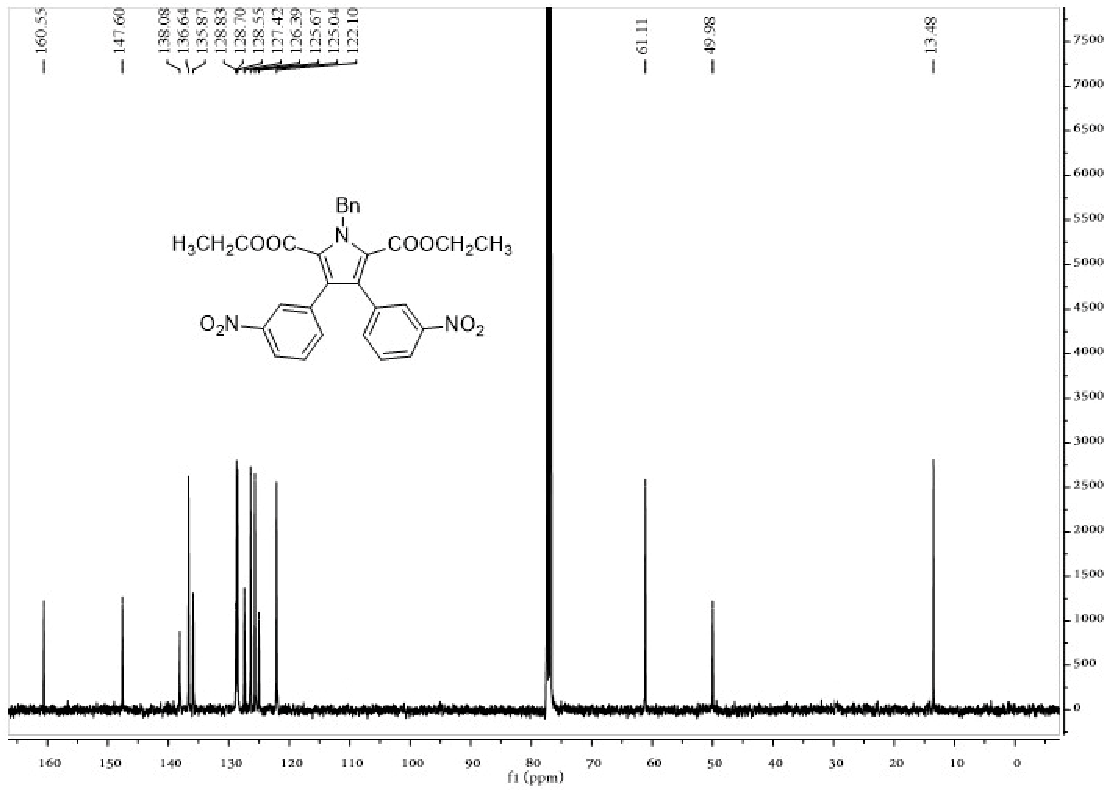

Figure S32. ^{13}C NMR spectrum of **6i**

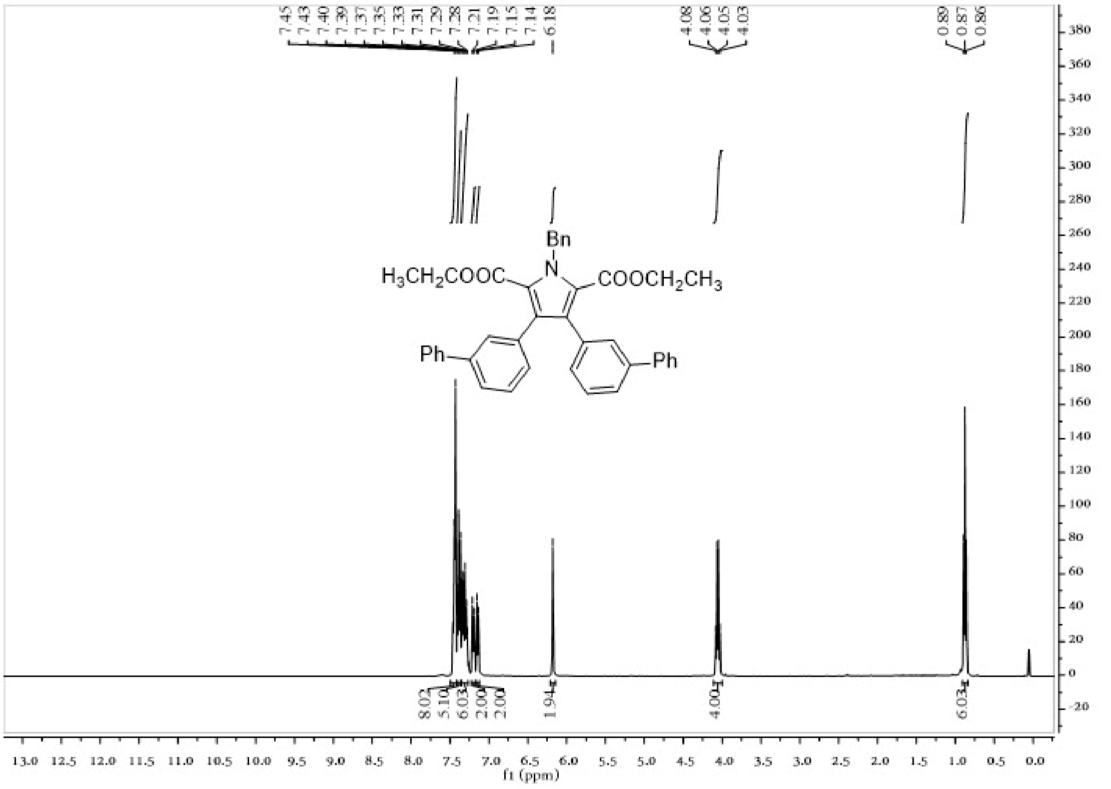

Figure S33. ^{1}H NMR spectrum of **6j**

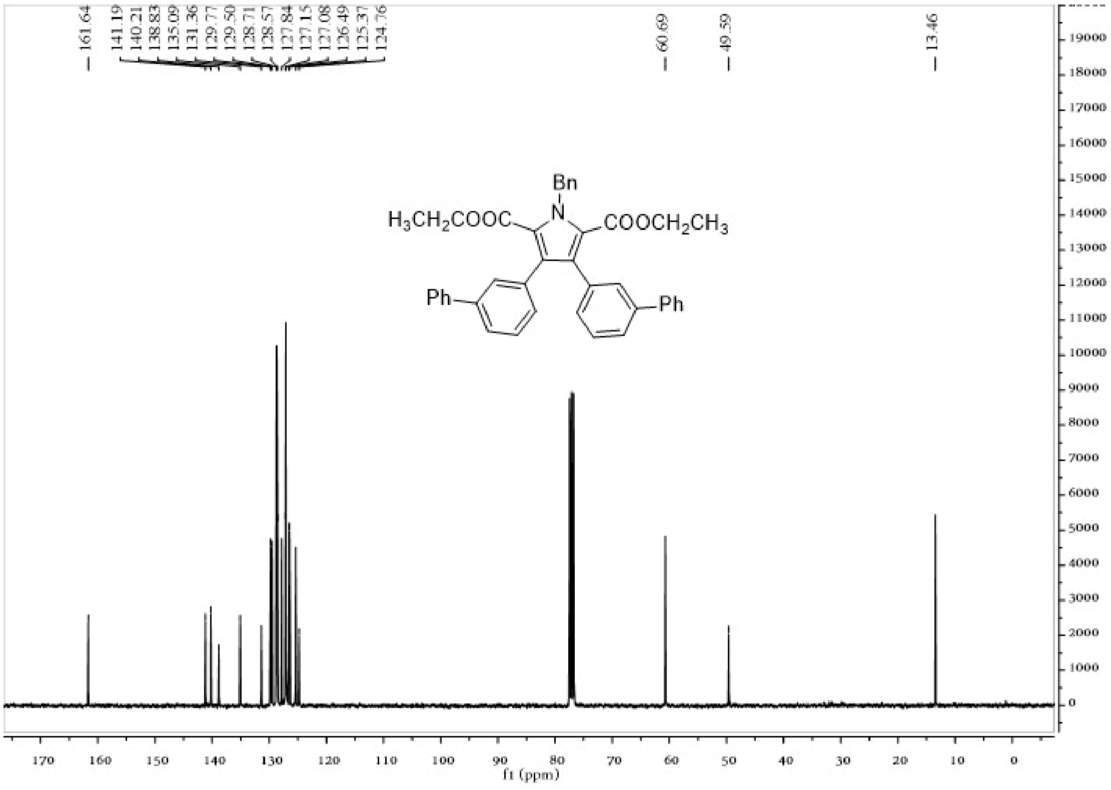

Figure S34. ^{13}C NMR spectrum of **6j**

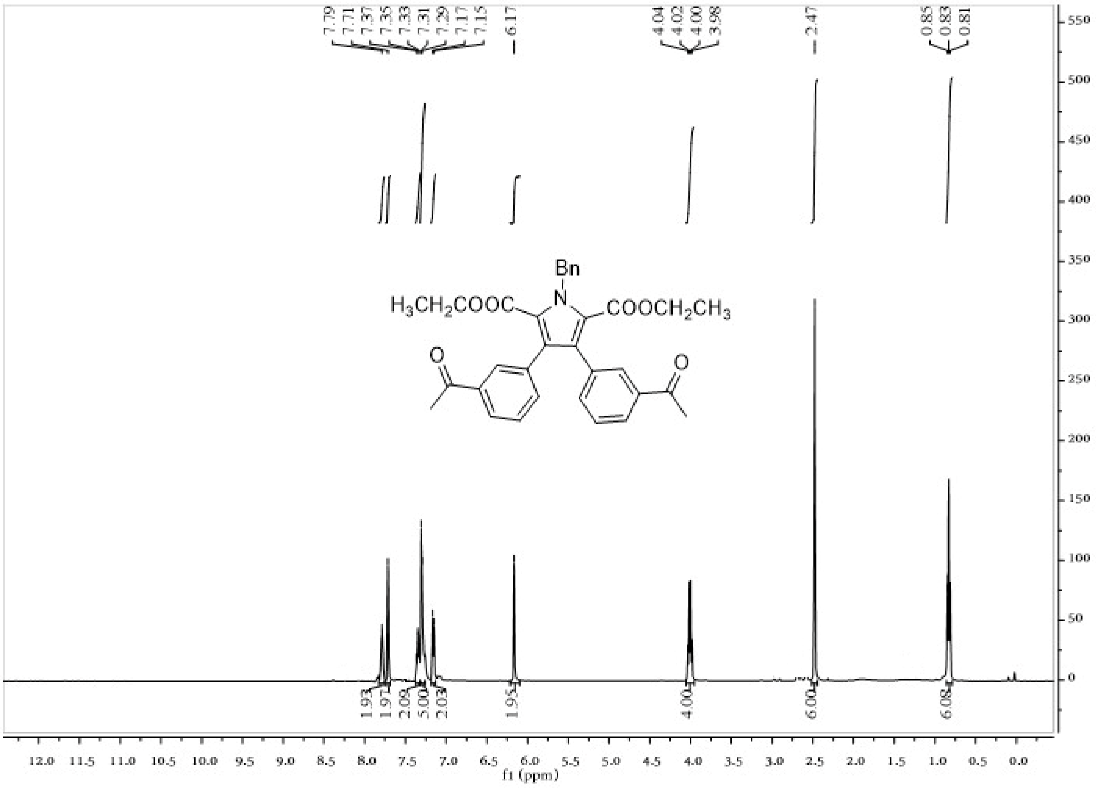

Figure S35. ^{1}H NMR spectrum of **6k**

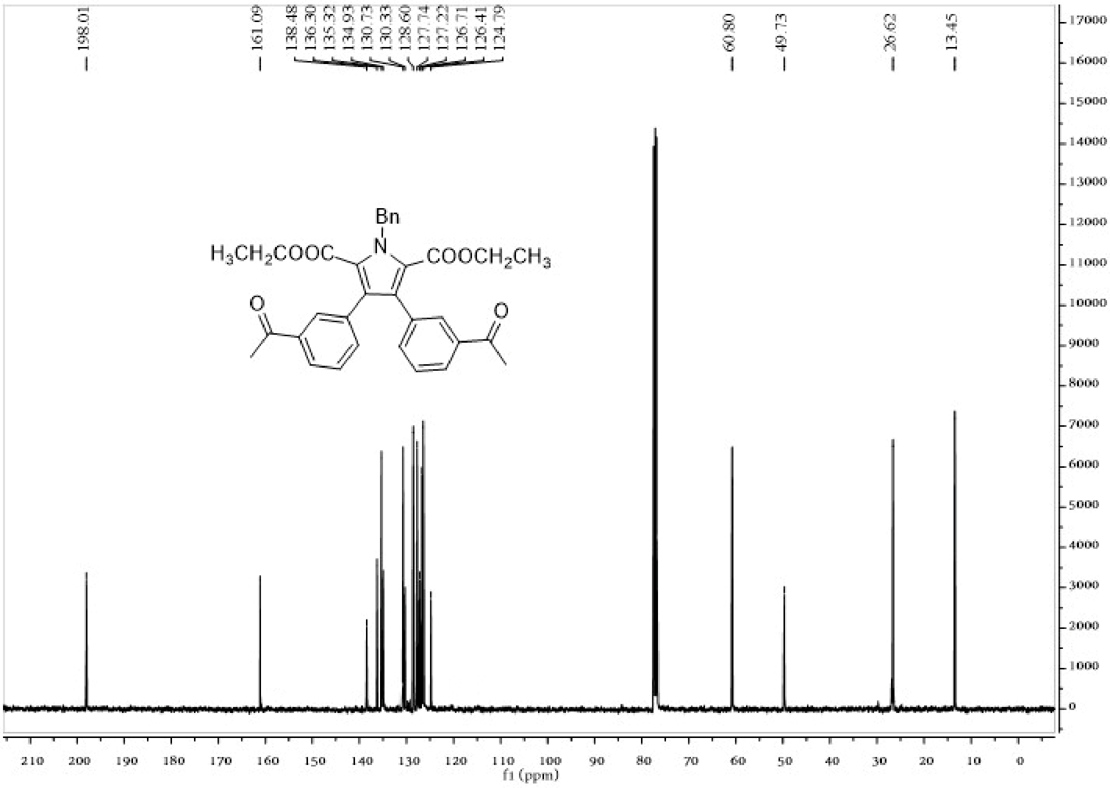

Figure S36. ^{13}C NMR spectrum of **6k**

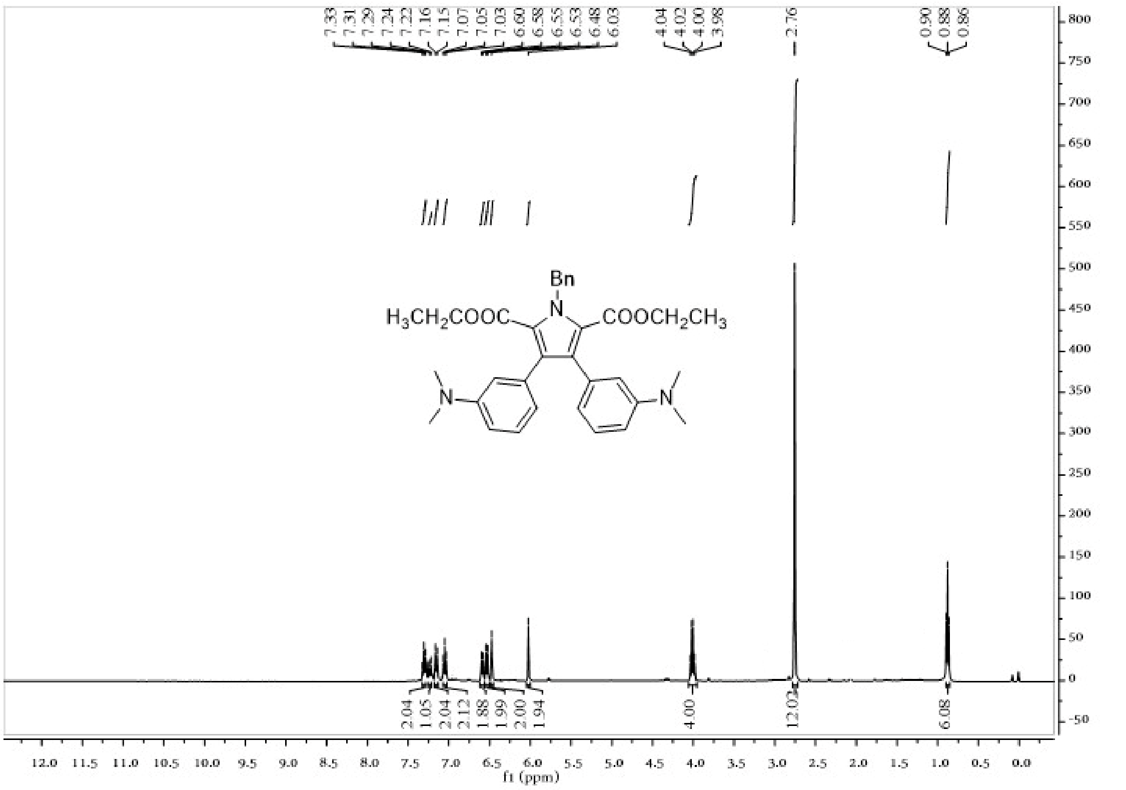

Figure S37. ^{1}H NMR spectrum of **6l**

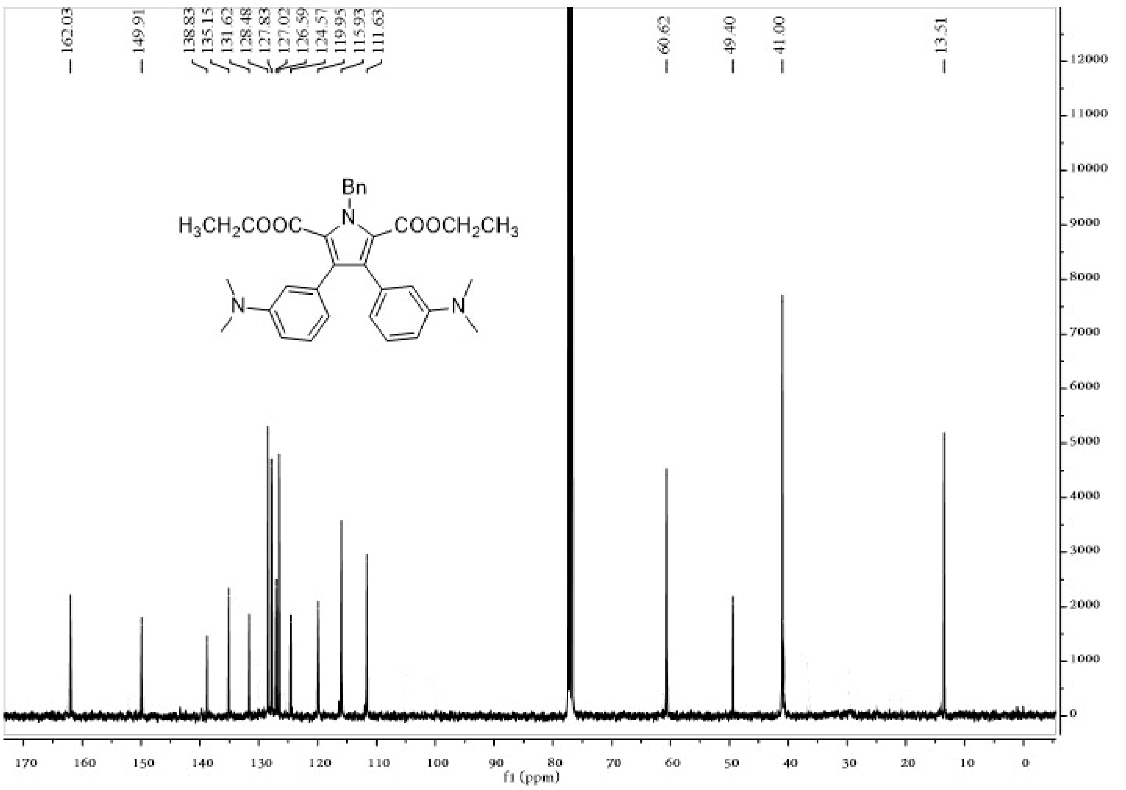

Figure S38. ^{13}C NMR spectrum of **6l**

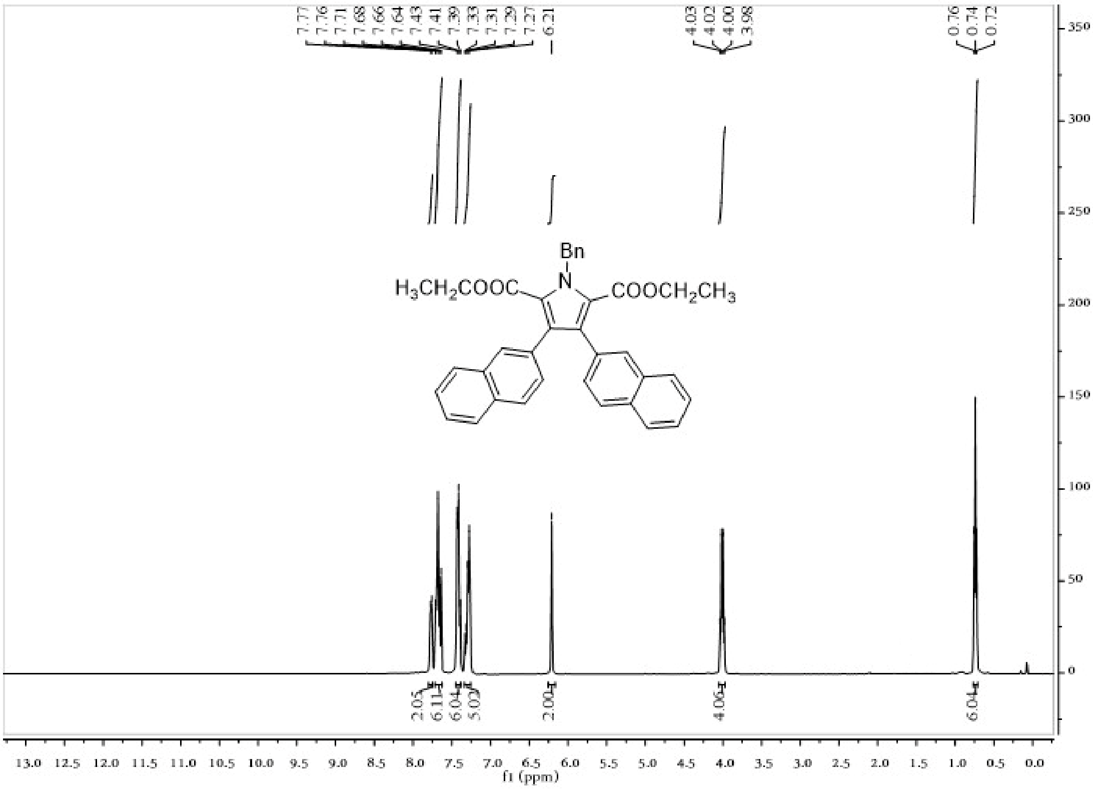

Figure S39. ^{1}H NMR spectrum of **6m**

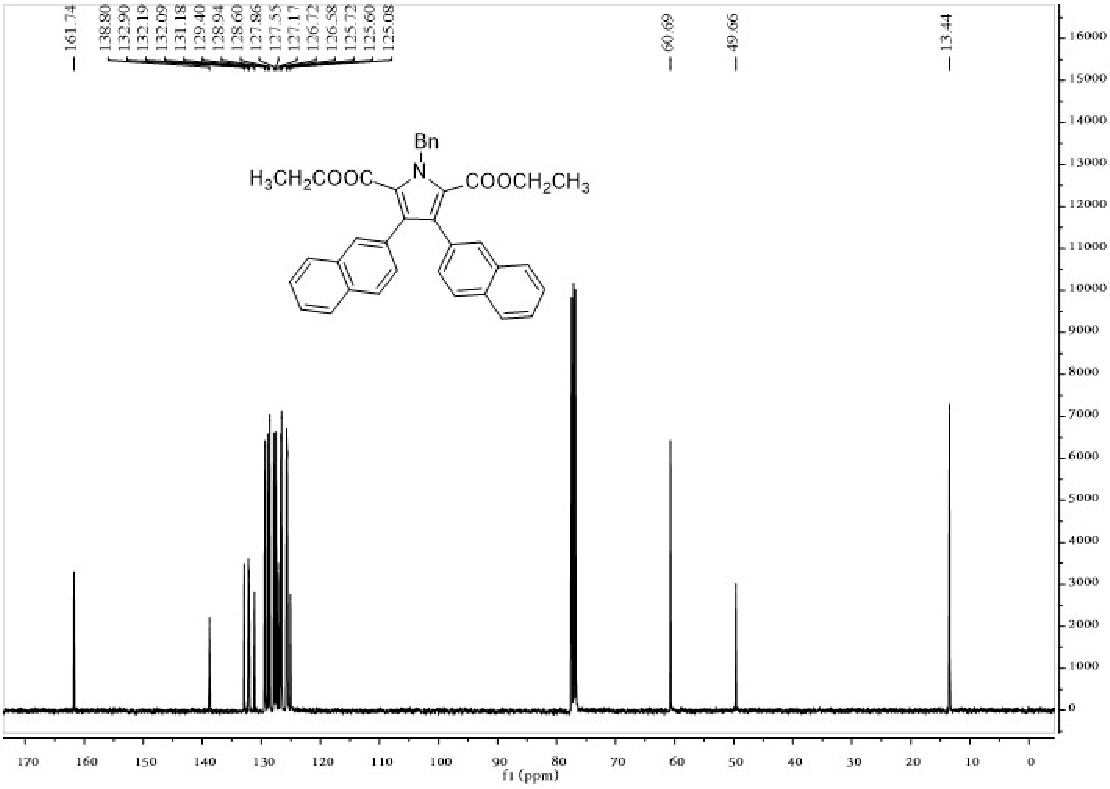

Figure S40. ^{13}C NMR spectrum of **6m**

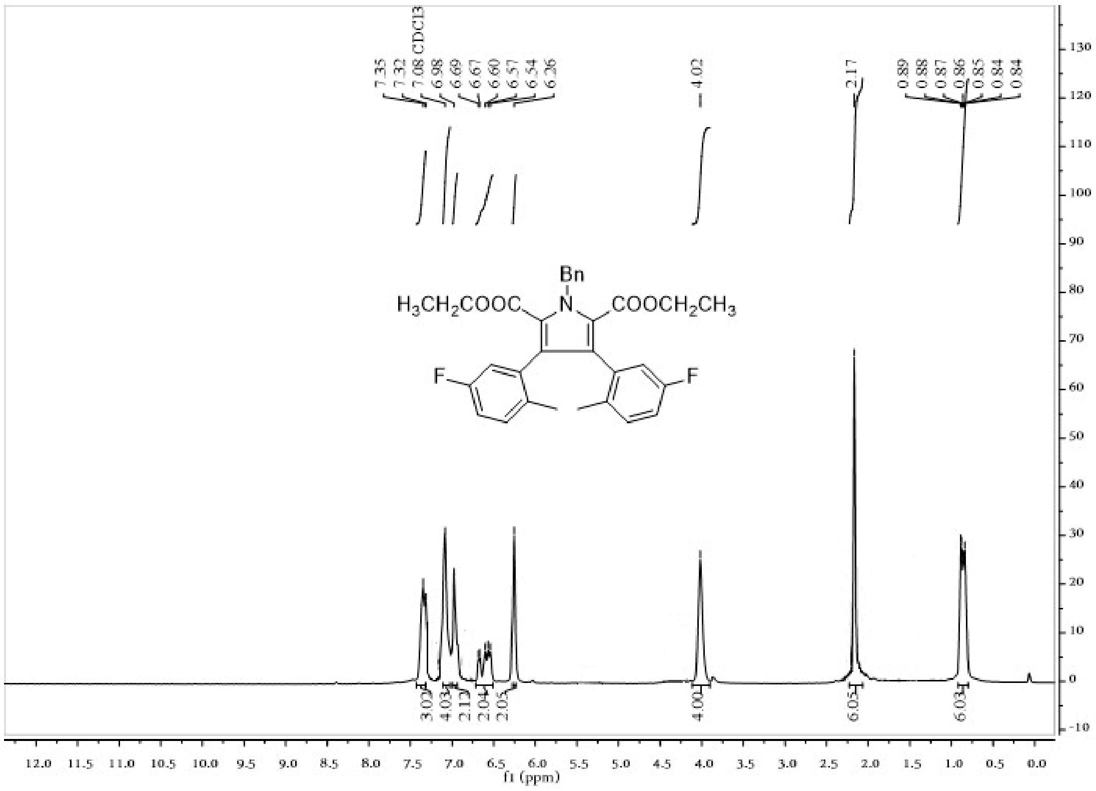

Figure S41. ^{1}H NMR spectrum of **6n**

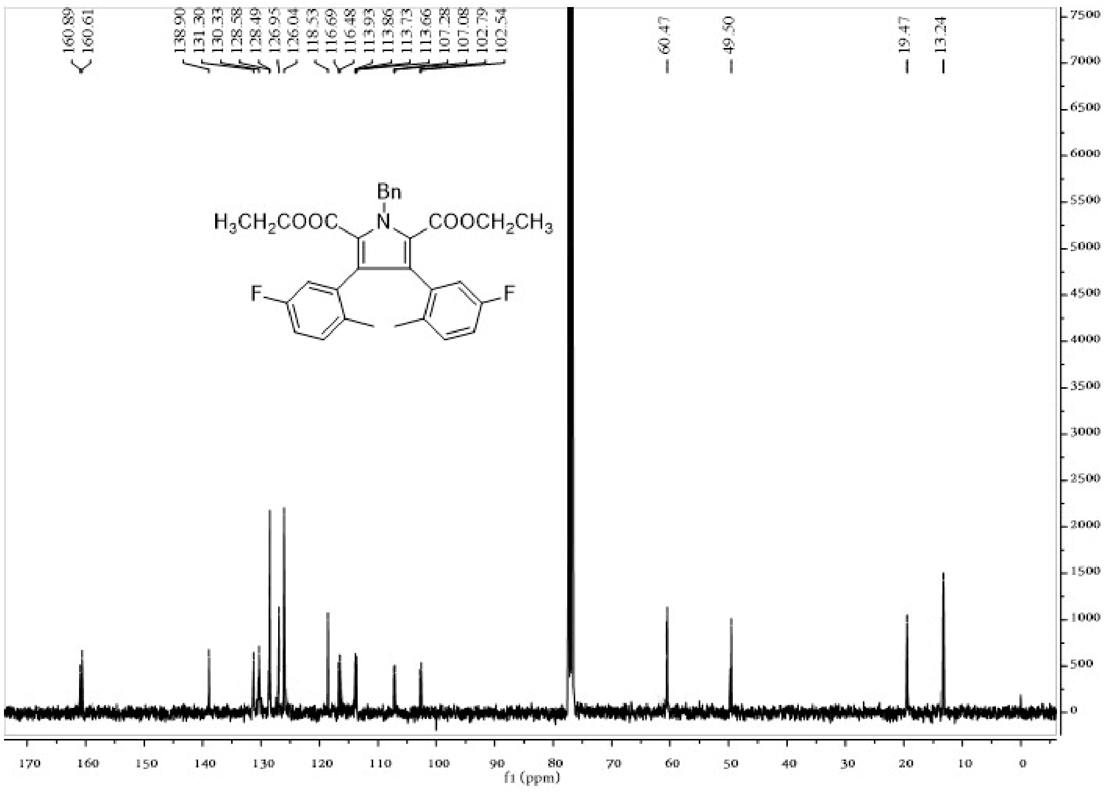

Figure S42. ^{13}C NMR spectrum of **6n**

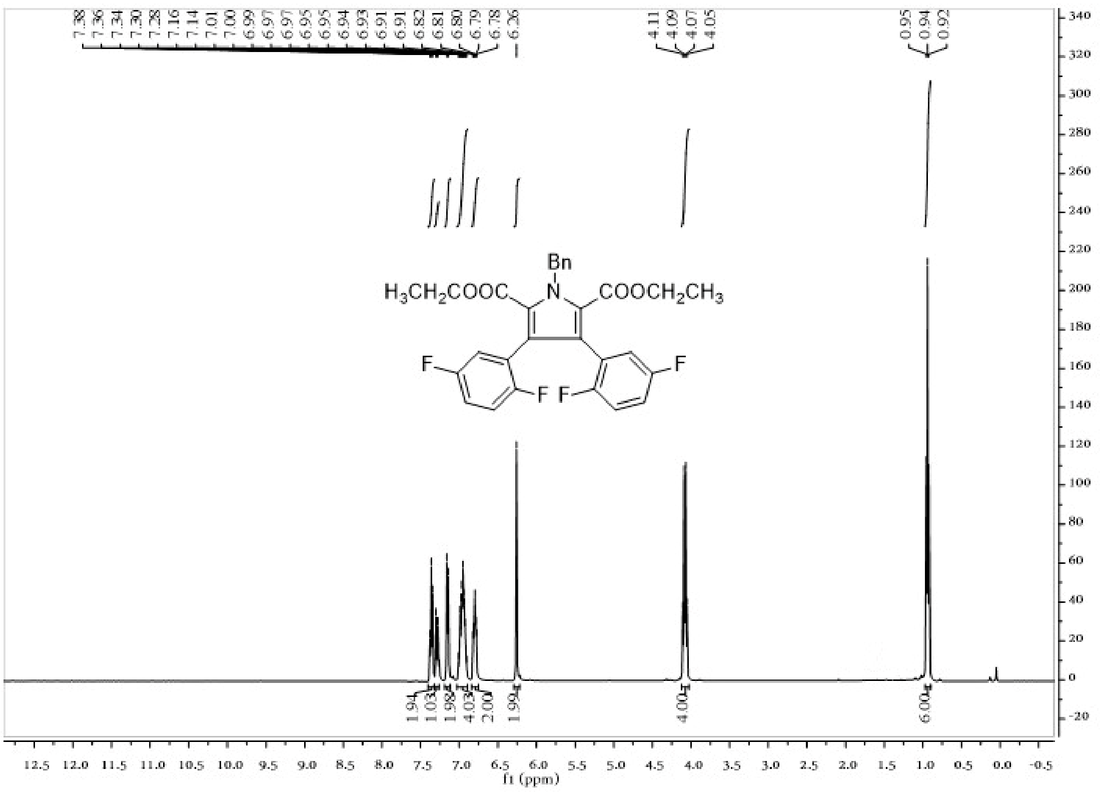

Figure S43. ^{1}H NMR spectrum of **6o**

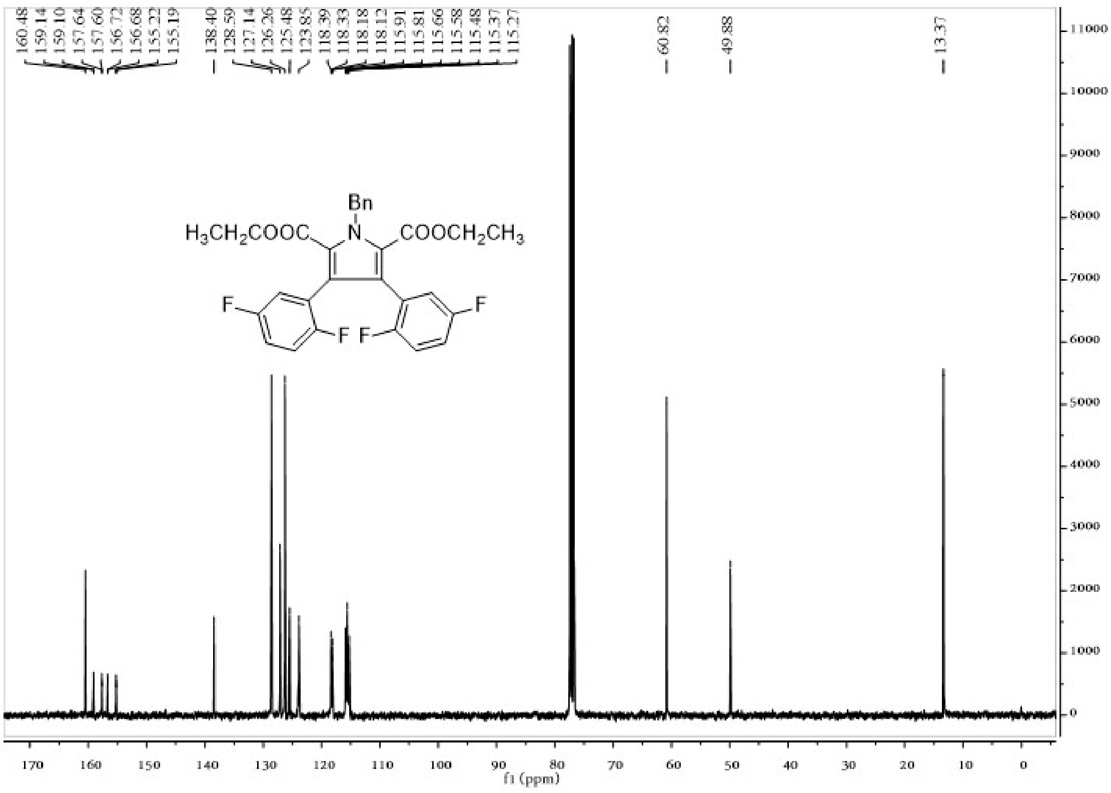

Figure S44. ^{13}C NMR spectrum of **6o**

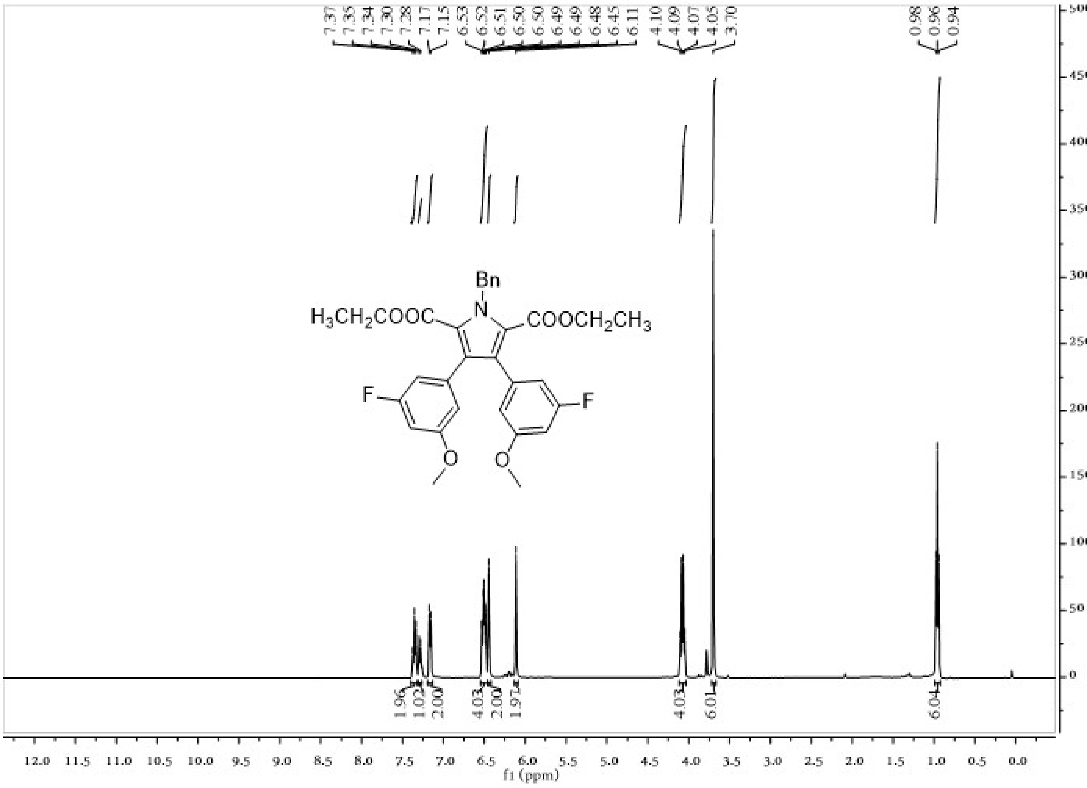

Figure S45. ^{1}H NMR spectrum of **6p**

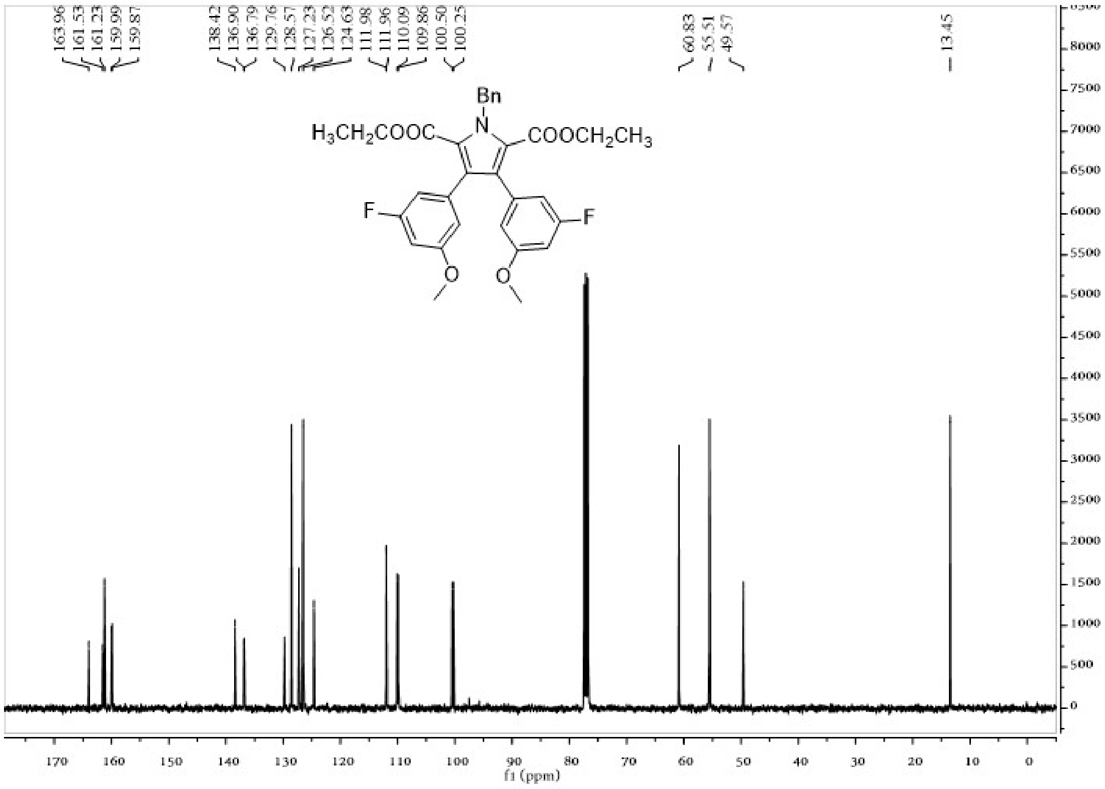

Figure S46. ^{13}C NMR spectrum of **6p**

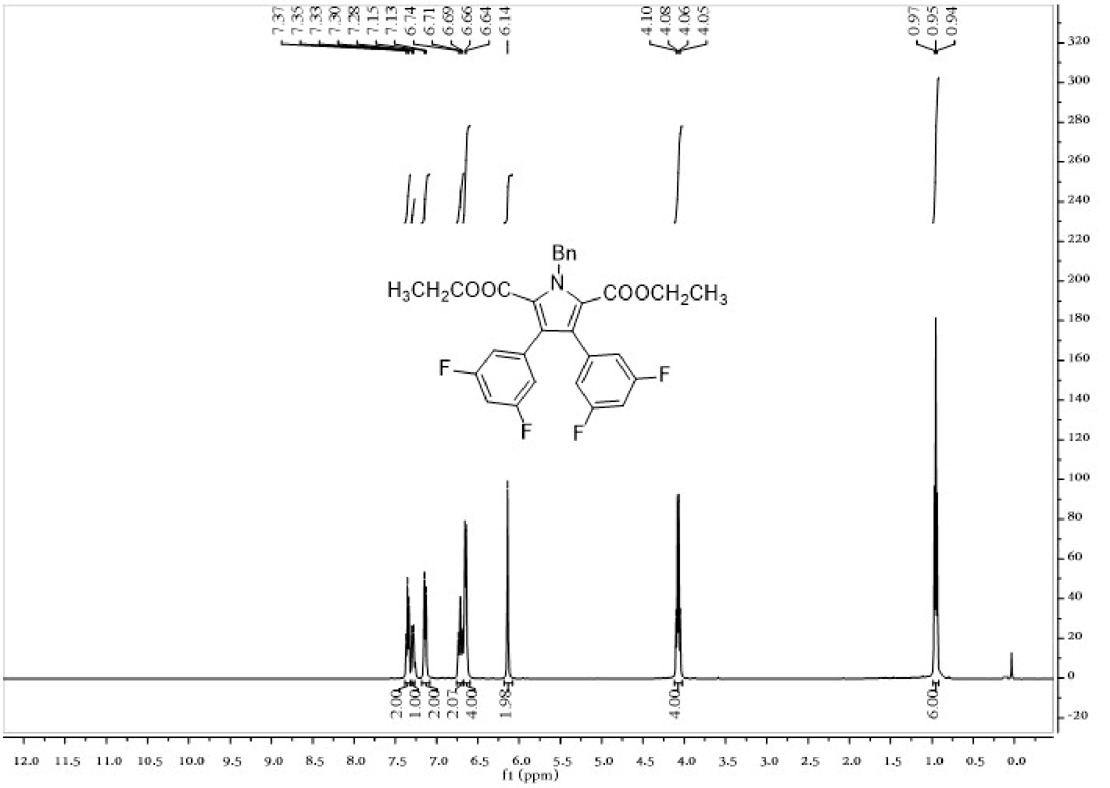

Figure S47. ^{1}H NMR spectrum of **6q**

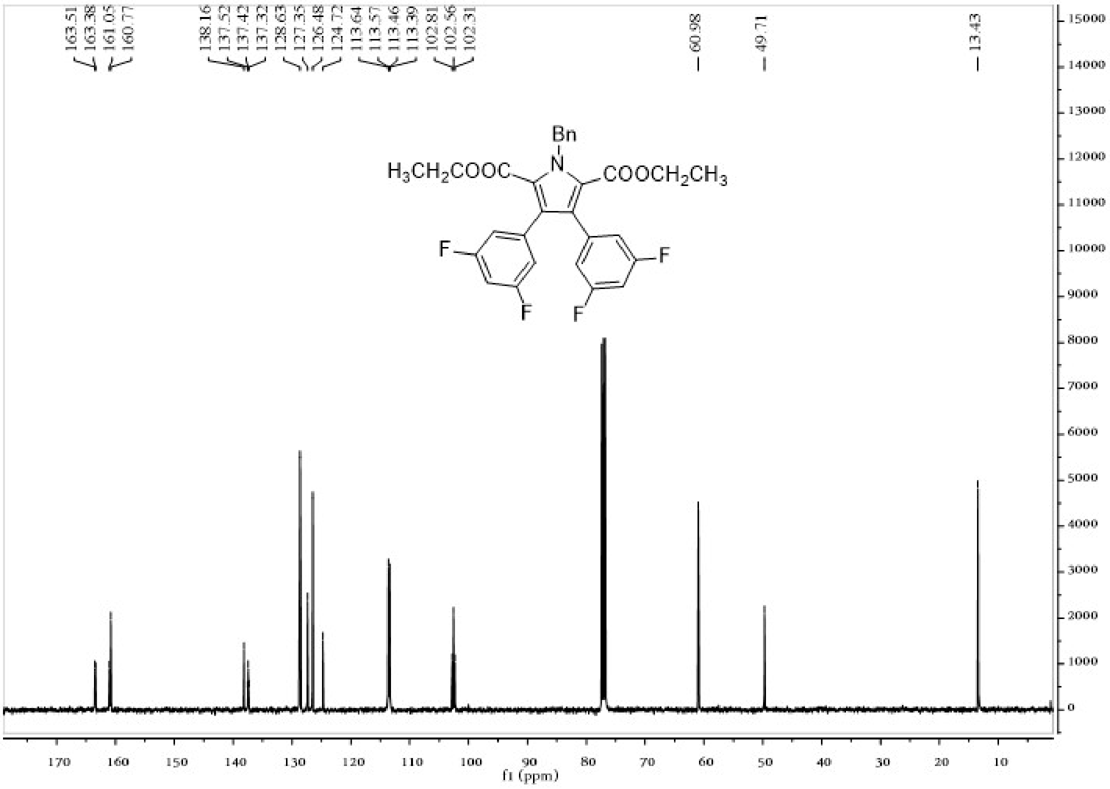

Figure S48. ^{13}C NMR spectrum of **6q**

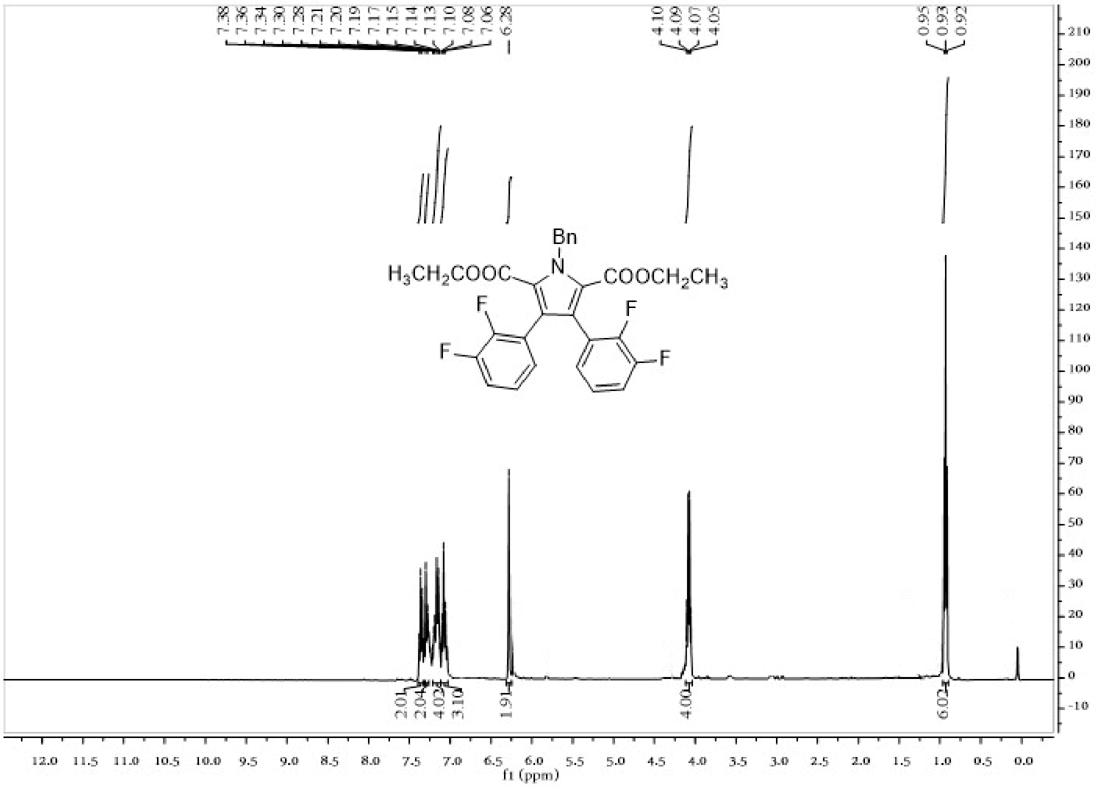

Figure S49. ^{1}H NMR spectrum of **6r**

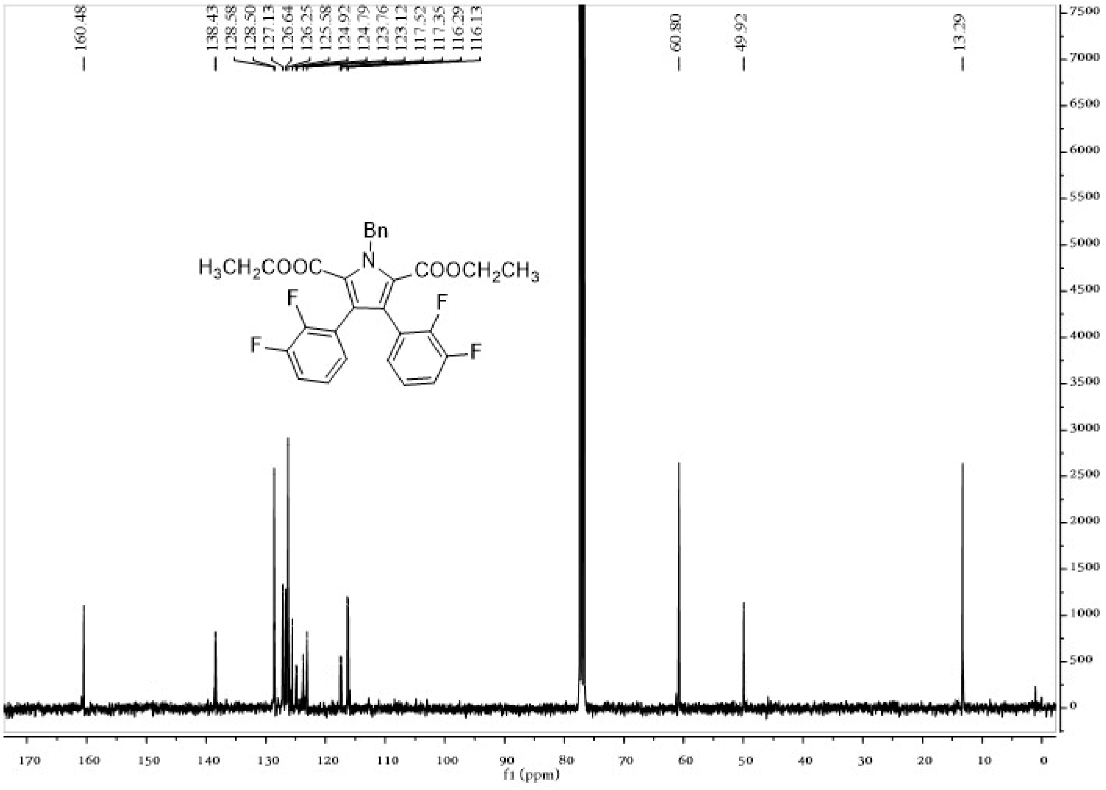

Figure S50. ^{13}C NMR spectrum of **6r**

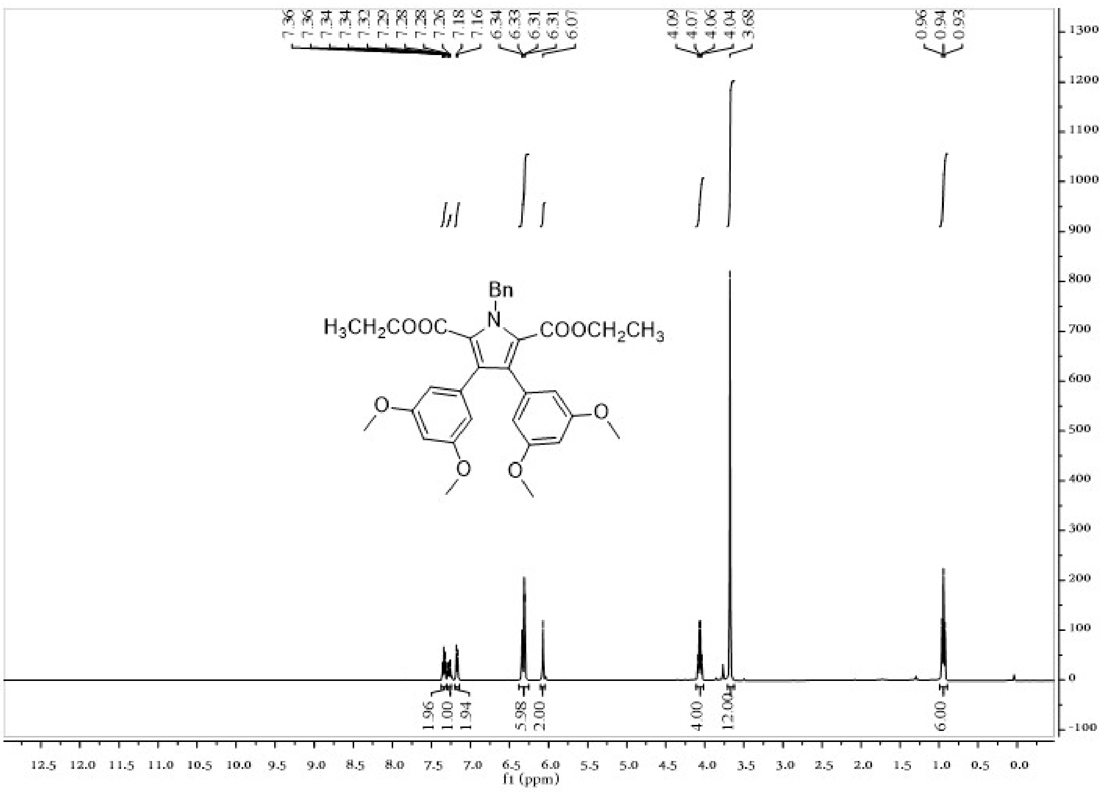

Figure S51. ^{1}H NMR spectrum of **6s**

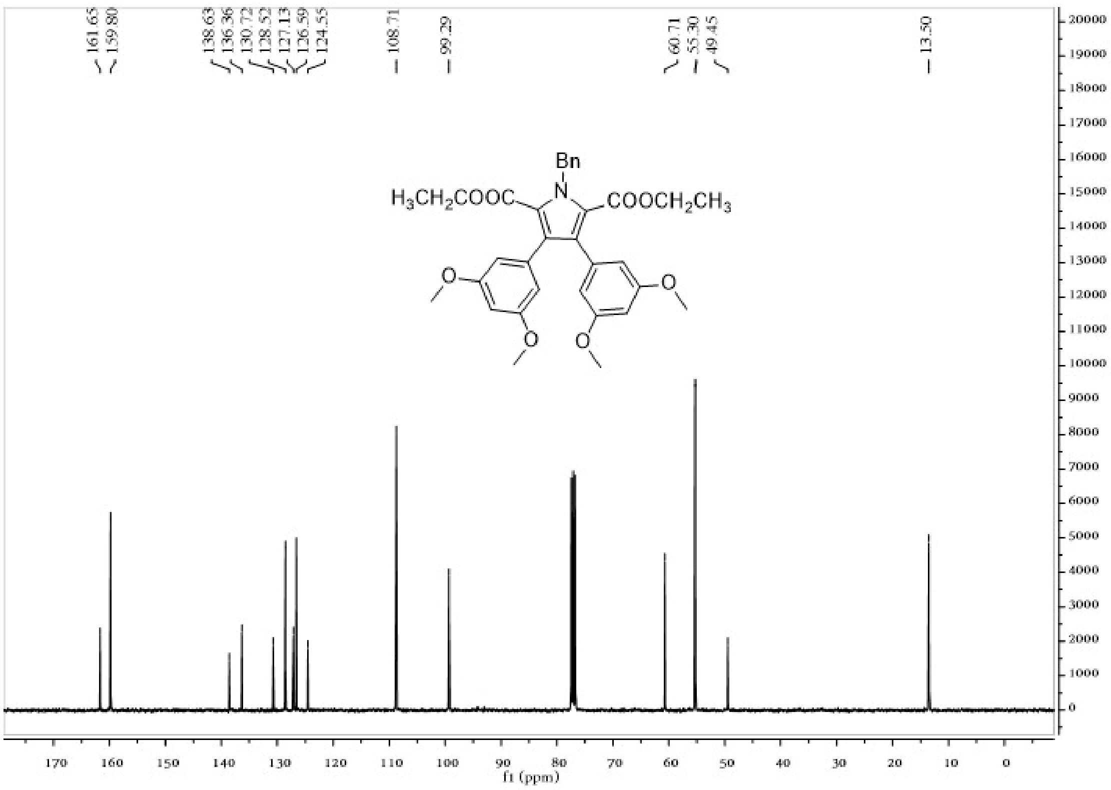

Figure S52. ^{13}C NMR spectrum of **6s**

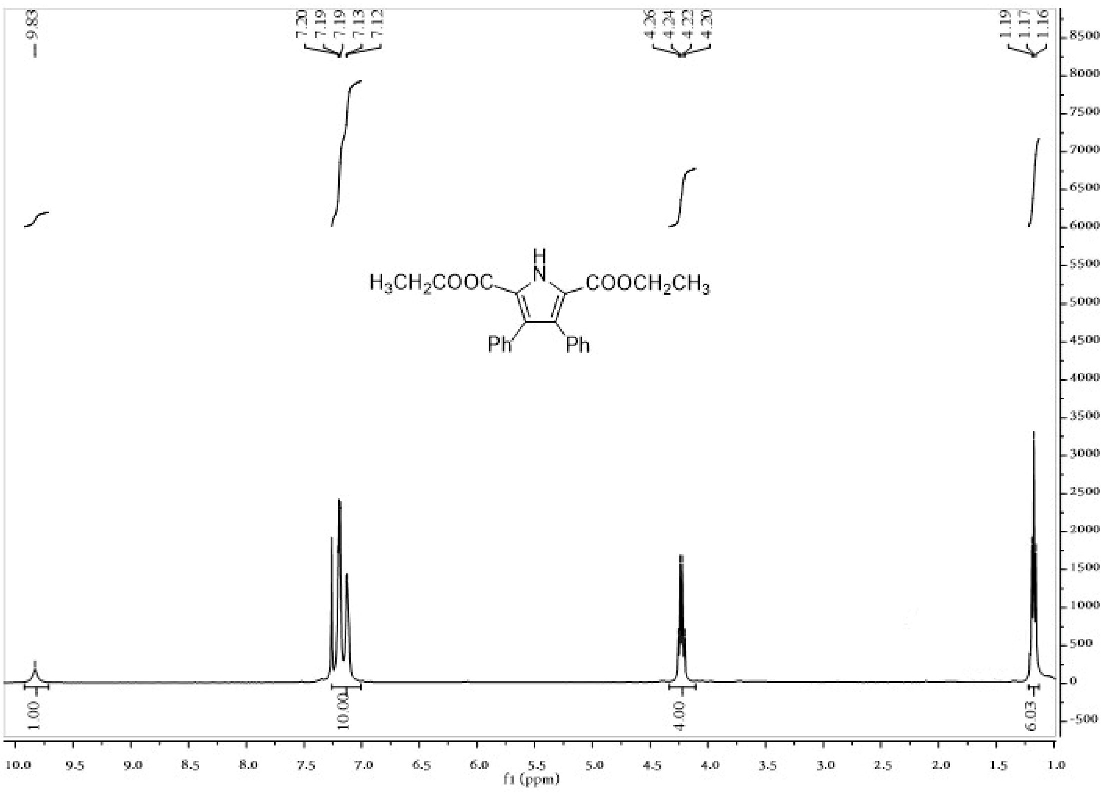

Figure S53. ^{1}H NMR spectrum of **7a**

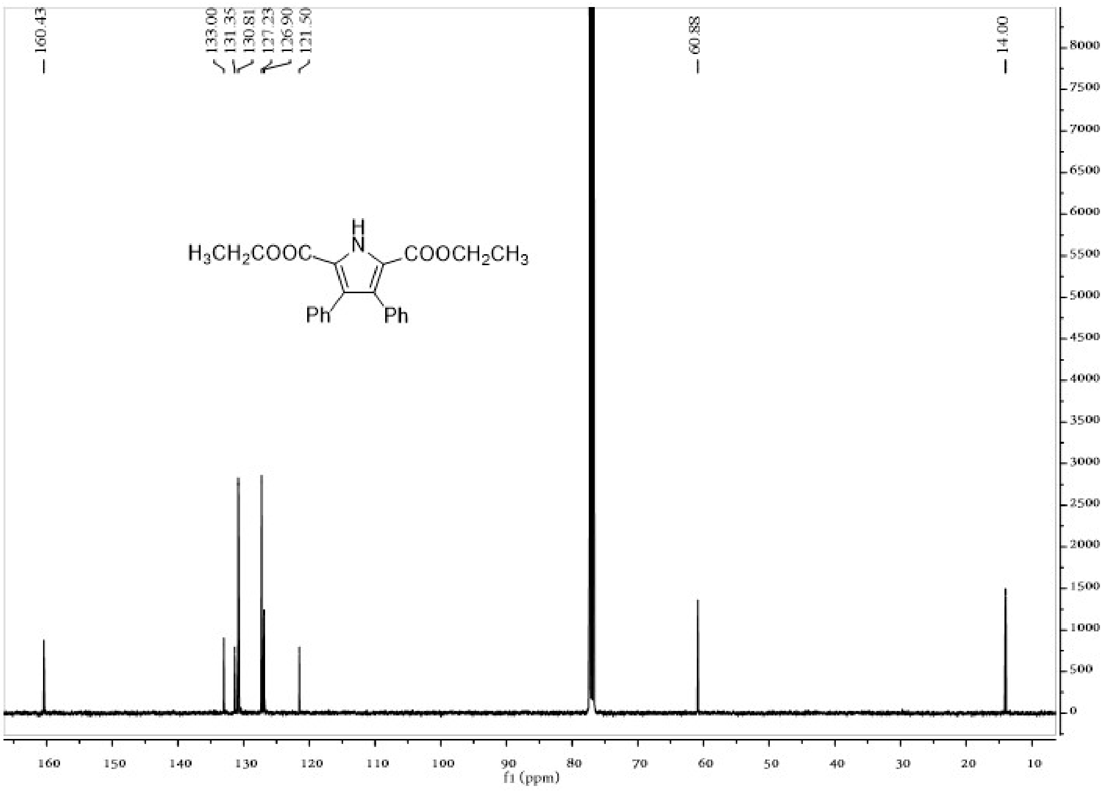

Figure S54. ^{13}C NMR spectrum of **7a**

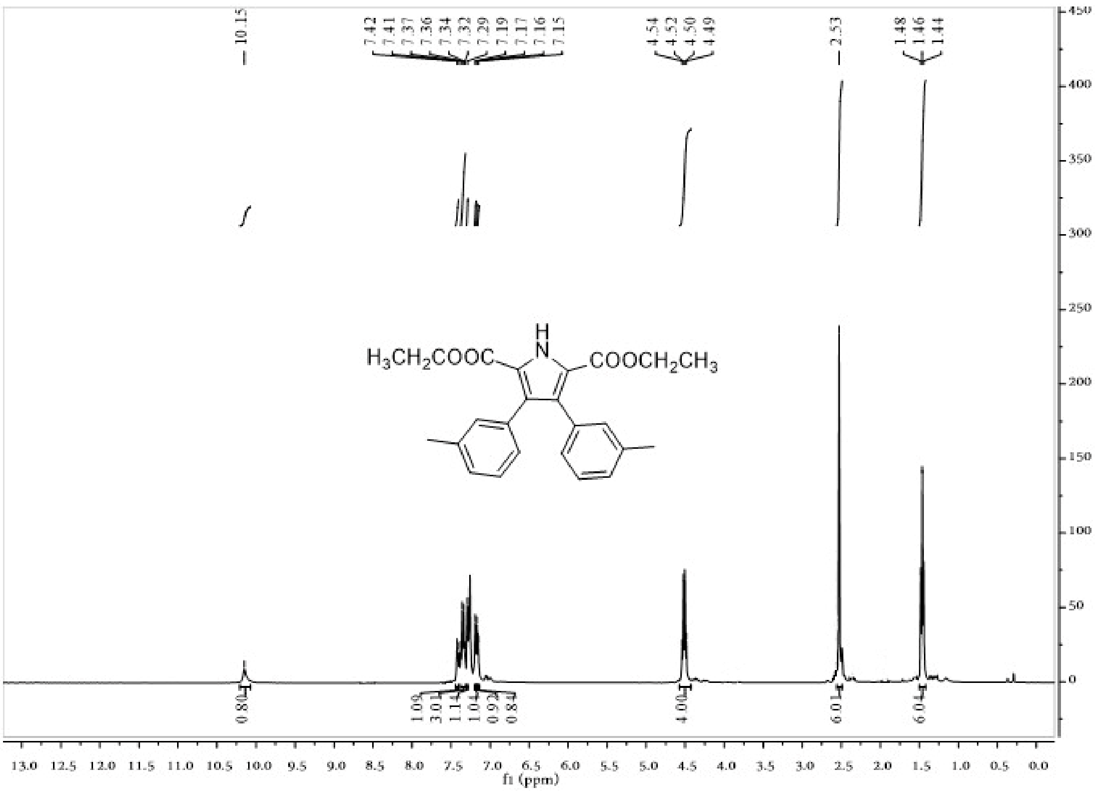

Figure S55. ^{1}H NMR spectrum of **7b**

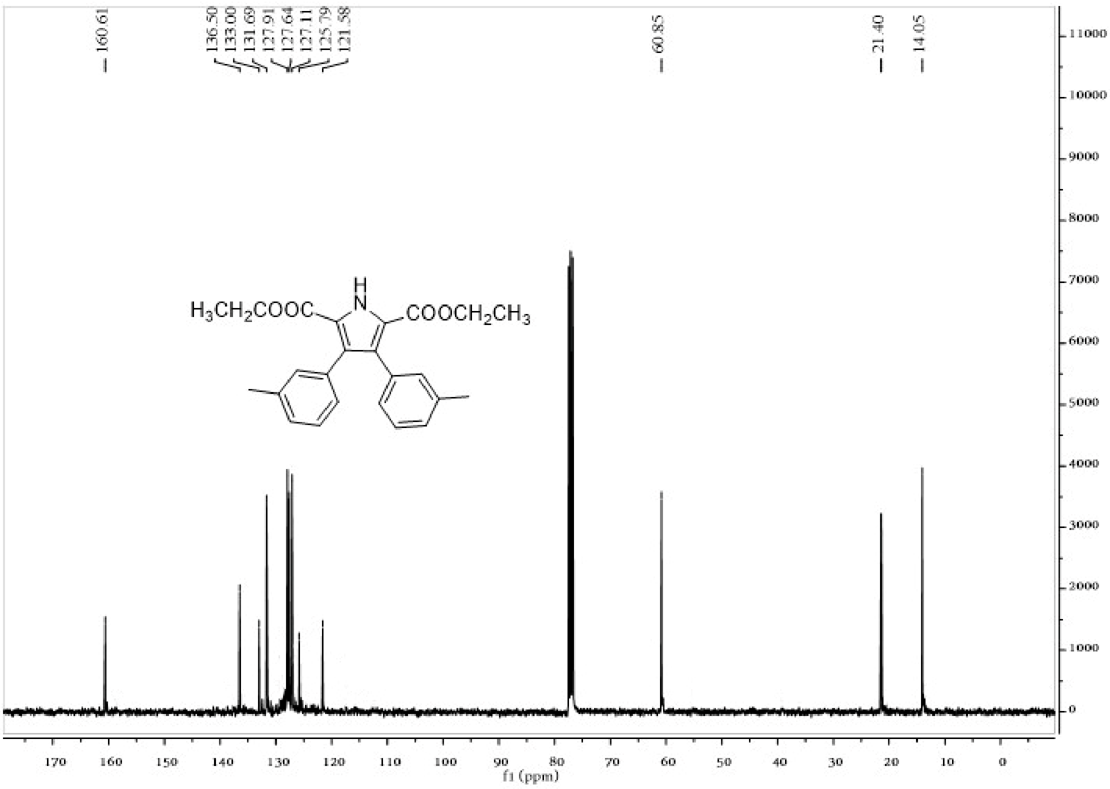

Figure S56. ^{13}C NMR spectrum of **7b**

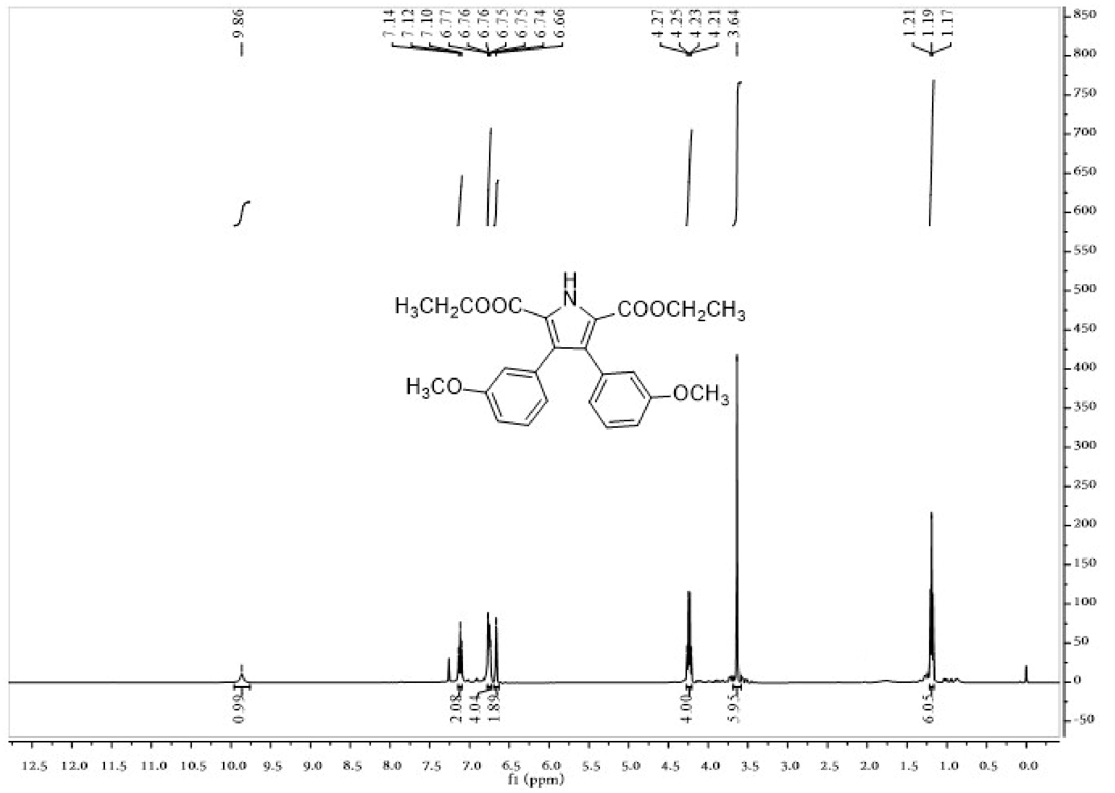

Figure S57. ^{1}H NMR spectrum of **7c**

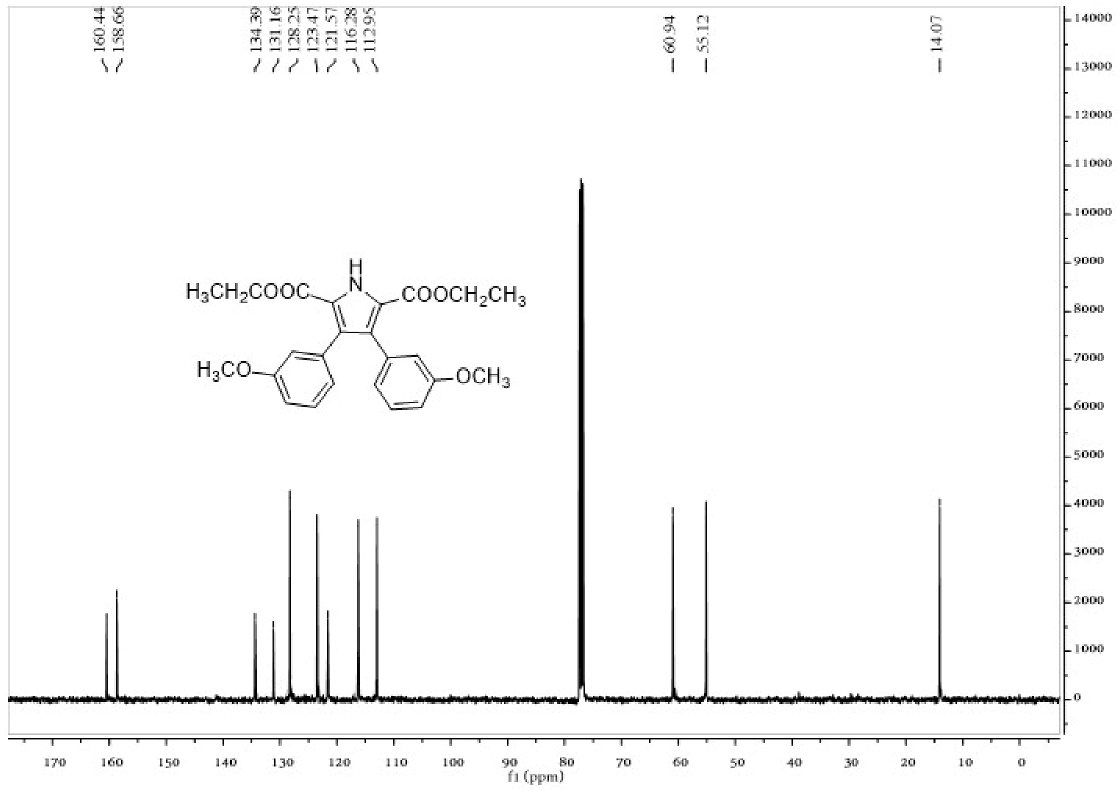

Figure S58. ^{13}C NMR spectrum of **7c**

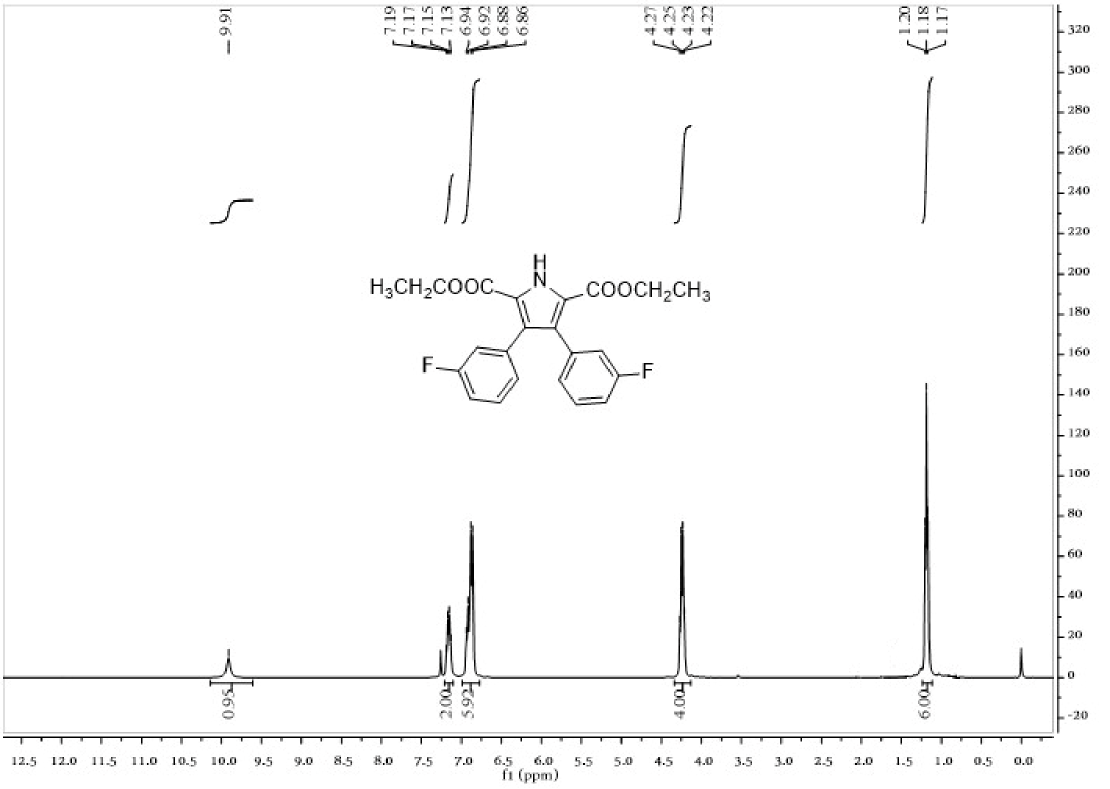

Figure S59. ^{1}H NMR spectrum of **7d**

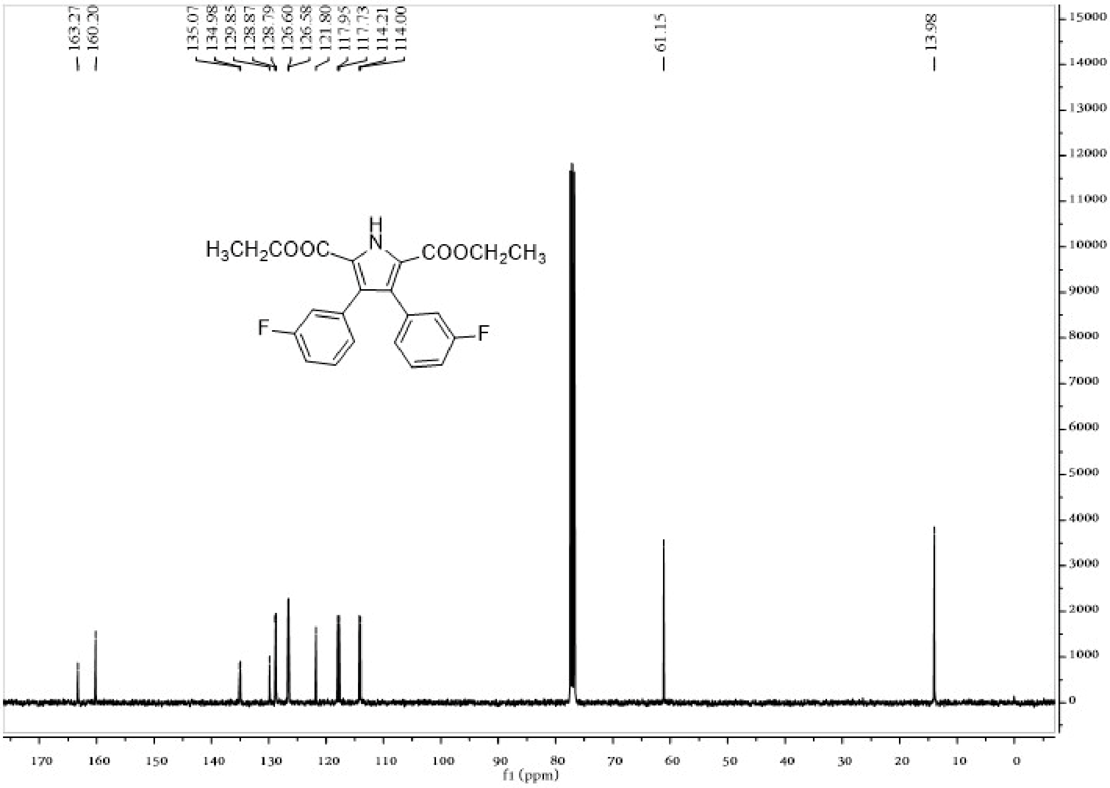

Figure S60. ^{13}C NMR spectrum of **7d**

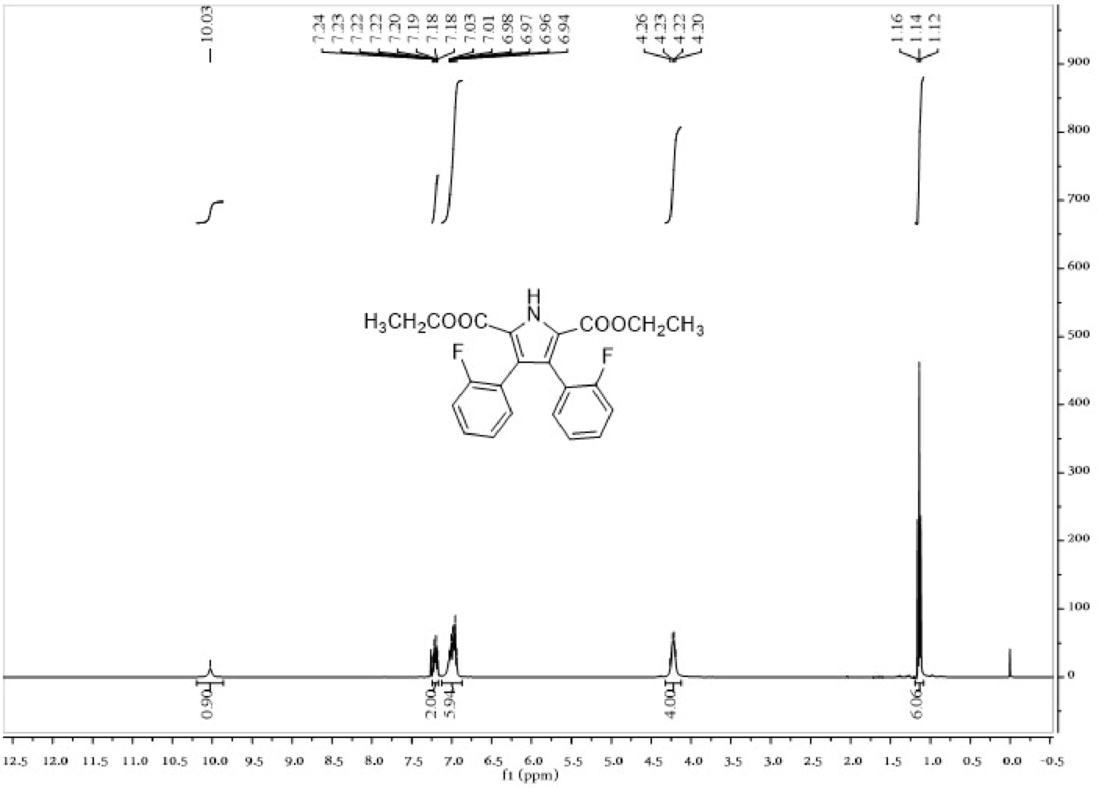

Figure S61. ^{1}H NMR spectrum of **7e**

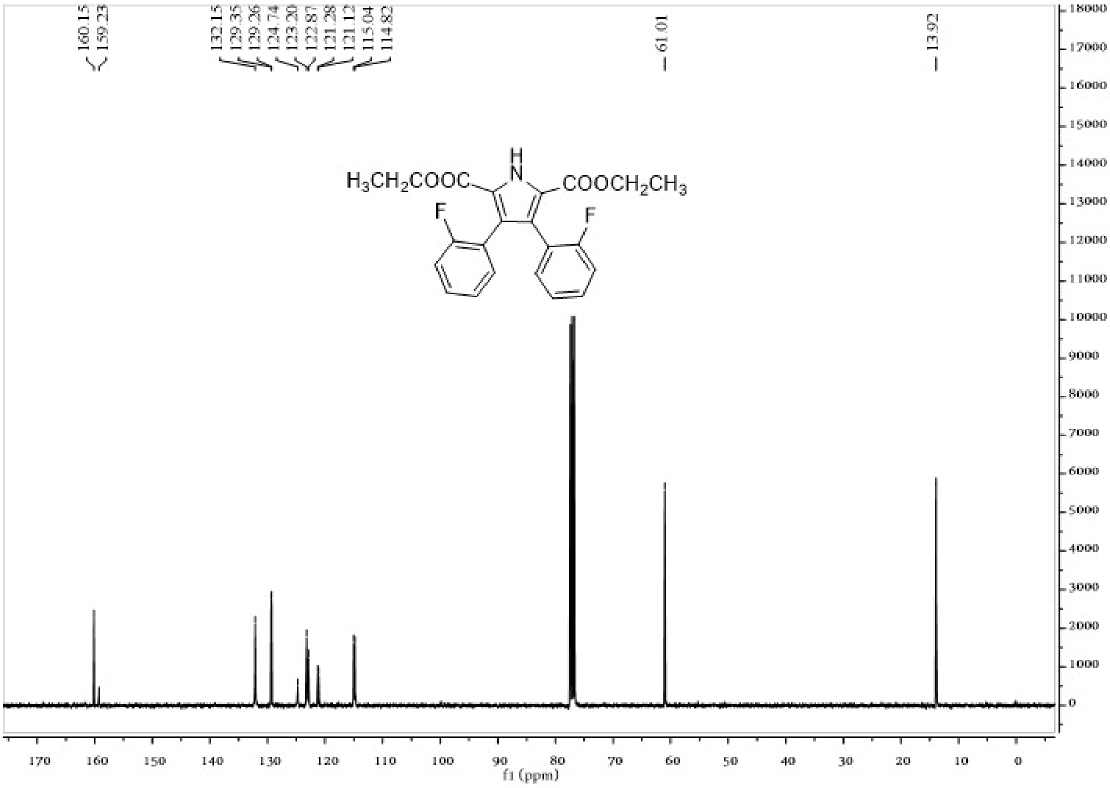

Figure S62. ^{13}C NMR spectrum of **7e**

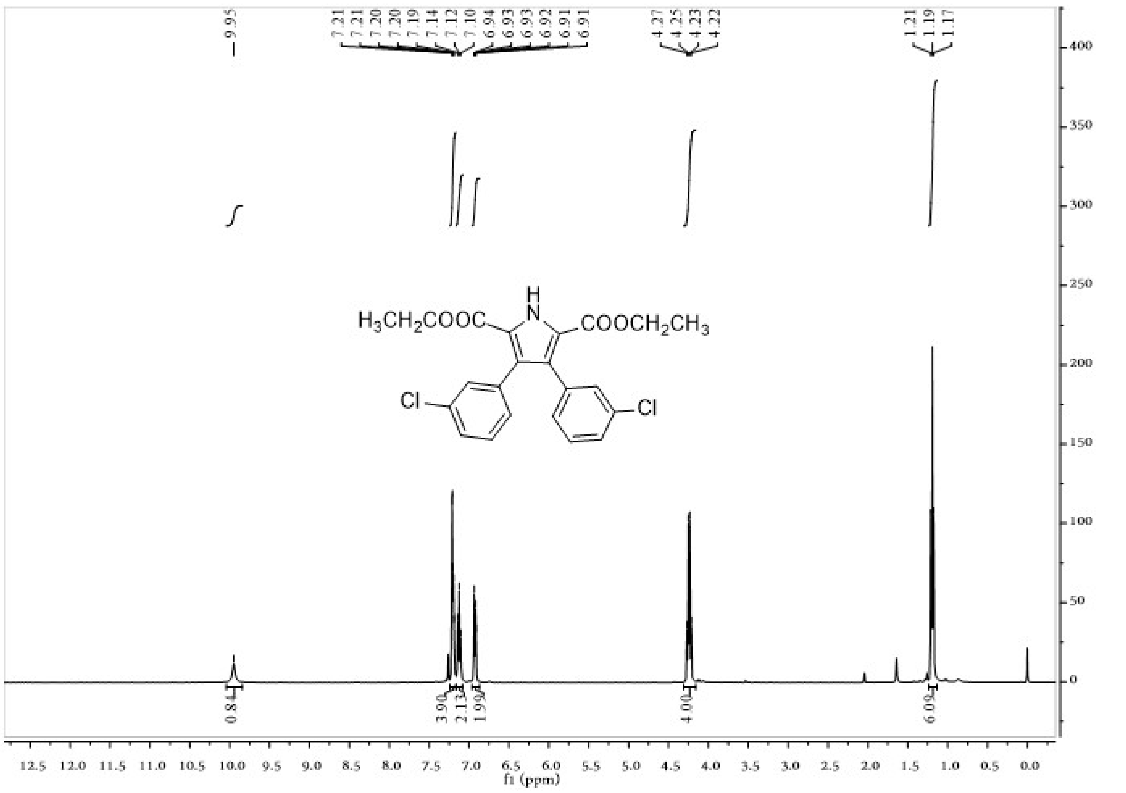

Figure S63. ^{1}H NMR spectrum of **7f**

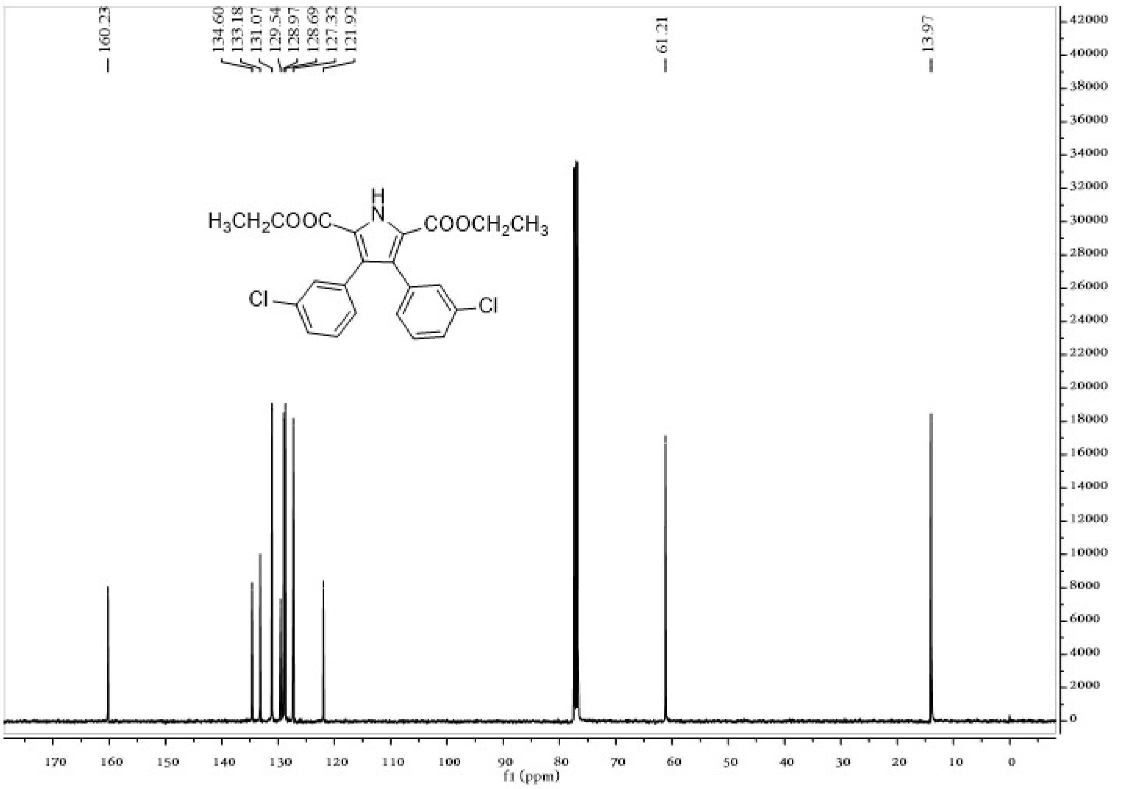

Figure S64. ^{13}C NMR spectrum of **7f**

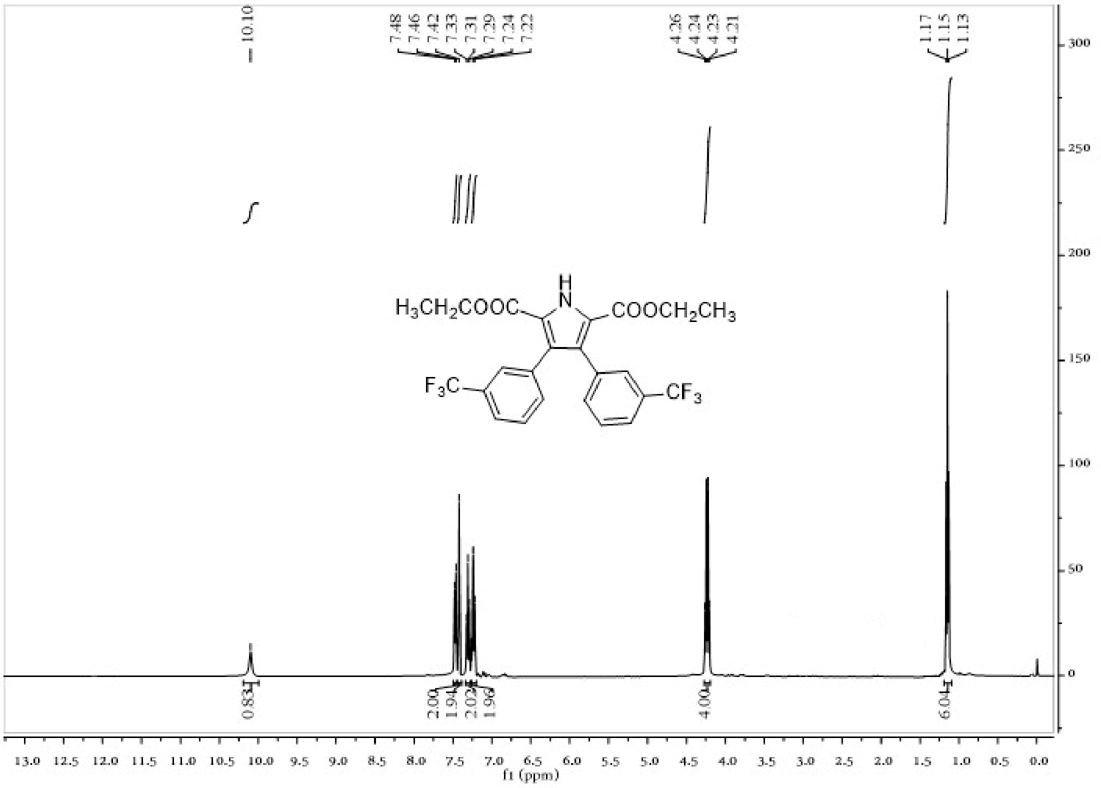

Figure S65. ^{1}H NMR spectrum of **7g**

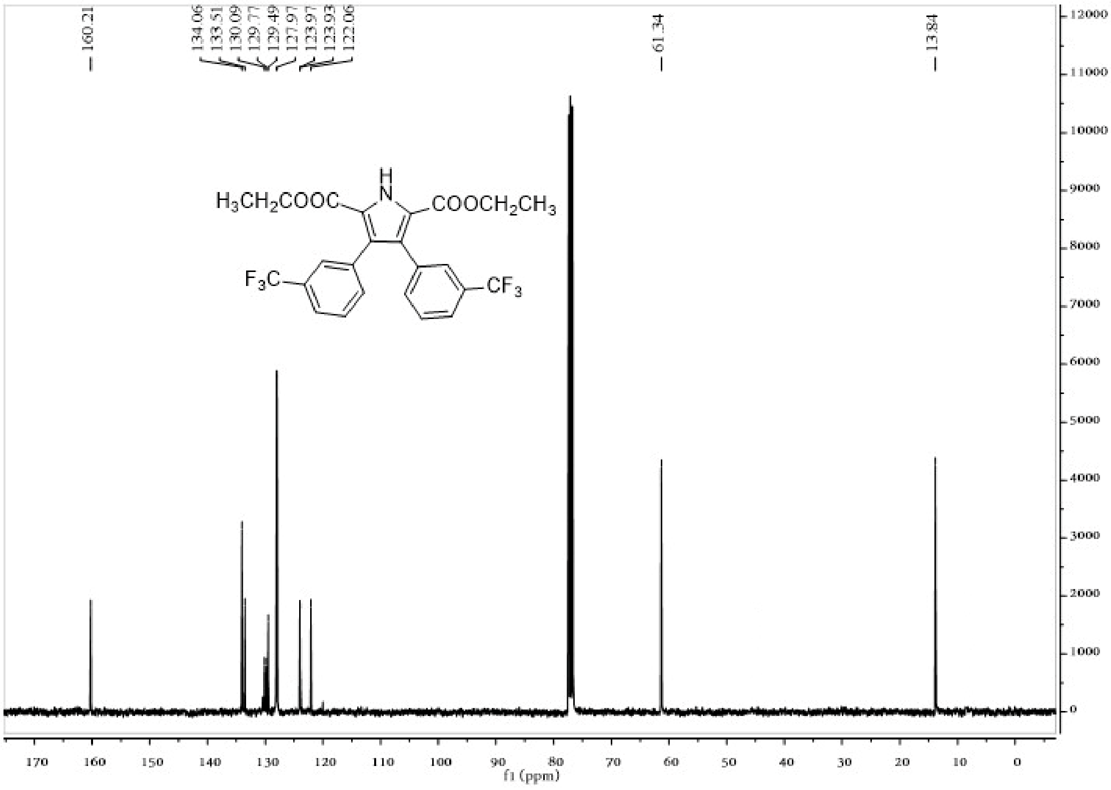

Figure S66. ^{13}C NMR spectrum of **7g**

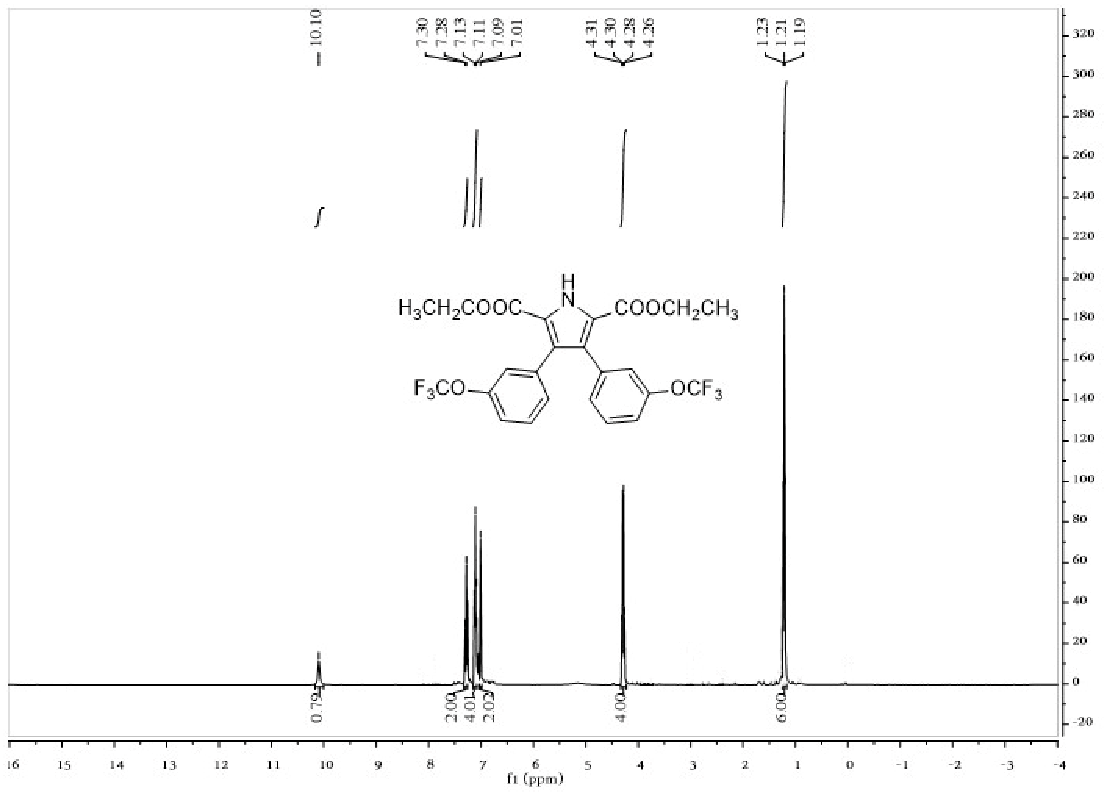

Figure S67. ^{1}H NMR spectrum of **7h**

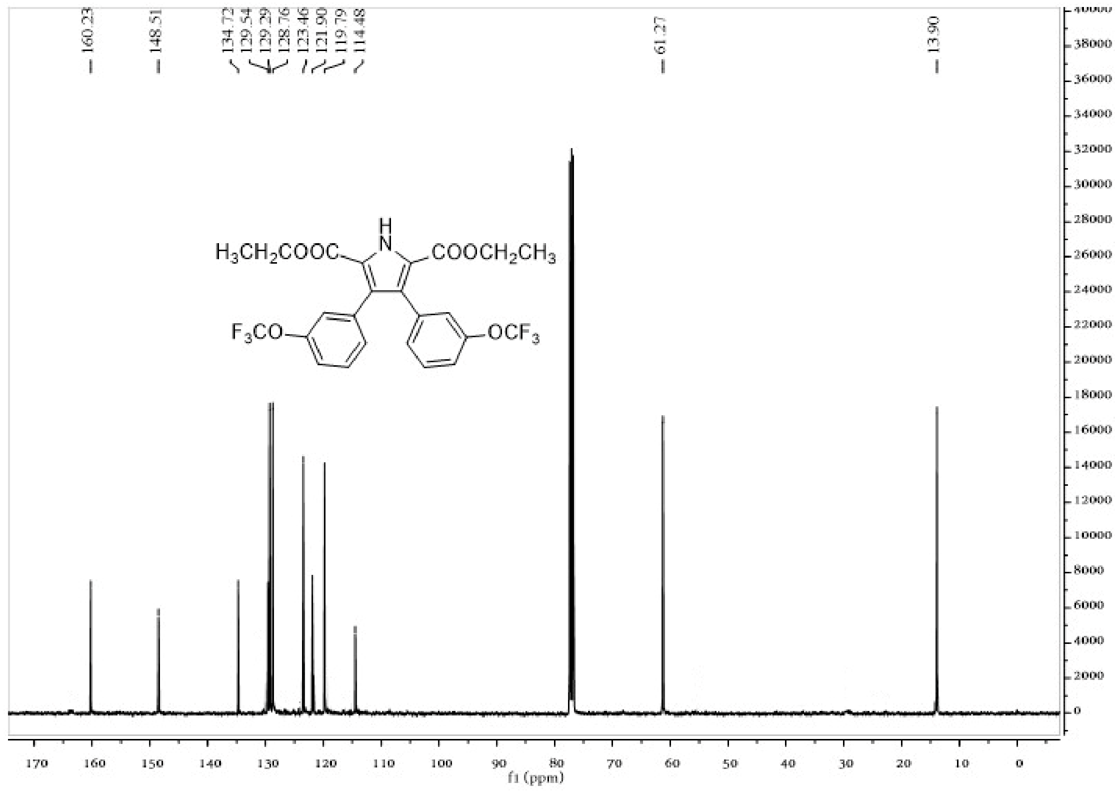

Figure S68. ^{13}C NMR spectrum of **7h**

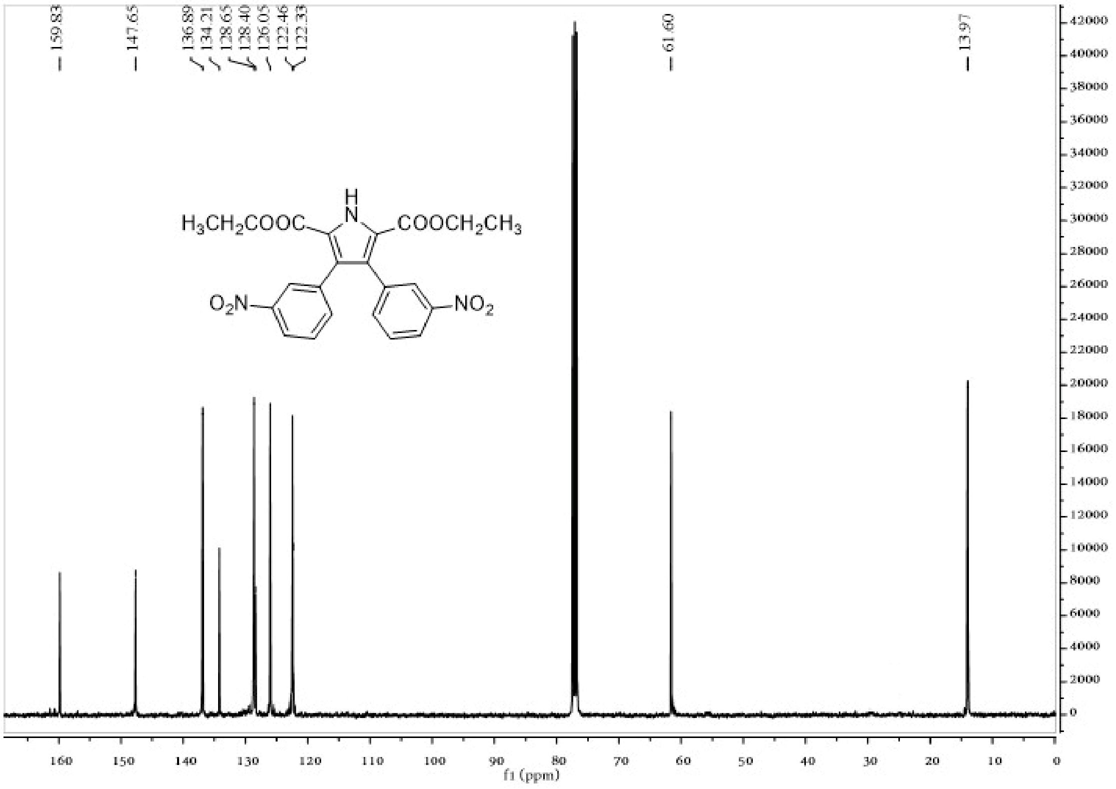

Figure S69. ¹H NMR spectrum of **7i**

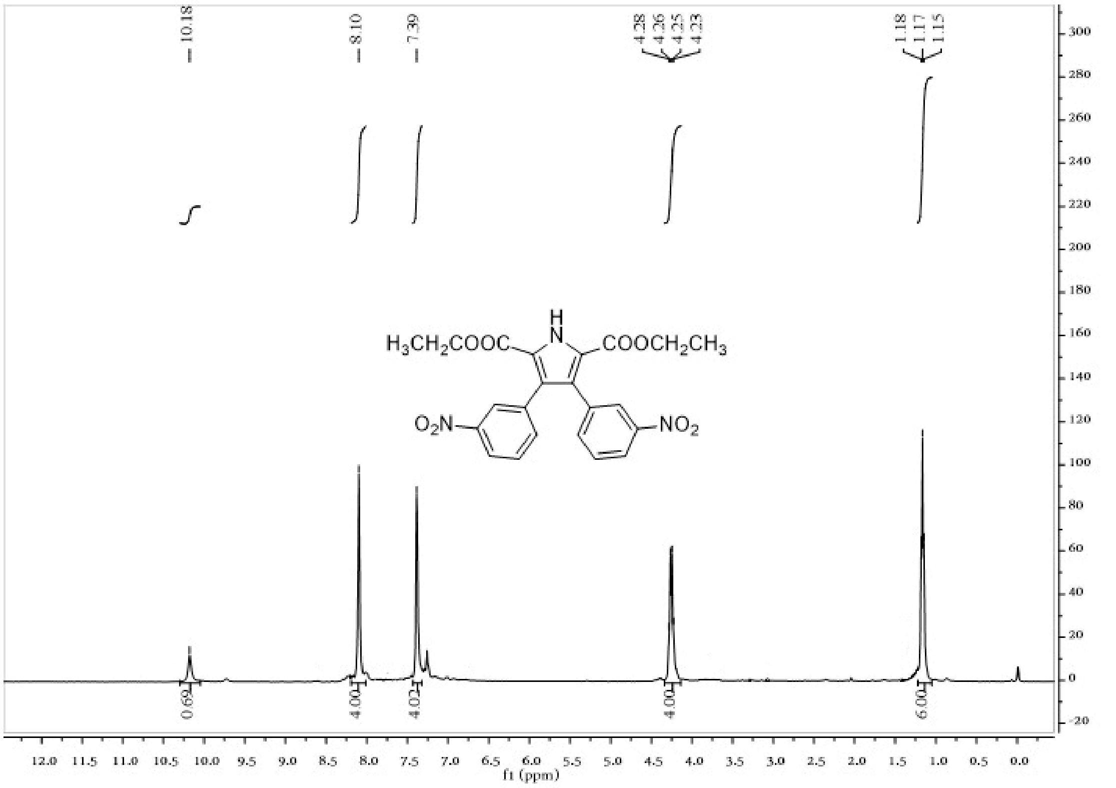

Figure S70. ¹³C NMR spectrum of **7i**

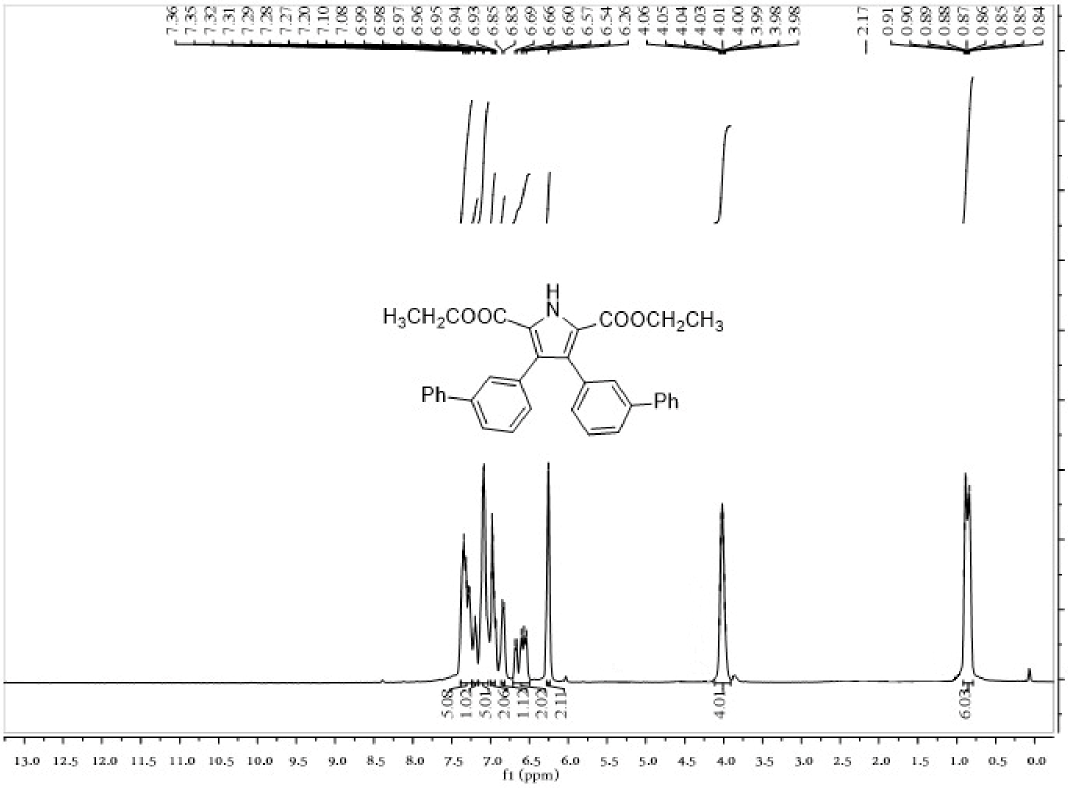

Figure S71. ^{1}H NMR spectrum of **7j**

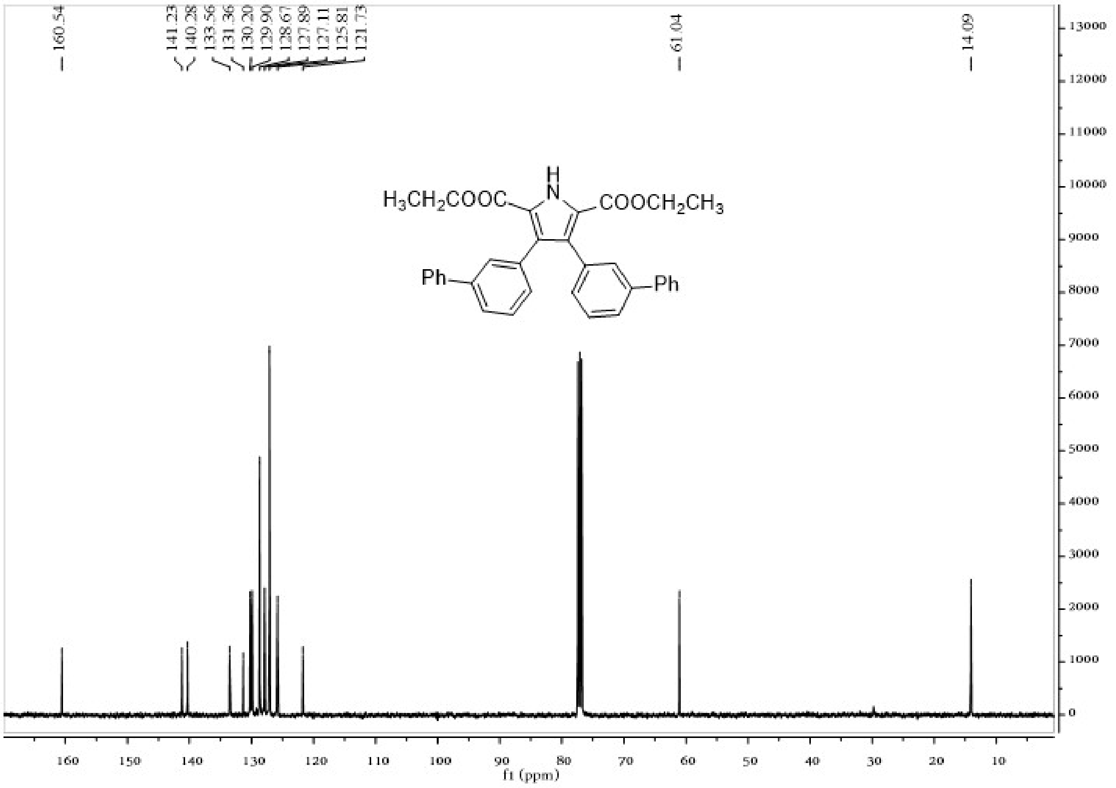

Figure S72. ^{13}C NMR spectrum of **7j**

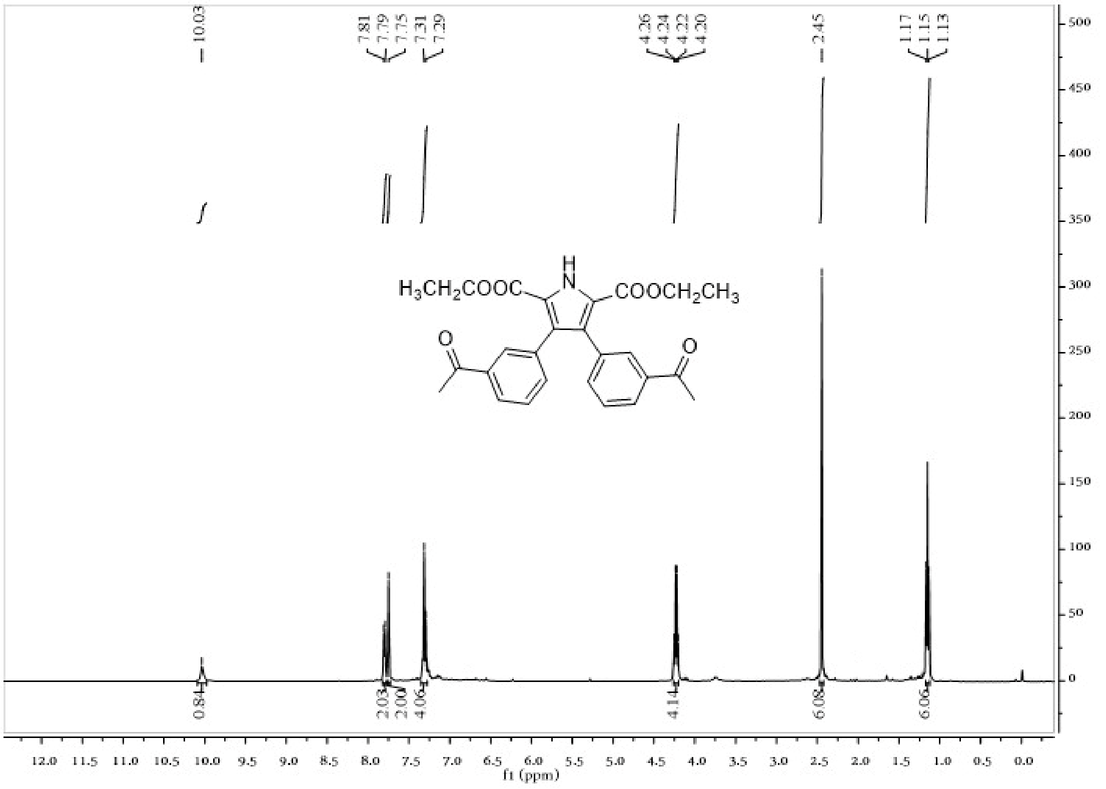

Figure S73. ^{1}H NMR spectrum of **7k**

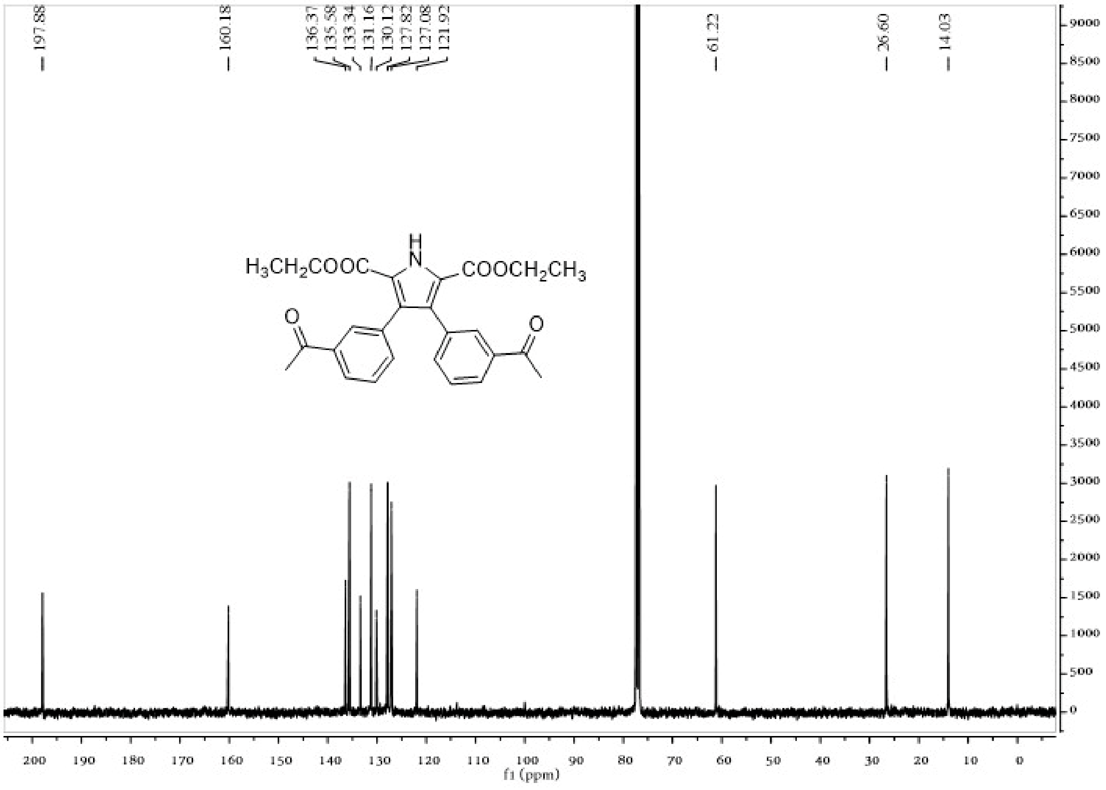

Figure S74. ^{13}C NMR spectrum of **7k**

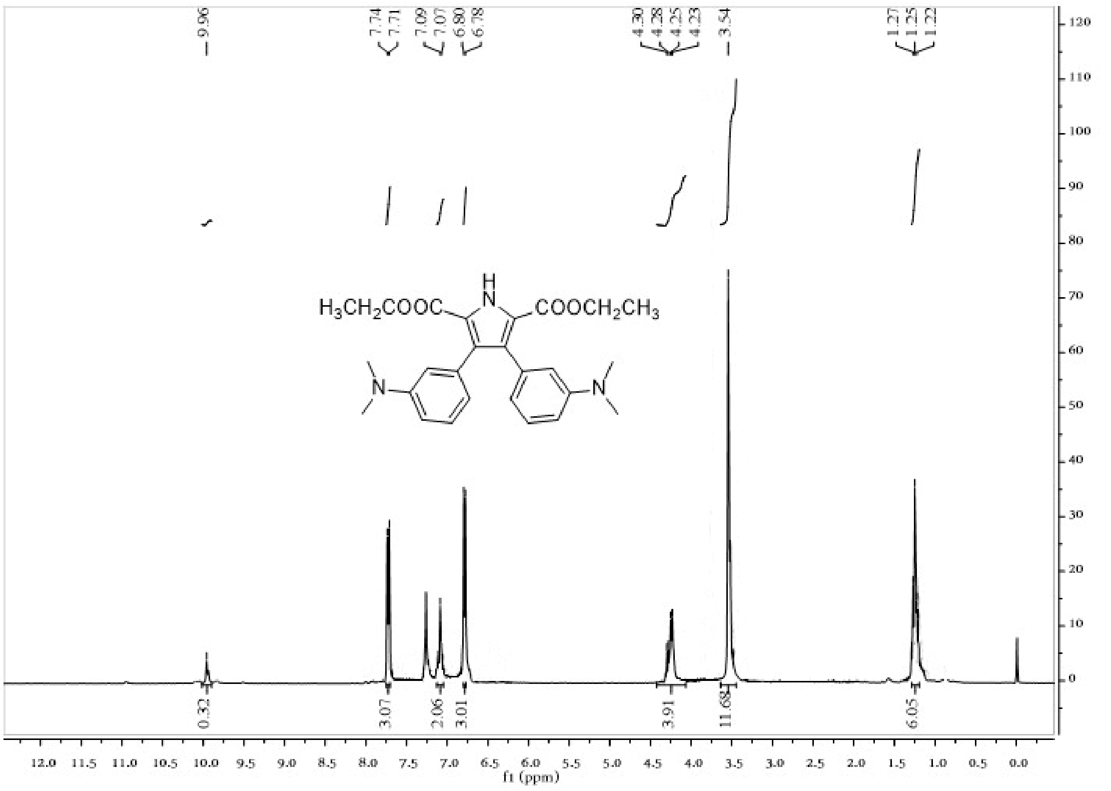

Figure S75. ^{1}H NMR spectrum of **7l**

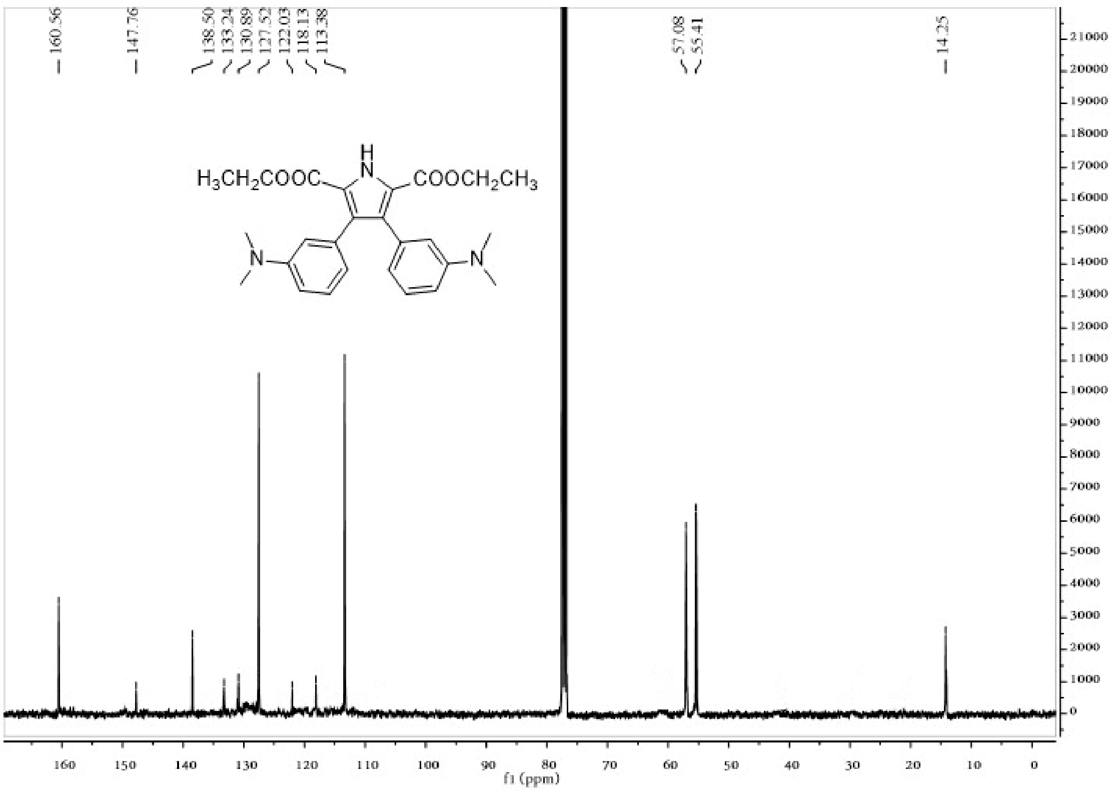

Figure S76. ^{13}C NMR spectrum of **7l**

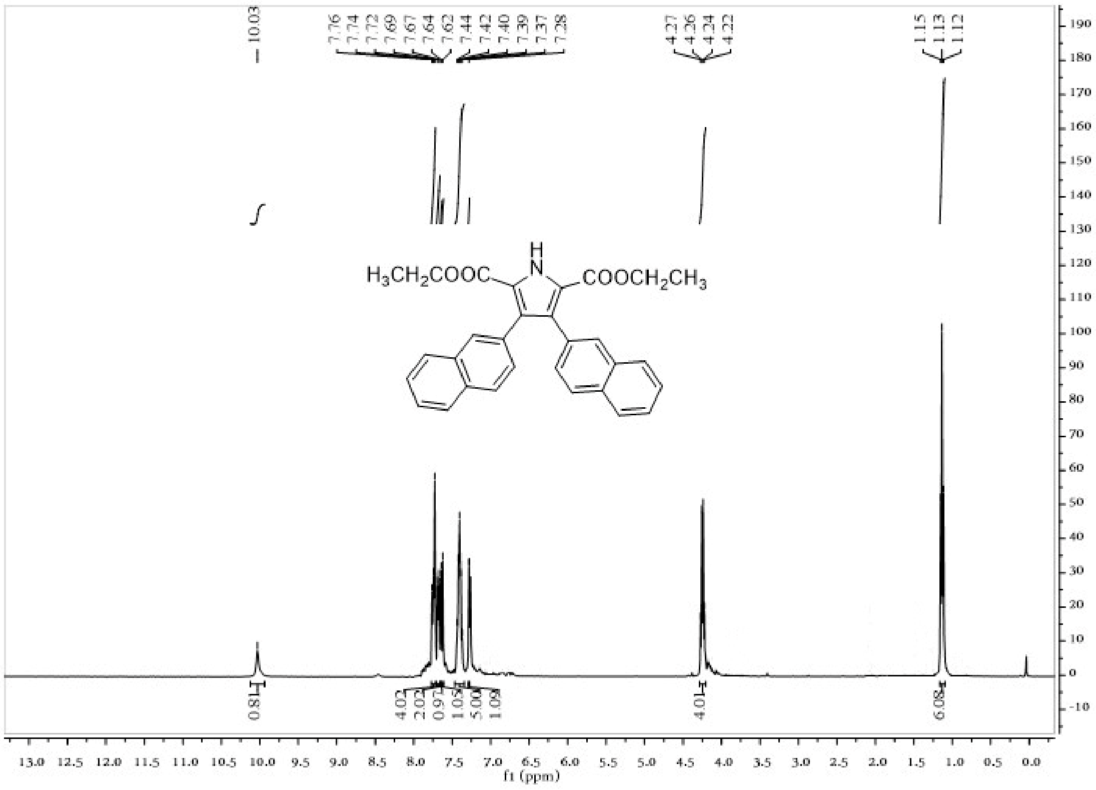

Figure S77. ^{1}H NMR spectrum of **7m**

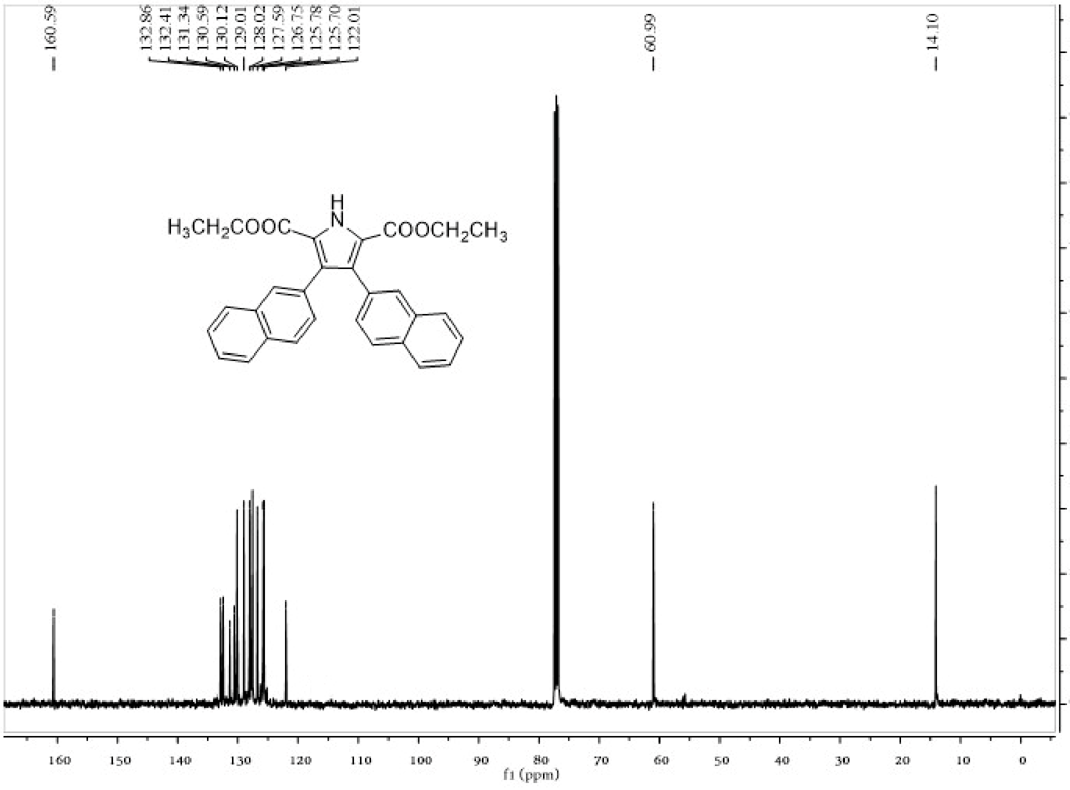

Figure S78. ^{13}C NMR spectrum of **7m**

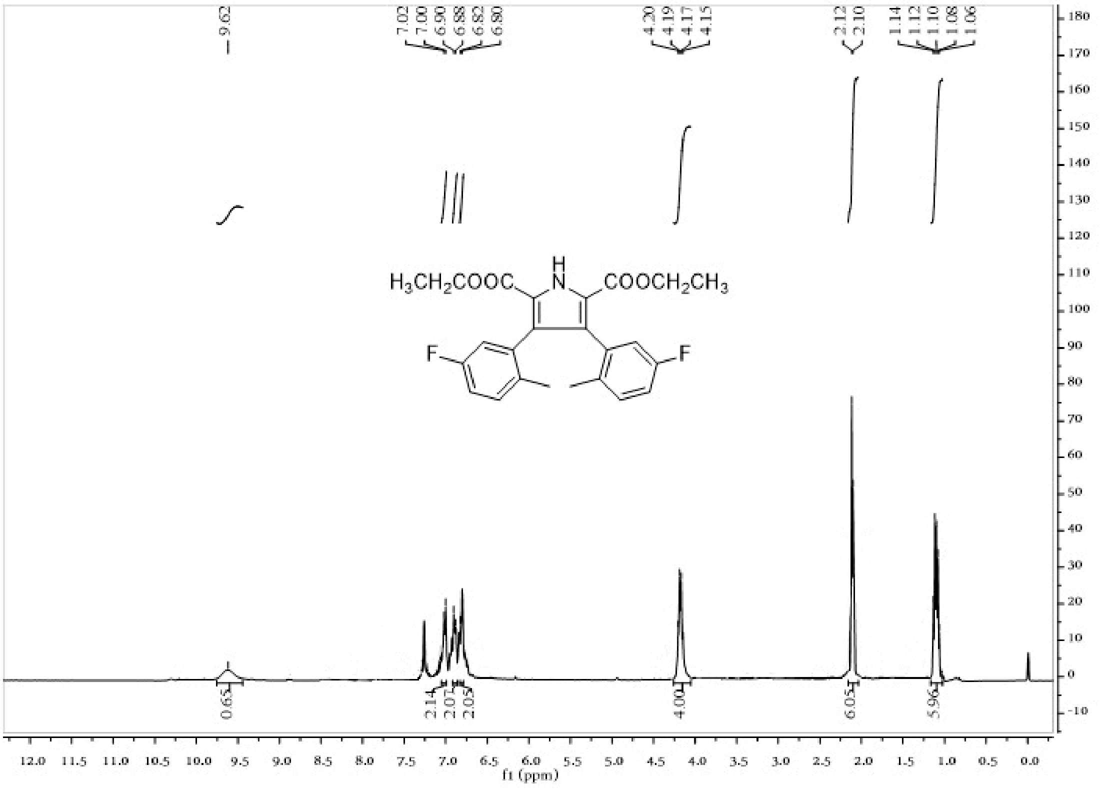

Figure S79. ^{1}H NMR spectrum of **7n**

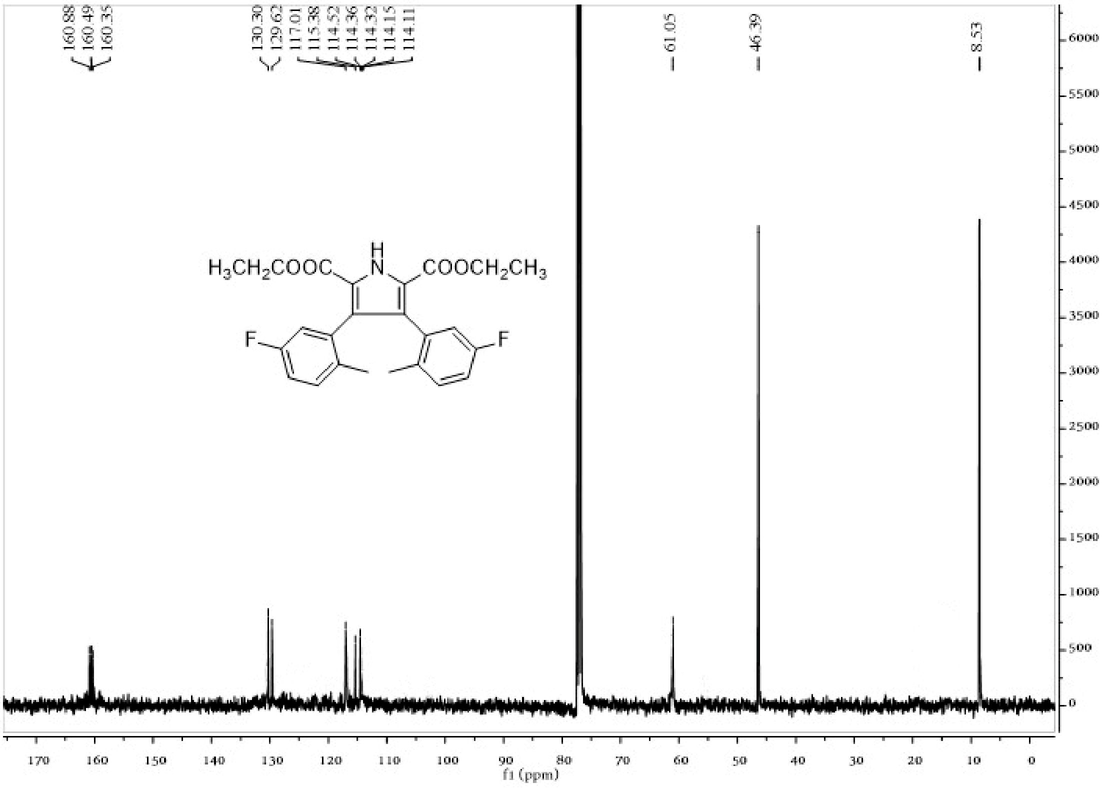

Figure S80. ^{13}C NMR spectrum of **7n**

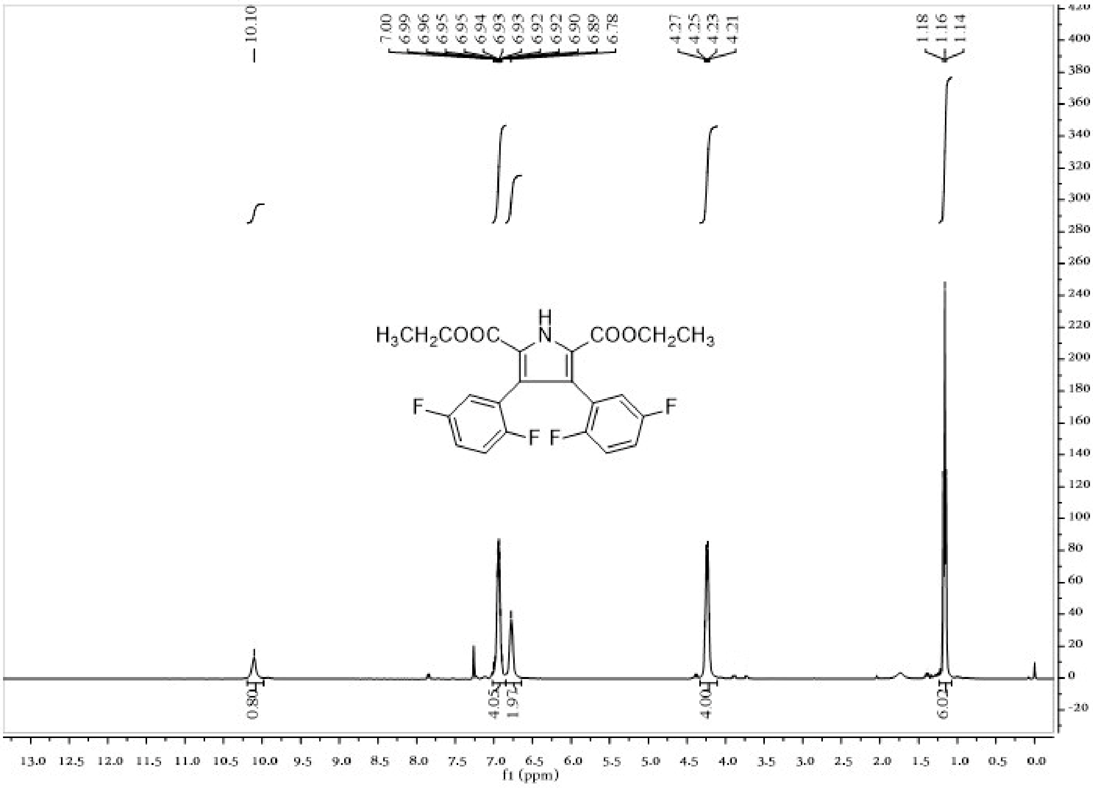

Figure S81. ^{1}H NMR spectrum of **7o**

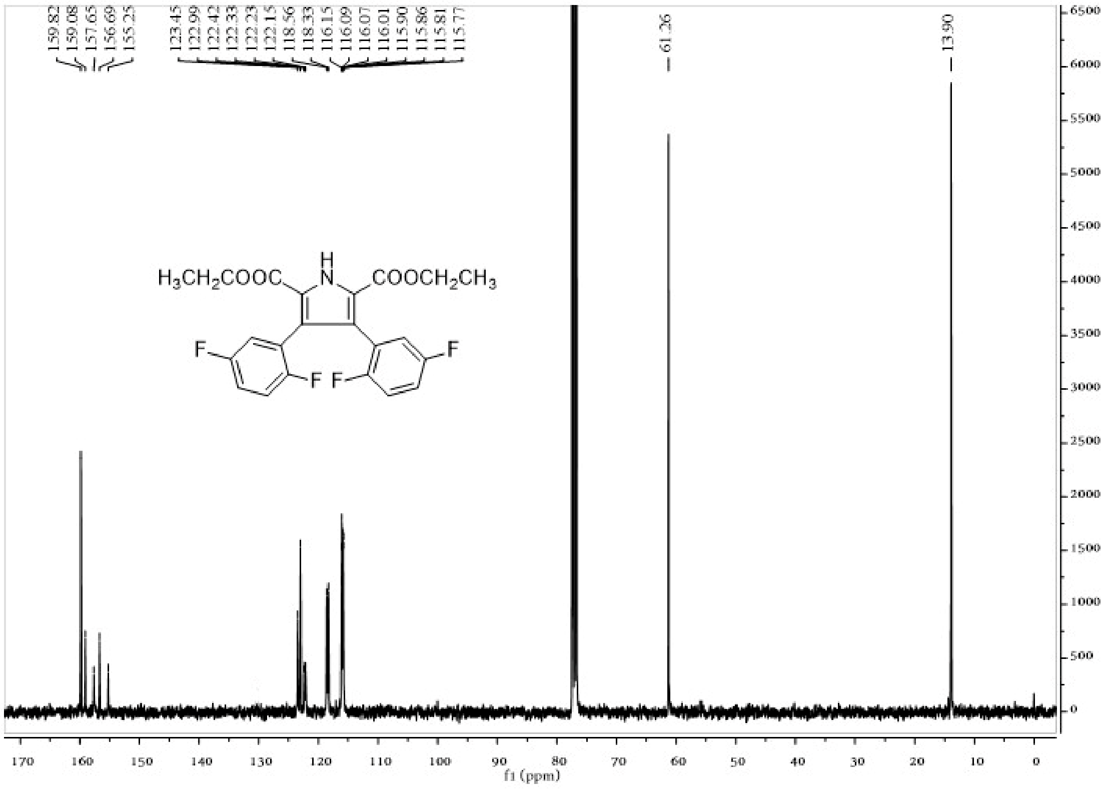

Figure S82. ^{13}C NMR spectrum of **7o**

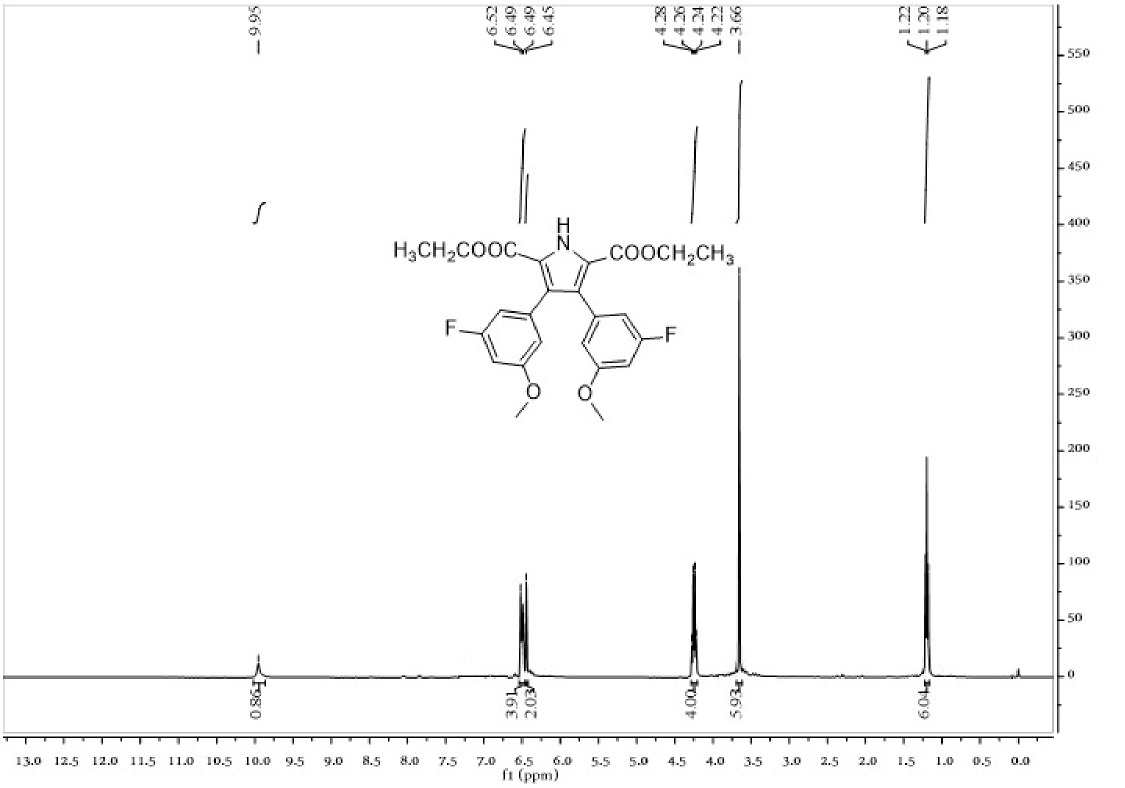

Figure S83. ^{1}H NMR spectrum of **7p**

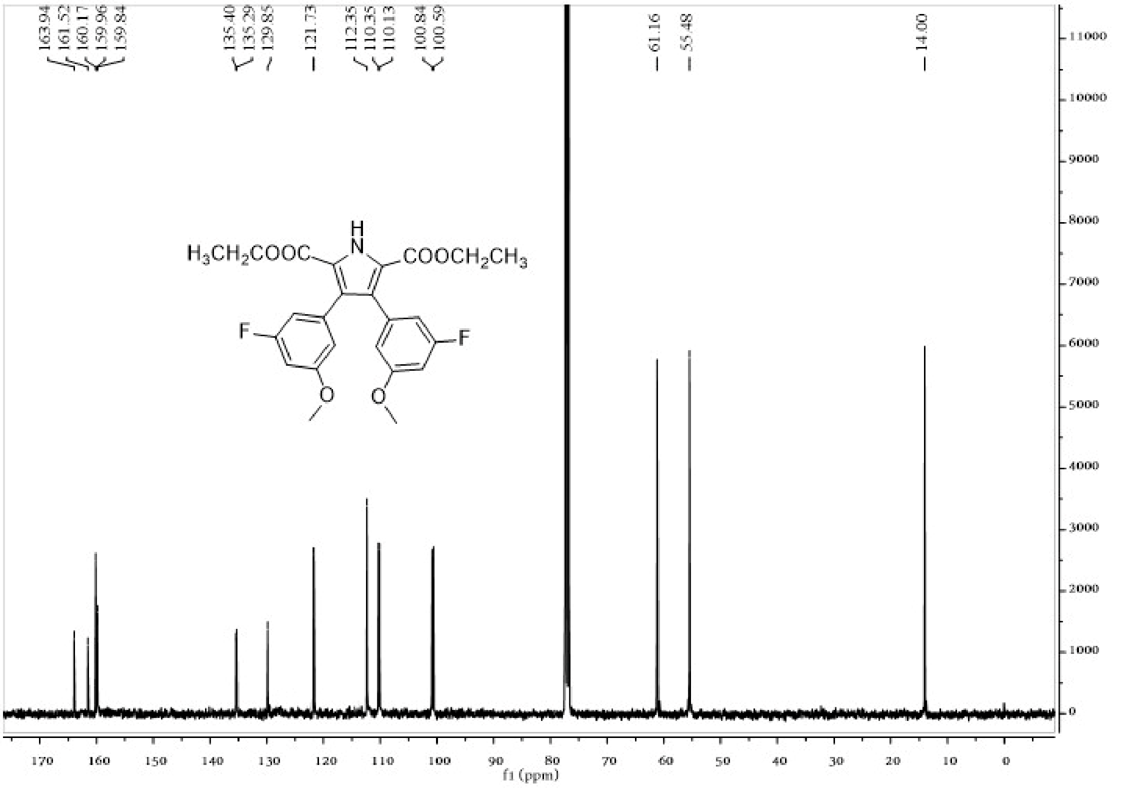

Figure S84. ^{13}C NMR spectrum of **7p**

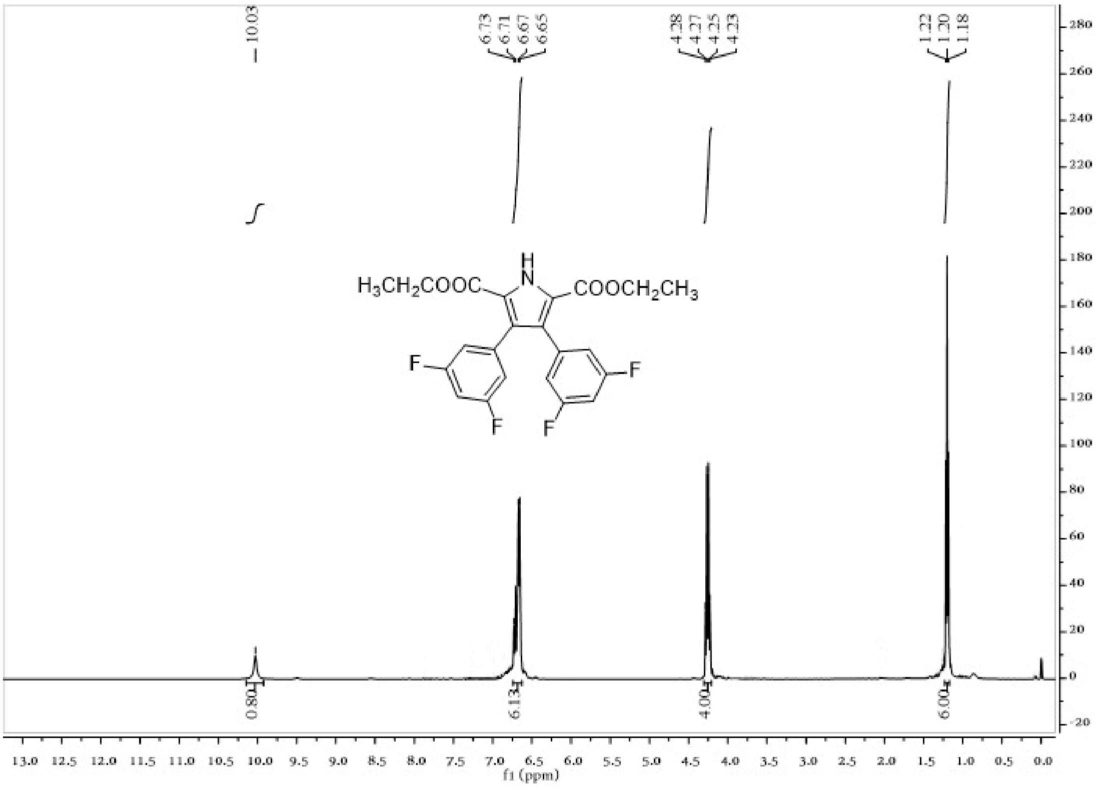

Figure S85. ^{1}H NMR spectrum of **7q**

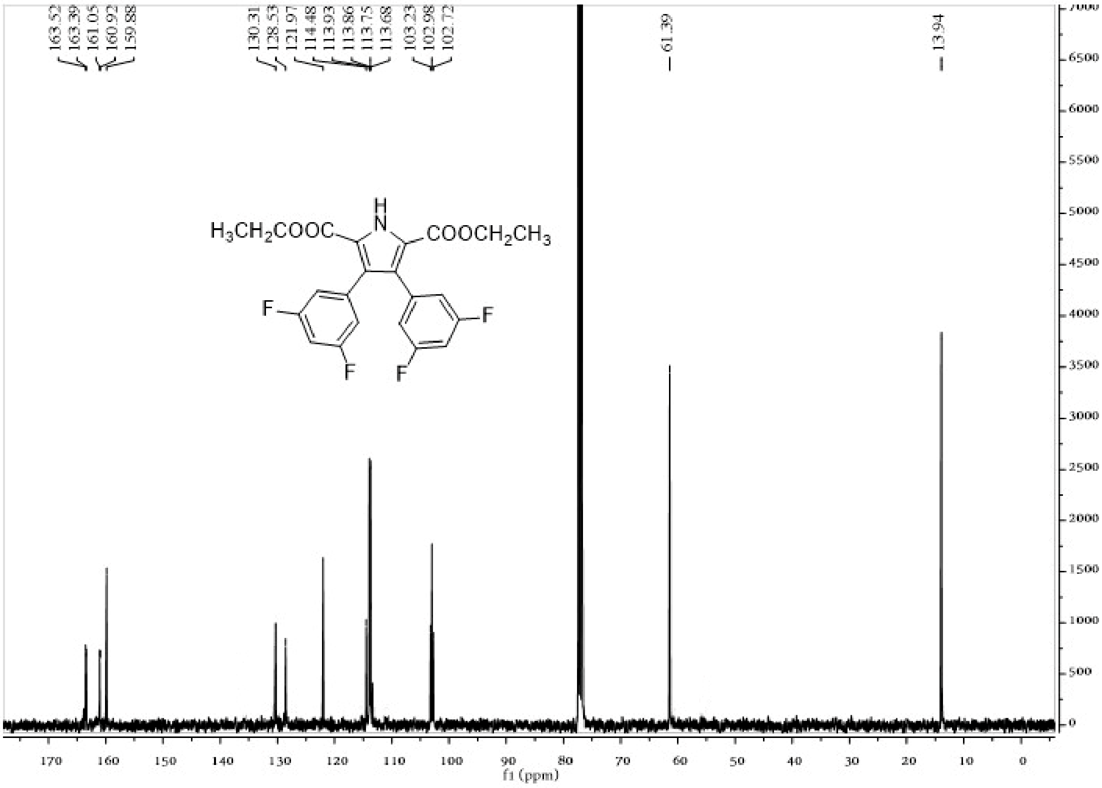

Figure S86. ^{13}C NMR spectrum of **7q**

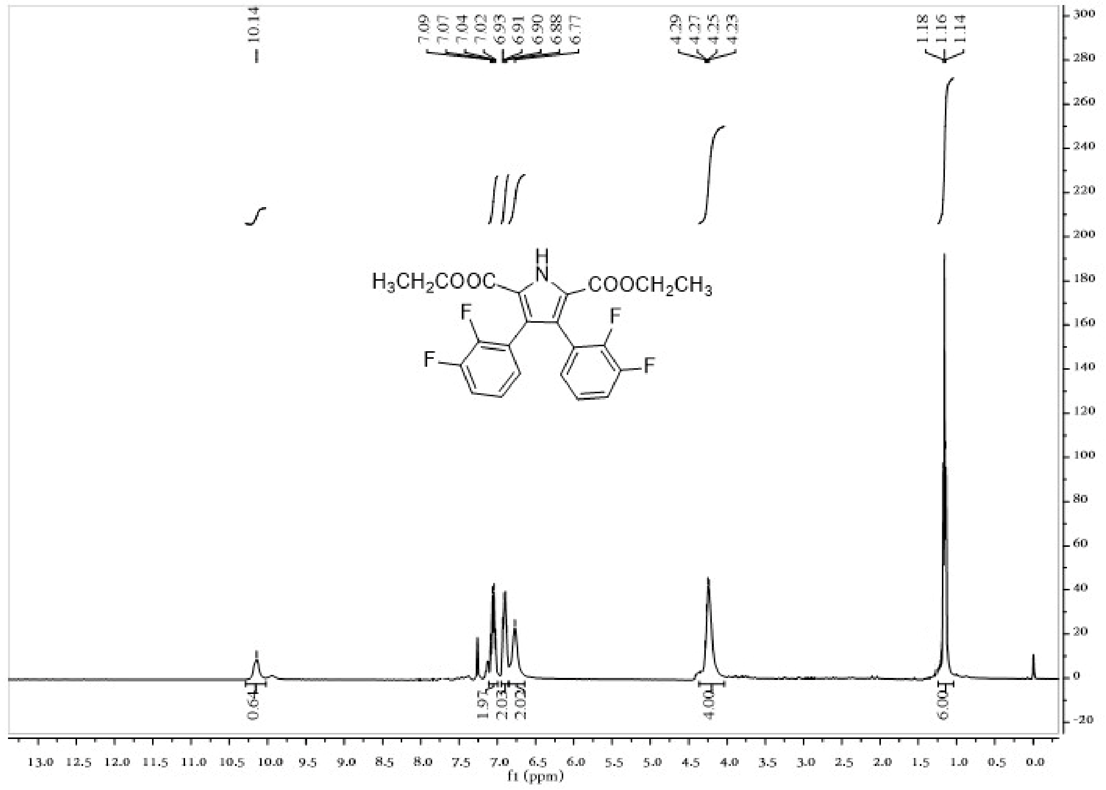

Figure S87. ^{1}H NMR spectrum of **7r**

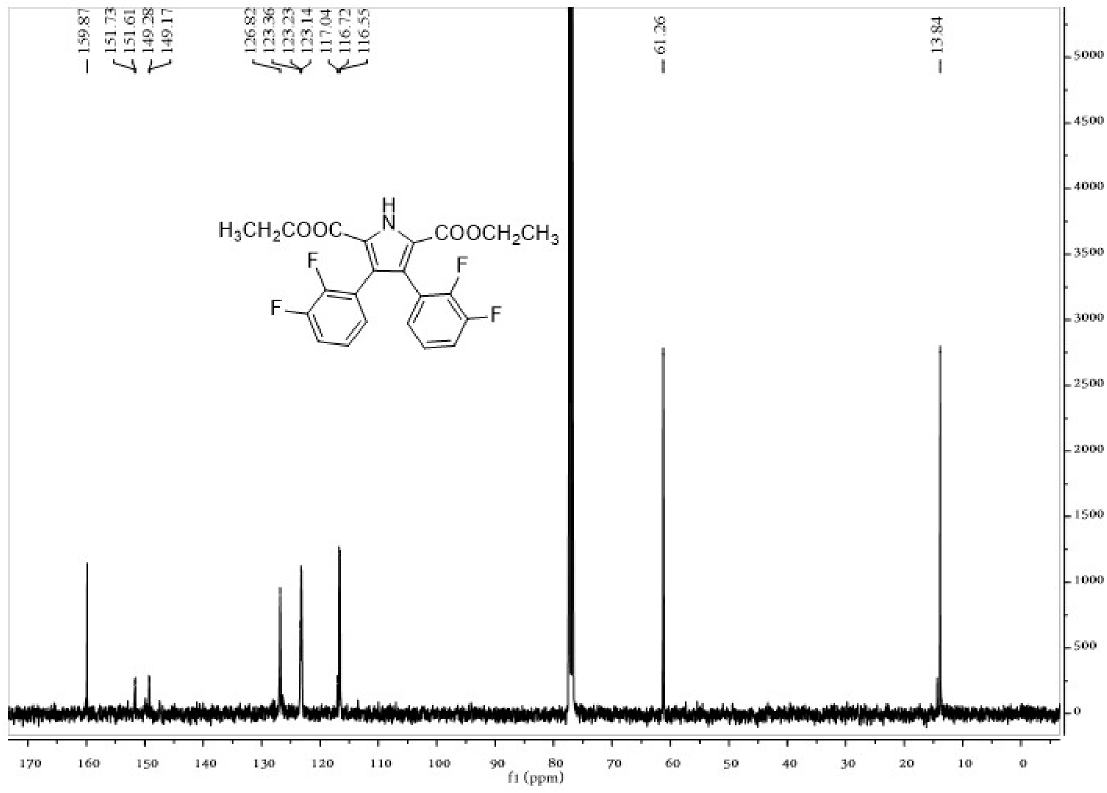

Figure S88. ^{13}C NMR spectrum of **7r**

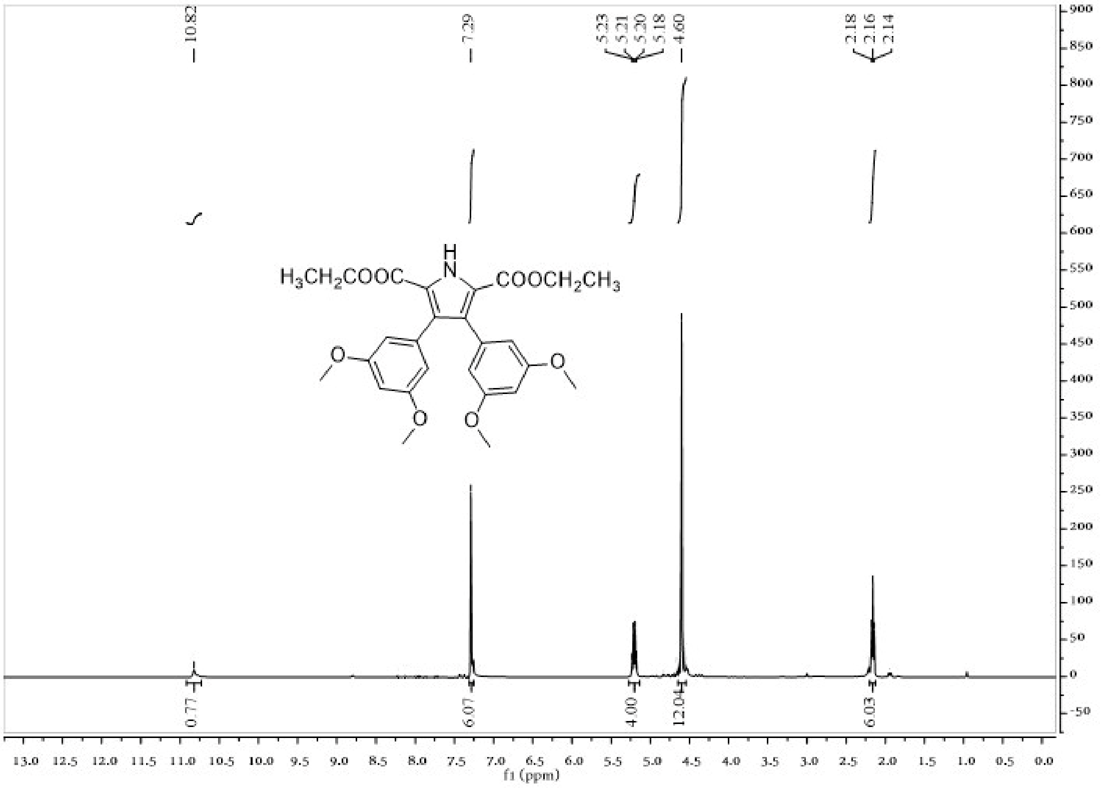

Figure S89. ^{1}H NMR spectrum of **7s**

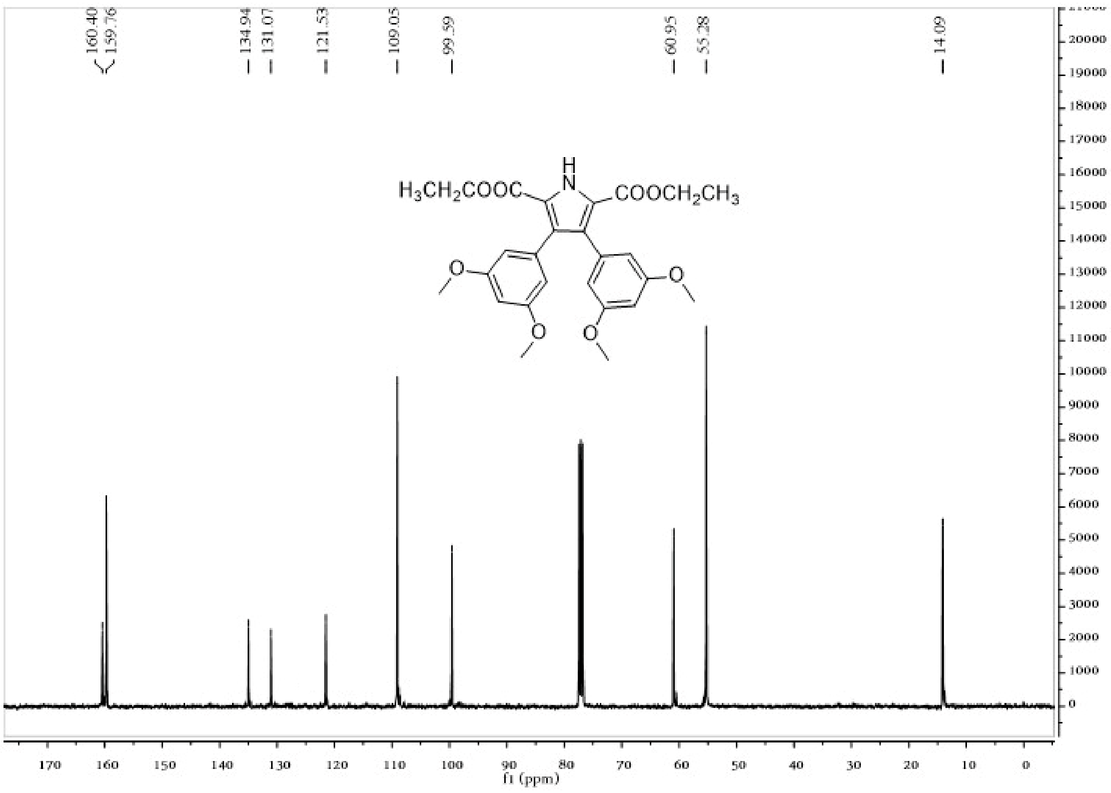

Figure S90. ^{13}C NMR spectrum of **7s**

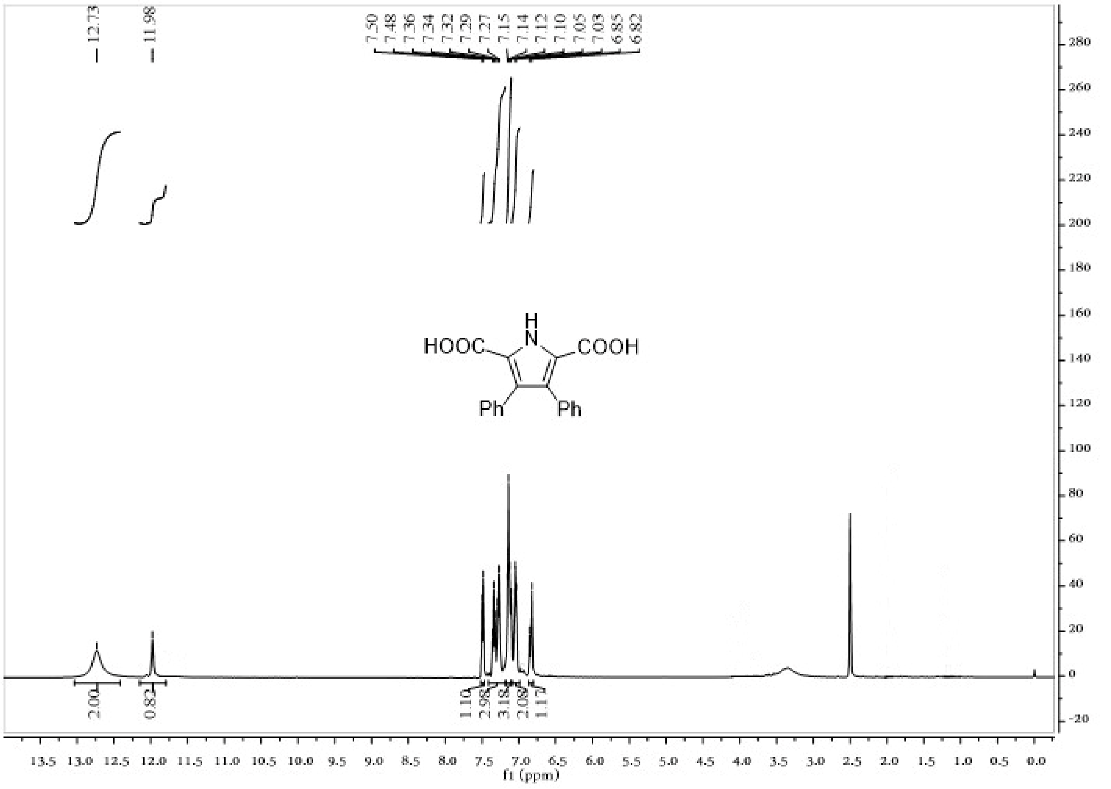

Figure S91. ^{1}H NMR spectrum of **8a**

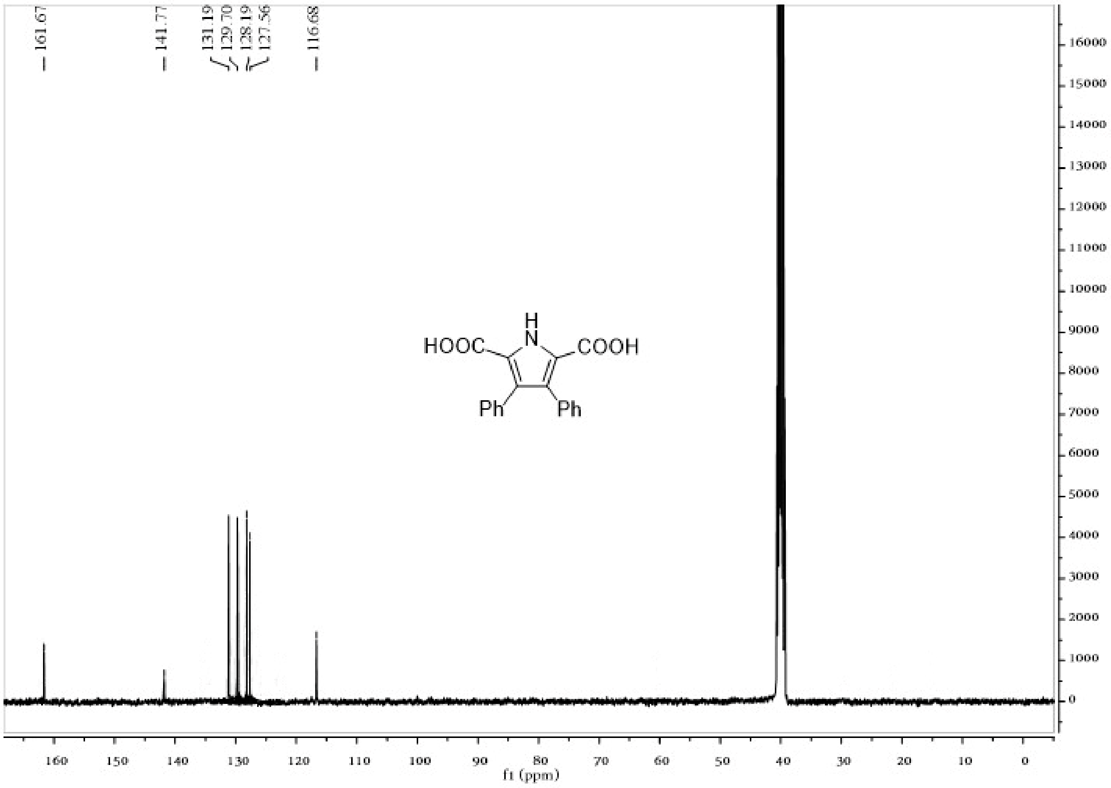

Figure S92. ^{13}C NMR spectrum of **8a**

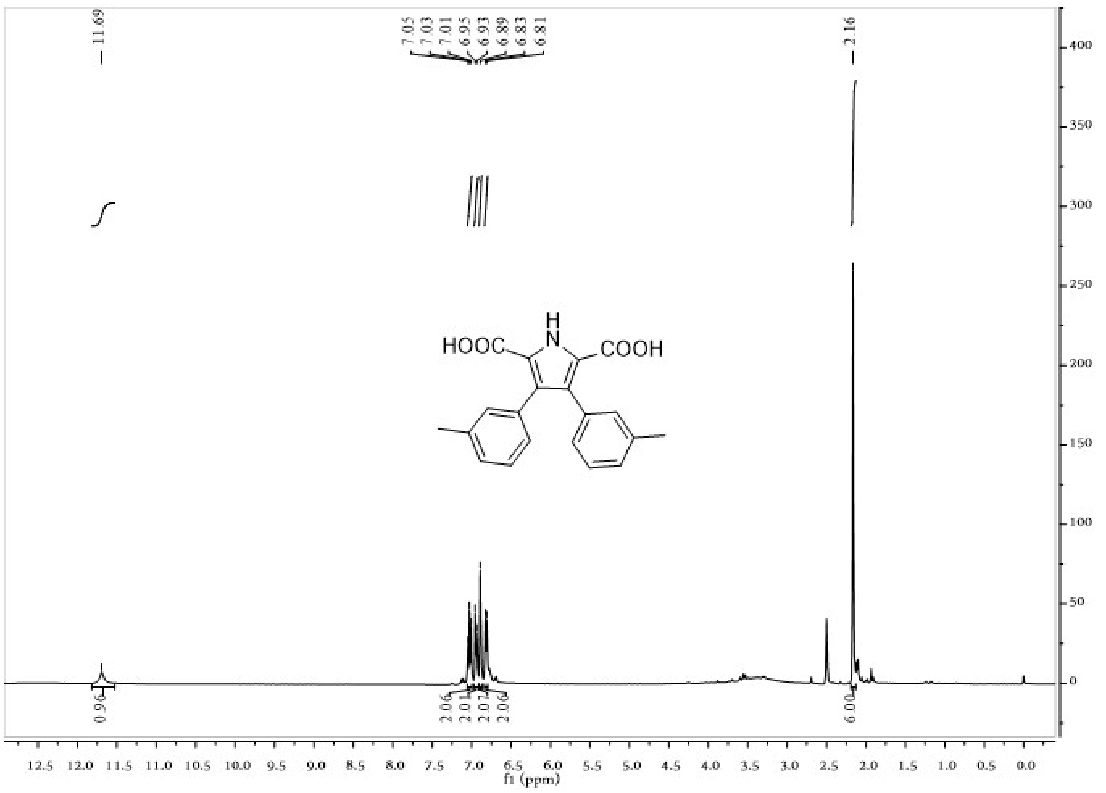

Figure S93. ^{1}H NMR spectrum of **8b**

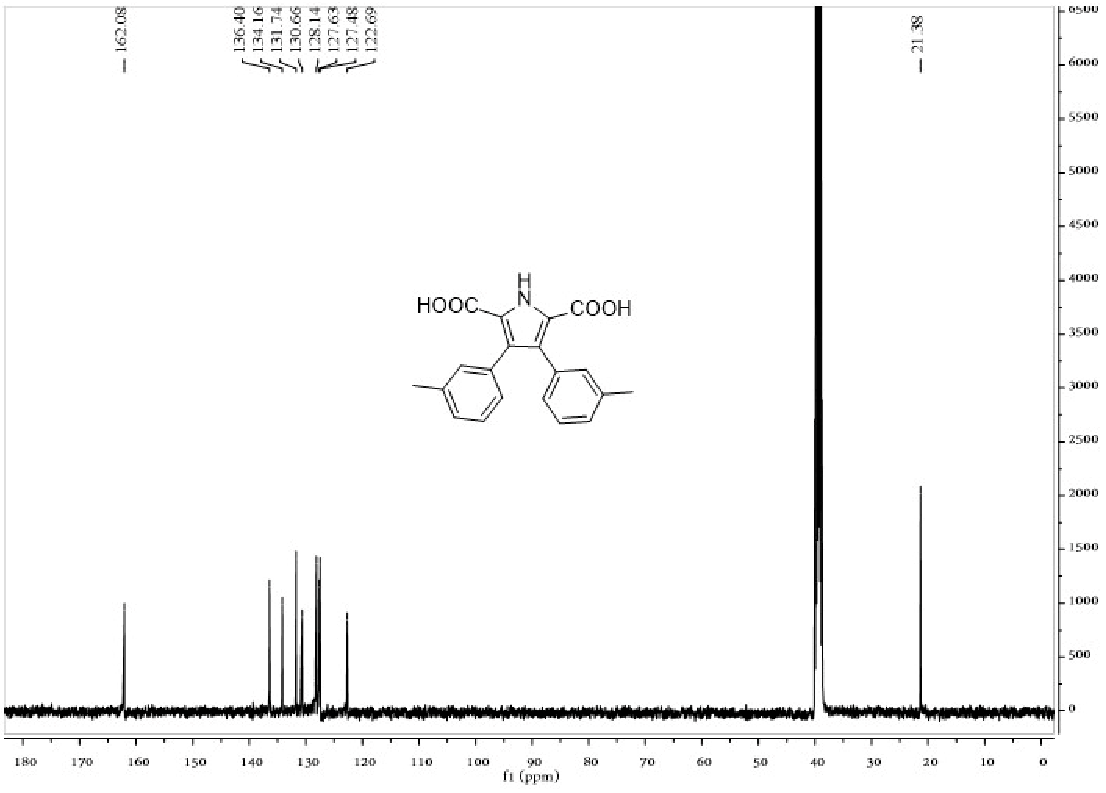

Figure S94. ^{13}C NMR spectrum of **8b**

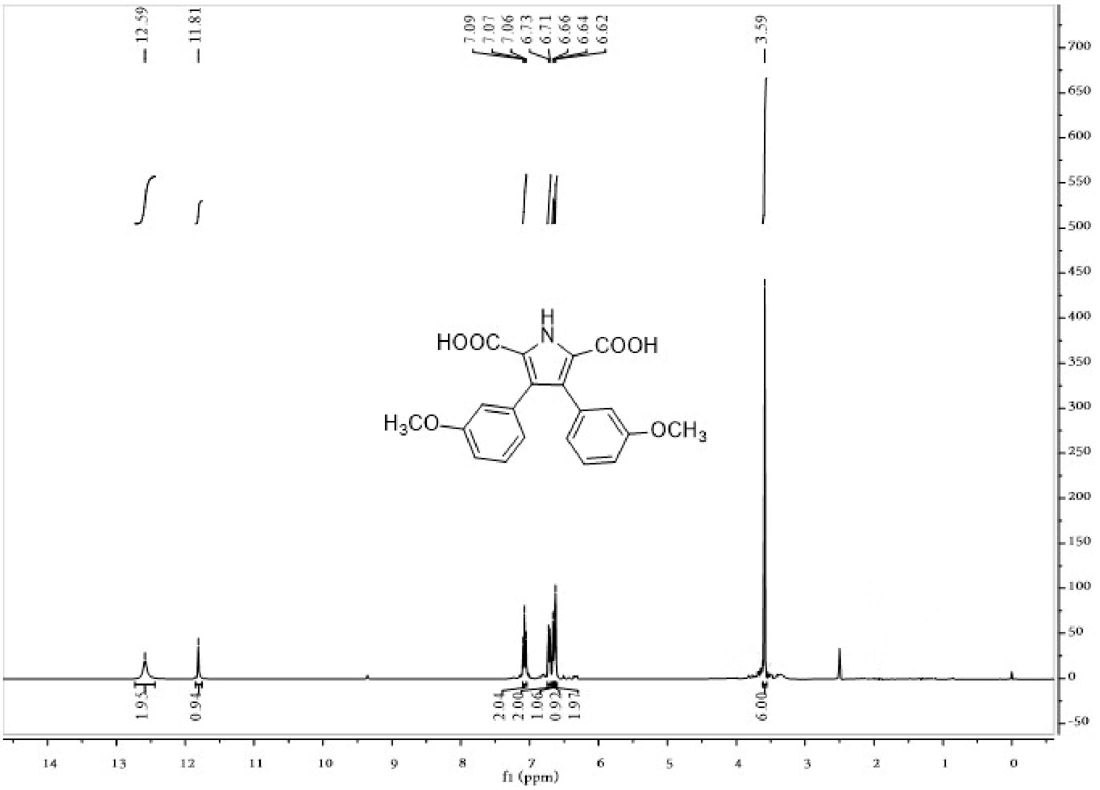

Figure S95. ^{1}H NMR spectrum of **8c**

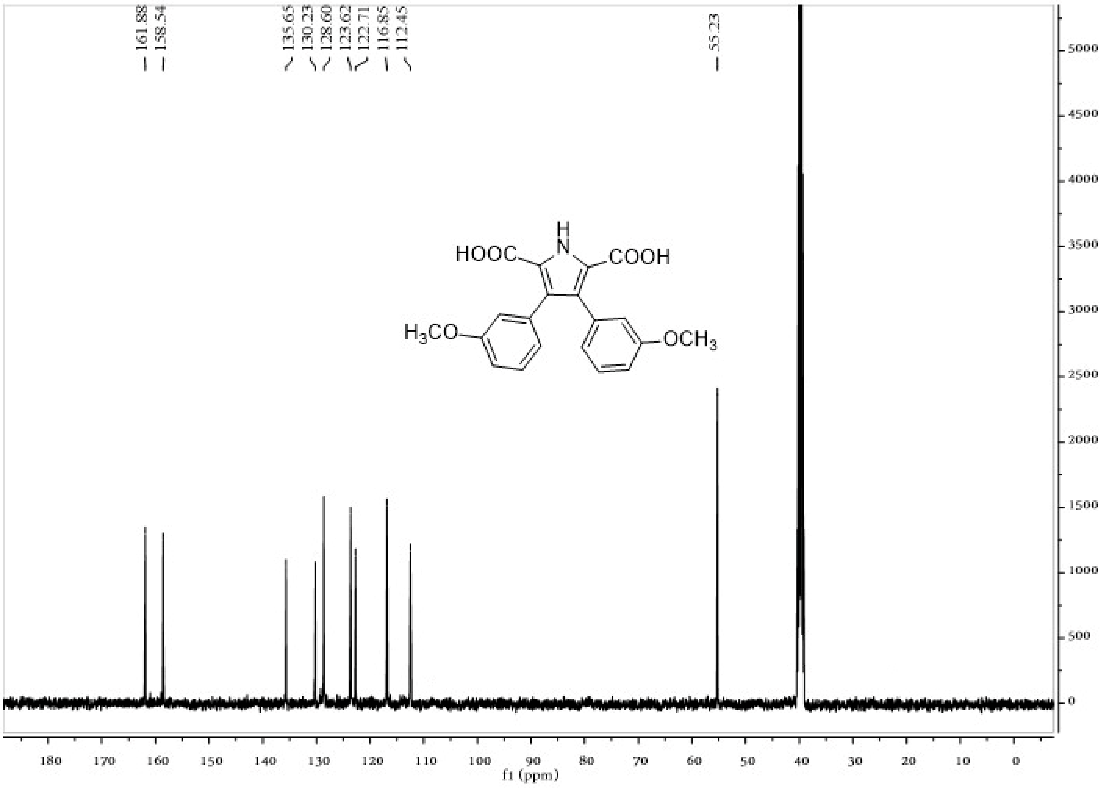

Figure S96. ^{13}C NMR spectrum of **8c**

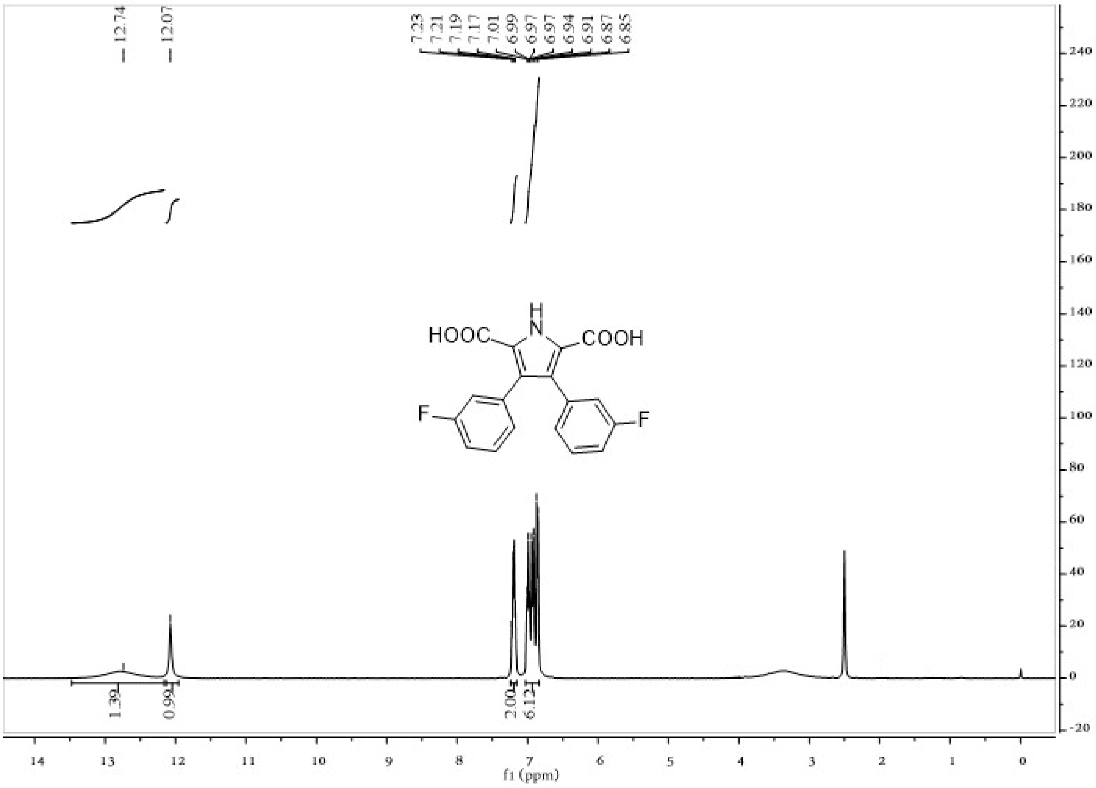

Figure S97. ^{1}H NMR spectrum of **8d**

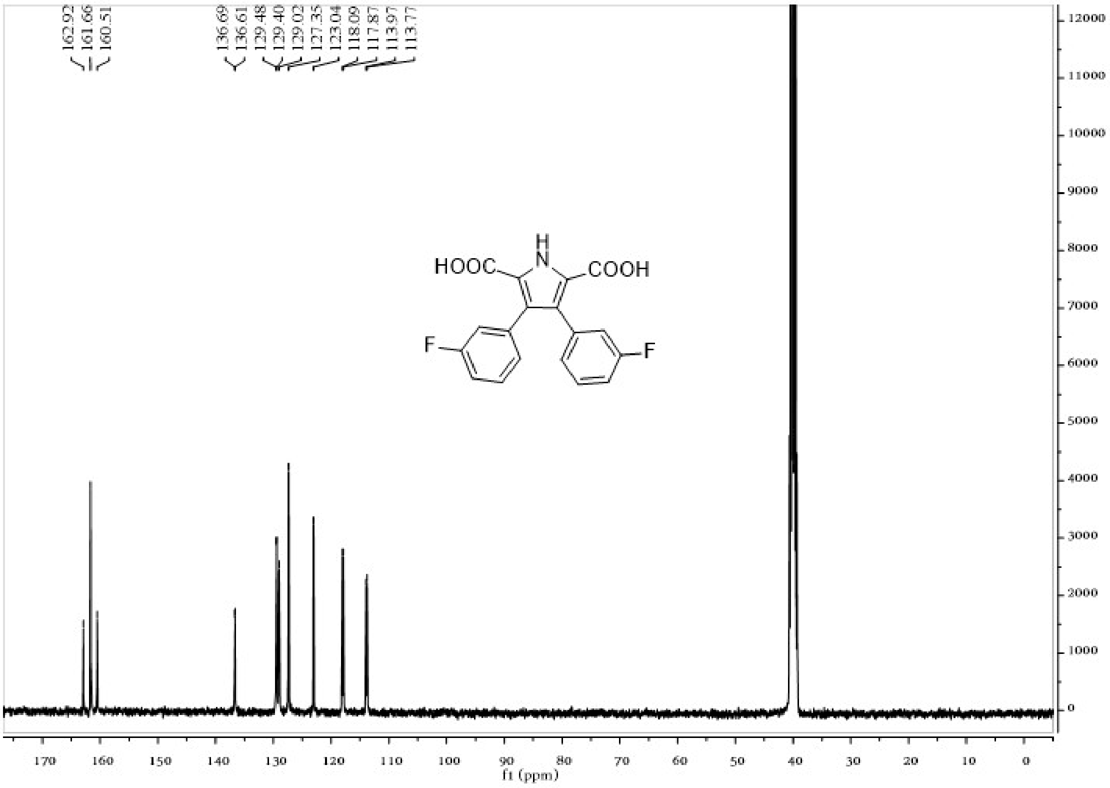

Figure S98. ^{13}C NMR spectrum of **8d**

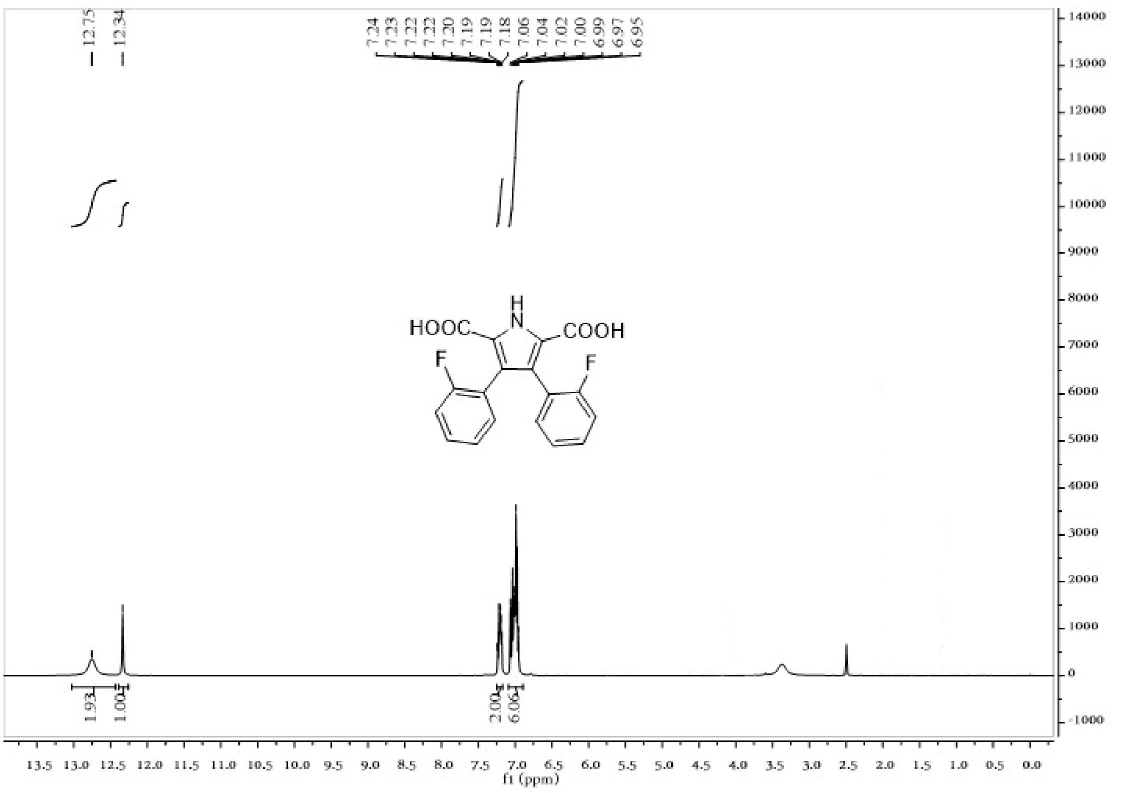

Figure S99. ^{1}H NMR spectrum of **8e**

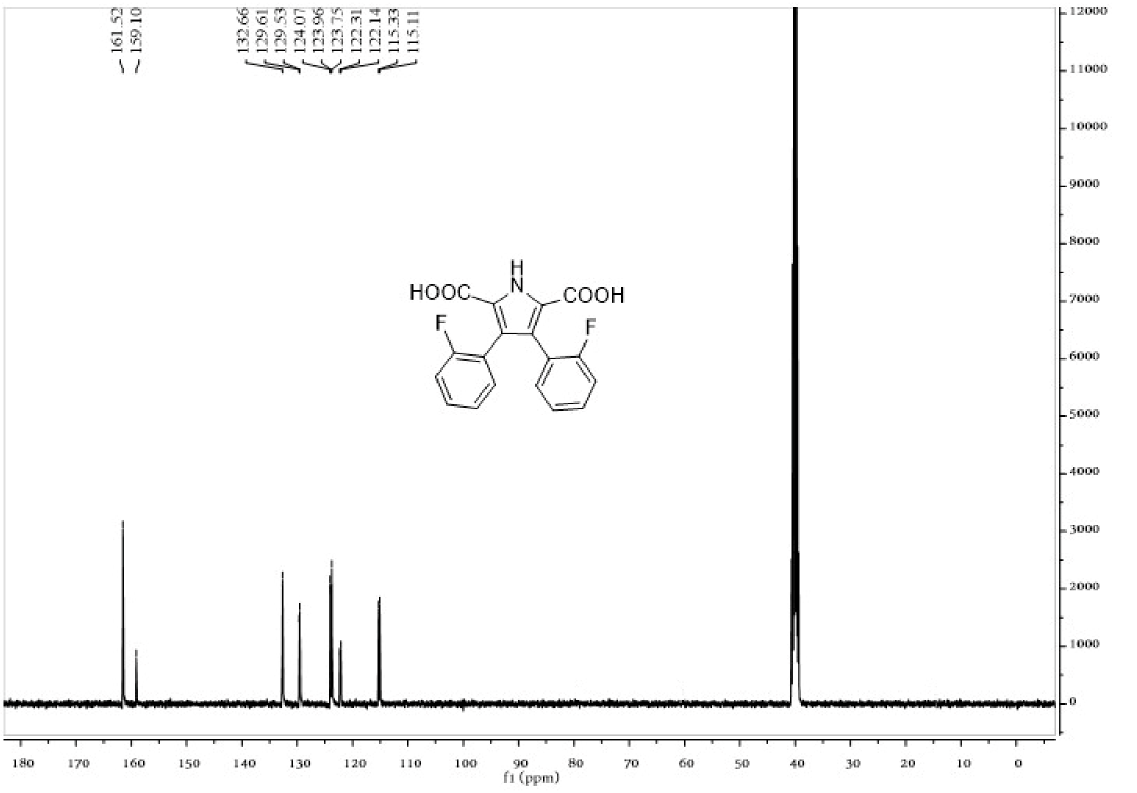

Figure S100. ^{13}C NMR spectrum of **8e**

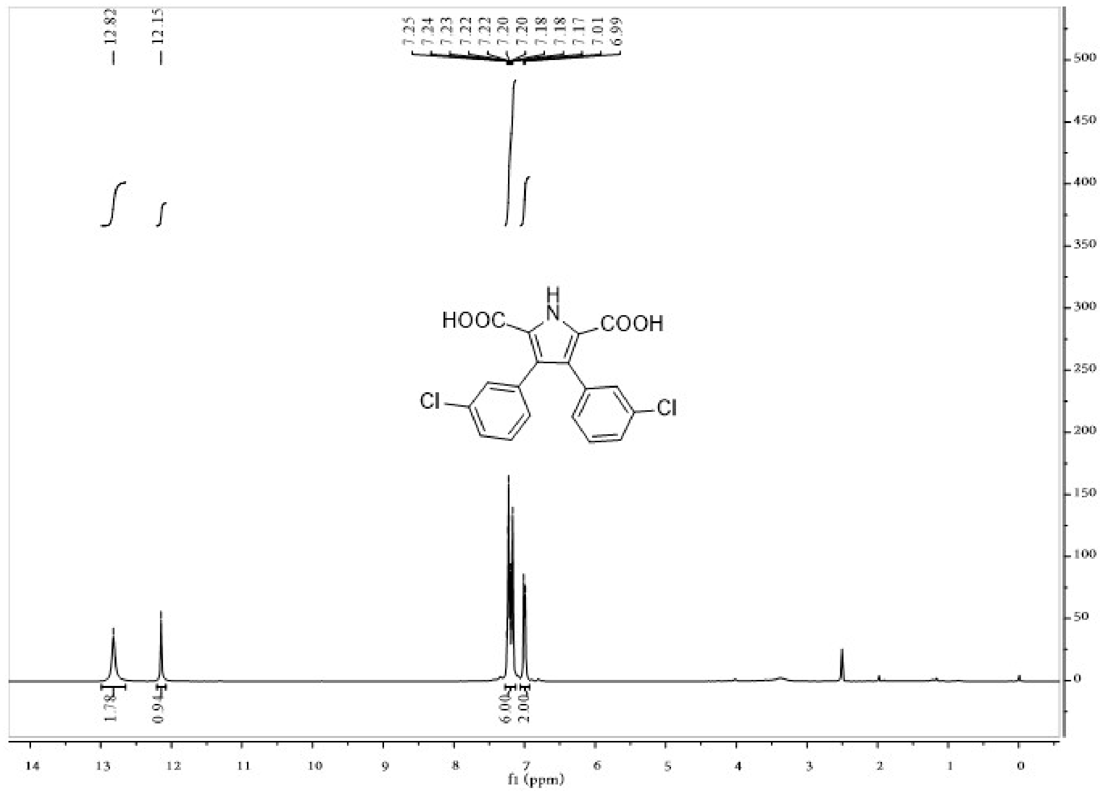

Figure S101. ^{1}H NMR spectrum of **8f**

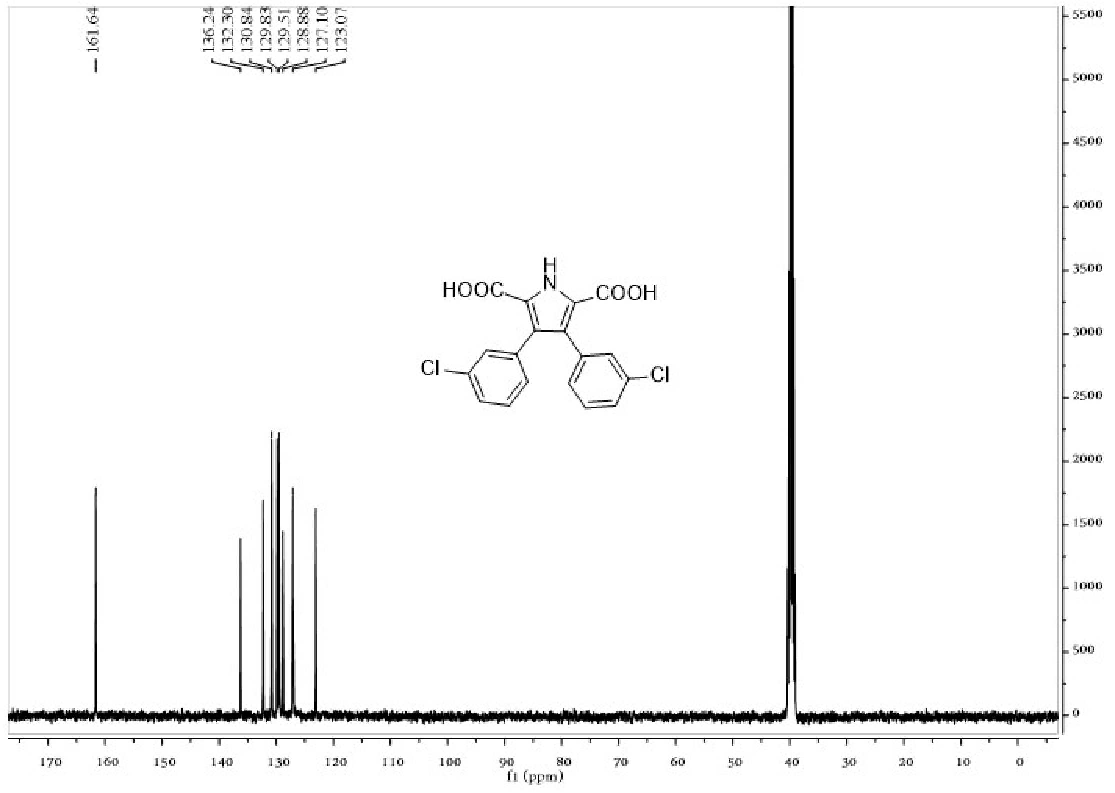

Figure S102. ^{13}C NMR spectrum of **8f**

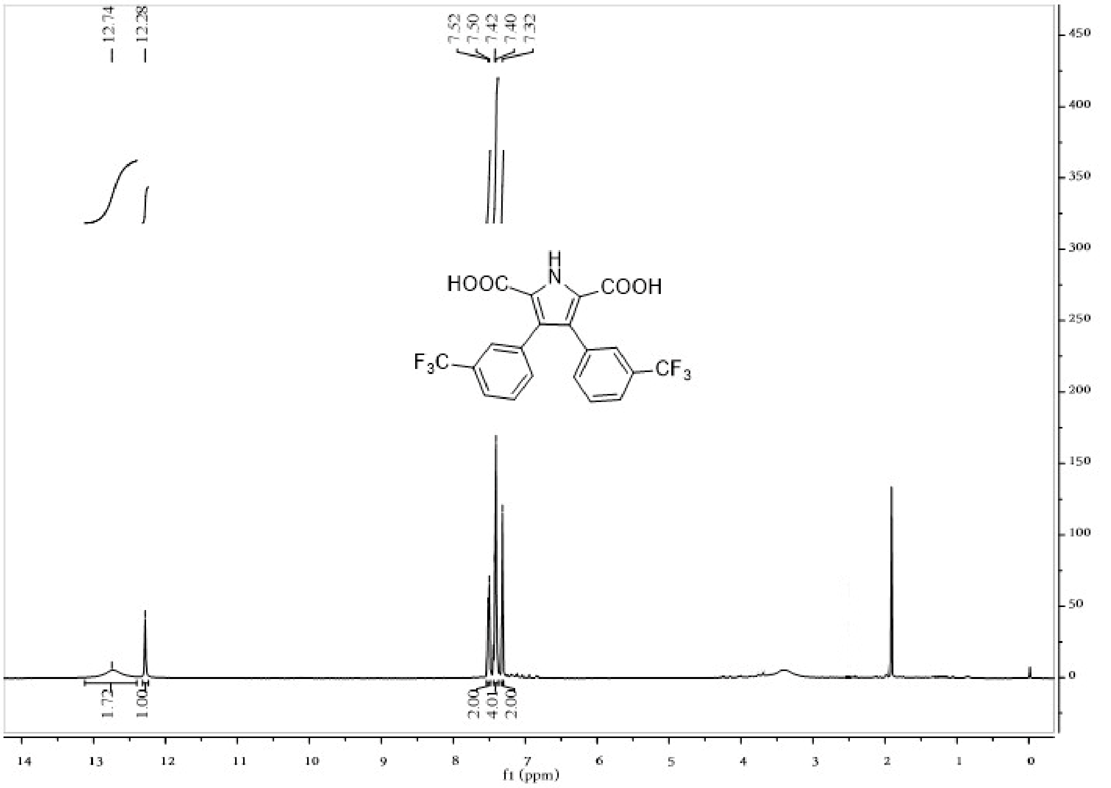

Figure S103. ^{1}H NMR spectrum of **8g**

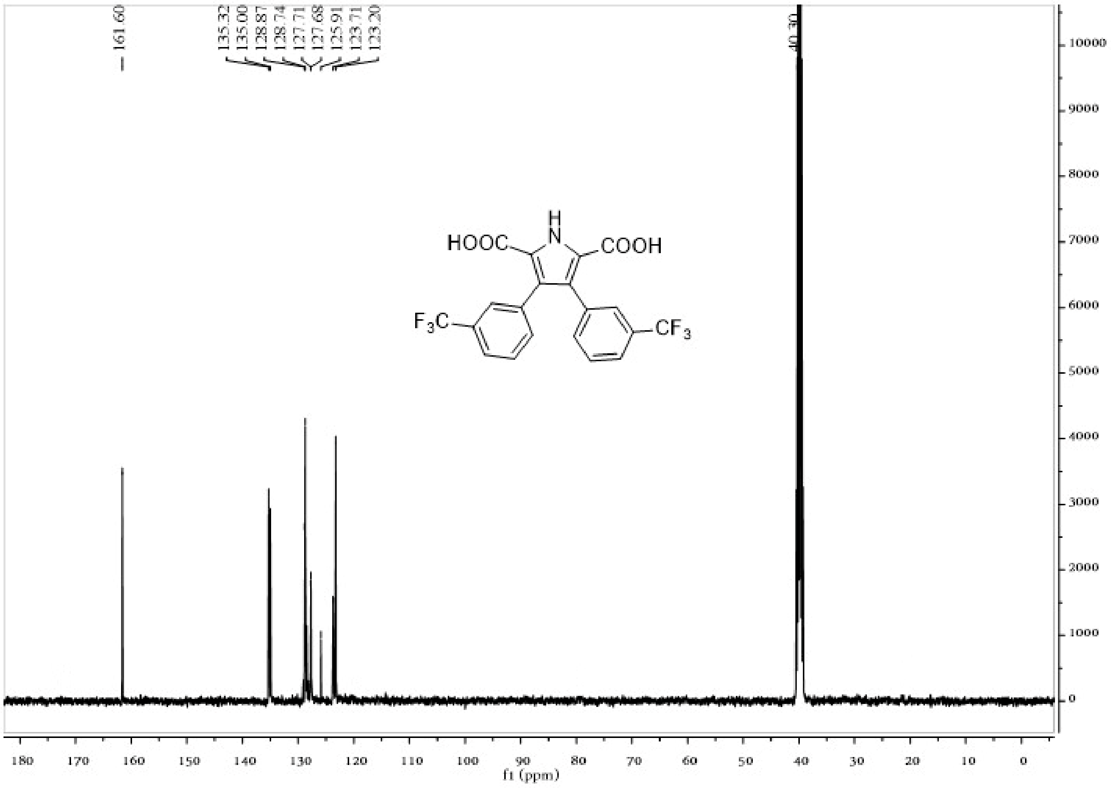

Figure S104. ^{13}C NMR spectrum of **8g**

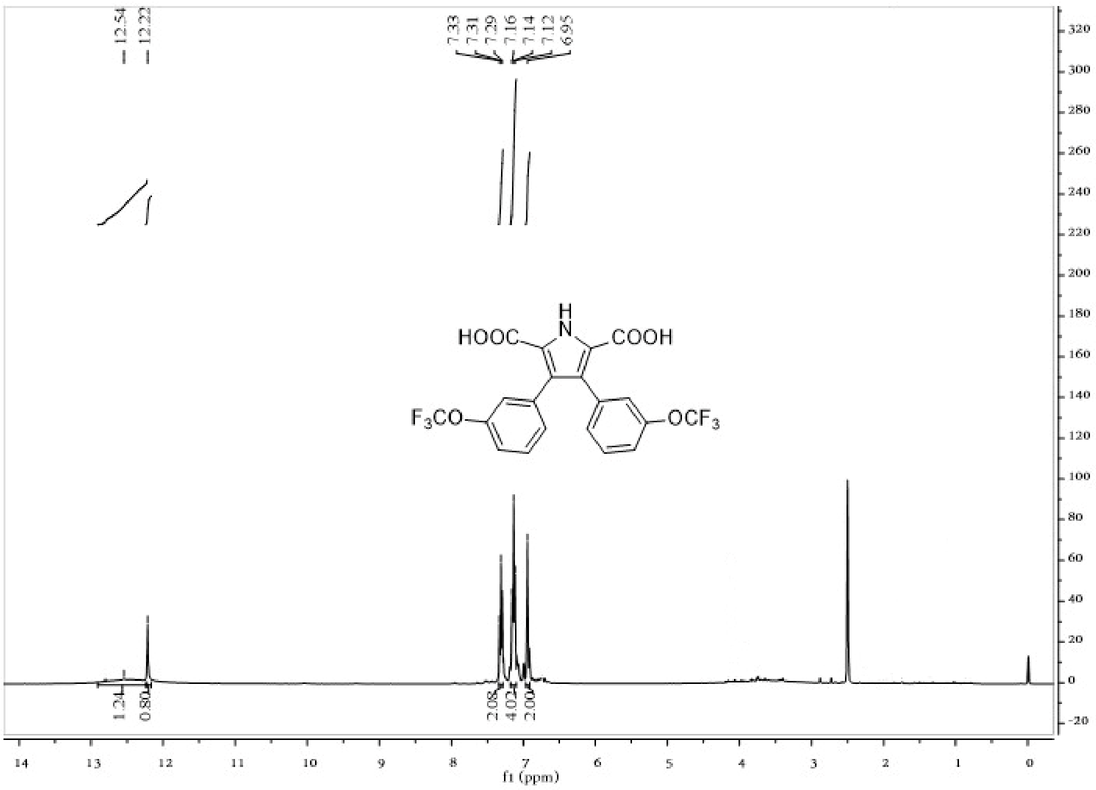

Figure S105. ^{1}H NMR spectrum of **8h**

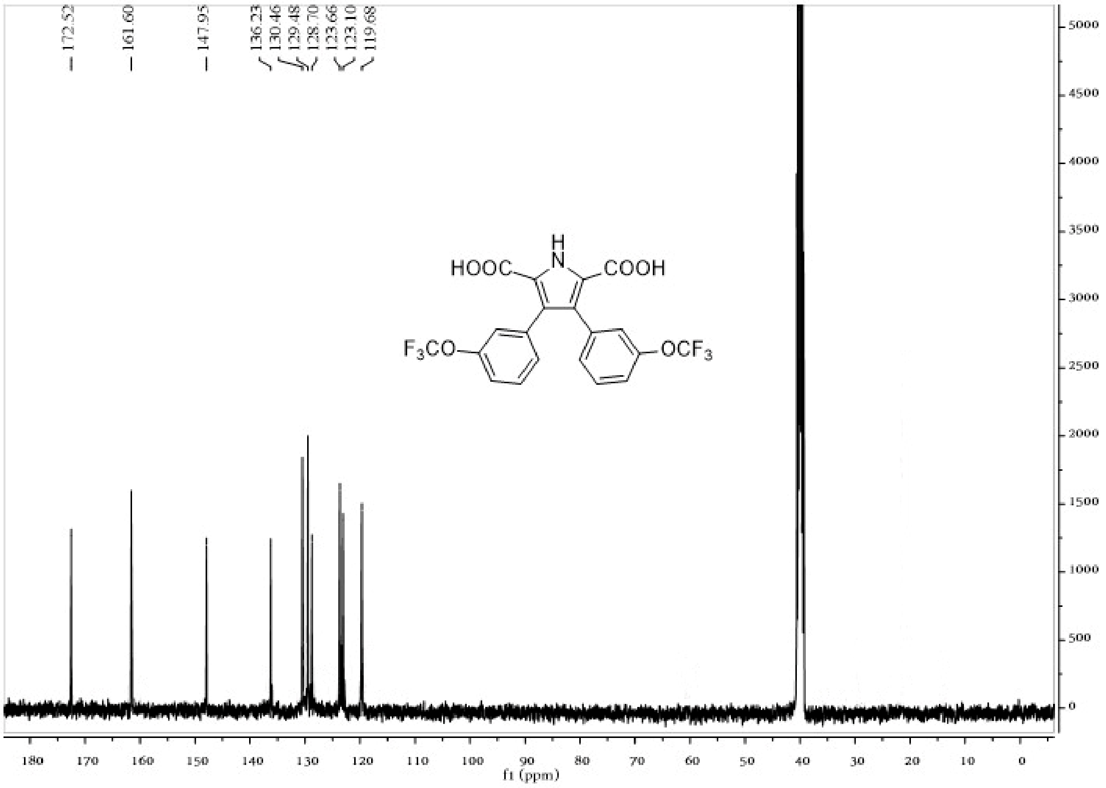

Figure S106. ^{13}C NMR spectrum of **8h**

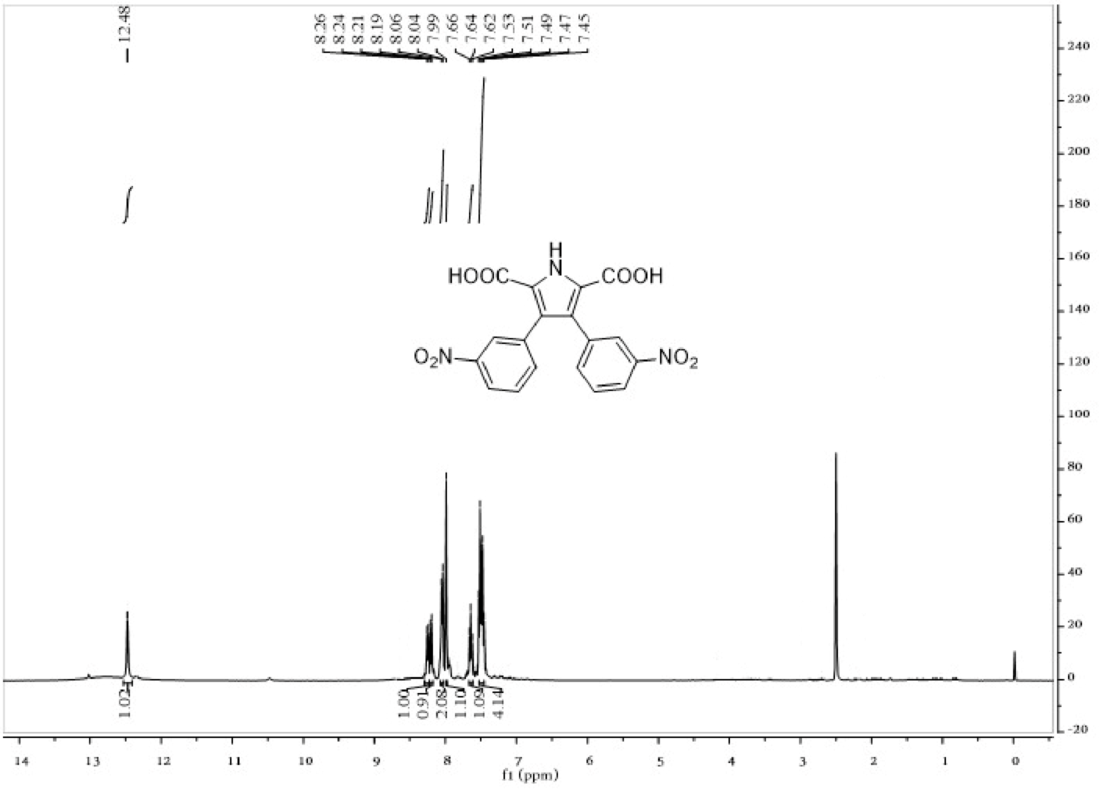

Figure S107. ^{1}H NMR spectrum of **8i**

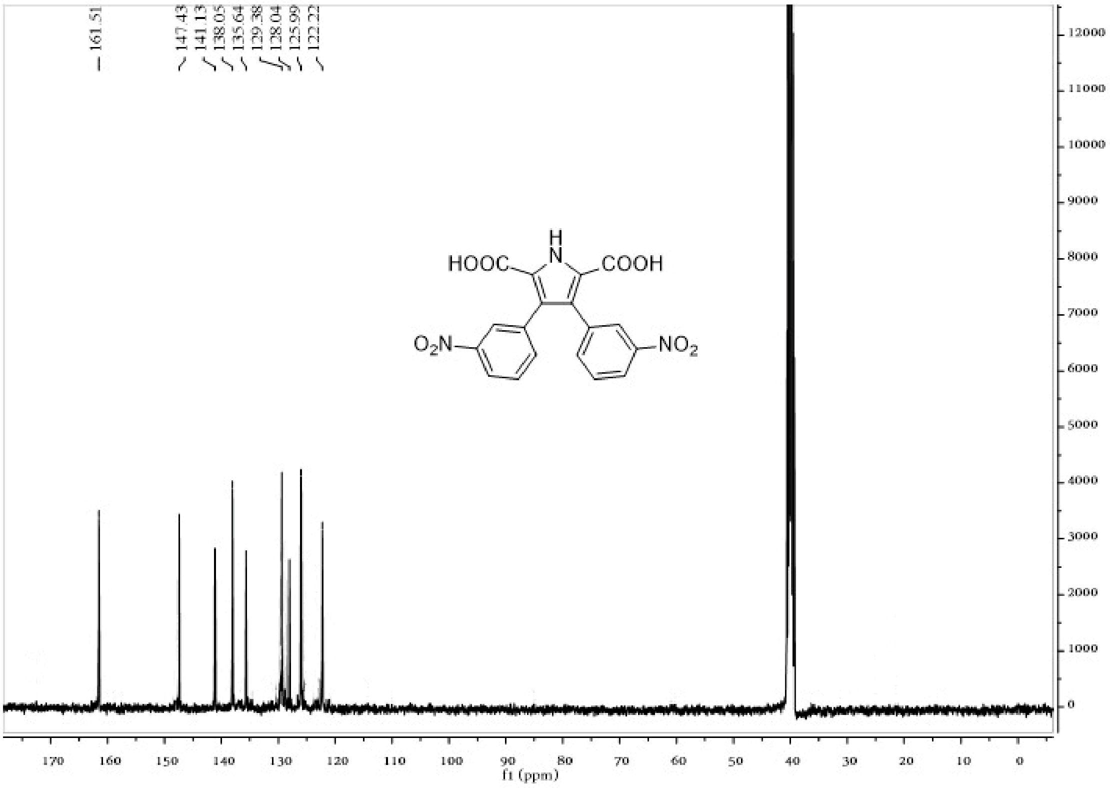

Figure S108. ^{13}C NMR spectrum of **8i**

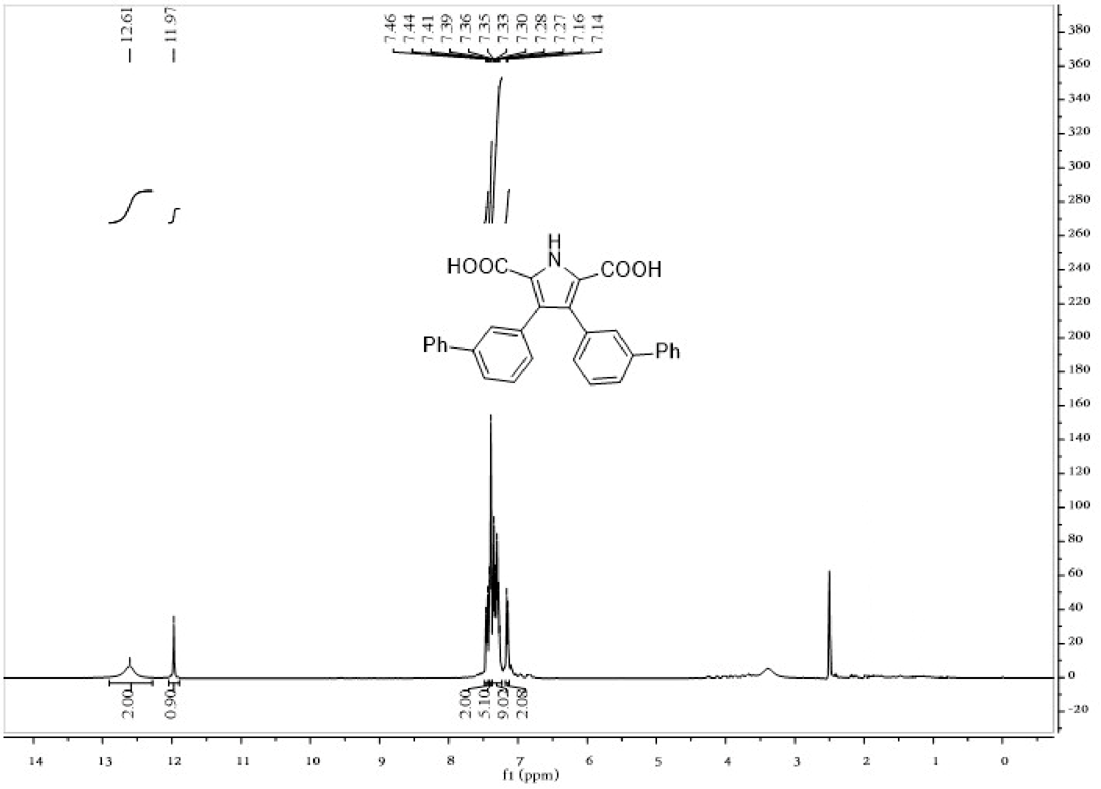

Figure S109. ^{1}H NMR spectrum of **8g**

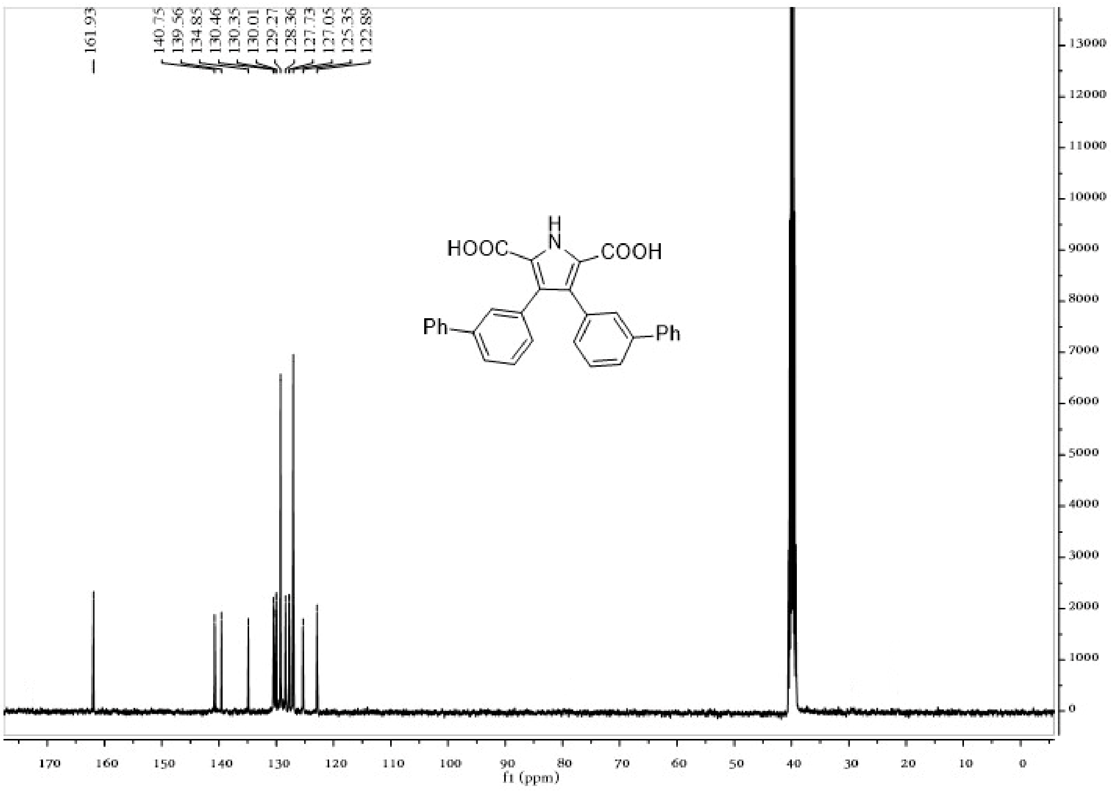

Figure S110. ^{13}C NMR spectrum of **8g**

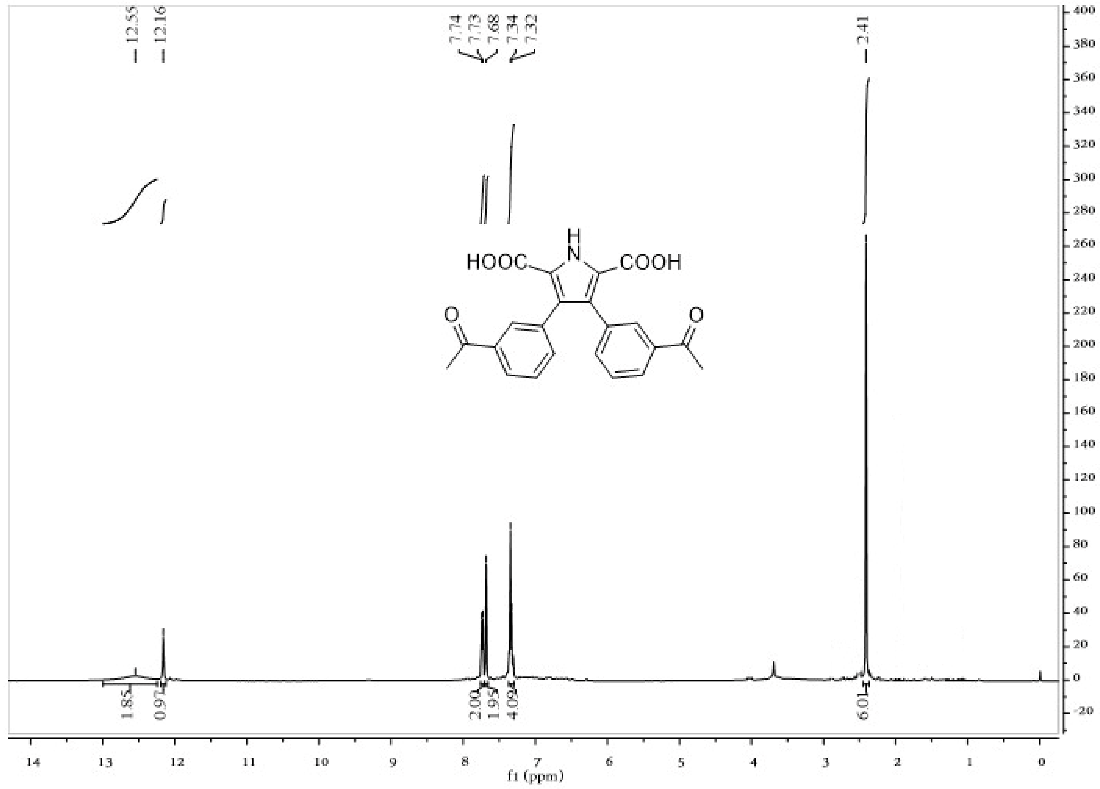

Figure S111. ^{1}H NMR spectrum of **8k**

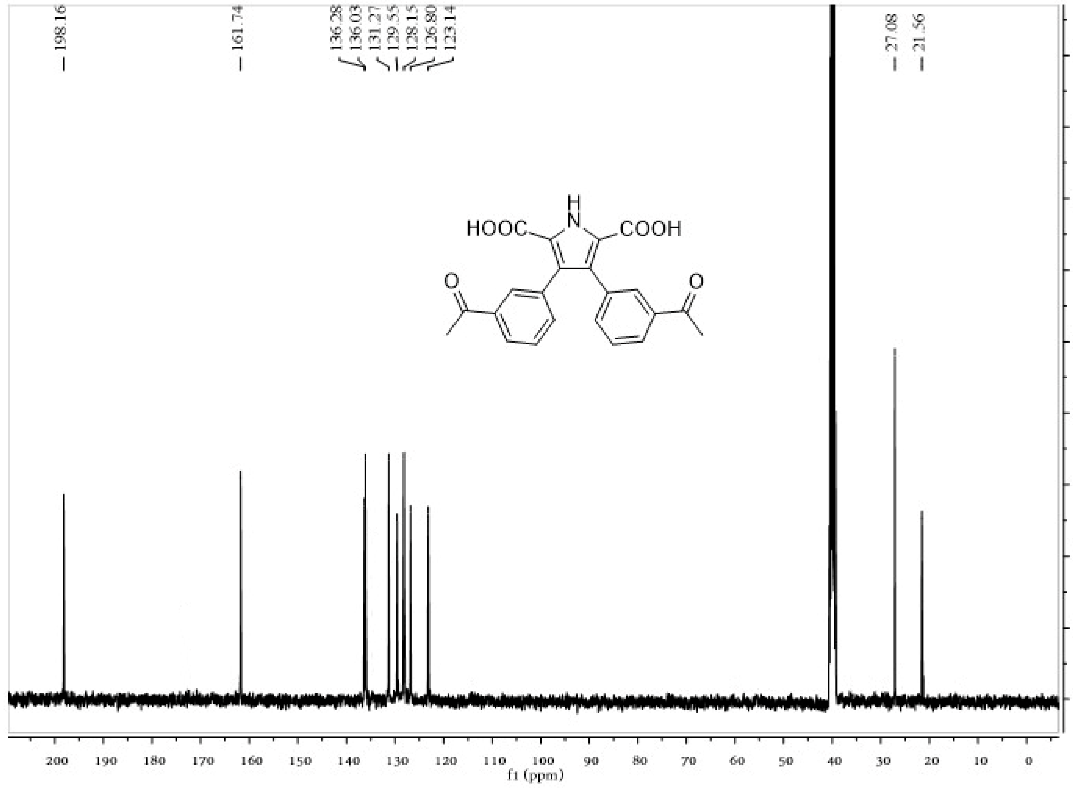

Figure S112. ^{13}C NMR spectrum of **8k**

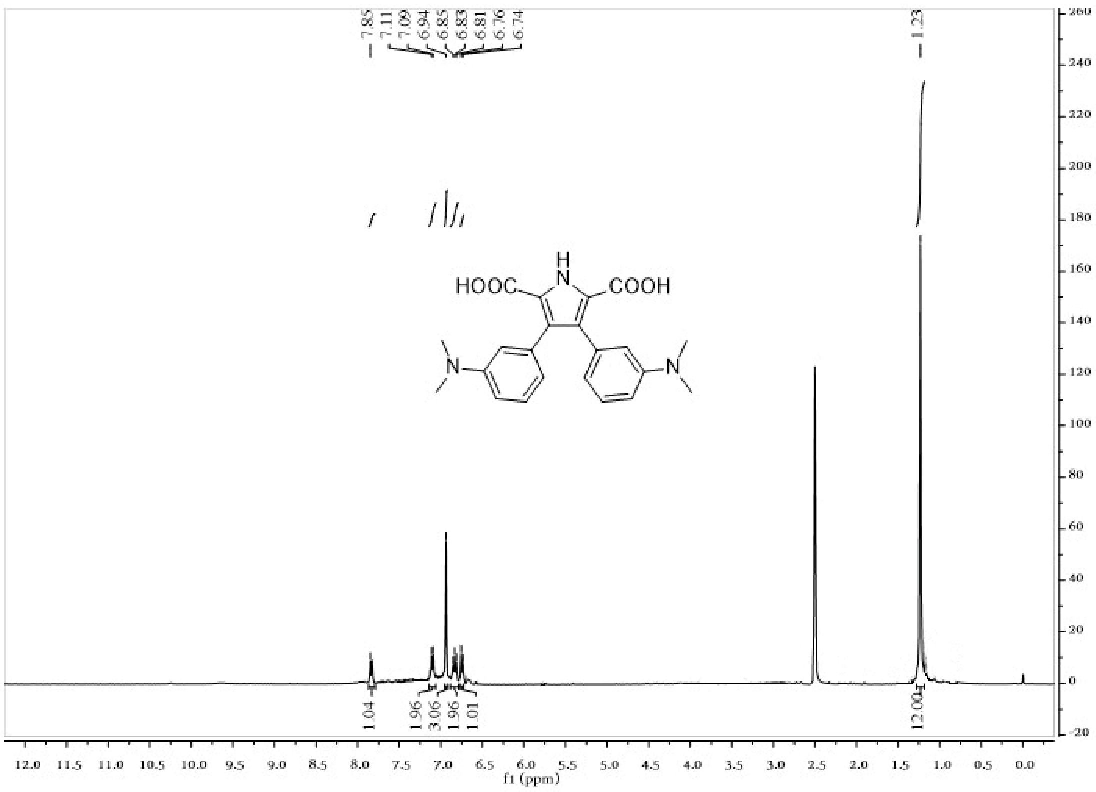

Figure S113. ^{1}H NMR spectrum of **8l**

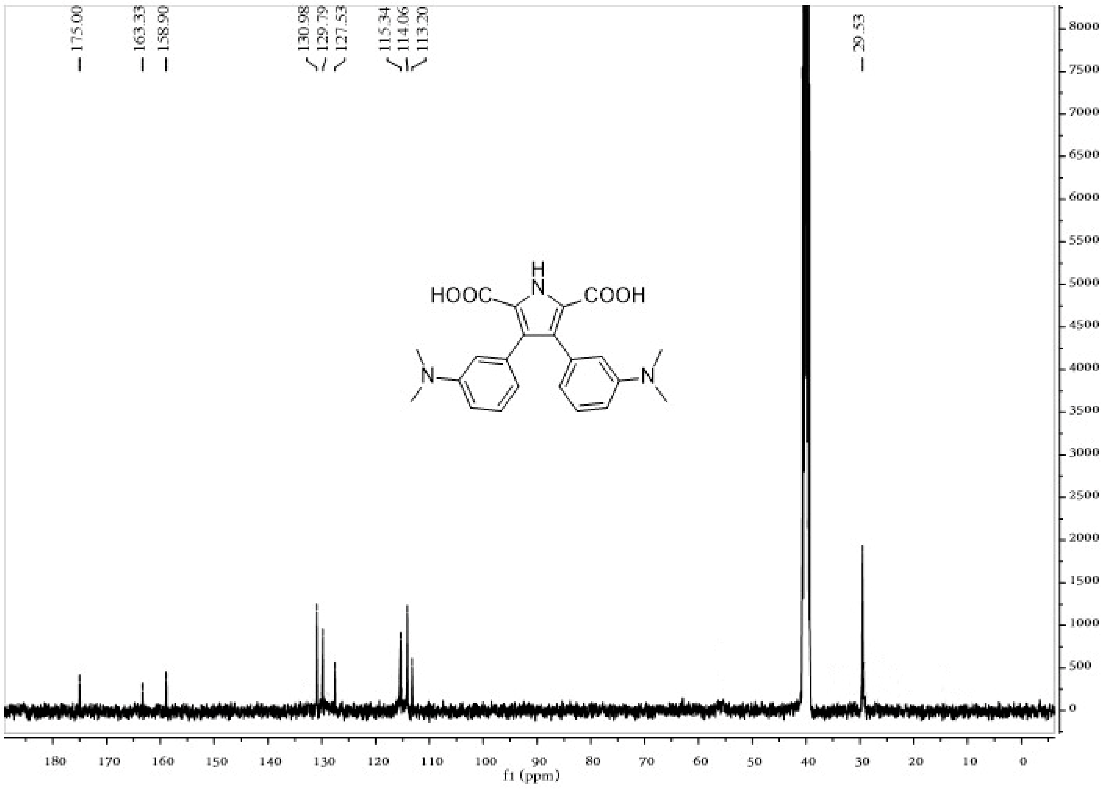

Figure S114. ^{13}C NMR spectrum of **8l**

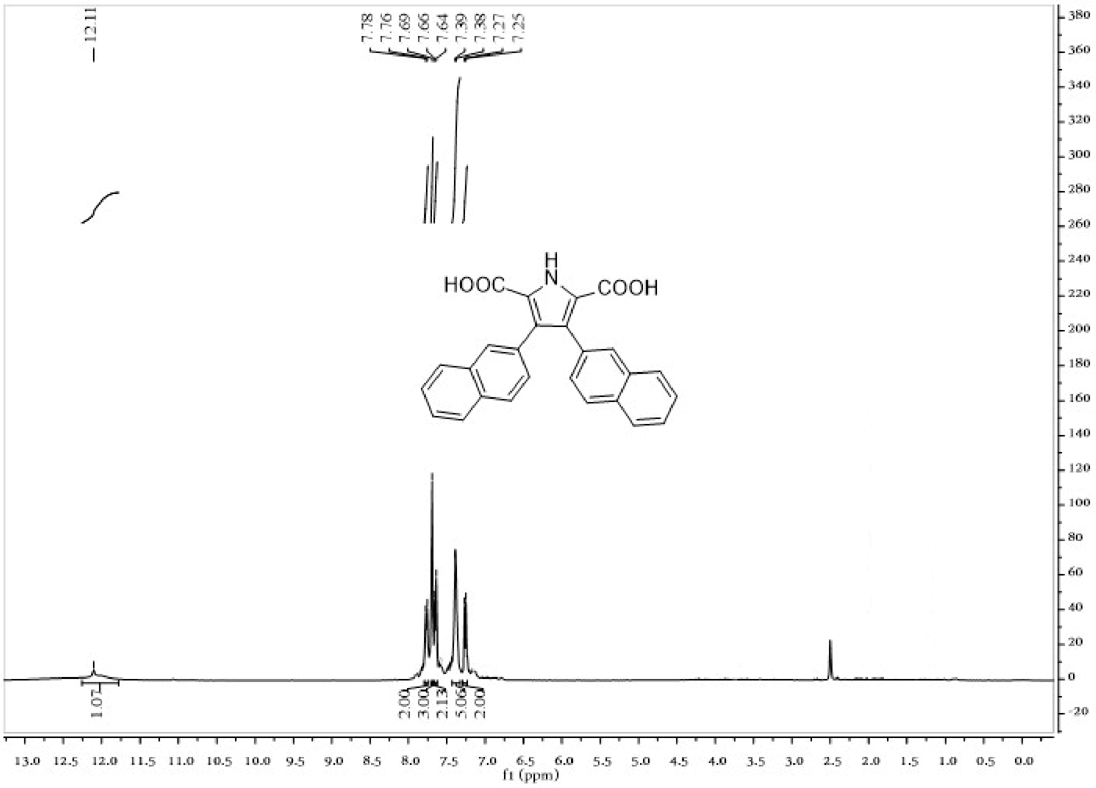

Figure S115. ^{1}H NMR spectrum of **8m**

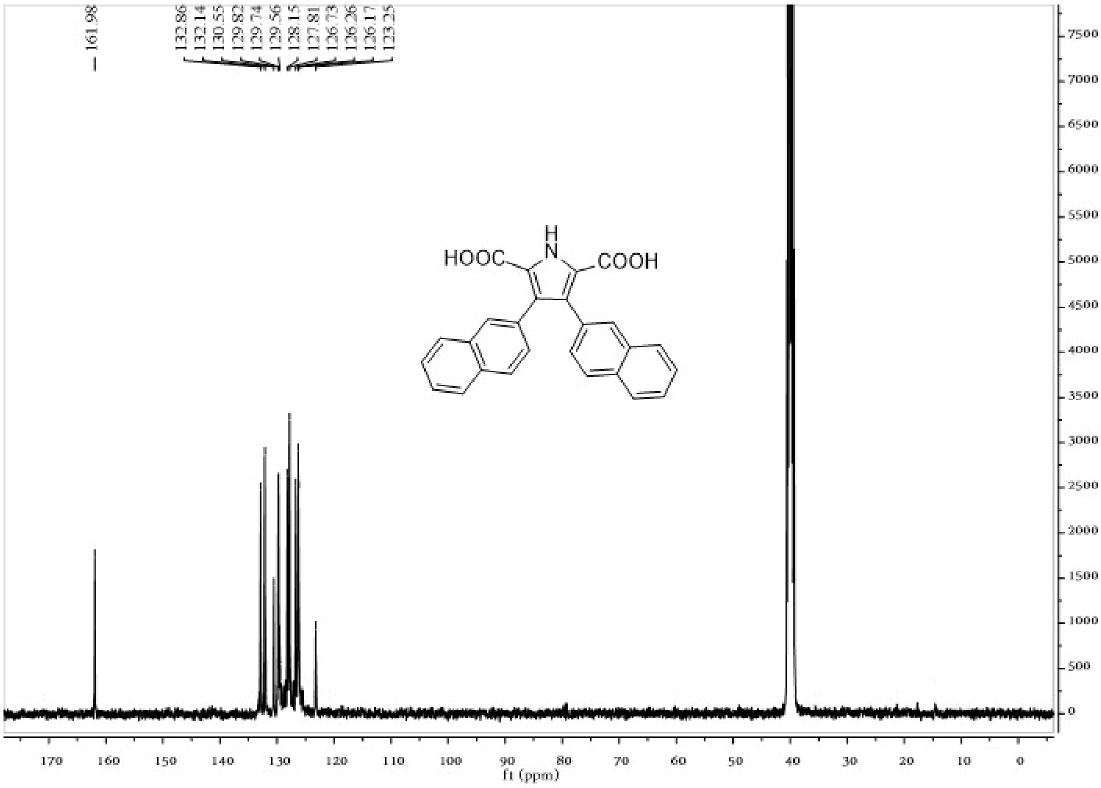

Figure S116. ^{13}C NMR spectrum of **8m**

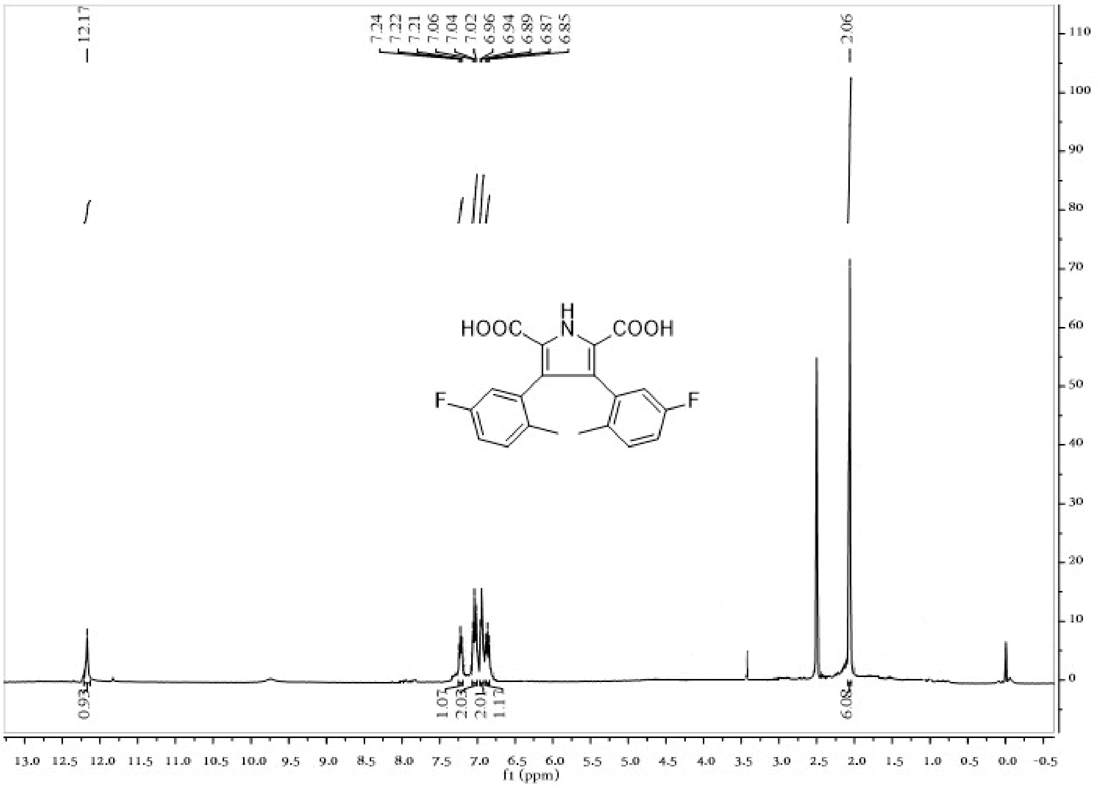

Figure S117. ^{1}H NMR spectrum of **8n**

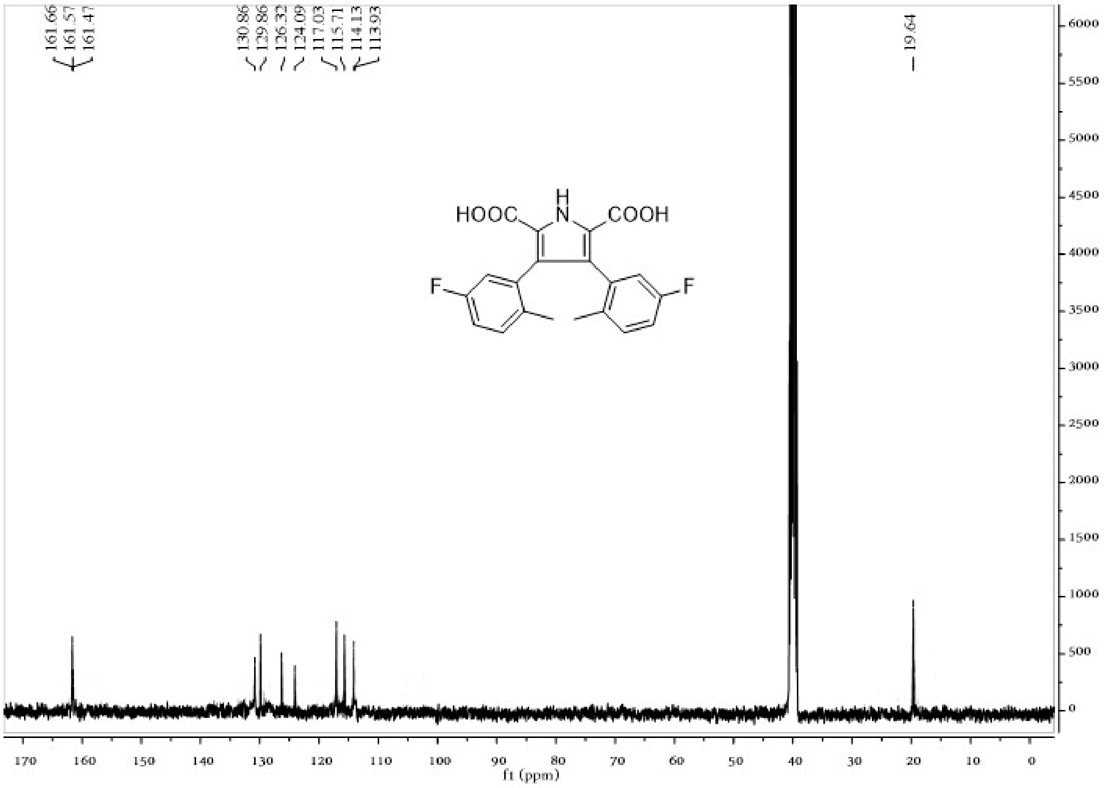

Figure S118. ^{13}C NMR spectrum of **8n**

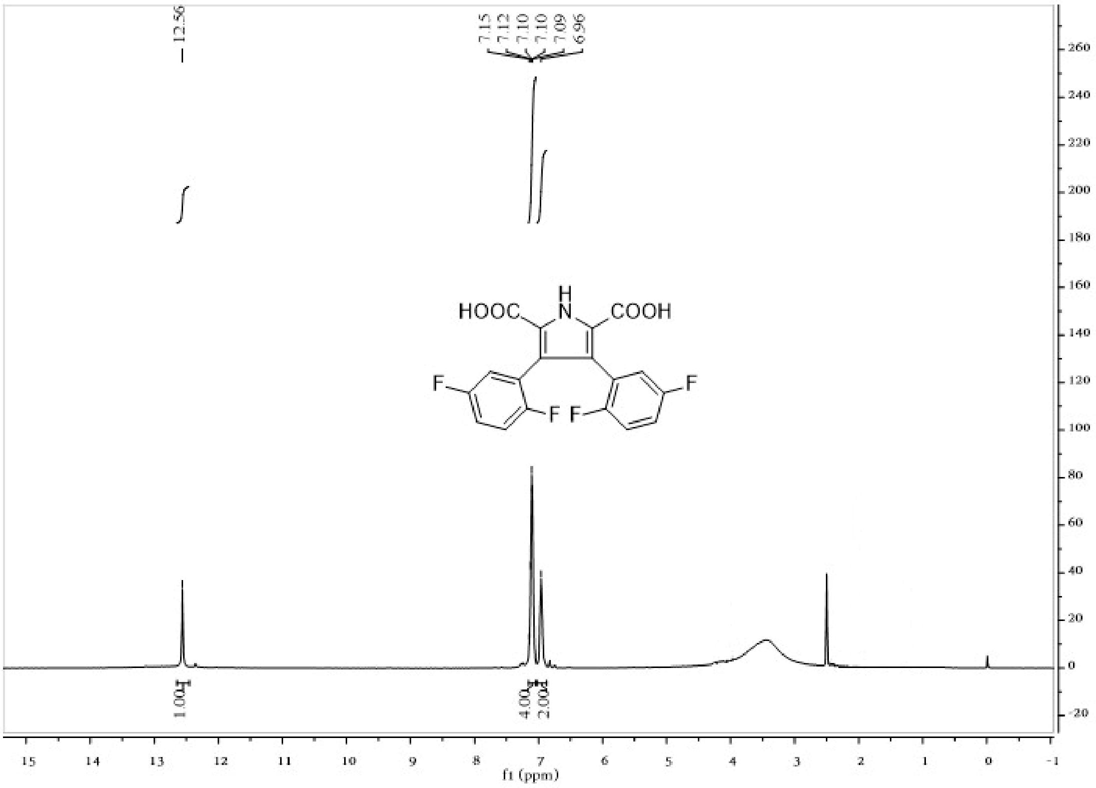

Figure S119. ^{1}H NMR spectrum of **8o**

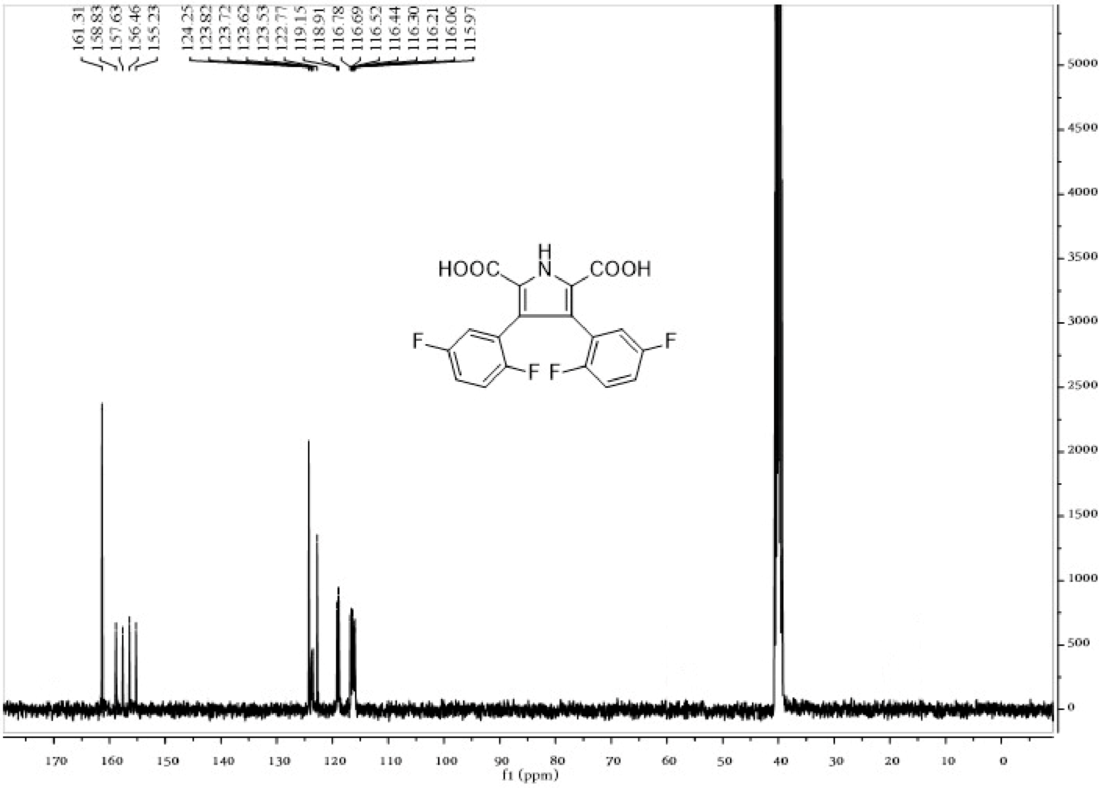

Figure S120. ^{13}C NMR spectrum of **8o**

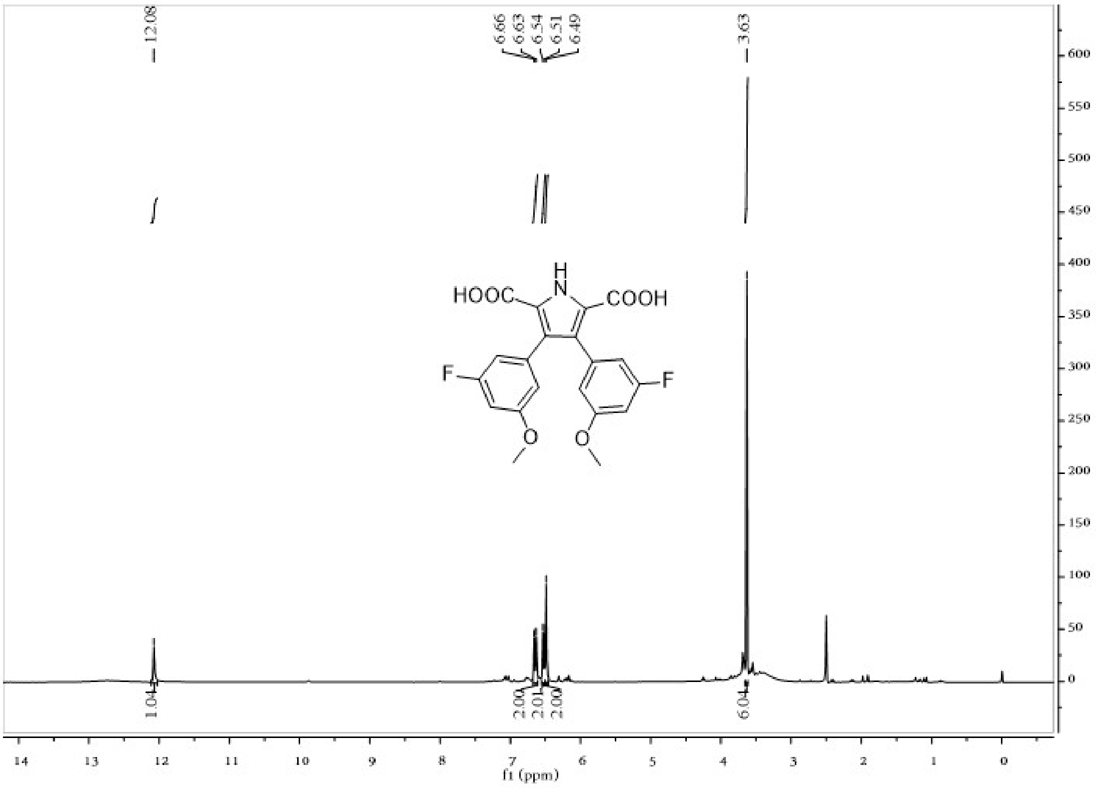

Figure S121. ^{1}H NMR spectrum of **8p**

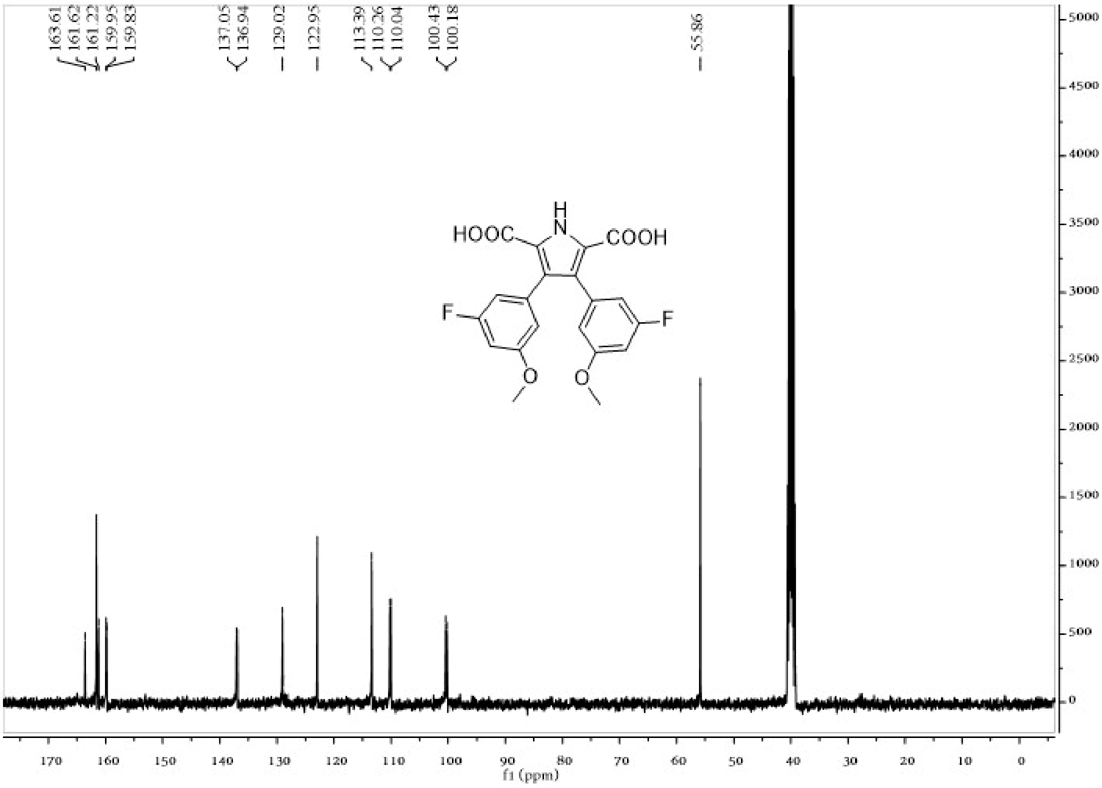

Figure S122. ^{13}C NMR spectrum of **8p**

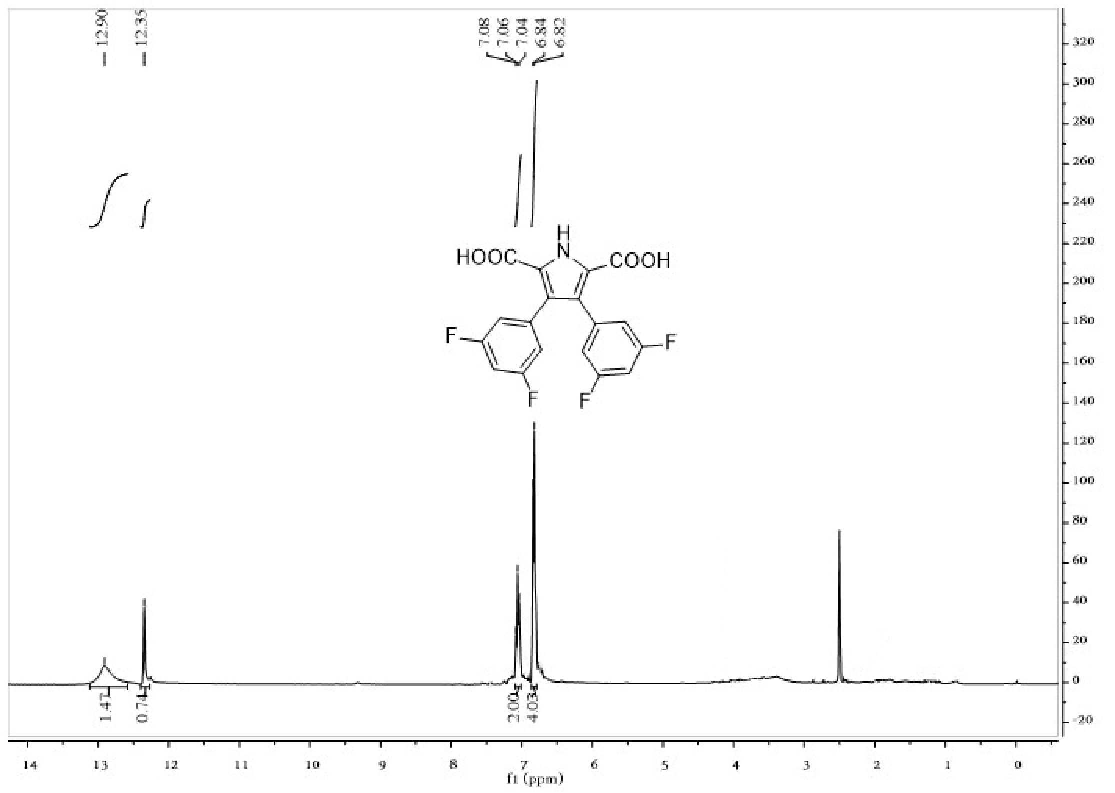

Figure S123. ^{1}H NMR spectrum of **8q**

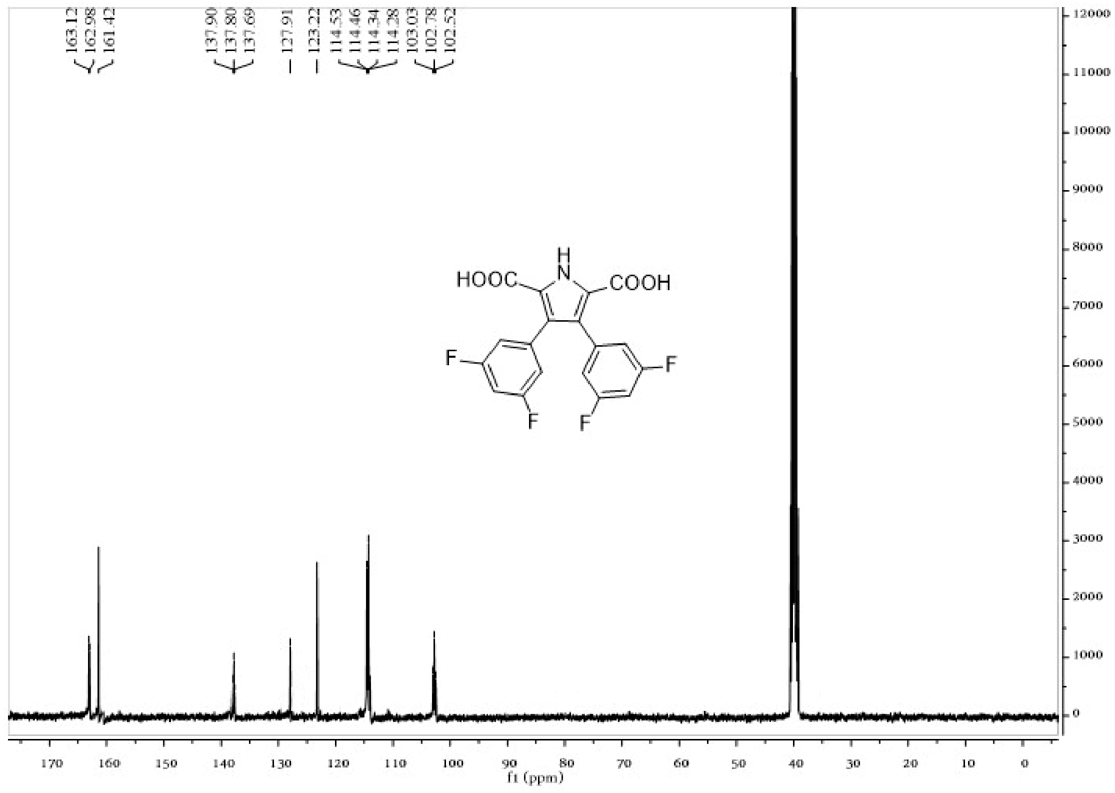

Figure S124. ^{13}C NMR spectrum of **8q**

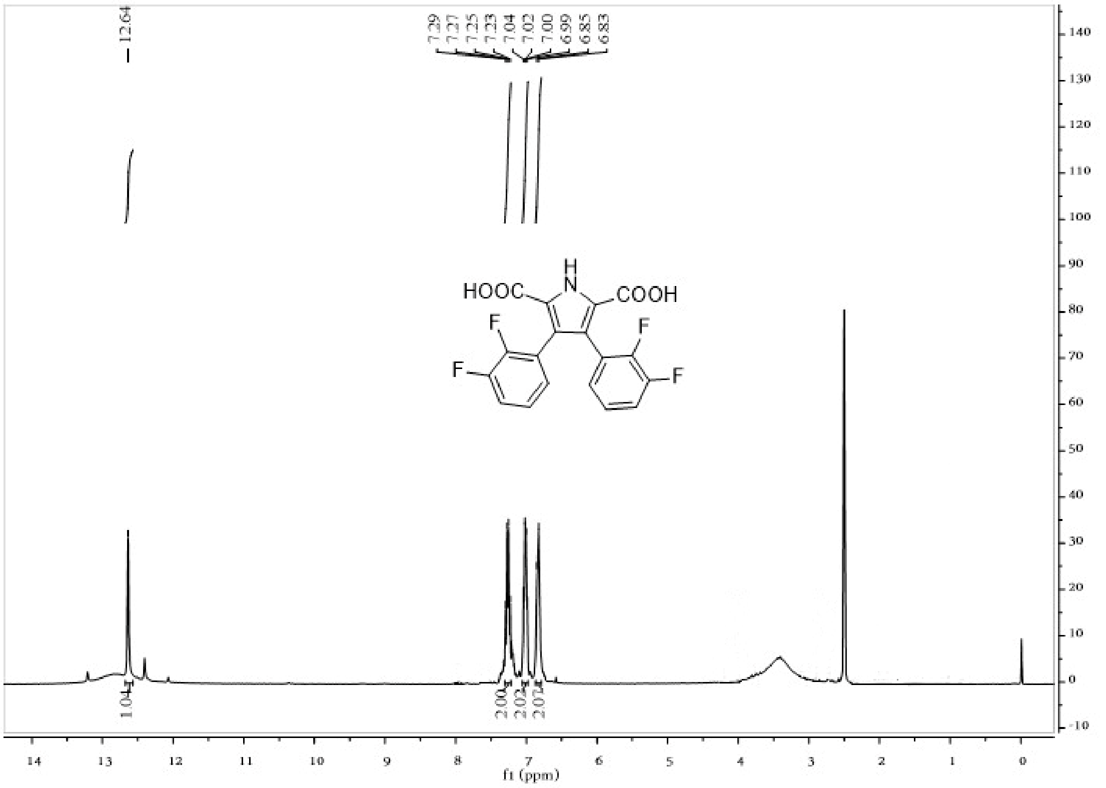

Figure S125. ^{1}H NMR spectrum of **8r**

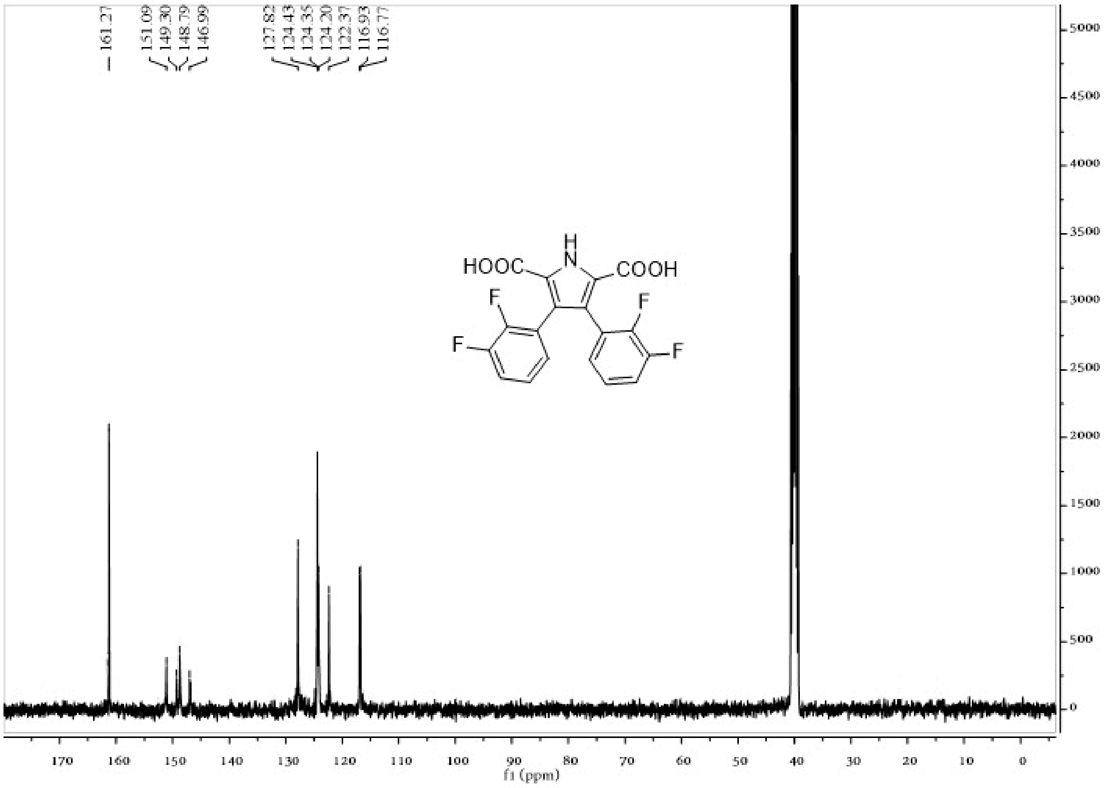

Figure S126. ^{13}C NMR spectrum of **8r**

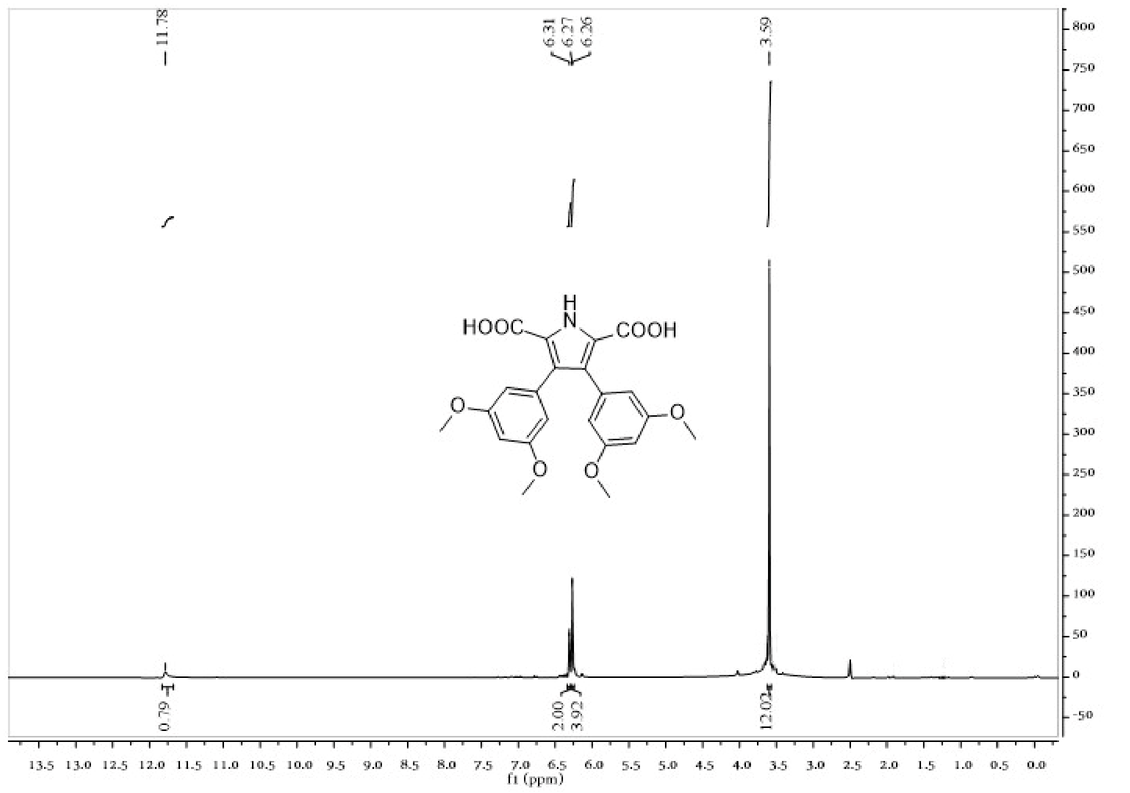

Figure S127. ¹H NMR spectrum of **8s**

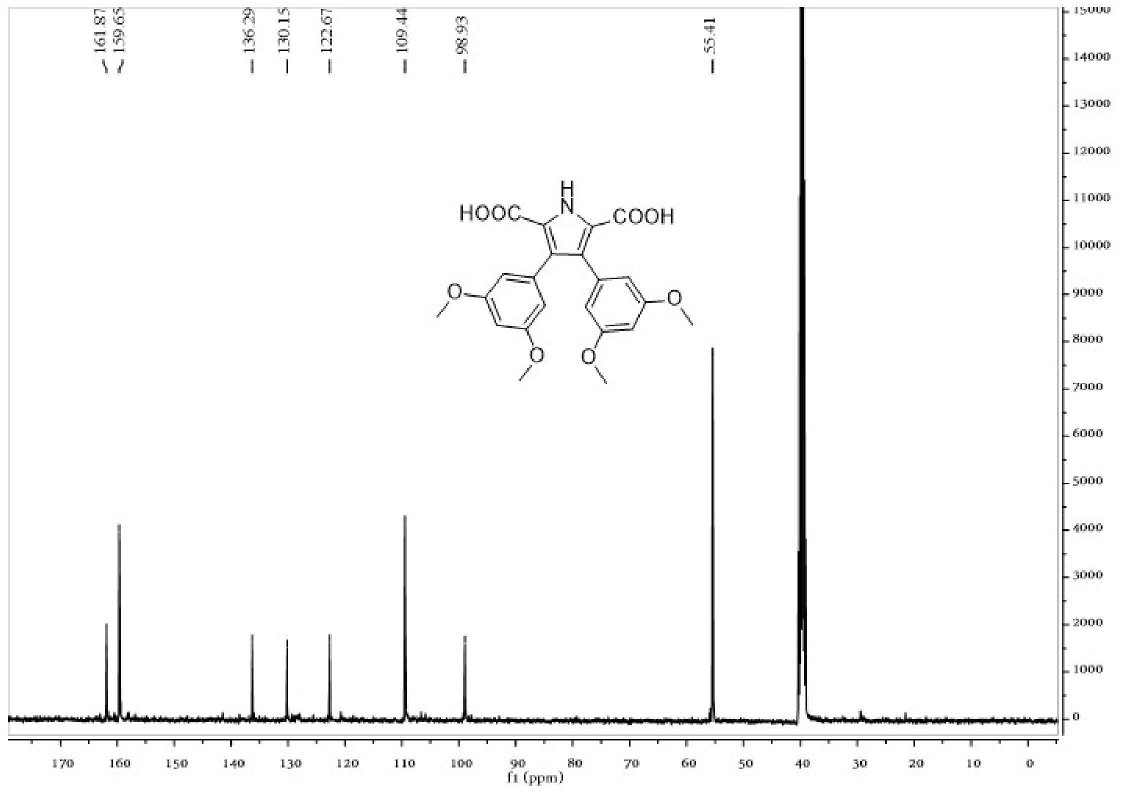

Figure S128. ¹³C NMR spectrum of **8s**
